# Review of Inorganic Separator Engineering for Next‐Generation Lithium–Sulfur Batteries: Compromise or Cornerstone?

**DOI:** 10.1002/adma.74605

**Published:** 2026-08-20

**Authors:** Yuting Qin, Tianyi Wang, Xingyu Wang, Kaifu Xu, Yuzhou Wang, Chengyin Wang, Bing Sun, Guoxiu Wang

**Affiliations:** ^1^ School of Chemistry & Materials Yangzhou University Yangzhou Jiangsu China; ^2^ Department of Mechanical Engineering National University of Singapore Singapore Singapore; ^3^ Centre for Clean Energy Technology School of Mathematical and Physical Science Faculty of Science University of Technology Sydney Sydney New South Wales Australia

**Keywords:** interfacial reaction kinetics, Li metal anodes, lithium–sulfur batteries, metal compound‐based separator, polysulfide shuttling, separator engineering

## Abstract

Lithium–sulfur (Li–S) batteries are promising next‐generation energy‐storage systems, but their practical application remains limited by polysulfide shuttling, sluggish redox kinetics, and interfacial instability. Separator engineering offers an effective route to regulate sulfur chemistry. This review summarizes metal compound‐based separators as active interfacial architectures that promote polysulfide adsorption and catalytic conversion while regulating ion and electron transport. To evaluate diverse separator systems, we propose a Practical Relevance Index (PRI)‐guided framework that integrates intrinsic electrochemical improvements with system‐level constraints. A localized figure of merit is further introduced to interpret performance trends without direct cross‐study ranking. Recent advances in metal oxides, sulfides, nitrides, carbides, and MXene‐based separators are discussed, with emphasis on heterostructures, defect engineering, electronic‐structure regulation, and atomic‐scale design. Particular attention is given to constraint‐aware strategies that connect interfacial chemistry with practical energy density. Finally, major challenges and future directions are outlined, including reaction‐pathway regulation, operando characterization, system integration, and scalable manufacturing. This review provides a unified framework for designing practical, high‐energy‐density Li–S batteries.

## Graphical abstract

Inorganic compound‐modified separators transform lithium–sulfur batteries from passive polysulfide confinement to active reaction‐pathway regulation. A Practical Relevance Index (PRI)‐guided framework bridges interfacial chemistry of inorganic separators with practical constraints, establishing unified design principles for scalable, high‐energy‐density lithium–sulfur batteries.

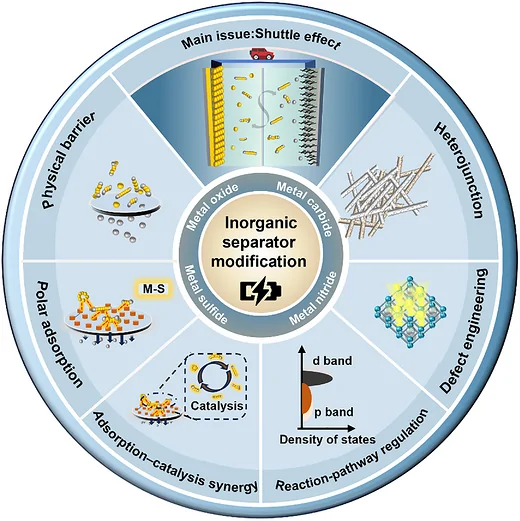

## 1 Introduction

### 1.1 From Mechanistic Superiority to System‐Level Constraints: Rethinking the Role of Inorganic Separators

Lithium–sulfur (Li–S) batteries have been widely regarded as one of the most promising next‐generation energy storage systems due to their extremely high theoretical energy density (∼2600 Wh kg^−1^), low cost, and environmental compatibility of sulfur cathodes [1, 2, 3]. Despite these advantages, practical deployment remains severely hindered by sluggish reaction kinetics, rapid capacity fading, and poor cycling stability under realistic operating conditions [4].

The fundamental origin of these limitations lies in the complex multistep conversion between elemental sulfur and lithium sulfides, accompanied by the formation and dissolution of soluble lithium polysulfides (Li_2_S_n_, 2 ≤ *n* ≤ 8) [5, 6]. These species induce the well‐known “polysulfide shuttle effect,” which results in active material loss, severe electrode polarization, and parasitic reactions on the lithium metal anode [7, 8, 9, 10]. However, recent studies have demonstrated that the shuttle phenomenon is not merely a diffusion‐controlled process, but rather a coupled interfacial reaction network involving electrolyte, cathode, anode, and separator domains [11, 12, 13]. Against this background, separator engineering has evolved from a passive physical barrier to an active interfacial regulator of sulfur electrochemistry [14, 15, 16]. Early strategies relied on dense coatings [17, 18, 19] or porous carbon layers [20, 21, 22] to suppress polysulfide migration through physical confinement. Although partially effective, such approaches fail to regulate polysulfide conversion kinetics and interfacial redox reactions. With deeper mechanistic understanding, separator design has progressively shifted toward functional interfaces capable of adsorption, catalytic conversion, and interfacial charge modulation [23]. A central and unresolved question in this field is therefore whether inorganic separator engineering should be regarded as a “cornerstone” enabling high‐performance Li–S batteries or merely a “compromise” solution constrained by interfacial and architectural trade‐offs. This question forms the conceptual axis of this review. From a mechanistic perspective, inorganic separators—particularly metal compound‐based systems—offer unique advantages including tunable electronic structures, variable valence states, and intrinsic polarity [24, 25]. Interfacial nanofibrous frameworks have been shown to regulate Li^+^ flux, stabilize interfacial chemistry, and suppress dendrite growth under low N/P full‐cell configurations, enabling improved cycling stability in practical lithium metal batteries [26]. These properties enable synergistic adsorption‐catalysis behavior and more effective regulation of polysulfide chemistry, suggesting a potential “cornerstone” role in redefining separator functionality. However, from a device‐level perspective, these systems simultaneously introduce non‐negligible trade‐offs, including increased interfacial resistance, additional inactive mass, reduced ionic transport efficiency, and limited scalability of functional coatings. Recent studies in organic electronics demonstrate that shear‐force fields during solution processing can regulate polymer crystallinity and phase separation kinetics, indicating that device performance is strongly governed by kinetic rather than purely thermodynamic factors [27]. These constraints become particularly significant under practical conditions such as high sulfur loading, lean electrolyte conditions, and pouch‐cell configurations, thereby positioning inorganic separators as a potential “compromise” in real‐world applications. In practical Li–S pouch cells, these limitations are further exacerbated under lean electrolyte conditions, where dead lithium accumulation and electrolyte depletion are significantly accelerated. Recent studies have demonstrated that regenerative redox mediators can partially restore inactive lithium in pouch‐cell systems, highlighting the importance of interfacial reaction regulation under practical operating conditions [28]. Therefore, inorganic separator engineering should be understood as a condition‐dependent duality rather than a binary classification, which motivates a systematic re‐evaluation of its design principles and performance metrics.

To clarify the development logic of this field, this review organizes the discussion around progressively connected analytical frameworks. As shown in Figure 1a,b, bibliometric analysis reveals a clear transition from polymer‐based separator modification toward inorganic and metal compound‐based systems. This shift reflects not only a change in material preference, but also a deeper paradigm transition from physical blocking strategies to electronically and chemically active interfacial regulation [29, 30, 31, 32]. Importantly, this evolution directly motivates the central question of this review: whether such functional enhancements can truly overcome system‐level constraints. The bibliometric methodology underlying this analysis is detailed in Supporting Information (Section S1).

**FIGURE 1:**
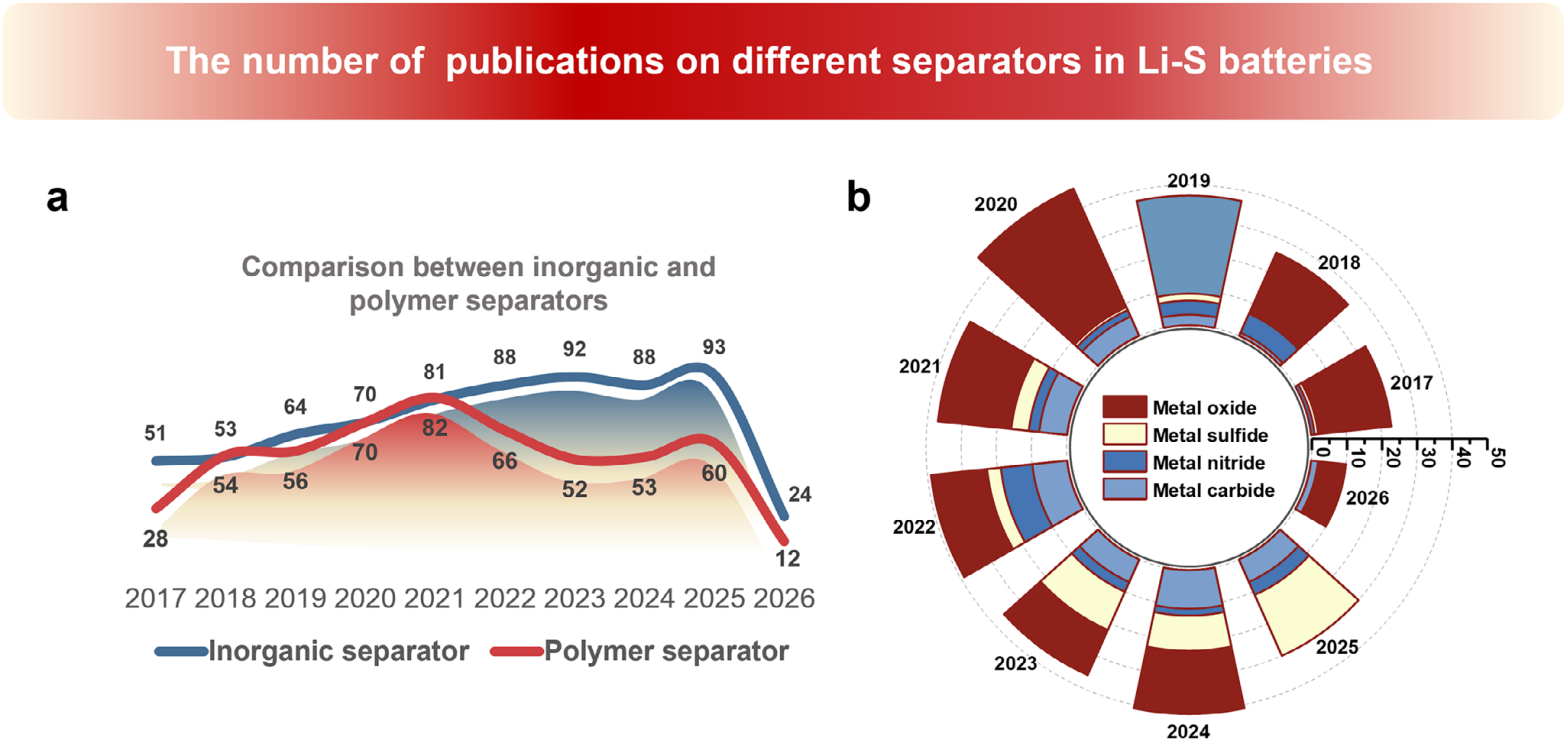
Statistical evolution of metal compound‐based separators in battery systems and the development of inorganic material applications in Li–S batteries. (a) The comparison of scientific‐publications based on the search keywords “inorganic separator” & “polymer separator” and “lithium sulfur battery”, as retrieved through the Web of Science database, covering data reported in the past ten years (2017–April, 2026). (b) The comparison of scientific‐publications based on the search keywords “metal oxide” and “metal sulfide” & “metal nitride” and “metal carbide”, as retrieved through the Web of Science database, covering data reported in the past ten years (2017–April, 2026).

Building upon this macroscopic evolution, the internal development of inorganic separator engineering can be decomposed into two complementary dimensions: temporal mechanistic evolution and knowledge structure organization. Figure 2 captures the time‐resolved evolution of separator engineering strategies, illustrating a progression from adsorption‐dominated mechanisms [33, 34, 35] to adsorption‐catalysis synergy [36, 37], and further toward heterostructure engineering [38, 39], defect modulation [40, 41], and built‐in electric field design [42, 43]. This evolution reflects a continuous strengthening of interfacial regulation capability [44, 45, 46, 47]. However, this temporal perspective alone is insufficient.

**FIGURE 2:**
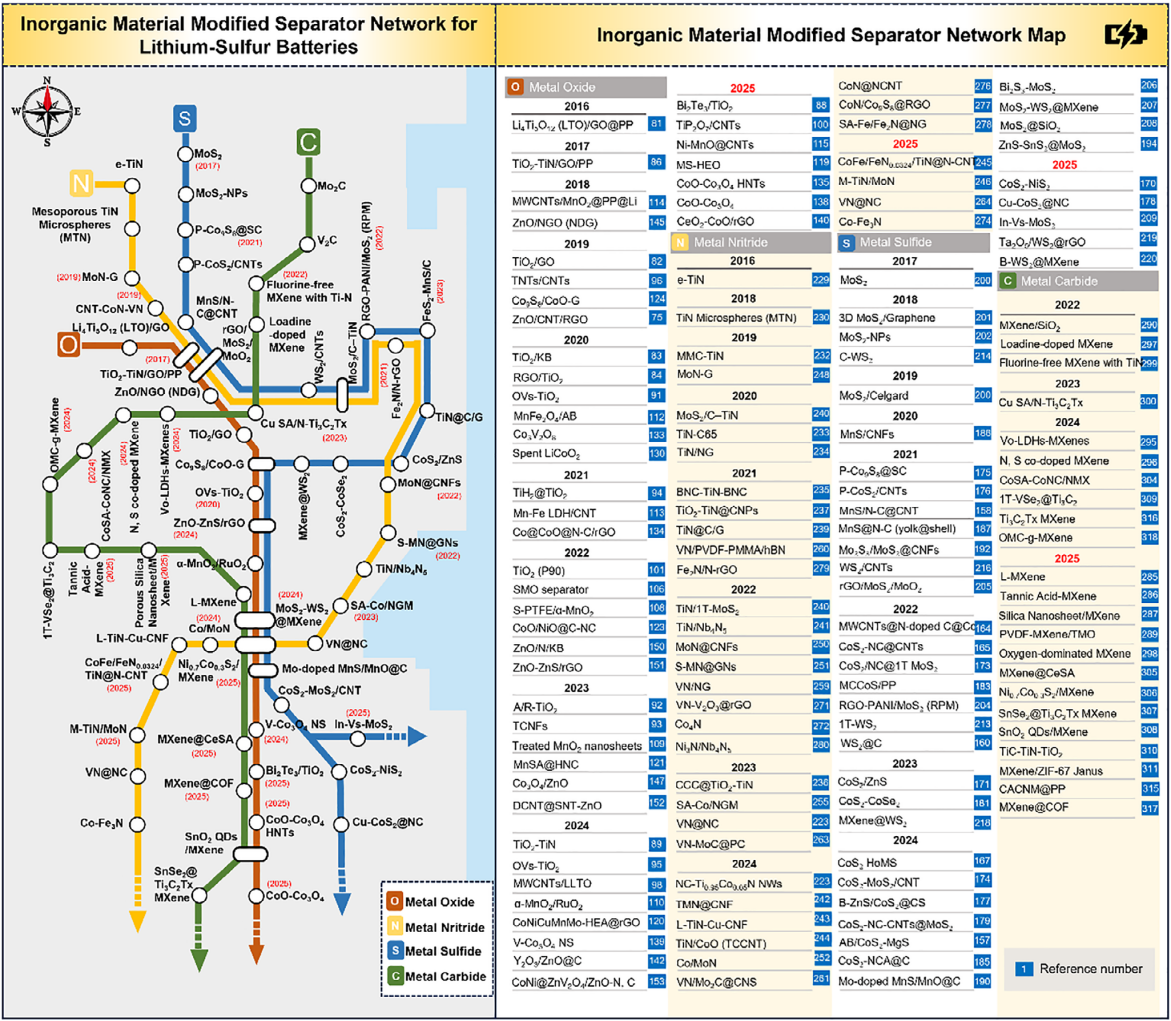
Relevant breakthroughs have been achieved in the modification of Li–S battery separators using metal compounds over the past decade (2017–2026).

As revealed in Figure 2, many mechanistic strategies overlap extensively across different material systems, suggesting that separator design cannot be fully understood from a chronological viewpoint. In contrast, Figure 3a and Figure S1 provides a structural perspective based on keyword co‐occurrence analysis. It reveals that modern separator research is governed by a coupled framework of adsorption, catalysis, conversion, and charge transport, rather than isolated mechanisms [48, 49, 50, 51, 52, 53]. Furthermore, different material classes exhibit distinct functional emphases, with oxides favoring adsorption [54, 55], while sulfides, nitrides, and carbides are more strongly associated with catalytic activity and charge transport regulation [56, 57, 58]. Beyond the material‐specific differentiation shown in Figures 3a, a higher‐level integration of functional mechanisms is presented in Figure 3b. This schematic consolidates the distinct roles of metal compounds into a unified framework centered on adsorption, catalytic conversion, and charge transport regulation. Unlike the previous subfigures that emphasize categorical differences among oxides, sulfides, nitrides, and carbides, Figure 3b highlights their convergence toward a shared design principle. This principle relies on d‐block electronic structures to simultaneously regulate polysulfide binding strength, redox kinetics, and interfacial electron transfer. In this context, non‐metal components primarily function as structural and electronic modulators, whereas metal centers serve as the dominant active sites governing sulfur redox behavior. Together, Figures 2 and 3 establish a dual‐perspective understanding of the field, describing both its temporal evolution and its underlying conceptual architecture. While Figure 3 highlights the mechanistic organization of separator design, practical implementation is strongly governed by fabrication‐induced constraints. In real battery systems, separator performance is determined not only by intrinsic material properties, but also by structural parameters such as thickness, porosity, electrolyte uptake, and interfacial contact resistance.

**FIGURE 3:**
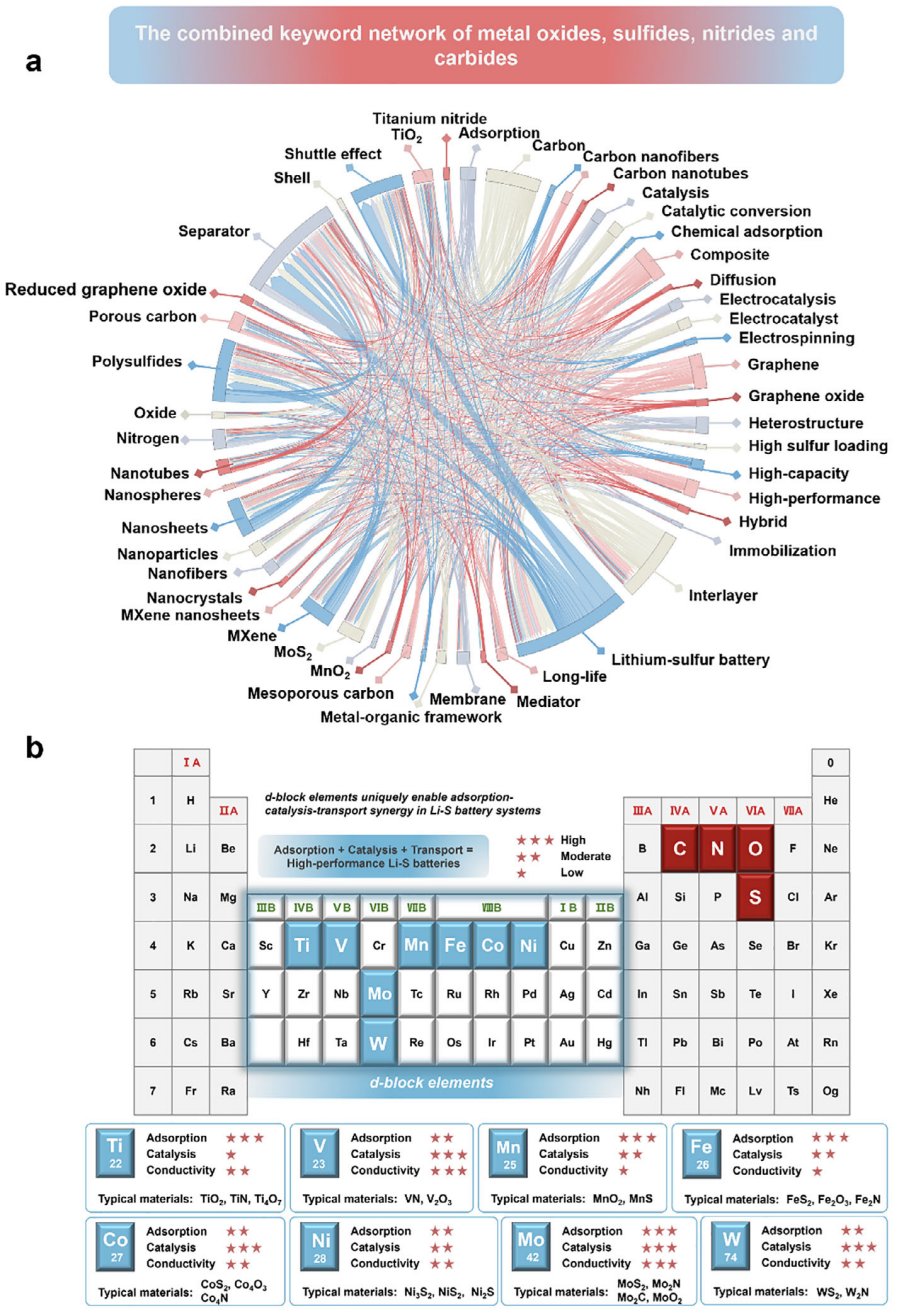
A network visualization graph of high‐frequency keywords “separator”, “inorganic materials”, and “Li–S batteries” during the recent 5 years (2022–2026) (data collected from Web of Science and analyzed by VOS‐viewer). (a) Combined keyword network of metal oxides, sulfides, nitrides, and carbides, revealing the overall research framework centered on polysulfide adsorption, catalytic conversion, and separator engineering. (b) Schematic illustration of the integrated functional evolution of metal compounds for Li–S battery separator modification. d‐block element‐centered systems act as the dominant active centers, enabling strong chemical adsorption, efficient catalytic conversion, and enhanced charge transport, owing to their flexible electronic configurations and multivalent characteristics. Meanwhile, nonmetallic components mainly contribute to polarity modulation and electronic structure tuning. The figure highlights the synergistic “adsorption‐catalysis‐transport” mechanism and reveals the material design trend toward multifunctional and heterostructured systems.

While material chemistry plays a central role in separator performance, fabrication strategy critically determines how functional components are integrated into practical architectures. Fabrication methods can be broadly categorized into solution‐based coating, electrospinning, and self‐assembly (Figure 4a). Solution‐based coating methods, including dip‐coating, doctor blade coating, spray‐coating, and vacuum filtration, are widely adopted due to their simplicity, scalability, and compatibility with existing battery manufacturing processes. These approaches typically produce thin and compact functional layers that ensure mechanical stability and effective suppression of polysulfide diffusion, but their limited porosity may restrict electrolyte uptake and ionic transport. As summarized in Figure 4c–e, coating‐based separators generally exhibit lower thickness and moderate wettability and ionic conductivity, which support stable cycling but limit rate capability [59, 60, 61].

**FIGURE 4:**
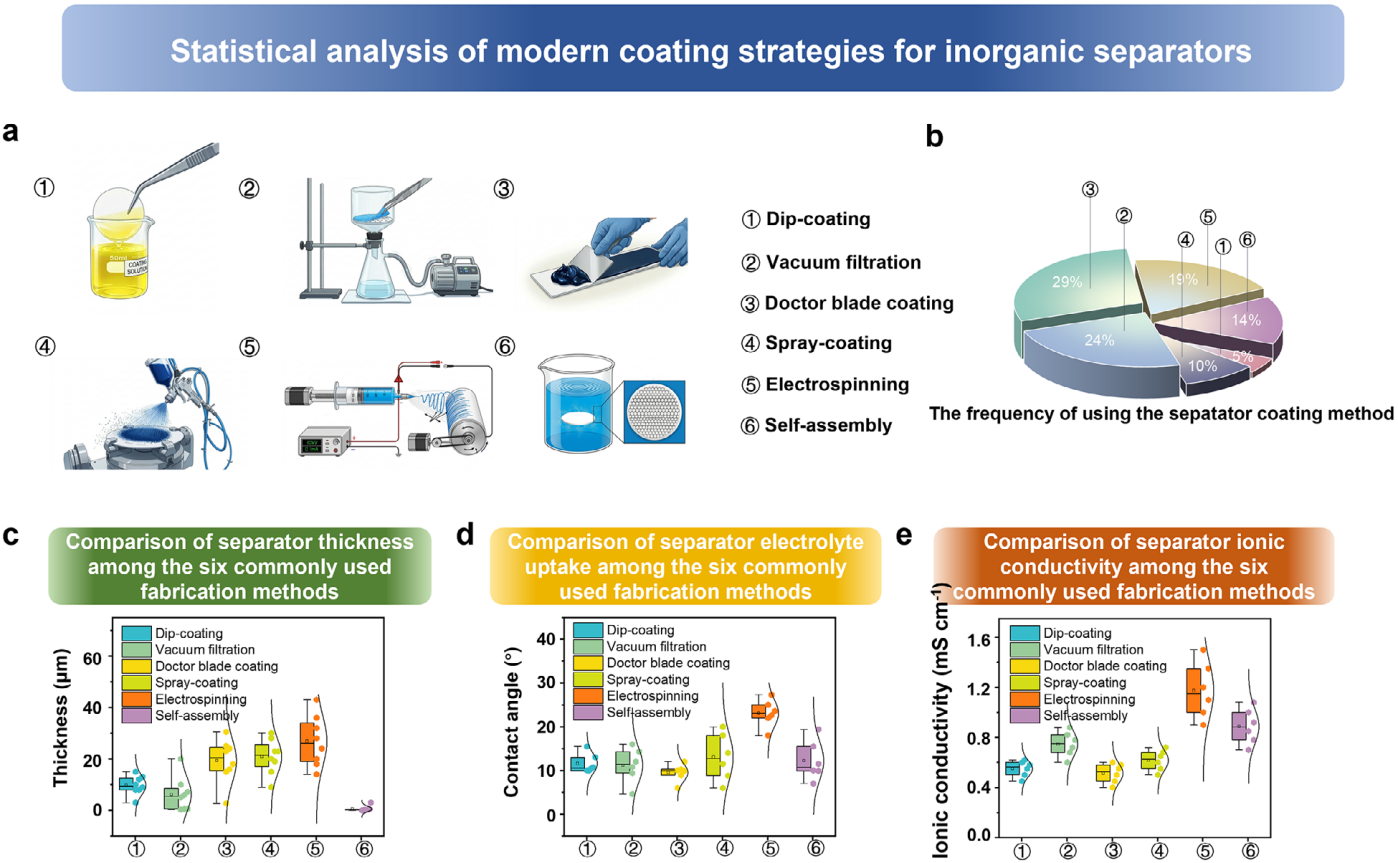
Fabrication strategies for functionalized separators and their impact on structural and electrochemical properties. (a) Schematic illustration of representative preparation methods, including dip‐coating, vacuum filtration, doctor blade coating, spray‐coating, electrospinning, and self‐assembly. (b) Relative frequency of these fabrication methods based on literature analysis. (c–e) Comparative boxplot analysis of separator (c) thickness, (d) electrolyte uptake, and (e) ionic conductivity for separators prepared using different fabrication strategies. The number of data points (*N*) for each method is: thickness (*N* = 48), electrolyte uptake (*N* = 36), ionic conductivity (*N* = 36). No statistical significance tests were performed because the original studies used different separator substrates, electrolytes, and measurement protocols; direct *p*‐value comparisons would be misleading. The figure is intended to show overall distribution trends rather than causal differences. The boxplots represent the median, interquartile range (IQR), and full data range (whiskers). Data were collected from different literature sources under varying experimental conditions (e.g., separator substrates, electrolyte compositions, and measurement protocols). Therefore, no statistical significance tests were performed, and the comparisons are intended to illustrate general distribution trends rather than statistically significant or causal differences.

In contrast, electrospinning enables highly porous and interconnected fibrous architectures with large surface area and enhanced electrolyte affinity. These structures facilitate rapid ion transport and provide abundant active sites for polysulfide adsorption and conversion. However, increased thickness and structural complexity may reduce mechanical robustness and limit scalability. Consistently, electrospun separators show higher thickness, improved wettability, and enhanced ionic conductivity compared with coating‐based systems, resulting in improved rate performance but potential challenges in long‐term stability and large‐scale application [60, 61, 62]. Self‐assembly strategies construct ordered or densely packed nanostructures through interfacial interactions. These architectures typically offer strong interfacial adhesion and simultaneous improvement in polysulfide confinement and catalytic activity, but their complex fabrication and limited scalability hinder practical use. As reflected in Figure 4c–e, self‐assembled separators exhibit intermediate structural and electrochemical characteristics, balancing stability and transport properties [63, 64, 65, 66].

The statistical distribution in Figure 4b further shows that coating‐based methods such as doctor blade coating and vacuum filtration dominate current research, whereas electrospinning and self‐assembly are less frequently reported due to higher processing complexity. This distribution highlights a fundamental trade‐off between scalability and structural tunability. Overall, fabrication strategy governs separator thickness, wettability, and ionic conductivity, thereby determining the balance between structural robustness and ion transport capability. The data are compiled from multiple literature sources under varying experimental conditions and are intended to reflect general trends rather than strictly comparable quantitative differences.

Despite numerous studies on inorganic separator modifiers for Li–S batteries, existing reviews largely emphasize material classification and performance comparison [67, 68], lacking a systematic view of the separator as an active reaction interface and the evolution of design paradigms. This work establishes a multidimensional framework (metal oxides, sulfides, nitrides, and 2D systems) mapping trade‐offs in polysulfide adsorption, catalysis, charge transport, and robustness (Figure 5a). We reveal an evolutionary shift from passive adsorption to interface‐driven regulation via heterostructures and electronic modulation (Figure 5b), adopt a mechanistic perspective on sulfur redox steps, Li_2_S dynamics, and charge redistribution (Figure 5c), and evaluate engineering feasibility, synthesis complexity, and scalability (Figure 5d). Integrating material chemistry, interfacial kinetics, and device‐level considerations, we summarize key principles for rational separator design in high‐performance Li–S batteries. In this context, this review primarily focuses on transition‐metal compound‐based separator materials as the main functional class of inorganic separator modifiers in Li–S batteries, while other inorganic or metalloid systems are briefly discussed for contextual completeness.

**FIGURE 5:**
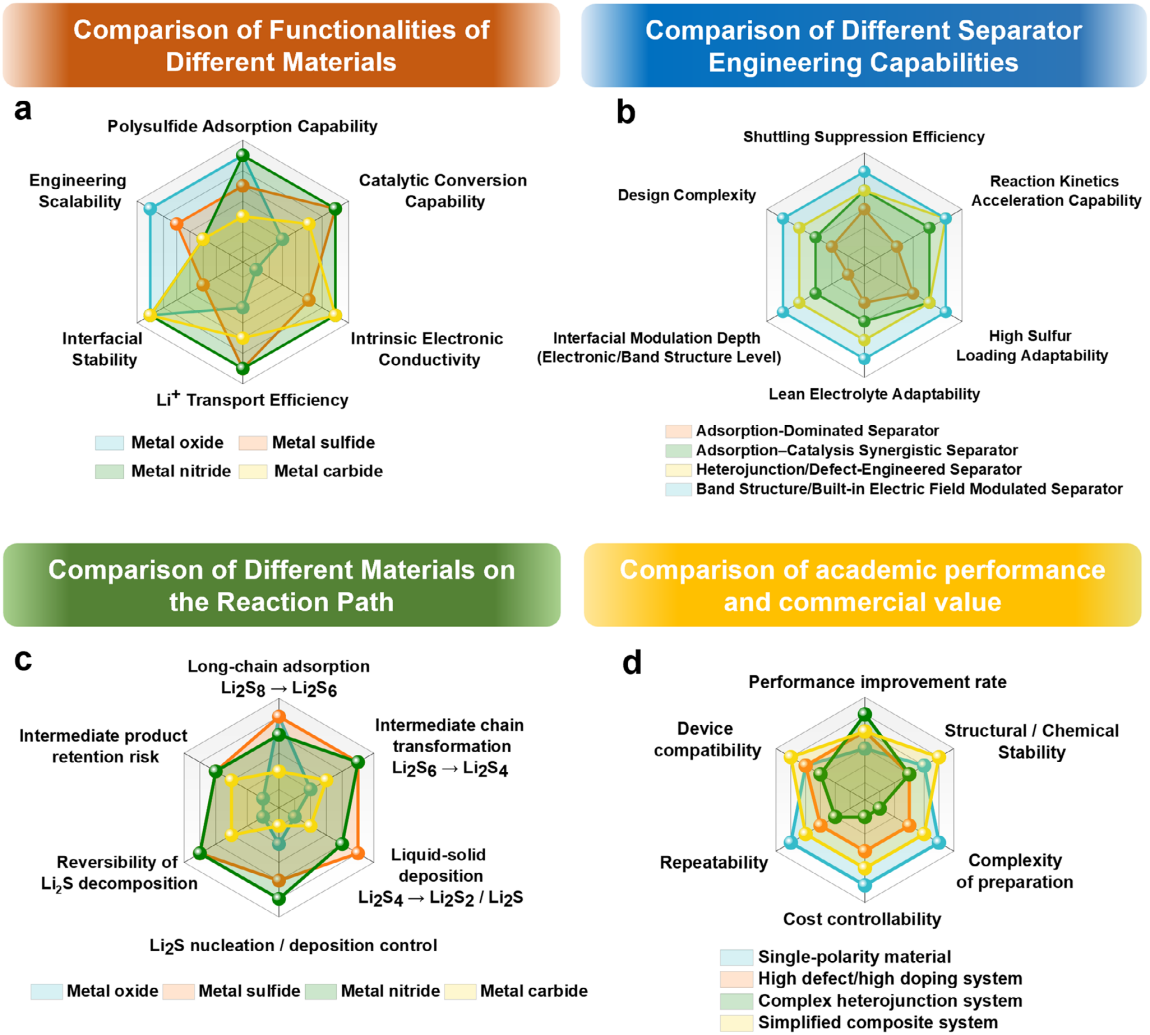
(a) Comparison of functionalities of different inorganic material systems, including polysulfide adsorption capability, catalytic conversion ability, charge transport regulation, and structural stability. (b) Schematic illustration of the evolution of separator engineering paradigms, from passive adsorption to interface‐driven regulation strategies. (c) Comparison of the roles of different material systems in regulating sulfur redox reaction pathways, including intermediate conversion and Li_2_S nucleation/decomposition processes. (d) Evaluation of academic performance and practical applicability based on factors such as structural/chemical stability, cost controllability, scalability, and device compatibility. It should be noted that the comparisons presented in this figure are semi‐quantitative and are derived from representative literature reports and general trends in the field, rather than standardized experimental datasets. Therefore, these plots are intended to provide a qualitative comparison of relative advantages and limitations among different material systems, rather than direct quantitative ranking or statistically rigorous evaluation.

### 1.2 Toward a Unified Evaluation of Inorganic Separator Systems Under Practical Constraints

The development of inorganic separator engineering in Li–S batteries is fundamentally characterized by a persistent duality between a “cornerstone” and a “compromise” role. On the one hand, the intrinsic physicochemical properties of inorganic materials, including tunable electronic structures, multivalent redox centers, and strong interfacial polarity, provide a solid foundation for regulating polysulfide adsorption, catalytic conversion, and interfacial charge transfer, thereby positioning these systems as a potential cornerstone for next‐generation Li–S battery architectures. On the other hand, the same systems inevitably introduce non‐negligible trade‐offs at the device level, such as increased inactive mass, additional interfacial resistance, limited ionic transport efficiency, and scalability constraints, which collectively restrict their practical implementation and thus define their compromise nature under realistic operating conditions. This inherent contradiction between mechanistic superiority and system‐level limitations highlights a critical gap in the field: existing evaluation approaches predominantly focus on isolated electrochemical metrics or material‐level performance descriptors, which are insufficient to resolve whether a given separator design truly approaches practical applicability or remains confined to laboratory‐scale optimization. In particular, the lack of a unified, constraint‐aware, and application‐oriented evaluation standard makes it difficult to directly compare heterogeneous systems reported under vastly different testing conditions.

To address this challenge, a Practical Relevance Index (PRI) is proposed as a unified framework to quantitatively bridge this cornerstone‐compromise duality. The PRI is constructed by integrating key application‐defining parameters, including energy contribution, electrolyte utilization efficiency, structural penalty, and device‐level validation. These metrics are formulated into a set of normalized, dimensionless expressions to ensure comparability across disparate literature reports. The detailed formulation of each component is given as follows

The Energy Contribution Score (ECS) is defined to quantify the effective electrochemical contribution of a separator‐enabled system by jointly considering areal capacity and sulfur loading under practical operating constraints. It is expressed as follows:

(1) $$ECS=\left(\frac{Q_{A}}{Q_{0}}\right)+{\left(\frac{s_{L}}{s_{0}}\right)}^{\frac{1}{2}}$$

where *Q_A_* represents the areal capacity in mAh cm^−2^ and *S_L_* denotes the sulfur loading in mg cm^−2^. The reference values *Q*_0_ = 5 mAh cm^−2^ and *S*_0_ = 5 mg cm^−2^ are introduced to represent practical operating benchmarks for Li–S batteries. The square‐root term is applied to account for the nonlinear decrease in sulfur utilization at elevated loading levels.

The Lean Electrolyte Compatibility Score (LECS) evaluates the ability of a system to operate under electrolyte‐lean conditions, which is essential for achieving high energy density. It is defined as

(2) $$LECS=\frac{{\left(E/S\right)}_{0}}{E/S}$$

where *E*/*S*is the electrolyte‐to‐sulfur ratio expressed in μ L mg^−1^, and (*E*/*S*)_0_ = 5 μ L mg^−1^is selected as a representative threshold for practical operation. This formulation directly reflects electrolyte utilization efficiency, with lower electrolyte usage corresponding to higher LECS values.

The Structural Penalty Score (SPS) is introduced to quantify the additional inactive mass and geometric constraints induced by separator modification. It is expressed as

(3) $$SPS={\left(\frac{m_{C}}{m_{0}}\right)}^{\frac{1}{2}}+{\left(\frac{t}{t_{0}}\right)}^{\frac{1}{2}}$$

where *m_c_* denotes the coating mass in mg cm^−2^ and *t*represents the separator thickness in μ m. The reference values *m*_0_ = 1 mg cm^−2^ and *t*_0_ = 10 μ *m* are adopted to represent minimal structural burden in practical separator configurations. Square‐root normalization is used to avoid over‐penalization of nonlinear structural contributions.

To reflect the level of practical implementation, the Device Validation Score (DVS) is defined as a discrete function.

(4) $$DVS=\left\{\begin{array}{c} 1.0,\;pouch-cell\;vavalidation\;demonstrated\;\;\;\\ \;0.5,\;high-loading\;coin-cell\;configuration\;\;\;\\ \;\;\;\;\;\;\;\;0.2,\;standard\;coin-cell\;testing\;\;\;\;\;\;\;\;\;\;\;\;\;\;\;\;\;\;\;\;\;\;\;\;\;\;\;\;\;\\ 0,no\;device-level\;validation\;reported\;\;\;\;\;\;\;\;\;\;\; \end{array}\right.$$

This metric differentiates between laboratory‐scale electrochemical demonstrations and system‐level device validation.

The Practical Relevance Index (PRI) is constructed as a weighted combination of the four sub‐metrics and is expressed as follows:

(5) PRI=ECS+LECS−SPS+DVS

Equal weighting is adopted for all terms to ensure transparency and avoid subjective parameter tuning. The resulting index is dimensionless and is intended for comparative evaluation rather than absolute physical prediction.

In this revised framework, the previously introduced FoM_Li–S_ is not used as an independent ranking criterion, but is redefined as a localized descriptor of intrinsic electrochemical contribution. The PRI further integrates this intrinsic contribution with practical system‐level constraints to avoid overestimating separator systems evaluated only under favorable laboratory conditions. Equal weighting is adopted not because all terms are assumed to possess identical physical significance, but to avoid introducing additional subjective coefficients into highly heterogeneous literature datasets. Therefore, PRI is intended to identify general constraint‐aware trends rather than provide absolute material rankings. Parameters that are consistently reported across the literature, including sulfur loading, E/S ratio, areal capacity, coating mass, separator thickness, and cell configuration, are incorporated into the PRI calculation, whereas less consistently reported descriptors such as porosity, tortuosity, electrolyte uptake, voltage window, current density, and N/P ratio are documented in the comparative tables when available but are not imposed as mandatory terms to avoid artificial assumptions. Importantly, PRI is formulated as a constraint‐based benchmarking framework rather than a physics‐derived model. Its primary purpose is to enable consistent and transparent evaluation of inorganic separator systems under unified practical constraints, particularly when comparing literature data obtained under heterogeneous experimental conditions.

## 2 Metal Oxide‐Based Separator Coating Materials

Metal oxides are among the most widely studied metal compounds for separator modification in Li–S batteries due to their strong polarity and abundant metal active sites [67, 68]. These characteristics enable strong chemical interactions with lithium LiPSs, effectively suppressing the shuttle effect while also providing catalytic sites for polysulfide conversion [69, 70]. Over the past decade, various transition‐metal oxides (e.g., TiO_2_ [71, 72, 73]_,_ MnO_2_ [74, 75], Co_3_O_4_ [76, 77], and ZnO [78, 79]) have been explored to regulate polysulfide adsorption and reaction kinetics. Representative metal oxide‐based separator systems and their electrochemical performances are summarized in Table S1.

Overall, these representative systems reveal consistent design principles, including strong polar surface‐driven polysulfide adsorption, conductive carbon coupling for improved electron/ion transport, and progressive evolution toward catalytic and defect‐regulated interfaces for enhanced sulfur redox kinetics. Despite differences in composition and structure, most metal oxide‐based coatings converge toward multifunctional integration rather than single adsorption‐dominated behavior.

### 2.1 Titanium Oxide

#### 2.1.1 Mechanistic Evolution of Titanium Oxide‐Based Inorganic Separator Systems for Polysulfide Regulation

In recent years, TiO_2_ has emerged as an effective material for regulating polysulfide chemistry in Li–S batteries owing to its polar surface, structural stability, and tunable interfacial properties [80, 81]. The design strategy of TiO_2_‐based systems has evolved from early adsorption‐driven confinement toward multifunctional architectures integrating catalytic regulation, defect engineering, structural design, and electronic‐structure modulation, reflecting a gradual transition from passive polysulfide trapping to active control of sulfur redox kinetics [82].

Early studies primarily relied on the strong polarity of TiO_2_ surfaces to immobilize lithium LiPSs through adsorption [83]. By coupling TiO_2_ with conductive carbon frameworks such as graphene and graphene oxide (GO), hybrid structures combining physical confinement and chemical anchoring were constructed to suppress the shuttle effect. Representative examples include LTO/graphene coatings [84], TiO_2_/GO 3D networks [85], KTO‐2/KB bifunctional layers [86], and RGO/TiO_2_(B) composites [87]. These systems effectively restricted polysulfide migration while improving electronic conductivity, establishing the early adsorption‐dominated strategy for polysulfide confinement. With increasing understanding of Li–S reaction mechanisms, the role of TiO_2_ has progressively expanded from adsorption to catalytic regulation of sulfur redox reactions. In particular, heterostructure engineering has emerged as a key strategy to couple strong adsorption with catalytic conversion [88]. For instance, the TiO_2_‐TiN “twin” heterostructure constructed an efficient capture‐diffusion‐conversion loop, enabling continuous regulation of polysulfide intermediates during cycling [89]. Subsequent studies further emphasized the importance of electronic‐state modulation at catalytic interfaces. For example, loading TiO_2_ nanoparticles onto glauconite surfaces enabled precise tuning of the p‐band center of oxygen active sites, thereby enhancing catalytic activity toward polysulfide conversion [90]. More recently, Chen et al. [91] reported a Bi_2_Te_3_/TiO_2_ topological‐insulator heterostructure in which the built‐in electric field and engineered band alignment generate interfacial potential wells and dipole domains. These features guide directional transport and confined conversion of LiPSs, simultaneously strengthening adsorption, mass transport, and catalytic kinetics. As a result, the battery delivered a high discharge capacity of 1375 mAh g^−1^ with significantly reduced polarization (Figure 6a,b). This progress highlights a transition from constructing physical confinement structures to designing electronically regulated catalytic interfaces for polysulfide conversion [92]. Beyond catalytic regulation, defect engineering has become an important strategy to further enhance TiO_2_ functionality [93]. Introducing oxygen vacancies generates Ti^3+^ active sites that strengthen polysulfide adsorption while improving electronic conductivity. Li et al. [94] first report was made on a separator coated on the surface with TiO_2_ nanosheets rich in oxygen vacancies (OVs‐TiO_2_), and the formation and function of high‐density oxygen vacancies were discussed and revealed. Building on this concept, Li et al. [95] further designed a TiO_2_ homojunction (A/R‐TiO_2_) featuring band alignment and abundant oxygen vacancies. (Figure 6c) The resulting interfacial active sites efficiently catalyze polysulfide conversion, enabling outstanding capacity retention after 800 cycles at 1 C (Figure 6d,e). In addition, several defect‐regulated TiO_2_ architectures have been reported to further enhance multifunctional performance. These include oxygen‐vacancy‐containing hollow TiO_2_ nanofibers capable of wide‐temperature operation (TCNFs) [96], TiO_2_‐_x_@TiH_2_ core‐shell structures generated through mild oxidation of TiH_2_ [97], and reversible adsorption systems based on in situ formed Li_x_TiO_2_ [98]. Collectively, these studies demonstrate that precise defect regulation can simultaneously improve adsorption capability, electronic conductivity, and catalytic activity. Apart from chemical and electronic regulation, structural engineering plays an essential role in optimizing TiO_2_ performance. Rational nanostructuring of TiO_2_ can expose abundant active sites while constructing rapid ion‐transport pathways and mechanical buffering frameworks. Representative examples include TiO_2_ NT/CNT 3D capture networks [99] and ultrafine TiO_2_‐embedded self‐supporting fibrous layers [100]. These architectures effectively improve active‐site accessibility, reduce ion diffusion resistance, and enhance electrode structural stability.

**FIGURE 6:**
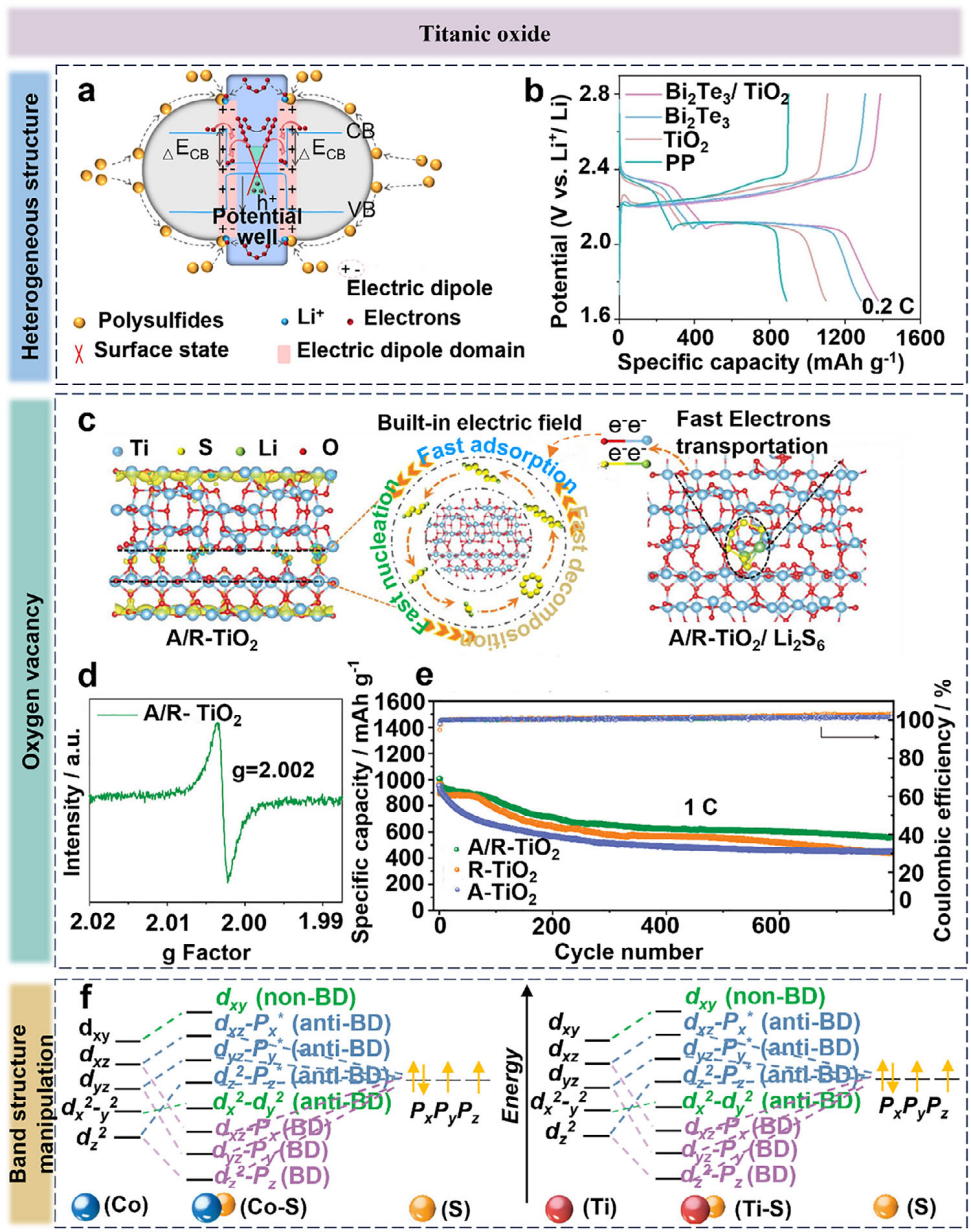
Titanic oxide modified separator strategy. (a) Schematic diagram of the synergistic catalytic mechanism of Bi_2_Te_3_/TiO_2_ heterojunction. The interface electric dipole captures Li^+^ and LiPSs, enabling directional electron migration and efficient interface catalysis. (b) The charge and discharge performance of Bi_2_Te_3_/TiO_2_‑PP separator at 0.2 C. Reproduced with permission [91]. Copyright 2025, Wiley‐VCH. (c) A schematic diagram of the A/R‐TiO_2_ adsorption‐catalysis mechanism. It has been demonstrated that through the synergistic regulation of band alignment and oxygen vacancy engineering, the homogeneous junction interface can effectively suppress the shuttle effect. (d) The electron paramagnetic resonance (EPR) spectrum of A/R‐TiO_2_ (*g* = 2.002). (e) The long‐cycle performance of Li–S batteries based on A/R‐TiO_2_ modified separators at 1 C. Reproduced with permission [95]. Copyright 2023, Wiley‐VCH. (f) Comparison of the orbital hybridization mechanism between high‐spin cobalt and unsaturated titanium. (Left) High‐spin cobalt (HSCO) has abundant low‐energy empty orbitals, which undergo strong covalent orbital hybridization with the p electrons of sulfur (Co─S σ/π bonds). (Right) The 3d orbital arrangement of unsaturated titanium is regular, and there are few low‐energy empty orbitals. Reproduced with permission [101]. Copyright 2025, Wiley‐VCH.

Current research is further advancing toward electronic‐structure modulation and dual electron‐ion conductive strategies to simultaneously achieve high sulfur loading, fast reaction kinetics, and long cycle life [102]. For example, Wang et al. [101] anchored high‐spin cobalt atoms onto porous, undercoordinated TiO_2x_ nanosheets. The symmetry‐broken d‐orbitals of Co strongly hybridize with sulfur lone‐pair electrons in LiPSs, generating strong Co‐S orbital coupling that enables molecular‐level anchoring and accelerated charge transfer (Figure 6f). Similarly, Qian et al. [103] constructed crystalline‐amorphous TiP_2_O_7_/CNT heterointerfaces to tune the d‐p band center, thereby enhancing polysulfide adsorption and catalytic conversion. In addition, nanocrystalline TiO_2_ (P90) was found to enter an accumulation state under specific potentials, enabling band‐structure‐modulated catalysis of sulfur reduction reactions [104]. Meanwhile, the function of TiO_2_‐based materials has gradually expanded from cathode‐side regulation to full‐cell interfacial management. For example, Zhong et al. [105] reported a MOF‐derived a‐TiO_2_‐BDC coating that not only catalyzes polysulfide conversion but also promotes the formation of a flexible and highly ion‐conductive SEI layer, thereby effectively suppressing lithium dendrite growth.

Overall, TiO_2_‐based materials in Li–S battery separators have evolved from simple adsorption hosts toward multifunctional systems integrating heterostructure catalysis, defect engineering, structural design, and band‐structure regulation. Despite the diversity of reported architectures, a unified design trend can be identified: early‐stage systems primarily rely on physical–chemical adsorption, whereas recent strategies increasingly focus on coupling catalytic conversion with interfacial charge redistribution to accelerate polysulfide redox kinetics. This evolution highlights a clear transition from passive confinement to active and electronically regulated sulfur chemistry, providing a general framework for high‐efficiency Li–S separator design.

#### 2.1.2 PRI‐Guided Evaluation of Titanium Oxide‐Based Inorganic Separator Systems: From Mechanistic Advantage to Practical Limitation

To systematically evaluate TiO_2_‐based inorganic separator systems under unified practical constraints, representative architectures are summarized in Table 1. The table compiles key electrochemical parameters, including sulfur loading, electrolyte‐to‐sulfur ratio, areal capacity, coating mass, separator thickness, and device‐level validation. To ensure cross‐study comparability, all systems are further assessed using the PRI, which integrates energy contribution, lean electrolyte compatibility, structural penalty, and device validation into a single constraint‐based framework (Table S2). This enables a consistent evaluation of whether reported performance enhancements originate from intrinsic mechanistic advantages or are limited by practical operating constraints.

**TABLE 1:** Representative titanium oxide‐based inorganic separator systems for Li–S batteries, summarizing key electrochemical parameters and practical operating conditions, including sulfur loading, electrolyte‐to‐sulfur ratio, areal capacity, coating mass, separator thickness, and device‐level validation. This table highlights representative architectures that illustrate the evolution from adsorption‐dominated designs to defect‐engineered and heterostructure‐based systems.

System	Mechanism	Sulfur loading (mg cm^−2^)	E/S (μ L mg^−1^)	Areal capacity (mAh cm^−2^)	Testing condition (rate, cycles)	Coating mass (mg cm^−2^)	Thickness (μ m)	Pouch cell	Refs.
TiO_2_‐TiN/GO/PP	Adsorption‐catalysis synergistic	4.3	10.29	3.03	0.2C, 100 cycles	0.23	9	/	[89]
Bi_2_Te_3_/TiO_2_	Band structure/built‐in electric field	13.2	3.8	11.19	0.05C, first cycle	/	/	(11.2 mAh cm^−2^, 0.05C, first cycle)	[91]
Anatase/rutile TiO_2_ heterojunction	Band structure/built‐in electric field	3.25	/	1.77	0.5C, 300 cycles	/	7	(S: 4.0 mg cm^−2^, 128.5 mAh,0.05C, first cycle)	[95]
OVs‐TiO_2_	Heterojunction/defect‐engineered	2.5	25	3.60	0.3C, first cycle	1.3	25	/	[98]
Co/TiO_2‐x_	Adsorption‐catalysis synergistic	10.9	/	5.96	0.1C, 100 cycles	0.35	13.1	(0.47 Ah,379.3 Wh kg^−1^, first cycle)	[101]
TiP_2_O_7_/CNTs	Adsorption‐catalysis synergistic	5.0	8	3.00	0.1C, 100 cycles	0.6	10	/	[103]

The PRI‐based evaluation reveals clear differences in practical relevance among TiO_2_‐based separator systems, closely associated with their engineering strategies. Heterojunction and multicomponent interface engineering, represented by Bi_2_Te_3_/TiO_2_ and TiO_2_‐TiN/GO/PP, can enhance energy contribution and electrolyte utilization, but their apparent advantages may be affected by incomplete structural reporting. Phase‐junction engineering, as in anatase/rutile TiO_2_, and defect engineering, represented by oxygen‐vacancy‐rich TiO_2_, improve polysulfide conversion kinetics but remain constrained by limited electrolyte efficiency or substantial structural penalties. Catalytic coupling strategies such as Co/TiO_2‐x_ and TiP_2_O_7_/CNTs show moderate practical relevance by balancing redox enhancement with structural burden. The contribution of individual evaluation components, including energy contribution, lean‐electrolyte compatibility, structural penalty, and device validation, to the overall PRI values is further deconvoluted in Table S2, highlighting the intrinsic trade‐off between functional enhancement and practical constraints. Overall, heterostructure, phase‐junction, defect, and conductive‐network engineering do not lead uniformly to practical superiority. Systems with lightweight architectures and favorable electrolyte utilization show greater potential, whereas defect‐rich or heavily modified separators may become compromise‐dominated when coating mass and thickness offset their electrochemical benefits. Standardized reporting of E/S ratio, coating mass, thickness, and cell configuration is therefore essential for reliable PRI interpretation.

### 2.2 Manganese Oxide

#### 2.2.1 Mechanistic Evolution of Manganese Oxide‐Based Inorganic Separator Systems for Polysulfide Regulation

Metal manganese compounds have attracted increasing attention as separator modification materials for Li–S batteries owing to their unique multivalent characteristics, highly polar surfaces, and tunable nanostructures [106]. These features endow manganese‐based materials with significant advantages for regulating polysulfide chemistry. The research and application strategies of manganese compounds have followed a clear evolutionary trajectory, progressing from fundamental adsorption mechanisms to catalytic regulation, and further toward atomic‐level design and scalable engineering approaches [107].

Early studies mainly exploited the strong polarity of manganese oxides, particularly MnO_2_, to immobilize lithium LiPSs through S─Mn─O chemical bonding and electrostatic interactions, thereby constructing the first line of defense against the shuttle effect. Song et al. [108] pioneered the in situ oxidation of polyethylene (PE) separators using KMnO_4_ solution, forming an ultrathin, dense MnO_2_ functional layer with strong adhesion. Subsequently, Wang et al. [109] employed rapid chemical oxidation of polypropylene (PP) separators using acidic KMnO_4_ solution, producing a self‐assembled MnO_2_ layer with an extremely low loading (0.02 mg cm^−2^). Through synergistic adsorption and catalytic effects, this layer effectively improved sulfur utilization (Figure 7a,b). In addition, MnO_2_ nanofiber interlayers [110] and α‐MnO_2_ nanowire “size‐sieving” tunnel structures [111] further demonstrated the strong adsorption capability of manganese oxides, establishing the fundamental framework of manganese‐based adsorption‐type separator modifiers. With a deeper understanding of Li–S reaction mechanisms, the catalytic conversion capability derived from the tunable valence states (Mn^3+^/Mn^4+^) and coordination environments of manganese oxides has become a major research focus. On one hand, regulating the intrinsic valence states of manganese has proven effective in optimizing catalytic activity. For example, Yu et al. [112] introduced mixed Mn^3+^/Mn^4+^ valence states into δ‐MnO_2_ through thermal annealing, forming continuous Mn^3+^‐O‐Mn^4+^ electron transport pathways that significantly accelerated the deposition and dissolution kinetics of Li_2_S. On the other hand, constructing heterostructures to achieve synergistic catalysis has emerged as an even more efficient strategy. Ma et al. [113] designed α‐MnO_2_/RuO_2_ heterostructured nanowires with coherent interfaces (Figure 7c), where spontaneous electron transfer from RuO_2_ to α‐MnO_2_ increased the Mn valence state and altered the Ru chemical environment (Figure 7d). This charge redistribution effectively accelerated polysulfide catalytic conversion, resulting in significantly enhanced Li_2_S deposition capacity. The separator modified with this structure exhibited excellent cycling stability in symmetric batteries, maintaining a low overpotential of approximately 40 mV after 240 h (Figure 7e), indicating efficient interfacial charge transfer and electrochemical stability. Furthermore, Si et al. [114] developed MnO_x_/NiO_x_ composite systems that enabled functional division between Mn (adsorption) and Ni (catalysis), revealing new polysulfide conversion pathways.

**FIGURE 7:**
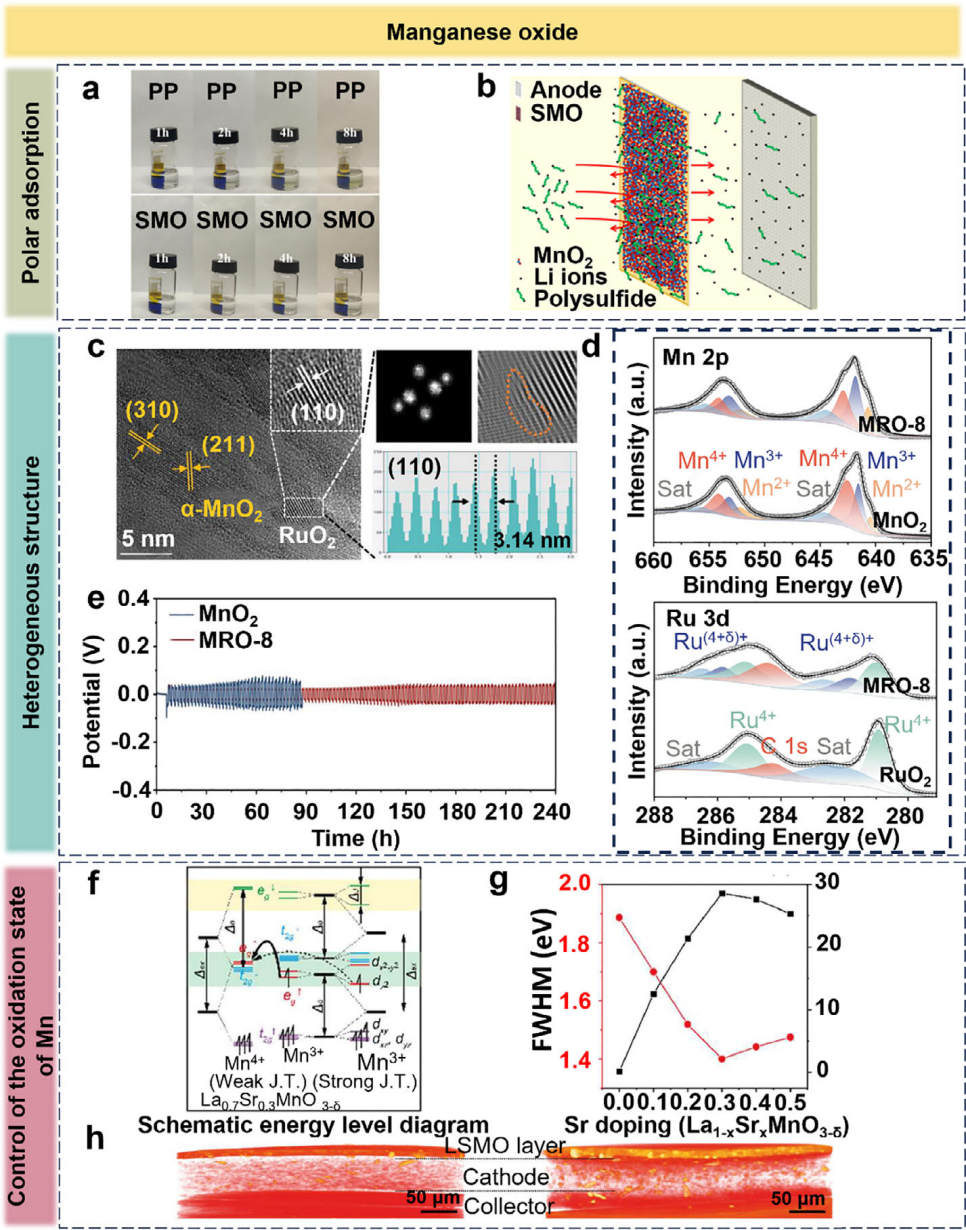
Manganese oxide modified separator strategy. (a) Comparison of polysulfide penetration experiments. The one above is the PP separator, and the one below is the SMO separator. (b) Schematic diagram illustrating the mechanism of SMO membrane‐mediated inhibition of the “shuttling effect”. Reproduced with permission [109]. Copyright 2022, Elsevier. (c) High‐resolution TEM images of the α‐MnO_2_/RuO_2_ heterostructure show the coherent interface formed between the two materials. (d)XPS spectra of the α‐MnO_2_/RuO_2_ heterostructure: (above) Mn 2p region, showing an increase in the Mn oxidation state; (below) Ru 3d region, reflecting the shift in binding energy caused by the transfer of electrons from Ru to Mn. (e) Cycling performance of the Li||Li symmetric battery modified with the α‐MnO_2_/RuO_2_ heterostructure. Atomic‐level regulation and performance optimization. Reproduced with permission [113]. Copyright 2024, Wiley‐VCH. (f) Schematic diagram of electron orbital regulation: Sr^2+^ doping weakens the Jahn‐Teller distortion and promotes electron transitions between Mn^3+^/Mn^4+^. (g) Conductivity optimization curve: As the Sr doping amount changes, the material's conductivity reaches a peak of 28.61 S cm^−1^ at x = 0.3 h) Visualization verification of the diaphragm function (left): Before the cycle, the battery CT image shows that sulfur is evenly distributed in the positive electrode. (right) After the cycle, the battery CT image shows that the sulfur species are effectively enriched and blocked at the diaphragm interface. Reproduced with permission [115]. Copyright 2022, Elsevier.

Subsequent studies expanded from single metal oxides to bimetallic oxides and multicomponent composite systems, aiming to integrate adsorption, conductivity, and catalytic functionalities. Wang et al. [116] combined MnFe_2_O_4_ with acetylene black to construct a bifunctional coating that simultaneously provides chemical adsorption and conductive catalytic activity. Similarly, Zhou et al. [117] fabricated composites of manganese‐iron layered double hydroxide (Mn‐Fe LDH) with carbon nanotubes, achieving enhanced chemical adsorption and catalytic conversion simultaneously. In terms of structure, function coupling, integrating manganese compounds with carbon nanotube frameworks can impart excellent mechanical flexibility to the battery system [118]. Nickel and manganese oxide co‐modified carbon nanotubes (Ni‐MnO@CNTs) further demonstrated outstanding cycling stability [119]. In addition, hollow, layered, and 3D porous structures‐including MnFe_2_O_4_/MnO heterostructures [120] and CoMn_2_O_4_/PPy hollow spheres [121] provide additional physical confinement and interfacial stability. These structural innovations collectively promote the evolution of separator modification from simple single‐layer coatings toward multiscale interfacial engineering. Current research frontiers are increasingly focused on precise atomic‐ and electronic‐level regulation as well as the integration of multiple active sites. Hao et al. [115] achieved precise regulation of Mn valence states and orbital environments by doping Sr^2+^ into perovskite lattices (Figure 7f), which significantly improved electrical conductivity (Figure 7g). Moreover, advanced characterization techniques enabled direct visualization of polysulfide distribution at the separator interface (Figure 7h). Meanwhile, high‐entropy design concepts have been introduced to construct systems with exceptional structural stability and multi‐metal synergistic activity. Representative examples include quinary high‐entropy oxides (HEO) [122]. and CoNiCuMnMo high‐entropy alloys (HEA) [123], which maintain excellent electrochemical performance even under high sulfur loading conditions. In addition, the single‐atom MnN_2_O_2_ catalyst reported by Zhang et al. [124] represents a frontier strategy for maximizing active‐site utilization at the atomic scale. From a practical perspective, controllable and scalable fabrication techniques are essential for real‐world applications. Hirt et al. [125] demonstrated magnetron sputtering as an effective method for depositing ultrathin and uniform amorphous MnO_2_ films on separators. The film thickness can be precisely controlled while minimizing substrate damage, highlighting strong engineering potential. Combined with other approaches such as chemical oxidation, hydrothermal growth, and vacuum filtration, manganese‐based separator modification has gradually established a relatively complete technological pathway from laboratory research to industrial implementation.

MnO_2_‐based separators are distinguished by their intrinsic multivalent Mn^3+^/Mn^4+^ redox couple, which enables dynamic mediation of polysulfide conversion rather than simple surface adsorption. The reversible valence transitions facilitate continuous electron transfer pathways, accelerating LiPS nucleation and decomposition while stabilizing intermediate species. Compared with purely adsorption‐driven oxides, MnO_2_ functions more as a redox mediator that actively participates in sulfur chemistry. Structural and compositional engineering further enhances interfacial accessibility and reaction efficiency, enabling sustained electrochemical kinetics under high loading conditions.

#### 2.2.2 PRI‐Guided Evaluation of Manganese Oxide‐Based Inorganic Separator Systems: From Mechanistic Advantage to Practical Limitation

To provide a more quantitative comparison of the manganese oxide‐based separator systems discussed above, the representative architectures and their corresponding electrochemical performance metrics are summarized in Table 2. The table integrates key experimental parameters, including sulfur loading, electrolyte‐to‐sulfur ratio, areal capacity, structural characteristics, and device‐level validation, enabling a unified assessment under the PRI framework (Table S3). This enables a direct correlation between mechanistic features and practical performance constraints, facilitating a systematic evaluation of whether each system approaches cornerstone‐like behavior or remains limited by compromise‐dominated characteristics under realistic operating conditions.

**TABLE 2:** Representative manganese oxide‐based inorganic separator systems for Li–S batteries, summarizing key electrochemical parameters and practical operating conditions, including sulfur loading, electrolyte‐to‐sulfur ratio, areal capacity, coating mass, separator thickness, and device‐level validation. This table highlights representative architectures that illustrate the evolution from adsorption‐dominated designs to defect‐engineered and heterostructure‐based systems.

System	Mechanism	Sulfur loading (mg cm^−2^)	E/S (μ L mg^−1^)	Areal capacity (mAh cm^−2^)	Testing condition (rate, cycles)	Coating mass (mg cm^−2^)	Thickness (μ m)	Pouch cell	Refs.
MnO_2_@PE	Adsorption‐dominated	2.5	7.0	1.5	0.5C, 500 cycles	0.014	0.38	/	[108]
MnO_2_@PP (SMO)	Adsorption‐dominated	1.2	20	0.59	0.5C, 500 cycles	0.07	0.3	/	[109]
Treated MnO_2_	Heterojunction/defect‐engineered	10.91	6.46	4.0	0.6C, 100 cycles	/	/	/	[112]
α‐MnO_2_/RuO_2_ (MRO‐8)	Band structure/built‐in electric field	6.5	8	5.0	0.2C, 18 cycles	0.2	/	(S: 1.88 mg cm^−2^, 953.16 mAh g^−1^, 0.1C, 200, cycles)	[113]
MnOx‐NiOx/C@PP	Adsorption‐catalysis synergistic	1.3	11	0.66	2C, 700 cycles	0.4	12	/	[114]
MnFe_2_O_4_/AB@PP (MFO‐5%)	Adsorption‐catalysis synergistic	1.0	25	0.63	1C, 500 cycles	0.57	14.6	/	[116]
MFLC (Mn‐Fe LDH/CNT)	Adsorption‐catalysis synergistic	1.4	20	0.66	1C, 500 cycles	0.5	9	/	[117]
MWCNTs/S‐MWCNTs/MnO_2_@PP@Li	Adsorption‐catalysis synergistic	2.0	30	1.4	1C, 500 cycles	0.2‐0.48 (MnO_2_) + 2.0 (S)	25+6+19	/	[118]
Ni‐MnO@CNTs/PP	Adsorption‐catalysis synergistic	3.5	10	3.43	1C, 500 cycles	0.25	12	/	[119]
MS‐HEO	Adsorption‐catalysis synergistic	7	15	5.33	1C, 450 cycles	0.23	5	/	[122]
MnSA@HNC	Band structure/built‐in electric field	6.58	15	3.15	1C, 1400 cycles	0.2	21	(33.2 mg, 701 mAh g^−1^, 0.2C, 40 cycles)	[12]

The PRI‐guided evaluation reveals a highly dispersed and mechanism‐dependent performance landscape for MnO_2_‐based separators, where strong intrinsic electrochemical activity does not necessarily translate into practical applicability. Treated MnO_2_, α‐MnO_2_/RuO_2_, MS‐HEO, and MnSA@HNC exhibit relatively high energy contributions, reflecting the effectiveness of defect engineering, built‐in electric fields, high‐entropy structures, and atomic‐scale catalytic sites in promoting sulfur redox reactions. However, many MnO_2_‐based systems still require high E/S ratios, indicating limited compatibility with lean‐electrolyte conditions. Structural burden is another decisive constraint. Thick coatings, multilayer conductive networks, and multicomponent architectures increase inactive mass and separator thickness, partially or completely offsetting their electrochemical advantages. Consequently, MnO_x_‐NiO_x_/C@PP, MnFe_2_O_4_/AB@PP, MFLC, and multilayer MWCNT‐based separators fall into a compromise‐dominated regime with low or negative PRI values. Among systems with relatively complete data, MS‐HEO, MnO_2_@PE, and MnSA@HNC show the most favorable balance between energy contribution and practical constraints. Although treated MnO_2_ and α‐MnO_2_/RuO_2_ display high apparent PRI values, their incomplete structural reporting may underestimate SPS and overstate practical relevance. Therefore, future MnO_2_ separator design should prioritize lean‐electrolyte operation, low coating mass, reduced thickness, and standardized parameter reporting rather than catalytic complexity alone.

Overall, the MnO_2_‐based separator family does not exhibit a uniform trajectory toward either cornerstone or compromise behavior. Instead, its practical relevance is governed by a strong coupling between catalytic activity and structural/electrolyte constraints. The PRI framework therefore highlights that only those systems achieving simultaneous optimization of ECS, LECS, and SPS can be regarded as true cornerstone candidates under realistic Li–S battery operating conditions.

### 2.3 Cobalt Oxide

#### 2.3.1 Mechanistic Evolution of Cobalt Oxide‐Based Inorganic Separator Systems for Polysulfide Regulation

Cobalt oxides, including CoO [126, 127], Co_3_O_4_ [128, 129], and their composites [130, 131], have been widely recognized as one of the most promising inorganic functional materials for separator modification in Li–S batteries. Benefiting from their highly polar surfaces, multiple valence states, tunable electronic structures, and strong compatibility with carbon materials, cobalt oxides offer distinct advantages for regulating polysulfide chemistry. In addition to providing strong chemical anchoring for lithium LiPSs, they can also serve as efficient catalytic centers to accelerate the bidirectional redox conversion of LiPSs, making them ideal candidates for constructing high‐performance functional separators. The development of cobalt oxide‐based separator materials has followed a clear evolution from basic adsorption functionality to catalytic conversion, interfacial electronic regulation, and ultimately atomic‐scale defect engineering [132].

The strong polarity of cobalt oxides enables chemical bonding interactions with LiPSs, forming the basis for physical–chemical adsorption and shuttle suppression. For instance, the Co_9_S_8_/CoO heterostructure cleverly integrates the strong adsorption capability of CoO with the catalytic activity of Co_9_S_8_, achieving an extremely low‐capacity decay rate of 0.049% per cycle even under high sulfur loading [127]. Spinel Co_3_O_4_ regenerated from recycled lithium‐ion battery cathode materials (S‐LCO) also exhibits excellent LiPS capture ability due to its abundant metal active sites, maintaining high capacity at elevated current densities [133]. In addition, bimetallic oxides such as CoMn_2_O_4_ [121] and CoFe_2_O_4_ [134], along with various hollow structural designs, further enhance adsorption and confinement effects by increasing active interfacial areas and facilitating ion diffusion. However, adsorption alone cannot fundamentally eliminate capacity fading. Therefore, utilizing the variable valence states of cobalt oxides to catalyze rapid polysulfide conversion has become a critical development direction [135]. Catalytic separators aim to rapidly convert trapped LiPSs into solid end products, thereby reducing their concentration in the electrolyte and effectively suppressing the shuttle effect [136]. A representative example is the Co@CoO@N‐C/rGO core‐shell structure designed by Li et al. [137]. As illustrated in Figure 8a, the outer CoO and N‐doped carbon shell provide strong chemical adsorption, while the internal metallic Co core functions as a catalytic “scissor” that efficiently promotes the conversion of LiPSs into Li_2_S. As a result, the battery maintained a capacity retention of 83.2% after 100 cycles with a sulfur loading of 1.5 mg cm^−2^. Similarly, systems such as CoFe_2_O_4_@PANI [93] and CoO/NiO@C‐NC [126] accelerate the solid‐liquid‐solid phase transformation through multimetal synergy or stepwise catalytic mechanisms, significantly improving rate capability. Constructing heterostructures to regulate interfacial charge distribution and generate built‐in electric fields provides a deeper level of control over catalytic pathways and reaction energy barriers, and has become a major research focus. Zhang et al. [138] fabricated CoO‐Co_3_O_4_ hollow nanotube heterostructures (HNTs), where the difference in work functions induces interfacial electron transfer and generates a built‐in electric field directed from Co_3_O_4_ to CoO (Figure 8b). This electric field not only enhances electrostatic adsorption but also optimizes the sulfur reduction reaction pathway, minimizing the Gibbs free energy of key reaction steps and significantly improving conversion kinetics (Figure 8c). A similar mechanism has been observed in the CoSe_2_/CoO Mott–Schottky heterostructure [139], where the resulting p–p junction electric field enables selective catalytic conversion. In addition, systems such as CoO‐Co_4_N [140] and TiN/CoO‐CNT [126] further enhance interfacial electronic activity through synergistic interactions between polar and conductive phases. Atomic‐level tailoring of intrinsic electronic structures represents the frontier of cobalt oxide‐based separator research, mainly involving defect engineering and spin‐state regulation. Bai et al. [141] created La vacancies in LaCoO_3_, exposing Co‐rich active surfaces and inducing mixed Co^3+^/Co^4+^ valence states. This modification shifts the d‐band center upward, simultaneously enhancing adsorption capability and electronic conductivity, thereby effectively suppressing the shuttle effect (Figure 8d,e). Zhou et al. [142] further regulated the spin state of Co^3+^ in Co_3_O_4_ by V doping, converting it from a low‐spin state to an intermediate‐spin configuration. This optimization of orbital occupation significantly strengthens the bonding interaction with LiPSs (Figure 8f), guiding uniform 3D nucleation of Li_2_S and enabling ultralong cycling stability (Figure 8g,h). In addition, CeO_2_‐CoO heterostructures enriched with oxygen vacancies exhibit enhanced adsorption and catalytic performance due to increased d‐orbital electron density [143]. These studies collectively advance catalytic separator design toward precise atomic‐level engineering.

**FIGURE 8:**
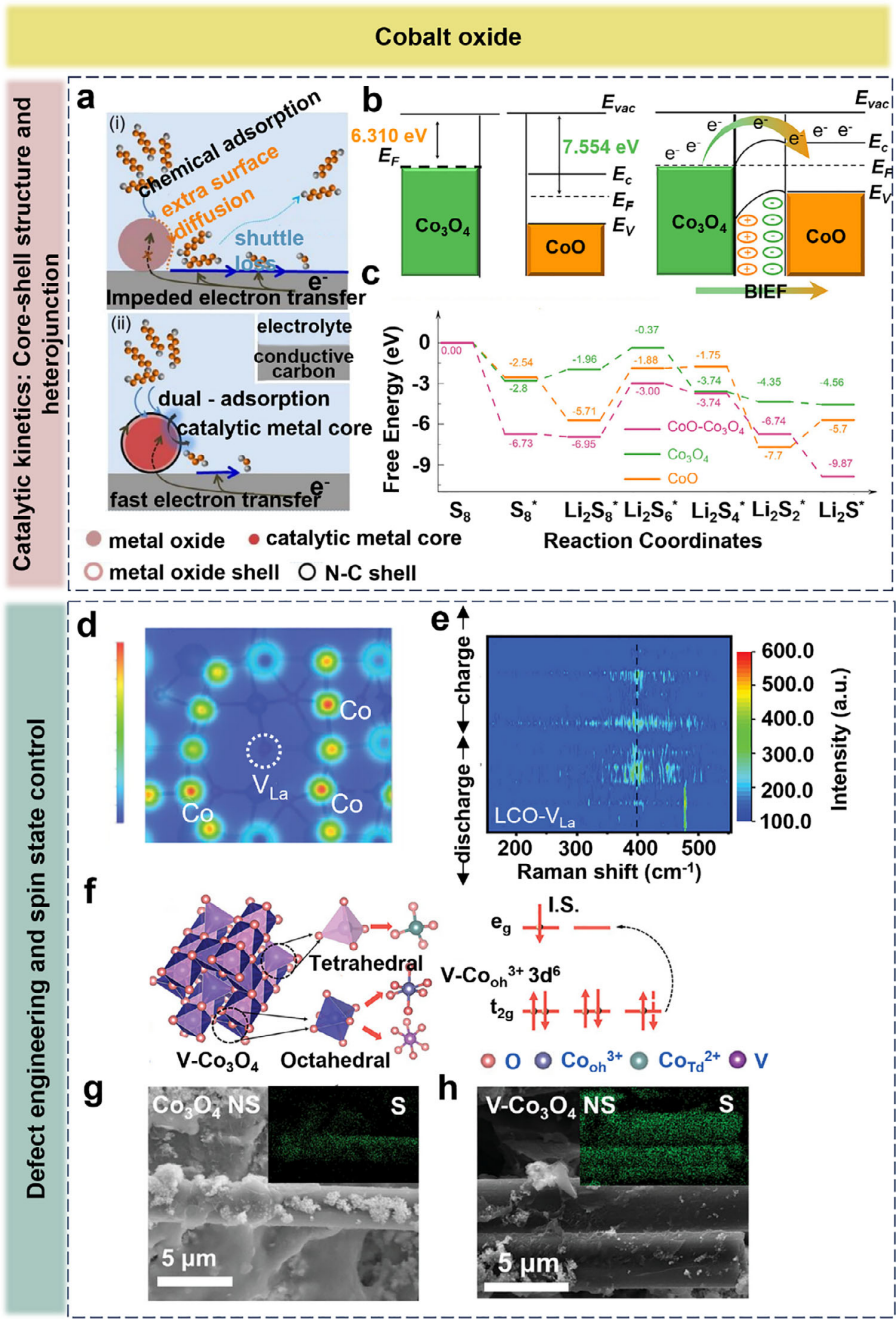
Cobalt oxide‐modified separator strategy. (a) The synergistic mechanism of Co@CoO@N‐C/rGO with LiPSs. DFT Analysis of Built‐In Electric Field at Heterojunction Interface and Catalytic Mechanism. (b) The difference in work functions between CoO and Co_3_O_4_ induces electron transfer, resulting in a built‐in electric field pointing towards CoO at their interface. (c) The CoO‐Co_3_O_4_ heterojunction exhibits the lowest Gibbs free energy in each step of the sulfur reduction reaction, indicating its optimal thermodynamic and catalytic kinetic advantages. Reproduced with permission [138]. Copyright 2025, Wiley‐VCH. Defect engineering: Vacancies in La cause the charge redistribution and electronic structure activation of LaCoO_3_. (d) Theoretical calculation of the charge density difference map. (e) The contour plot of in situ Raman spectra of the battery based on La vacancies LaCoO_3_ (LCO‐V‐La) during the charging and discharging process. Reproduced with permission [141]. Copyright 2025, Wiley‐VCH. Spin state engineering: V doping regulates the electronic configuration and bonding ability of Co_3_O_4_. (f) Schematic diagram of the spin state transformation of spinel Co_3_O_4_ induced by V atom doping. (g) SEM morphology comparison between the original Co_3_O_4_ and the (h) V‐doped Co_3_O_4_ after the Li_2_S deposition experiment. Reproduced with permission [142]. Copyright 2024, Wiley‐VCH.

The development of cobalt oxide‐based separator materials is characterized by multivalent redox chemistry and spin‐dependent electronic modulation, enabling coupled regulation of LiPS adsorption and conversion. Instead of relying on surface polarity, Co‐based systems leverage interfacial charge asymmetry and spin‐state tuning to optimize reaction pathways. Hierarchical structures such as hollow nanocages, 2D nanosheets, and aerogels [126, 144] further enhance interfacial exposure and catalytic cooperation. These features collectively enable efficient polysulfide confinement and fast redox kinetics under practical conditions.

#### 2.3.2 PRI‐Guided Evaluation of Cobalt Oxide‐Based Inorganic Separator Systems: From Mechanistic Advantage to Practical Limitation

To quantitatively evaluate the practical relevance of cobalt oxide‐based inorganic separator systems, representative architectures are summarized in Table 3. The table compiles key electrochemical parameters, including sulfur loading, electrolyte‐to‐sulfur ratio, areal capacity, coating mass, separator thickness, and device‐level validation. Based on the PRI, all systems are assessed under a unified constraint‐based framework, enabling a consistent comparison of energy contribution, electrolyte utilization efficiency, and structural penalty across different cobalt‐based configurations (Table S4). This allows a direct interpretation of whether the reported performance improvements originate from intrinsic mechanistic advantages or are limited by structural and operational constraints.

**TABLE 3:** Representative cobalt oxide‐based inorganic separator systems for Li–S batteries, summarizing key electrochemical parameters and practical operating conditions, including sulfur loading, electrolyte‐to‐sulfur ratio, areal capacity, coating mass, separator thickness, and device‐level validation. This table highlights representative architectures that illustrate the evolution from adsorption‐dominated designs to defect‐engineered and heterostructure‐based systems.

System	Mechanism	Sulfur loading (mg cm^−2^)	E/S (μ L mg^−1^)	Areal capacity (mAh cm^−2^)	Testing condition (rate, cycles)	Coating mass (mg cm^−2^)	Thickness (μ m)	Pouch cell	Refs.
CoO/NiO@C‐NC	Adsorption‐catalysis synergistic	5.87	/	4.00	0.2C, 100 cycles	/	19	/	[126]
Co_9_S_8_/CoO‐G	Adsorption‐catalysis synergistic	2.5	15	2.31	1C, 1000 cycles	0.4	18	/	[127]
Co_3_V_2_O_8_ (CVO)	Adsorption‐catalysis synergistic	1.5	15	0.76	5C, first cycle	0.3	6.0	(796 mAh g^−1^, 1C, 100 cycles)	[136]
Co@CoO@N‐C/rGO	Adsorption‐dominated	1.5	20	1.35	0.2C, 100 cycles	0.4	/	/	[137]
CoO‐Co_3_O_4_ (HNTs)	Band structure/built‐in electric field	5.0	10.17	3.16	0.5C, 60 cycles	/	/	/	[138]
LaCoO_3_ (LCO‐VLa)	Heterojunction/defect‐engineered	4.5	/	4.00	0.1C, 70 cycles	0.14	10	/	[141]
Co_3_O_4_ (V‐Co_3_O_4_ NS)	Band structure/built‐in electric field	15.3	8	13.68	0.1C, 100 cycles	/	/	/	[142]
CeO_2_‐CoO/rGO	Heterojunction/defect‐engineered	6.7	4.7	4.01	0.2C, 50 cycles	/	/	(895 mAh g^−1^, 0.2C,30 cycles)	[143]
TiN/CoO@N‐CNT (TCCNT)	Adsorption‐catalysis synergistic	1.0	10	0.73	1C, 500 cycles	/	/	/	[145]

The PRI‐based evaluation reveals a highly heterogeneous practical‐relevance landscape among Co‐based separator systems, closely linked to their engineering strategies. Band‐structure and built‐in‐electric‐field engineering, represented by V‐Co_3_O_4_ nanosheets and CoO‐Co_3_O_4_ heterostructures, deliver strong energy contributions and comparatively high apparent PRI values. Heterojunction and defect engineering, as in CeO_2_‐CoO/rGO and LaCoO_3_‐based systems, also improves sulfur‐redox kinetics, but incomplete structural reporting or limited electrolyte optimization prevents a definitive assessment of their practical superiority. Adsorption‐catalysis synergistic architectures, including CoO/NiO@C‐NC, Co_9_S_8_/CoO‐G, Co_3_V_2_O_8_, and TiN/CoO@N‐CNT, show widely varying outcomes. Systems with relatively low structural burden achieve moderate positive PRI values, whereas Co_9_S_8_/CoO‐G is compromise‐dominated because its coating mass and separator thickness offset its electrochemical benefits. Adsorption‐dominated designs such as Co@CoO@N‐C/rGO occupy a transitional regime, providing moderate practical relevance but limited energy contribution. Overall, no single engineering route guarantees practical applicability. Band‐structure regulation and defect/heterojunction engineering appear most promising, while multicomponent adsorption‐catalysis designs require tighter control of coating mass, thickness, and electrolyte consumption. Standardized reporting of these parameters is essential for distinguishing genuine cornerstone candidates from systems with only high apparent PRI values.

### 2.4 Zinc Oxide

#### 2.4.1 Mechanistic Evolution of Zinc Oxide‐Based Inorganic Separator Systems for Polysulfide Regulation

Among separator modification materials for Li–S batteries, zinc oxide (ZnO), as a typical highly polar metal oxide, has attracted considerable attention due to its strong chemical interaction with lithium LiPSs [146]. The surface Zn^2+^ species can form strong Zn─S bonds with S^2−^ in LiPSs, enabling effective chemical anchoring and making ZnO one of the key materials for suppressing the shuttle effect [147]. Research on ZnO‐based systems has systematically evolved around fundamental adsorption behavior, structural regulation, conductive composites, heterostructure catalysis, and advanced fabrication techniques, forming a clear development pathway.

The primary advantage of ZnO lies in its strong chemical adsorption capability, which has been confirmed by numerous studies [148]. For example, a separator coating composed of ZnO nanoparticles and nitrogen‐doped graphene (ZnO/NDG) utilizes the synergistic interaction between the polar adsorption of ZnO and nitrogen functional groups, achieving a capacity retention of 90.02% after 200 cycles at 0.1 C [149]. Similarly, a 3D hierarchical interlayer constructed from polypyrrole (PPy) and ZnO effectively reduces charge‐transfer resistance through the synergistic combination of hydrogen‐bond adsorption from PPy and chemical anchoring from ZnO [150]. Theoretical calculations further indicate that defect engineering, such as introducing oxygen vacancies, can significantly enhance the adsorption energy and electronic conductivity of ZnO, providing a basis for optimizing the dual functionality of adsorption and catalysis [151]. In addition, the geometric dimensionality of ZnO structures (0D, 1D, and 3D) plays a decisive role in determining their electrochemical performance [152]. Studies have shown that 1D ZnO nanotubes provide the best balance among adsorption capability, ion transport, and structural stability due to their directional ion channels, double‐shell architecture, and internal cavities, outperforming both 0D nanoparticles and 3D hollow octahedra. These findings suggest that multidimensional structural coupling is an important strategy for optimizing ZnO‐based separator coatings [153]. Given the intrinsically low electrical conductivity of ZnO, integrating ZnO with highly conductive carbon materials has become an essential strategy for improving rate capability and reaction kinetics. Early studies demonstrated that co‐coating ZnO nanoparticles with porous carbon enables simultaneous polysulfide adsorption and electronic conduction under limited coating mass [78]. To further enhance this effect, Pi et al. [147] designed a “chain‐bead” structured ZnO@N‐CNTs system, which constructs a conductive network with abundant active sites (Figure 9a). As a result, the battery maintained a reversible capacity of 671.5 mAh g^−1^ at a high rate of 5 C and exhibited excellent capacity recovery (Figure 9b). Using metal‐organic frameworks (MOFs) as precursors represents another effective approach. For instance, porous ZnO/N‐C carbon frameworks derived from pyrolyzed ZIF‐8 [154] and Y_2_O_3_/ZnO@C composite functional layers derived from Y‐Zn MOFs [146] both demonstrate significantly improved separator performance through the construction of porous conductive skeletons. To overcome the limitations of simple adsorption, constructing ZnO‐based heterostructures to introduce built‐in electric fields and achieve multiphase synergistic catalysis has emerged as an advanced strategy for accelerating polysulfide conversion kinetics. Zhang et al. [155] fabricated a heterostructure between ZnO and another transition‐metal compound, where interfacial electron redistribution accelerates charge transfer and polysulfide conversion (Figure 9c). A similar catalytic enhancement was observed in Co_3_O_4_/ZnO heterostructures [156]. In terms of structural design, the SiO_2_‐ZnO triple‐layer tubular structure realizes multifunctional synergy through a “conductive skeleton‐insulating layer‐polar layer” configuration (Figure 9d), integrating physical confinement, chemical adsorption, and ion sieving [157]. More advanced studies focus on band‐structure regulation and defect coupling. For example, Ouyang et al. [79] constructed an oxygen‐vacancy‐rich ZnV_2_O_4_/ZnO heterostructure (CoNi@ZnV_2_O_4_/ZnO‐N, C). In this system, the heterostructure regulates band alignment, oxygen vacancies enhance adsorption and lattice stability, and CoNi alloys together with N‐doped carbon layers provide catalytic active sites. These multiple effects collectively accelerate the deposition and dissolution kinetics of Li_2_S (Figure 9e,f). For practical applications, advanced fabrication techniques and integrated separator architectures are equally important. Atomic layer deposition (ALD) can deposit ultrathin and uniform ZnO films on substrates, effectively improving lithium deposition behavior without significantly increasing interfacial resistance, thereby enhancing Coulombic efficiency and interfacial stability [158]. In terms of multifunctional composite separators, Chang et al. [159] reported an Al_2_O_3_@ZnO‐ANF (aramid nanofiber) composite separator, where high‐strength and heat‐resistant ANF serves as a flexible framework. Owing to its relatively high thermal conductivity, this structure can effectively dissipate internal heat within the battery and improve overall safety.

**FIGURE 9:**
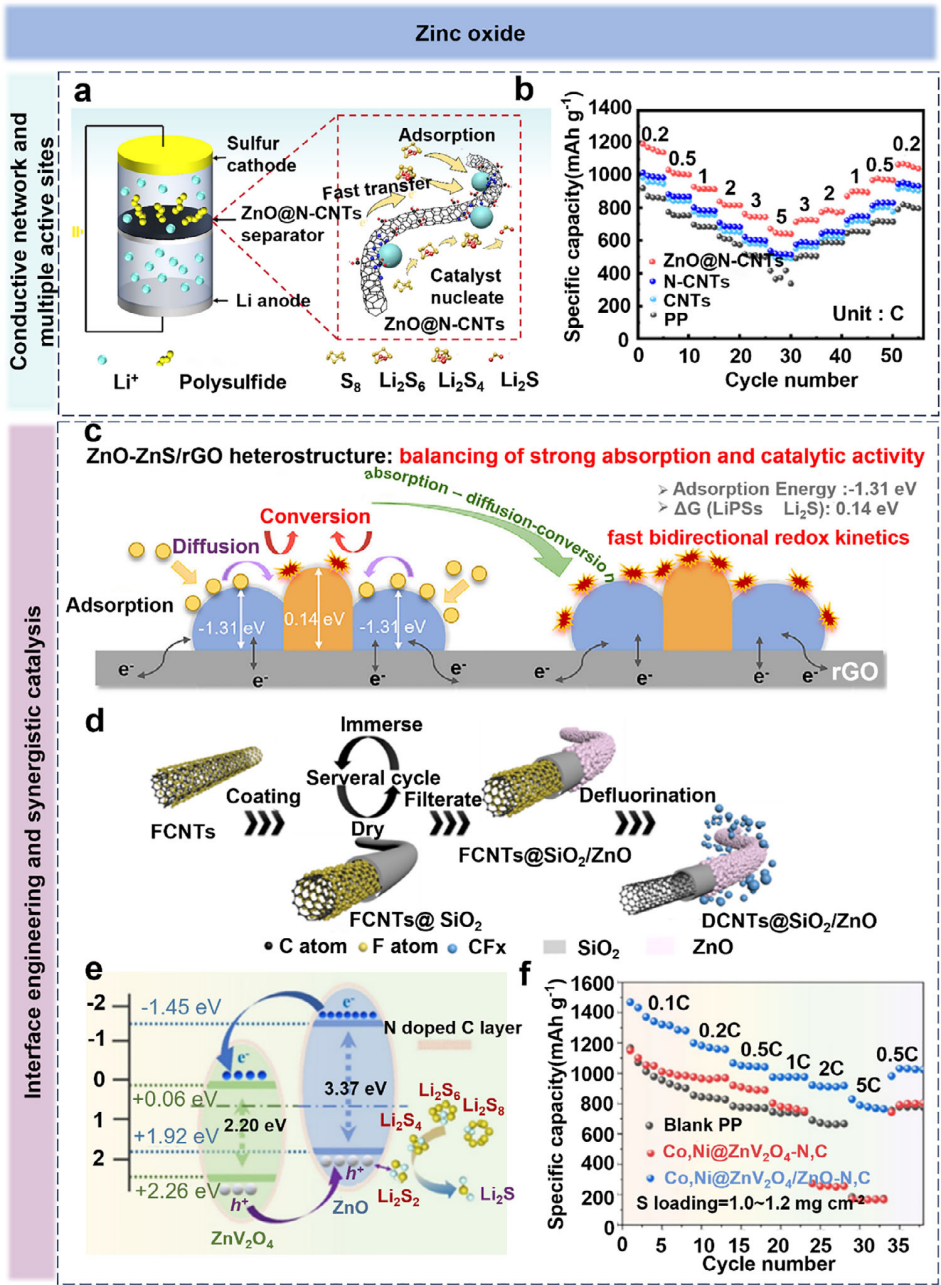
Zinc oxide‐modified separator strategy. (a) Schematic diagram of the mechanism of action of ZnO@N‐CNTs modified separator. (b) Rate performance of the battery using ZnO@N‐CNTs separator. Reproduced with permission [147]. Copyright 2025, Elsevier. (c) Schematic illustration for the working mechanism in Li–S batteries with ZnO‐ZnS/rGO heterostructures functionalized separators. Reproduced with permission [155]. Copyright 2022, Elsevier. (d) Schematic diagram of the preparation process of DCNT@SNT‐ZnO. Reproduced with permission [157]. Copyright 2024, Elsevier. (e) Schematic illustration of the band structure of the oxygen‑vacancy‑rich ZnV_2_O_4_/ZnO heterojunction and its synergistic adsorption‑catalysis mechanism toward lithium LiPSs. (f) Discharge capacities of the Li‑S battery assembled with CoNi@ZnV_2_O_4_/ZnO‑N, C‑modified separator at various C‑rates. Reproduced with permission [79]. Copyright 2024, Elsevier.

ZnO‐based separators are uniquely defined by strong polar Zn‐S interactions combined with intrinsically poor electronic conductivity, making performance highly dependent on interfacial and structural engineering. To overcome this limitation, ZnO is typically integrated with conductive carbon matrices or engineered into heterostructures, where interfacial charge redistribution and band alignment accelerate LiPS conversion while maintaining effective adsorption. Meanwhile, dimensional and architectural control (0D‐3D) further optimizes ion transport pathways and structural stability. These characteristics make ZnO a representative system in which functional performance is primarily achieved through conductivity compensation and multiscale structural integration rather than intrinsic catalytic activity.

#### 2.4.2 PRI‐Guided Evaluation of Zinc Oxide‐Based Inorganic Separator Systems: From Mechanistic Advantage to Practical Limitation

To systematically evaluate ZnO‐based inorganic separator systems under the unified PRI framework, representative architectures are summarized in Table S5. Compared with TiO_2_‐, MnO_2_‐, and Co‐based systems, ZnO‐based separators exhibit a more pronounced dependence on interfacial engineering strategies such as heterostructure construction, surface modification, and conductive network integration, leading to a wider dispersion in performance across different design routes. Based on the calculated PRI components, including energy contribution (ECS), electrolyte utilization efficiency (LECS), structural penalty (SPS), and device‐level validation (DVS), these systems are quantitatively compared to distinguish mechanistic enhancement from practical applicability (Table S5). Accordingly, Table 4 provides a unified benchmark for evaluating whether the reported performance improvements originate from intrinsic material activity or are primarily driven by structural optimization.

**TABLE 4:** Representative zinc oxide‐based inorganic separator systems for Li–S batteries, summarizing key electrochemical parameters and practical operating conditions, including sulfur loading, electrolyte‐to‐sulfur ratio, areal capacity, coating mass, separator thickness, and device‐level validation. This table highlights representative architectures that illustrate the evolution from adsorption‐dominated designs to defect‐engineered and heterostructure‐based systems.

System	Mechanism	Sulfur loading (mg cm^−2^)	E/S (μ L mg^−1^)	Areal capacity (mAh cm^−2^)	Testing condition (rate, cycles)	Coating mass (mg cm^−2^)	Thickness (μ m)	Pouch cell	Refs.
ZnO/CNT/RGO	Adsorption‐catalysis synergistic separator	4.3	10	3.09	0.2C, 150 cycles	0.85	/	/	[78]
Y_2_O_3_/ZnO@C	Adsorption‐catalysis synergistic separator	5	25	4.33	0.1C, 100 cycles	0.1	10	/	[146]
ZnO@N‐CNTs	Adsorption‐catalysis synergistic separator	3.7	13	2.67	0.2C, 150 cycles	/	17	/	[147]
ZnO/RGO	Heterojunction/defect‐engineered separator	3.5	25	2.94	0.2C, 150 cycles	0.4	71.2	/	[149]
ZnO/N/KB	Adsorption‐dominated separator	3	/	1.49	0.5C, 400 cycles	/	10	/	[154]
ZnO‐ZnS/rGO	Heterojunction/defect‐engineered separator	4.2‐7.6	6	4.27	1C, 50 cycles	0.3	4.8	/	[155]
DCNT@SNT‐ZnO	Adsorption‐catalysis synergistic separator	4.8	10	3.77	1C, 200 cycles	/	53.4	/	[156]
CoNi@ZnV_2_O_4_/ZnO‐N, C	Band structure/built‐in electric field modulated separator	4.2	12	3.77	0.1C, 30 cycles	/	/	/	[157]

The PRI‐guided evaluation reveals that the practical relevance of ZnO‐based separators is strongly dependent on the adopted engineering strategy. Heterostructure and phase‐interface engineering, represented by ZnO‐ZnS/rGO and Y_2_O_3_/ZnO@C, provides relatively favorable PRI values by combining enhanced sulfur‐redox activity with manageable structural penalties. Conductive‐network engineering, including ZnO/CNT/RGO and ZnO@N‐CNTs, offers moderate practical relevance, although incomplete thickness or coating‐mass data introduces uncertainty into their apparent PRI values. In contrast, excessively thick carbon‐supported architectures, particularly ZnO/RGO and DCNT@SNT‐ZnO, suffer from substantial structural penalties that offset their electrochemical gains. Multimetal and defect‐coordination engineering, represented by CoNi@ZnV_2_O_4_/ZnO‐N, C, yields a high apparent PRI because of balanced energy contribution and electrolyte utilization; however, the absence of coating‐mass and thickness data prevents a reliable assessment of its true practical relevance. ZnO/N/KB also remains difficult to classify because of incomplete electrolyte and structural information. Overall, heterointerface, phase‐coupling, and lightweight conductive‐network engineering appear more promising than simply increasing architectural complexity. Future ZnO‐based separator design should prioritize low coating mass, reduced thickness, lean‐electrolyte compatibility, and complete parameter reporting to distinguish genuine cornerstone candidates from systems whose high apparent PRI primarily results from unreported structural penalties.

## 3 Metal Sulfide‐Based Separator Coating Materials

Compared with metal oxides, transition‐metal sulfides generally exhibit higher electronic conductivity and more favorable catalytic properties, enabling faster polysulfide conversion kinetics [45, 46]. The strong affinity between sulfide surfaces and sulfur species further facilitates effective polysulfide immobilization through metal‐sulfur interactions. As a result, various metal sulfides, including CoS_x_ [160, 161], MnS [162], MoS_2_ [163], and WS_2_ [164, 165], have been widely investigated as functional separator coatings. Representative metal sulfide‐based separator systems are summarized in Table S6.

### 3.1 Cobalt Sulfide

#### 3.1.1 Mechanistic Evolution of Cobalt Sulfide‐Based Inorganic Separator Systems for Polysulfide Regulation

Polar transition‐metal sulfides, represented by cobalt sulfide (CoS_2_), have attracted extensive attention as separator modification materials for Li–S batteries due to their combined advantages of strong chemical adsorption, high electrocatalytic activity, and favorable intrinsic electrical conductivity [166]. These characteristics enable CoS_2_ to effectively regulate polysulfide chemistry while maintaining fast electron transport, making it a highly promising multifunctional material for functional separators. In recent years, systematic strategies involving structural engineering, interfacial design, electronic‐structure modulation, and conductive network construction have been developed to optimize CoS_2_ performance, driving its evolution from a single‐function material toward multifunctional integrated systems [167].

Designing multidimensional nanostructures represents the fundamental strategy for enhancing the overall performance of CoS_2_‐based systems. Three‐dimensional conductive network structures, such as the MWCNTs@N‐doped‐C@CoS_2_ sandwich architecture [168], the CoS_2_‐NC@CNT 3D network [169], and the separator‐free self‐supporting 3D CoS_2_/rGO aerogel [170], utilize conductive carbon frameworks to provide rapid electron transport pathways while uniformly dispersing CoS_2_ catalytic sites. These architectures integrate adsorption and catalytic conversion within a single spatial framework. Hollow and hierarchical porous structures further emphasize physical confinement and interfacial optimization. For example, hollow multishelled CoS_2_ structures achieve stepwise blocking and catalytic conversion of LiPSs through multiple shell layers, exhibiting excellent cycling stability (Figure 10a,b) [171]. Similarly, biomass‐derived 3D porous carbon loaded with CoS_2_ possesses an ultrahigh specific surface area of 2910 m^2^ g^−1^ and abundant pore structures, enabling efficient polysulfide confinement and catalytic conversion within the pore channels while maintaining ideal performance even at high sulfur loading (Figure 10c) [172]. Additional designs, including CoS_2_ intercalated or coated montmorillonite composites [173] and hierarchical porous CoS_2_‐NiS_2_ heterostructures [174], further extend structural engineering from individual material design toward coordinated host‐separator system integration. Constructing heterointerfaces that induce strong electronic coupling has become a key strategy for enhancing the catalytic kinetics of CoS_2_. Ren et al. [175] designed a CoS_2_/ZnS heterostructure rich in p‐n junctions, where the built‐in electric field formed at the interface not only enhances polysulfide adsorption and bidirectional catalytic conversion but also promotes uniform lithium‐ion distribution. As a result, both the shuttle effect and lithium dendrite growth are effectively suppressed (Figure 10d,e). A similar mechanism has been reported in FeS_2_‐CoS_2_ heterostructures, where interfacial charge redistribution strengthens polar adsorption and induces uniform lithium deposition, thereby improving both cycling stability and safety [176]. In addition, CoS_2_/NC@1T‐MoS_2_ materials induce a phase transition of MoS_2_ from the semiconducting 2H phase to the metallic 1T phase through interfacial interactions, significantly enhancing electrical conductivity and catalytic activity [177]. Likewise, CoS_2_‐MoS_2_/CNT heterostructures further strengthen catalytic activity through interfacial carrier regulation and activation of sulfur vacancies [178].

**FIGURE 10:**
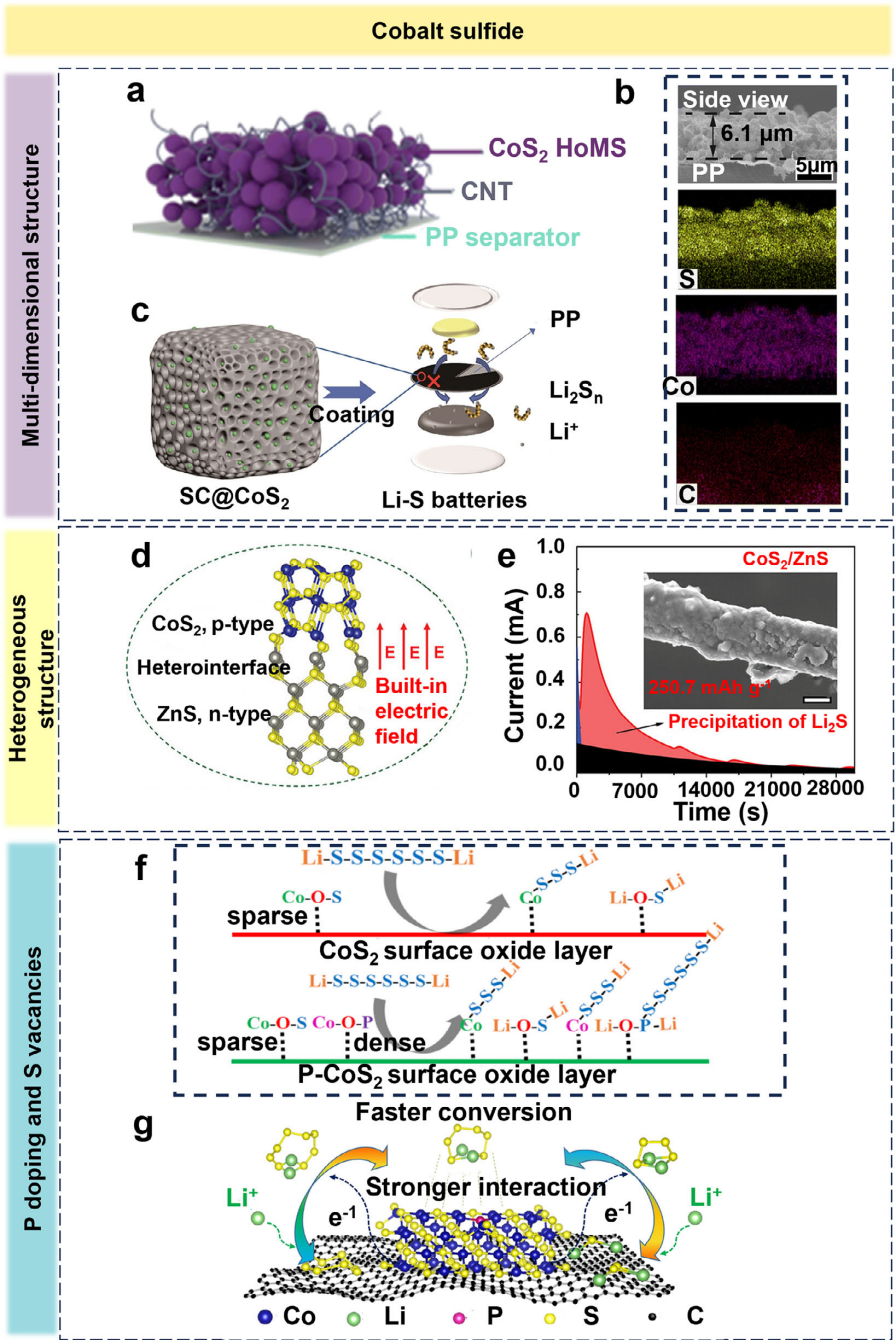
Cobalt Sulfide modified separator strategy. (a) Schematic diagram of the CoS_2_ HoMS‐coated separator. (b) Cross‐section SEM images of the CoS_2_ HoMS‐coated separator, as well as the mapping image. Reproduced with permission [171]. Copyright 2024, Royal Society of Chemistry. (c) Scheme illustration of synthesis processes of SC@CoS_2_ and its application as functional interlayer in Li–S batteries. Reproduced with permission [172]. Copyright 2024, Wiley‐VCH. (d) Schematic diagram of the synthesis of the CoS_2_/ZnS heterostructure embedded with N‐doped carbon nanosheets. (e) The constant potential discharge (Li_2_S precipitation) curve of CoS_2_/ZnS and pure CoS_2_ electrodes at 2.05 V. Reproduced with permission [175]. Copyright 2023, Wiley‐VCH. (f) Schematic illustration of advantages of P‐Co_9_S_8_@SC interaction with LiPSs. Reproduced with permission [179]. Copyright 2021, Elsevier (g) Proposed adsorption schemes of Li_2_S_6_ on CoS_2_ and P‐CoS_2_ surfaces. Reproduced with permission [180]. Copyright 2021, American Chemical Society.

Precise modulation of the electronic structure of CoS_2_ through heteroatom doping or defect engineering provides an effective approach to optimizing adsorption and catalytic active sites. Phosphorus doping is a representative strategy. For example, the P‐Co_9_S_8_@SC composite regulates the electronic structure of Co_9_S_8_ through P doping (Figure 10f), achieving an extremely low‐capacity decay rate of only 0.05% per cycle after 900 cycles at 1 C and delivering high areal capacity under high sulfur loading [179]. Similarly, studies such as NTs@Celgard [180] demonstrate that P doping modifies the electronic distribution of Co─S bonds, enhancing Lewis acidity and accelerating polysulfide conversion (Figure 10g). Other doping strategies, including B‐doped ZnS/CoS_2_ heterostructures [181] and Cu‐doped CoS_2_ nanocages [182], activate additional catalytic sites through enhanced Lewis acidic centers or lattice distortion. Moreover, introducing anion vacancies, such as sulfur vacancies in CoS_2‐x_, can generate additional Co^3+^ Lewis acid sites, significantly strengthen polysulfide adsorption, and lower the nucleation energy barrier of Li_2_S, making vacancy engineering an effective strategy for catalytic regulation [174]. Integrating CoS_2_ with other functional components within highly conductive frameworks is essential for achieving synergistic adsorption‐catalysis‐transport regulation. Multicomponent composite systems such as CoS_2_‐MoS_2_ structures combine the strong adsorption capability of MoS_2_, the high catalytic activity of CoS_2_, and the electrical conductivity of CNT networks [183]. Similarly, CoS_2_‐MgS composites construct low‐resistance pathways by utilizing the ultrafast lithium‐ion transport capability of MgS [161]. Heterostructures including CoS_2_‐WS_2_ [184] and CoS_2_‐CoSe_2_ [185] further demonstrate the advantages of interfacial synergy for achieving both high‐rate capability and long cycle life. Regarding conductive network construction, rGO (e.g., CoS_2_/rGO interlayers [186]) and CNT frameworks (e.g., CoS_2_‐NC@CNTs [169]) are commonly used as 2D and 1D conductive scaffolds. Emerging studies using MXene frameworks, such as MCCoS/PP [187] and N‐doped MXene‐CoS_2_ nanohybrids [188], exploit the high conductivity and surface functional groups of MXenes to achieve strong interfacial coupling. Furthermore, 3D open frameworks such as aerogels and sponge‐like carbon structures (e.g., CoS_2_‐NCA@C [189] and P‐Co_9_S_8_@SC [179]) further enhance electrolyte wettability and overall electron/ion transport efficiency [190].

The development of CoS_2_‐based separator materials has established a systematic technological framework that integrates multidimensional structural engineering, heterointerface catalytic modulation, atomic‐scale electronic structure tuning, and the incorporation of multicomponent conductive networks. This evolution signifies a clear shift from conventional physical confinement and adsorption mechanisms toward deeper chemical catalytic processes, as well as from single‐material optimization to synergistic, multifunctional system design. With a growing understanding of interfacial coupling effects and defect‐induced catalytic behavior, alongside continuous progress in scalable fabrication techniques, CoS_2_‐based composites are poised to play an increasingly vital role in enabling high‐performance and safer practical Li–S batteries.

#### 3.1.2 PRI‐Guided Evaluation of Cobalt Sulfide‐Based Inorganic Separator Systems: From Mechanistic Advantage to Practical Limitation

To systematically compare sulfide‐based separator systems, representative CoS_2_‐derived architectures are summarized in Table 5 and evaluated using the PRI framework, enabling a unified assessment of their practical relevance under consistent performance and structural constraints.

**TABLE 5:** Representative cobalt sulfide‐based inorganic separator systems for Li–S batteries, summarizing key electrochemical parameters and practical operating conditions, including sulfur loading, electrolyte‐to‐sulfur ratio, areal capacity, coating mass, separator thickness, and device‐level validation. All systems are further evaluated using the PRI, which integrates energy contribution, lean electrolyte compatibility, structural penalty, and device validation into a unified constraint‐based framework (Table S7). This table highlights representative architectures that illustrate the evolution from adsorption‐dominated designs to defect‐engineered and heterostructure‐based systems.

System	Mechanism	Sulfur loading (mg cm^−2^)	E/S (μ L mg^−1^)	Areal capacity (mAh cm^−2^)	Testing condition (rate, cycles)	Coating mass (mg cm^−2^)	Thickness (μ m)	Pouch cell	Refs.
MWCNTs@N‐doped‐C@CoS_2_/PP	Adsorption‐catalysis synergistic	5.0	15	3.43	0.1C, 50 cycles	0.12	/	/	[168]
CoS_2_‐MoS_2_‐CNT/PP	Heterojunction/defect‐engineered	1.4	20	1.69	0.2C, 100 cycles	/	4.15	/	[178]
P‐Co_9_S_8_@SC/PP	Band structure modulated	5.6	14.3	3.37	0.1C, 50 cycles	0.17	12	/	[179]
NSPCFS@CoS_2_@C‐200	Adsorption‐catalysis synergistic	1.4	20	1.1	0.1C, 100 cycles	1.4	10	/	[180]
NSPCF@CoS_2_@C‐150	Adsorption‐catalysis synergistic	2.04	20	1.92	0.1C, 100 cycles	/	10	/	[191]
N‐MX‐CoS_2_/PP	Adsorption‐catalysis synergistic	1.2	16.7	1.24	1C, 700 cycles	0.2	15	/	[189]
P‐CoS_2_/CNTs@Celgard	Band structure modulated	4.8	20	4.5	0.2C, 100 cycles	0.63	/	/	[181]
CoS_2_@Montmorillonite/PP	Adsorption‐catalysis synergistic	4.0	8.5	3.41	0.2C, 100 cycles	0.5	19	/	[192]
CoS_2_/NC@1T MoS_2_/PP	Heterojunction/defect‐engineered	0.8	15	0.78	0.1C, 100 cycles	1.0	8.5	/	[193]
CoS_2_‐NC@CNTs/PP	Adsorption‐catalysis synergistic	1.0	20	0.85	0.1C, 100 cycles	1.4	21.4	/	[169]
CoS_2_/rGO/PP	Adsorption‐catalysis synergistic	3.1	5	1.81	0.1C, 100 cycles	0.19	4.8	/	[194]
CoS_2_‐CoSe_2_/GN/PP	Heterojunction/defect‐engineered	1.0	15	1.13	0.2C, 350 cycles	0.17	11	/	[186]
CNFs/CoS_2_‐X/PP	Heterojunction/defect‐engineered	4.42	20	3.04	0.1C, 70 cycles	0.53	11	/	[195]
B‐ZnS/CoS_2_@CS/PP	Band structure modulated	6.14	8	6.22	0.2C, 120 cycles	0.4	5.67	/	[196]

The PRI‐based evaluation of cobalt sulfide‐based separator systems reveals a highly heterogeneous performance landscape governed by a strong competition between catalytic activity and structural design. Although sulfide systems generally exhibit high intrinsic activity toward polysulfide conversion, their practical relevance is significantly influenced by structural configuration and interfacial engineering. Among all investigated systems, only a limited number of hybrid architectures, particularly B‐ZnS/CoS_2_@CS/PP, achieve simultaneously high energy contribution, acceptable electrolyte utilization, and manageable structural penalty, positioning them as near‐cornerstone candidates under the PRI framework. In contrast, most multi‐component sulfide composites suffer from excessive structural complexity and inactive mass accumulation, which substantially reduce their PRI values despite moderate catalytic activity, leading to compromise‐dominated behavior under practical conditions. Overall, sulfide‐based separator systems are best described as “catalysis‐rich but structure‐sensitive” architectures, where only optimized heterostructures can effectively translate intrinsic electrochemical advantages into practical applicability.

### 3.2 Manganese Sulfide

#### 3.2.1 Mechanistic Evolution of Manganese Sulfide‐Based Inorganic Separator Systems for Polysulfide Regulation

Manganese sulfides (MnS) have attracted considerable attention as separator modification materials for Li–S batteries owing to their moderate polarity, abundant active sites, and favorable chemical stability. The research strategy of MnS‐based systems has undergone a clear evolution from passive adsorption to catalytic conversion and further toward precise electronic‐state regulation, gradually transforming MnS from a simple “polysulfide catcher” into an active “sulfur reaction kinetics regulator”.

Early studies mainly exploited the thiophilic nature of MnS to chemically adsorb lithium LiPSs, aiming to physically and chemically intercept their shuttle migration. For example, Wang et al. [196] prepared MnS/carbon nanofiber interlayers through electrospinning followed by carbonization. In this flexible interlayer, MnS nanoparticles provide strong chemical adsorption sites, while the continuous carbon nanofiber network ensures efficient electron and ion transport. This design effectively improves cycling stability and significantly suppresses self‐discharge. At this stage, the strategy was largely adsorption‐dominated, with relatively limited functionality. As understanding of Li–S reaction mechanisms deepened, it became evident that adsorption alone was insufficient to meet the performance requirements of practical batteries. Research therefore shifted toward constructing multifunctional interfaces that combine physical confinement, chemical adsorption, and initial catalytic conversion. A representative example is the MOF‐derived MnS/N‐C@CNT separator coating reported by Li et al. [162] In this system, nitrogen‐doped porous carbon derived from ZIF‐8 serves as the structural framework and is integrated with a conductive carbon nanotube network, while MnS nanoparticles are anchored on the surface. This architecture enables the integration of adsorption and catalytic functions, marking the transition of MnS from a simple “anchoring agent” to a multifunctional interfacial material. To further address the accumulation of LiPSs at the interface, the design strategy evolved toward synergistic adsorption‐catalysis regulation, where MnS actively participates in and accelerates sulfur conversion reactions. A typical example is the yolk‐shell structured MnS@N‐doped carbon composite developed by Ma et al. [197] (Figure 11a). The internal void space generates a confinement effect, while the synergistic interaction between MnS and the N‐doped carbon matrix not only enhances polysulfide adsorption but also significantly lowers the reaction energy barrier for Li_2_S formation (Figure 11b). Consequently, the separator evolves from a passive barrier into an active reaction interface. Further studies revealed that adsorption strength and catalytic activity must be carefully balanced to avoid the so‐called Sabatier dilemma. Zhu et al. [198] constructed oxygen‐vacancy‐rich Mn_3_O_4‐x_ coatings via surface engineering. In this system, polysulfide adsorption is dominated by Li─O bond formation, which preferentially promotes the heterogeneous conversion of LiPSs into solid products (Figure 11c). Meanwhile, the energy barriers of key reaction steps are simultaneously reduced in both directions (Figure 11d), fundamentally optimizing the reaction pathway from a kinetic perspective. Recent research has further advanced toward precise regulation of adsorption strength and catalytic activity through multicomponent heterostructure construction and atomic‐level doping. For example, Li et al. [199] reported a FeS_2_‐MnS/C heterostructure (Figure 11e), in which MnS provides strong polysulfide anchoring while FeS_2_ offers high catalytic activity for accelerated conversion. The interfacial charge redistribution induced by the heterostructure further reduces the overall reaction energy barrier (Figure 11f). More recently, studies have begun to explore electronic‐state regulation at a deeper level. In the Mo‐doped Mo‐MnS/MnO@C system [200], modulation of the electronic structure of Mn enables a more optimal balance between strong adsorption and rapid desorption of reaction intermediates. This design follows the Sabatier principle to avoid excessive stabilization of reaction intermediates and thereby accommodate the demanding conditions of high sulfur loading.

**FIGURE 11:**
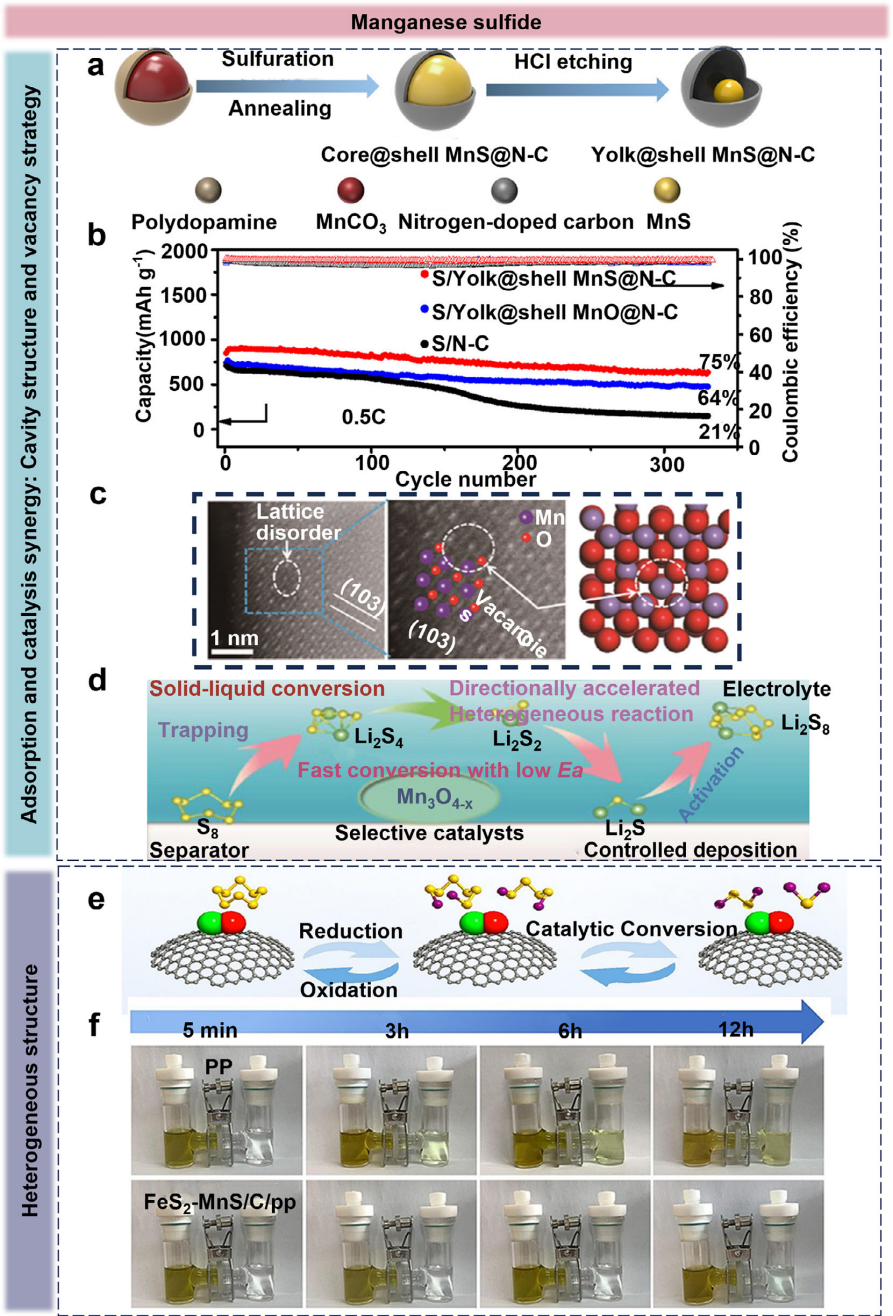
Manganese sulfide‐modified separator strategy. (a) Scheme illustration of the preparation of yolk@shell MnS@N‐C. (b) Long‐term cycling performance of S/yolk@shell MnS@N‐C, S/yolk@shell MnO@N‐C, and S/N‐C. Reproduced with permission [197]. Copyright 2021, American Chemical Society. (c) Atomic‐resolution HAADF‐STEM images and the corresponding structure model of Mn_3_O_4‐x_. (d) Schematic of the protective effect of the Mn_3_O_4‐x_‐catalyzed separator without dendrite (left) and Celgard (right) on a Li anode by adsorption of PSs. PS, polysulfide species; XPS, x‐ray photoelectron spectroscopy. Reproduced with permission [198]. Copyright 2023, Wiley‐VCH. (e) The mechanism diagram of FeS_2_‐MnS/C//PP modified separator catalytic conversion of LiPSs. (f) LiPSs permeation measurement photos of bare PP and FeS_2_‐MnS/C//PP. Reproduced with permission [199]. Copyright 2023, Elsevier.

The evolution of MnS‐based separator materials demonstrates a clear progression from simple chemical adsorption to the development of adsorption‐catalysis synergistic interfaces, and further toward precise regulation of reaction pathways and electronic structures enabled by heterostructure engineering and atomic‐level doping. This advancement reflects a broader shift in the field from macroscopic structural design to microscopic, mechanism‐oriented control, offering valuable insights and versatile material platforms for the rational design of high‐performance functional separators in Li–S batteries.

#### 3.2.2 PRI‐Guided Evaluation of Manganese Sulfide‐Based Inorganic Separator Systems: From Mechanistic Advantage to Practical Limitation

To enable a quantitative comparison of MnS/MnO_x_‐based separator systems, representative architectures are summarized in Table 6 and evaluated within the PRI framework, allowing unified assessment of their energy contribution, electrolyte utilization, and structural penalty under practical constraints.

**TABLE 6:** Representative manganese sulfide‐based inorganic separator systems for Li–S batteries, summarizing key electrochemical parameters and practical operating conditions, including sulfur loading, electrolyte‐to‐sulfur ratio, areal capacity, coating mass, separator thickness, and device‐level validation. All systems are further evaluated using the PRI, which integrates energy contribution, lean electrolyte compatibility, structural penalty, and device validation into a unified constraint‐based framework (Table S8). This table highlights representative architectures that illustrate the evolution from adsorption‐dominated designs to defect‐engineered and heterostructure‐based systems.

System	Mechanism	Sulfur loading (mg cm^−2^)	E/S (μ L mg^−1^)	Areal capacity (mAh cm^−2^)	Testing condition (rate, cycles)	Coating mass (mg cm^−2^)	Thickness (μ m)	Pouch cell	Refs.
MnS/CNFs	Adsorption‐dominated	5.0	25	3.57	0.5C, 200 cycles	0.59	24	/	[201]
FeS_2_‐MnS/C	Heterojunction/defect‐engineered	4.5	/	3.43	0.2C, 100 cycles	4.8	4.8	/	[199]
MnS/N‐C@CNT	Adsorption‐catalysis synergistic	3.3	30.3	1.65	0.5C, 500 cycles	0.31	6	/	[202]
Mn_3_O_4‐x_	Heterojunction/defect‐engineered	7.10	8.4	5.3	01C, 100 cycles	0.19	14	/	[198]
Mo‐doped MnS/MnO@C	Adsorption‐catalysis synergistic	9.68	/	6.97	1C, 50 cycles	1.17	35	/	[200]

The PRI‐based evaluation of manganese sulfide‐based separator systems reveals a strongly compromise‐dominated landscape, where high structural penalties significantly outweigh moderate electrochemical activity. Although certain defect‐engineered Mn_3_O_4_ systems demonstrate improved energy contribution and partial electrolyte compatibility, most composite architectures suffer from excessive inactive mass and thick interfacial layers, leading to severely reduced practical relevance.

Unlike sulfide‐ or oxide‐dominated systems discussed previously, MnS‐based configurations exhibit particularly strong sensitivity to structural design, where increases in coating mass and separator thickness rapidly deteriorate PRI values despite moderate catalytic or adsorption contributions. Overall, MnS/MnO_x_‐based systems can be categorized as predominantly compromise‐dominated, with only a limited number of defect‐engineered oxide systems approaching transition behavior under the unified PRI framework.

### 3.3 Molybdenum Sulfide

#### 3.3.1 Mechanistic Evolution of Molybdenum Sulfide‐Based Inorganic Separator Systems for Polysulfide Regulation

Among the numerous metal compounds used for separator modification in Li–S batteries, molybdenum disulfide (MoS_2_) has attracted significant attention owing to its layered structure, polar surface, and tunable electronic properties [203]. Its role has gradually evolved from a simple physical barrier to a multifunctional platform integrating polysulfide adsorption, catalytic conversion, and interfacial regulation. Accordingly, the research paradigm has shifted from passive polysulfide blocking to active regulation of interfacial reactions [204].

As a representative transition‐metal sulfide, MoS_2_ possesses a typical S‐Mo‐S layered structure that provides a relatively high specific surface area [205]. More importantly, the polarity of Mo─S bonds enables stronger chemical adsorption toward lithium LiPSs compared with conventional carbon materials [206]. Meanwhile, its phase structure (semiconducting 2H and metallic 1T) [207, 208], defect density [209], interlayer spacing [210], and heteroatom doping [211] can be flexibly tuned, offering opportunities to regulate electronic conductivity and catalytic activity. These characteristics allow MoS_2_ to simultaneously perform multiple functions at the separator interface, including polysulfide capture, catalytic conversion, and ion‐transport regulation. Early studies mainly focused on the physical blocking effect of the 2D layered structure of MoS_2_. For instance, Ghazi et al. [212] coated MoS_2_ nanosheets onto a Celgard separator via a simple filtration process, forming a dense additional barrier layer that effectively suppressed the shuttle effect while maintaining lithium‐ion transport, thereby significantly improving cycling stability (Figure 12a). However, simple stacking of 2D nanosheets often leads to restacking, which reduces active‐site utilization and increases ion‐transport resistance. To address this issue, researchers introduced 3D structural designs. Yang et al. [213] prepared 3D flower‐like MoS_2_/graphene microspheres through a spray‐drying method, effectively enhancing surface area and electrolyte wettability. Han et al. [214] further revealed that MoS_2_ nanoparticles with fewer than four layers and abundant edge sites exhibit significantly stronger polysulfide adsorption than bulk MoS_2_. Nevertheless, adsorption‐dominant strategies may lead to accumulation of intermediates on the separator side, potentially causing performance degradation during long‐term cycling.

**FIGURE 12:**
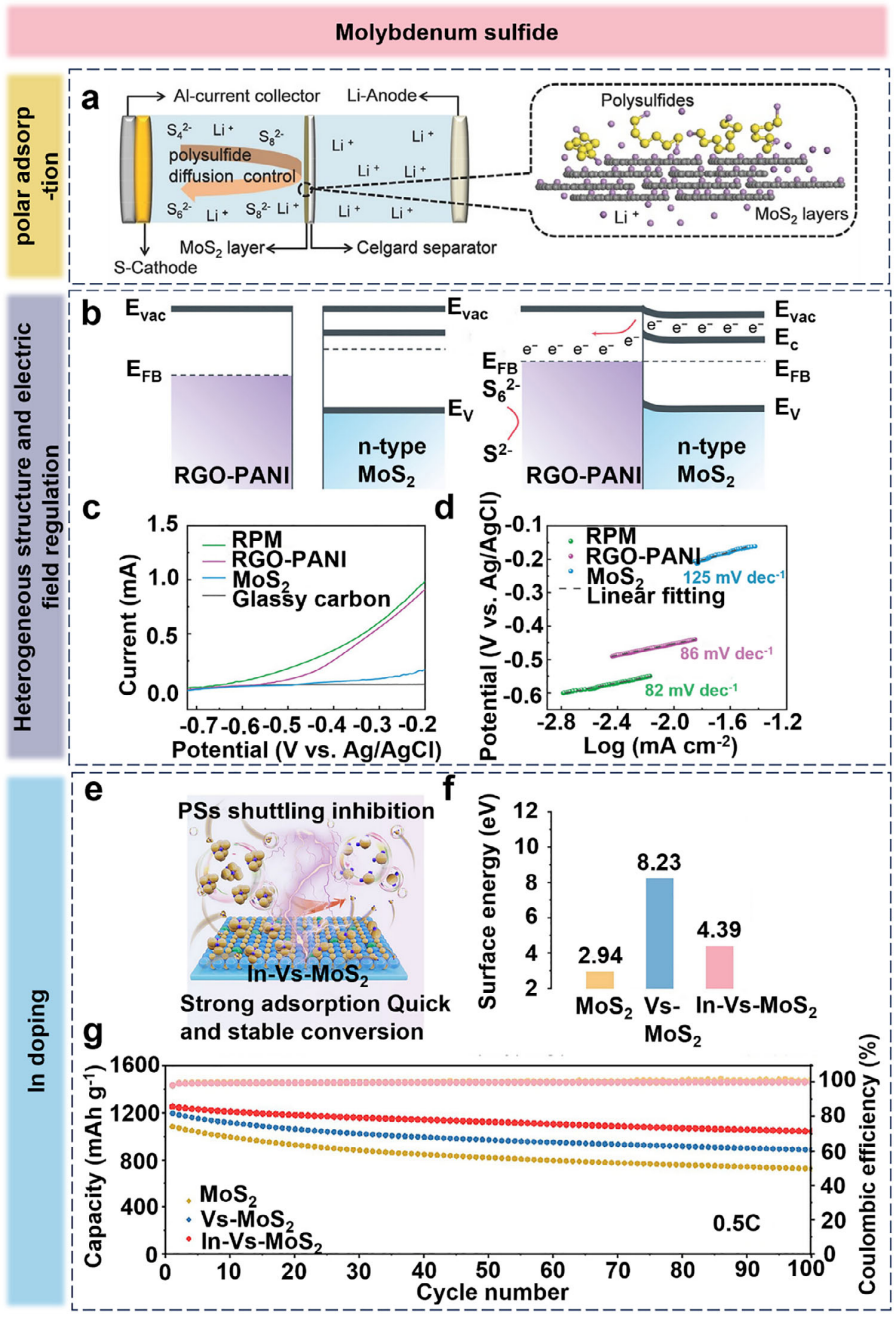
Molybdenum sulfide modified separator strategy. (a) Schematic cell configuration of Li–S batteries using MoS_2_/Celgard separator. Reproduced with permission [212]. Copyright 2017, Wiley‐VCH. (b) Energy band diagrams of RGO‐PANI and n‐type semiconducting MoS_2_ (left) before and (right) after Mott‐Schottky contact formation. Evac is the vacuum level, Ec is the conducting band minimum, EFB is the flat band, and Ev is the valence band. (c) LSV plots of Li_2_S oxidation with (d) the corresponding Tafel plots on RPM, MoS_2_, and RGO‐PANI surfaces. Reproduced with permission [215]. Copyright 2022, Wiley‐VCH. (e) The design principle of In‐Vs‐MoS_2_ catalyst and the schematic diagram of the “rapid stable conversion” mechanism. This diagram visually demonstrates the efficient adsorption and stable catalytic conversion process of LiPSs through the synergistic construction of inner indium (In) atoms and surface sulfur vacancies (Vs). (f) Comparison of surface energies of MoS_2_, Vs‐MoS_2_ and In‐Vs‐MoS_2_. (g) Comparison of the long‐cycle performance of Li–S batteries with separators modified by different catalysts at a current density of 0.5 C. Reproduced with permission [216]. Copyright 2025, Wiley‐VCH.

A key breakthrough was the recognition of the catalytic conversion capability of MoS_2_. It has been shown that MoS_2_ enriched with edge sites, defects, or metallic phases can significantly reduce the energy barrier for the conversion of LiPSs to Li_2_S, thereby accelerating reaction kinetics and mitigating the shuttle effect at its origin [209, 217]. A representative example is the Mott‐Schottky heterostructure RGO‐PANI/MoS_2_ reported by Shi et al. [215], where interfacial charge redistribution forms a built‐in electric field (Figure 12b), significantly enhancing both adsorption strength and conversion kinetics (Figure 12c,d). Similarly, the MoS_2_‐MoO_3_ heterojunction constructed by Lei et al. [218] achieves synergistic enhancement of strong polar adsorption and high catalytic activity, maintaining excellent stability even under high sulfur loading. These studies demonstrate the transition from simple physical blocking toward adsorption‐catalysis dual‐functional regulation. With further advances, interfacial engineering has evolved from simple component stacking to mechanism‐oriented design. The CoS_2_‐MoS_2_@CNT heterostructure reported by Zhang et al. [178] revealed that phase transformation and defect enrichment at the CoS_2_/MoS_2_ interface are key factors responsible for improved catalytic kinetics. Meanwhile, the Bi_2_S_3_‐MoS_2_ heterostructure developed by Li et al. [219] showed that introducing Bi_2_S_3_ not only enhances adsorption but also lowers the nucleation and decomposition energy barriers of Li_2_S through electronic‐structure modulation of MoS_2_. Liu et al. [200] further proposed an “in situ reconstruction‐catalyst regeneration” mechanism, where the formation of multivalent Mo_2_S_3_/MoS_2_ composite phases during cycling continuously maintains catalytic activity, enabling a high areal capacity of 5.8 mAh cm^−2^. Additional examples include the MoS_2_‐WS_2_@MXene heterostructure developed by Li et al. [220], where the electron‐injection effect of MXene and enhanced orbital hybridization significantly reduce the conversion energy barrier. Similar concepts are also reflected in systems such as MoS_2_@SiO_2_ core‐shell structures [221] and hollow ZnS‐SnS_2_@MoS_2_ heterostructures [206]. Recent studies further emphasize precise defect regulation and external physical‐field coupling. Jiang et al. [216] reported indium‐doped sulfur‐vacancy‐stabilized MoS_2_ (Figure 12e), demonstrating that indium doping reduces the surface energy of sulfur vacancies and stabilizes the defect structure from a thermodynamic perspective (Figure 12f). The resulting In‐Vs synergistic active centers exhibit both high catalytic activity and structural stability, enabling a reversible capacity of 865 mAh g^−1^ even at a high rate of 5 C (Figure 12g). In addition, Ding et al. [222] reported a ferroelectric‐catalytic composite separator PBTO@PNF‐MoS_2_, where the built‐in electric field generated by BaTiO_3_ not only suppresses polysulfide diffusion but also modulates the electronic structure of MoS_2_ and guides uniform Li^+^ deposition. Meanwhile, Chen et al. [223] reported an ML‐assisted MoS_2_/MoO_3_‐NCS separator, in which a machine‐learning model quantitatively analyzes the influence of synthesis parameters on capacity retention, representing an initial attempt toward data‐driven optimization of the adsorption‐catalysis balance.

Collectively, MoS_2_‐based separator materials have evolved from simple 2D physical blocking to adsorption‐catalysis synergy and further toward heterointerface engineering and precise defect regulation. Current research is expanding toward defect engineering, multiphysical‐field coupling, and data‐driven materials design. In the future, achieving engineering‐level structural stability while maintaining high catalytic activity, as well as simplifying complex material systems to realize system‐level synergy, will be critical for translating MoS_2_‐based separators into practical Li–S battery applications.

#### 3.3.2 PRI‐Guided Evaluation of Molybdenum Sulfide‐Based Inorganic Separator Systems: From Mechanistic Advantage to Practical Limitation

To evaluate the representative separator systems under a unified constraint‐based framework, the corresponding architectures are summarized in Table 7. These systems span multiple material classes and design strategies, including adsorption‐catalysis synergy, heterostructure engineering, and defect or phase modulation. Within the PRI framework, all systems are quantitatively assessed in terms of energy contribution (ECS), electrolyte utilization (LECS), and structural penalty (SPS), enabling a consistent comparison of their practical relevance under identical evaluation criteria. Accordingly, Table S9 provides a unified benchmark for distinguishing cornerstone, transition, and compromise behavior across different separator designs.

**TABLE 7:** Representative molybdenum sulfide‐based inorganic separator systems for Li–S batteries, summarizing key electrochemical parameters and practical operating conditions, including sulfur loading, electrolyte‐to‐sulfur ratio, areal capacity, coating mass, separator thickness, and device‐level validation. All systems are further evaluated using the PRI, which integrates energy contribution, lean electrolyte compatibility, structural penalty, and device validation into a unified constraint‐based framework. This table highlights representative architectures that illustrate the evolution from adsorption‐dominated designs to defect‐engineered and heterostructure‐based systems.

System	Mechanism	Sulfur loading (mg cm^−2^)	E/S (μ L mg^−1^)	Areal capacity (mAh cm^−2^)	Testing condition (rate, cycles)	Coating mass (mg cm^−2^)	Thickness (μ m)	Pouch cell	Refs.
MoS_2_‐NP coated separator	Adsorption‐catalysis synergistic separator	4.0	15	2.1	0.5C, 150 cycles	1.0	25	/	[224]
D‐MoWS‐CNF (Mo_0.5_W_0.5_S_2‐y_)	Heterojunction/defect‐engineered separator	10	10	13.5	0.05C, first cycle	0.3	/	/	[225]
HATM‐NBs@PP	Band structure/built‐in electric field modulated separator	7.43	5	4.70	5 mA cm^−2^, 200 cycles	0.09	3	/	[226]
MoO_2_/MoS_2_@CC	Heterojunction/defect‐engineered separator	7.6	12	5.3	0.2C, 100 cycles	/	/	/	[227]
Edge‐rich MoS_2_/C HMs	Adsorption‐catalysis synergistic separator	6.1	12	2.92	0.5C, 300 cycles	0.34	15	/	[228]
PVDF/C‐MoS_2_ interlayer	Adsorption‐dominated separator	5.0	10	2.5	1C, 1500 cycles	2.5	20	/	[229]
μ FGF‐MoS_2_/C‐TiN	Heterojunction/defect‐engineered separator	4.5	10	2.7	1C, 1000 cycles	1.8	3	/	[230]
SiO_2_@MoS_2_ interlayer	Adsorption‐dominated separator	4.0	15	2.0	1C, 200 cycles	0.8	3	/	[231]
3D MoS_2_ nanospheres (radial)	Adsorption‐catalysis synergistic separator	4.0	15	3.1	0.5C, 100 cycles	1.0	3	/	[232]
MoS_2_@NC (core‐shell)	Band structure/built‐in electric field modulated separator	4.0	15	3.1	1C, 150 cycles	0.6	3.3	/	[233]
MoP‐MoS_2_/PCNFs	Heterojunction/defect‐engineered separator	3.9	10	2.2	1C, 300 cycles	0.5	15	/	[234]
NSCNTs/MoS_2_ (two‐in‐one)	Adsorption‐catalysis synergistic separator	2.5	15	2.0	1C, 1000 cycles	0.5	25		[235]
VGCF/MoS_2_ (VM/PP)	Adsorption‐catalysis synergistic separator	2.5	10	1.8	1C, 600 cycles	0.25	12	/	[236]
Self‐assembled MoS_2_ (3L)	Adsorption‐dominated separator	2.5	20	1.4	0.5C, 400 cycles	0.022	0.04	/	[237]
MoP/MoS_2_@C (F‐PP)	Heterojunction/defect‐engineered separator	2.0	10	1.0	1C, 500 cycles	0.5	15	/	[238]
TiO_2_/C‐MoS_2_	Band structure/built‐in electric field modulated separator	1.5	15	0.8	1C, 1500 cycles	/	/	/	[239]
MoS_2_/BCF	Adsorption‐catalysis synergistic separator	1.5	20	0.7	0.5C, 300 cycles	1.2	150	/	[240]
S@HCS/MoS_2_ (Na‐S)	Adsorption‐dominated separator	1.5	20	0.8	1C, 1000 cycles	0.5	10	/	[241]
1T MoS_2_@CNT (tandem)	Band structure/built‐in electric field modulated separator	1.2	15	0.8	1C, 500 cycles	/	5	/	[145]
MoS_2_/graphene (3D flower)	Adsorption‐catalysis synergistic separator	1.2	15	1.1	0.2C, 100 cycles	0.8	20	/	[242]
NMCS@MoS_2_/S + CNT/CH	Adsorption‐catalysis synergistic separator	1.2	20	0.6	1C, 500 cycles	0.35	5	/	[243]
MoS_2_/NC@PP	Heterojunction/defect‐engineered separator	1.2	20	0.8	0.2C, 100 cycles	0.48	5	/	[244]
MoS_2_‐polymer LbL (M‐P/P)	Adsorption‐catalysis synergistic separator	1.2	15	0.5	1C, 2000 cycles	0.1	3	/	[245]
CNNFs‐MoS_2_ hierarchical	Adsorption‐catalysis synergistic separator	1.25	15	0.6	1C, 500 cycles	0.75	28	/	[246]
MoS_2_/Celgard (Ghazi)	Adsorption‐dominated separator	1.0	20	0.4	0.5C, 600 cycles	0.35	0.35	/	[212]
G@void@MoS_2_/C integrated	Band structure/built‐in electric field modulated separator	0.8	20	0.3	2C, 1000 cycles	0.5	/	/	[247]
rGO/MoS_2_/MoO_2_ (mwGMSMO)	Band structure/built‐in electric field modulated separator	1.2	20	0.6	0.5C, 300 cycles	0.3	10	/	[248]
MoS_2_@SnO_2_ microboxes	Heterojunction/defect‐engineered separator	6.0	9	6.0	0.2C, first cycle	/	/	/	[249]
MF‐GA‐MoS_2_ (1T/2H)	Adsorption‐catalysis synergistic separator	0.8		0.57	1C, 400 cycles	0.6	20	/	[250]
C@MoS_2_	Adsorption‐catalysis synergistic separator	1.3	/	1.30	0.5C, 200 cycles	0.13	/	/	[251]
CNTs@C@1T‐MoS_2_	Adsorption‐catalysis synergistic separator	0.9	/	0.62	1C, 400 cycles	0.57	7.5	/	[252]
Co‐MoS_2_@NC	Band structure/built‐in electric field modulated separator	1.0	40	0.55	1C, 1000 cycles	0.4	3	/	[253]
CoS_2_/NC@1T MoS_2_	Adsorption‐catalysis synergistic separator	0.8	15	0.49	1C, 500 cycles	1.0	8.5	/	[254]
MoS_2_/Al_2_O_3_@CNF	Adsorption‐catalysis synergistic separator	4.05	4.8	2.65	0.5C, 1000 cycles	0.4	23	/	[255]
MoS_2_@CF‐NRGO (Mo‐O‐C)	Band structure/built‐in electric field modulated separator	8.0	10	5.58	0.2C, 50 cycles	0.3	10	(S: 337mg, 3.7mAh cm^−2^, 0.1C, 40 cycles)	[256]
MXene/MoS_2_	Adsorption‐catalysis synergistic separator	5.0	7	3.50	1C, 200 cycles	0.4	16	/	[257]
O‐MoS_2_/CNS (O‐doped)	Heterojunction/defect‐engineered separator	8.2	/	4.50	0.5C, 100 cycles	/	16.8	(S: 5.1 mg, 3.7 mAh cm^−2^, 0.1C, 40 cycles)	[258]
P‐MoS_2_ (3D network)	Heterojunction/defect‐engineered separator	3.7	15	3.27	0.1C, 100 cycles	0.3	20	/	[259]
MoS_2_/MoO_3_@CNT	Heterojunction/defect‐engineered separator	8.7	10	6.61	0.2C, 100 cycles	0.3	12	(6.6mAh cm^−2^, 0.2C, 100 cycles)	[260]
Bi_2_S_3_‐MoS_2_‐PP	Band structure/built‐in electric field modulated separator	5.5	9	2.5	3C, 500 cycles	/	12	/	[219]
CoS_2_‐MoS_2_‐CNT	Adsorption‐catalysis synergistic separator	3.6	7	2.3	0.2C, 200 cycles	/	/		[163]
CoS_2_‐NC‐CNTs@MoS_2_	Heterojunction/defect‐engineered separator	7.2	5.6	2.7	0.2C, 50 cycles	2./	16	/	[261]
Mo_2_S_3_‐MoS_2_@CNFs	Heterojunction/defect‐engineered separator	4.5	5	3.3	0.1C, 100 cycles	0.45	15	/	[204]
MoS_2_@SiO_2_	Heterojunction/defect‐engineered separator	2.0	/	1.6	0.2C, 80 cycles	1.55	/	/	[262]
NiS_2_/1T‐MoS_2_	Heterojunction/defect‐engineered separator	1.0	7	0.8	0.2C, 100 cycles	/	/	/	[263]
rGO@MoS_2_	Adsorption‐catalysis synergistic separator	4.1	11.9	2.1	0.1C, 200 cycles	0.40	13		[264]
ZnS‐SnS_2_@MoS_2_	Adsorption‐catalysis synergistic separator	4.3	/	2.5	0.2C, 100 cycles	0.4	8	/	[265]
1T‐MoS_2_@PC	Heterojunction/defect‐engineered separator	4.78	8	3.9	0.05C, 40 cycles	0.4	11.7	/	[266]
CoP@MoS_2_	Band structure/built‐in electric field modulated separator	4.0	10	3.3	0.2C, 200 cycles	1.0	/	/	[267]
In‐Vs‐MoS_2_	Heterojunction/defect‐engineered separator	8.7	/	10.0	0.2C, 60 cycles	0.12	4.6	(S: 8.7 mg cm^−2^, 10 mAh cm^−2^, 150 mA g^−1^, 60 cycles)	[216]
MoSe_2_/MoS_2_@CNTs‐PP	Band structure/built‐in electric field modulated separator	3.8	10.5	2.8	0.1C, 100 cycles	/	/	/	[268]
MoS_2_/MoO_3_‐NCS	Heterojunction/defect‐engineered separator	8.4	6	7.25	0.1C, 100 cycles	1.05	18	(S: 8.4 mg cm^−2^, 6.53 mAh cm^−2^, 0.1C, 100 cycles)	[269]
M‐TiN/MoN	Band structure/built‐in electric field modulated separator	5.12	10	5.91	0.1C, 80 cycles	/	6.55	(S: 5.12 mg cm^−2^, 3.67 mAh cm^−2^, 0.1C, 80 cycles)	[270]
ZnS‐NC@MoS_2_	Adsorption‐catalysis synergistic separator	5.1	11.8	/	0.2C, 150 cycles	/	9	(S: 5.1 mg cm^−2^, 0.2C, 150 cycles)	[271]

The PRI‐based evaluation of MoS_2_‐based and MXene‐integrated separator systems reveals the most heterogeneous performance landscape among all material classes analyzed in this work. This diversity originates from the coexistence of multiple functional mechanisms, including adsorption‐dominated confinement, catalytic polysulfide conversion, and band‐structure or interfacial electric field modulation. Despite the generally favorable intrinsic activity of MoS_2_ and its derivatives toward polysulfide regulation, the PRI results clearly indicate that practical performance is not governed by catalytic activity alone. Instead, structural complexity and electrolyte utilization efficiency play a decisive role in determining overall system relevance. Many highly engineered architectures, although exhibiting enhanced ECS values, suffer from significant structural penalties or insufficient electrolyte compatibility, resulting in reduced PRI scores. Notably, only a limited number of systems, such as MXene/MoS_2_ hybrids and optimized 1T‐phase or heterostructure‐engineered configurations, achieve a relatively balanced optimization between ECS, LECS, and SPS. These systems can be classified as near‐cornerstone or strong transition candidates, as they successfully translate intrinsic catalytic advantages into improved practical relevance. In contrast, the majority of MoS_2_‐based composites fall into the compromise‐dominated regime, where excessive structural engineering, inactive mass accumulation, or insufficient electrolyte optimization outweigh the benefits of enhanced catalytic activity. Overall, MoS_2_‐based systems represent a “maximum diversity but maximum dispersion” class within the PRI framework, where practical performance is governed by a strong coupling between catalytic capability and structural design constraints. Only systems achieving simultaneous optimization of ECS, LECS, and SPS can be regarded as true cornerstone candidates under realistic operating conditions.

### 3.4 Tungsten Sulfide

#### 3.4.1 Mechanistic Evolution of Tungsten Sulfide‐Based Inorganic Separator Systems for Polysulfide Regulation

Among 2D transition‐metal sulfides employed for separator modification in Li–S batteries, tungsten disulfide (WS_2_) has recently attracted increasing attention due to its suitable band structure, abundant edge‐active sites, and tunable crystal phase and electronic properties [272]. The evolution of its application strategy clearly reflects a transition from simple physical blocking toward interfacial catalysis and electronic‐structure regulation, representing a progressive deepening of design principles for functional separators [273].

Early studies primarily utilized the polar surface of WS_2_ to chemically adsorb lithium LiPSs, thereby suppressing the shuttle effect. In 2018, the C‐WS_2_ bilayer separator strategy was reported [274], where polar WS_2_ was combined with a conductive carbon layer to achieve effective polysulfide trapping while maintaining efficient ion transport (Figure 13a,b). In the same year, Paolella et al. [275] provided mechanistic insights into the possible interfacial chemical reactions between WS_2_ and LiPSs, emphasizing for the first time that separator‐modifying materials should not be regarded as inert hosts but rather as active interfacial participants in electrochemical reactions. Subsequently, doping and composite strategies were introduced to further enhance adsorption and catalytic kinetics. For example, a Ni‐doped WS_2_@rGO separator system significantly improved both chemical anchoring and catalytic conversion capabilities through metal doping and the construction of a conductive network [165]. As the understanding of kinetic limitations deepened, research focus gradually shifted toward structural design and crystal‐phase regulation to achieve multifunctional synergy. For instance, the WS_2_/CNT 3D conductive network reported in 2021 [276] and the WS_2_@PB‐PPy ternary composite separator [277] both employed porous conductive frameworks to integrate adsorption, electron transport, and catalytic conversion. In the same year, studies on high‐percentage metallic 1T‐phase WS_2_ demonstrated from a phase‐engineering perspective that metallic 1T WS_2_ significantly enhances electrical conductivity and accelerates polysulfide conversion kinetics [278]. In addition, a WS_2_@C nanoflower core‐shell structure effectively suppressed nanoparticle aggregation through carbon encapsulation while improving mechanical stability, highlighting the importance of morphological design [164]. In recent years, WS_2_‐based separator modification has entered a new stage centered on heterostructure engineering, where interface design is employed to generate built‐in electric fields for deeper optimization of reaction kinetics. In 2023, Wang et al. vertically grew WS_2_ nanosheets on MXene substrates to form MXene@WS_2_ heterostructures [279]. The built‐in electric field generated at the heterointerface significantly reduced internal resistance and improved reaction kinetics, leading to minimized polarization voltage (Figure 13c,d). In 2025, the Ta_2_O_5_/WS_2_@rGO heterostructure was reported as a representative system in which the heterointerface serves as the key “active battlefield.” In this design, Ta_2_O_5_ provides strong adsorption sites while WS_2_/rGO ensures rapid electron transport. The atomically coupled interface enables excellent cycling stability even under high sulfur loading conditions (Figure 13e,f) [280]. In the same year, B‐doped WS_2_@MXene nanosheet arrays introduced synergistic regulation through both doping and structural ordering. Boron doping activates the otherwise inert basal planes of WS_2_, while vertically aligned nanosheet arrays expose abundant active sites. Together with the MXene substrate, this structure achieves efficient polysulfide adsorption, catalytic conversion, and uniform Li_2_S deposition (Figure 13g–i) [275]. In addition, the recently reported CoS_2_‐WS_2_/GNC heterostructure continues the design philosophy of synergistic adsorption‐catalysis‐conductivity regulation [192], demonstrating the continued advancement of this research direction.

**FIGURE 13:**
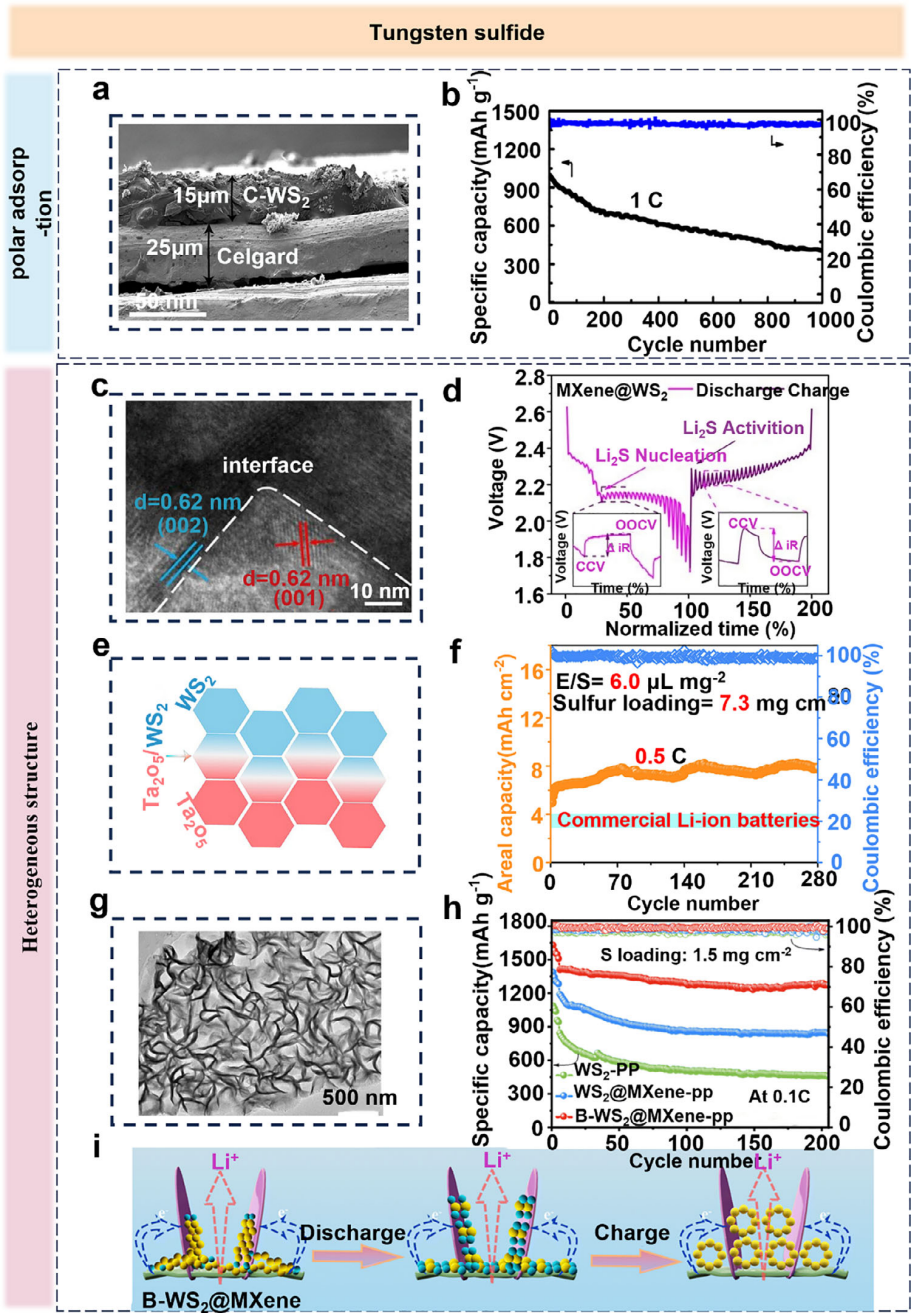
Tungsten sulfide modified separator strategy. (a) SEM images of C‐WS_2_ separator (cross‐section). (b) The long‐cycle performance of Li–S batteries with membranes modified by C‐WS_2_ at a current density of 1 C. Reproduced with permission [274]. Copyright 2022, American Chemical Society. (c) The microscopic interface of the MXene@WS_2_ heterostructure. The image clearly shows the uniform growth of WS_2_ nanosheets on the MXene substrate, as well as the tight and clear bonding interface formed between the two. (d) Analysis of battery polarization based on MXene@WS_2_ separator. Reproduced with permission [278]. Copyright 2023, Wiley‐VCH. (e) Schematic representation of the WS_2_, Ta_2_O_5_, and Ta_2_O_5_/WS_2_ heterostructure. (f) The long‐cycle performance of Ta_2_O_5_/WS_2_@rGO membrane under high‐load and harsh conditions. Reproduced with permission [279]. Copyright 2023, Elsevier. (g) The microscopic morphology of the B‐WS_2_@MXene nanosheet array. The image shows that the boron‐doped WS_2_ nanosheets grow vertically and orderly on the MXene surface, forming a highly open 3D forest‐like array structure. (h) Long‐term cycling performance of B‐WS_2_@MXene, WS_2_@MXene, and WS_2_. Reproduced with permission [280]. Copyright 2023, Royal Society of Chemistry.

The development trajectory of WS_2_‐based separator materials for Li–S batteries clearly outlines an evolution from single polar adsorption toward multidimensional structural and phase engineering, and ultimately toward heterointerface‐mediated electronic‐structure regulation. Through strategies such as heteroatom doping, defect engineering, morphology control, and heterostructure coupling, WS_2_ has gradually evolved from an early passive barrier material into a multifunctional active component capable of regulating interfacial reaction kinetics. These advances provide an important material foundation for the practical realization of high‐energy‐density Li–S batteries.

#### 3.4.2 PRI‐Guided Evaluation of Tungsten Sulfide‐Based Inorganic Separator Systems: From Mechanistic Advantage to Practical Limitation

To evaluate WS_2_‐based and MXene‐integrated separator systems, representative architectures are summarized in Table 8 and analyzed within the PRI framework, enabling a unified assessment of their practical relevance under consistent structural and electrochemical constraints.

**TABLE 8:** Representative tungsten sulfide‐based inorganic separator systems for Li–S batteries, summarizing key electrochemical parameters and practical operating conditions, including sulfur loading, electrolyte‐to‐sulfur ratio, areal capacity, coating mass, separator thickness, and device‐level validation. All systems are further evaluated using the PRI, which integrates energy contribution, lean electrolyte compatibility, structural penalty, and device validation into a unified constraint‐based framework (Table S10). This table highlights representative architectures that illustrate the evolution from adsorption‐dominated designs to defect‐engineered and heterostructure‐based systems.

System	Mechanism	Sulfur loading (mg cm^−2^)	E/S (μ L mg^−1^)	Areal capacity (mAh cm^−2^)	Testing condition (rate, cycles)	Coating mass (mg cm^−2^)	Thickness (μ m)	Pouch cell	Refs.
WS_2_@C‐PP	Adsorption‐catalysis synergistic	1.0	10	0.57	1C, 500 cycles	/	7.5	/	[164]
C‐WS_2_	Adsorption‐dominated	1.5	10	0.62	1C, 1000 cycles	0.79	15	/	[274]
WS_2_‐coated Celgard	Chemical reactivity/catalytic	1.2	/	0.60	0.5C, 300 cycles	4.0	50	/	[275]
WS_2_/CNTs	Adsorption‐catalysis synergistic	1.5	15	1.13	1C, 1000 cycles	0.18	4	/	[276]
Ni‐WS_2_@rGO‐Celgard	Adsorption‐catalysis synergistic + defect engineering	1.44	40	0.74	1C, 500 cycles	0.22	22.2	/	[165]
1T‐WS_2_/rGO	Heterojunction/defect‐engineered	1.2	16.7	0.61	1C, 1000 cycles	0.1	/	/	[281]
MXene@WS_2_	Band structure/built‐in electric field modulated	1.0	/	0.38	2C, 2000 cycles	0.6	/	/	[282]
CoS_2_‐WS_2_/GNC‐PP	Band structure/built‐in electric field modulated + adsorption‐catalysis synergistic	1.2	/	0.64	1C, 1000 cycles	0.3	15	/	[283]
Ta_2_O_5_/WS_2_@rGO‐PP	Heterojunction/defect‐engineered	1.2	/	0.62	2C, 900 cycles	0.22	30	/	[279]
B‐WS_2_@MXene‐PP	Heterojunction/defect‐engineered + band structure modulation	1.5	5	0.93	1C, 1000 cycles	/	6.3	(S: 5 mg cm^−2^, 650mAh g^−1^, 0.5C, 100 cycles)	[280]

The corrected PRI evaluation of WS_2_‐based separator systems further highlights their overall structure‐limited nature, but also reveals a critical exception in MXene‐integrated architectures. While most WS_2_‐based composites remain compromise‐dominated due to severe structural penalties and limited electrolyte utilization efficiency, the inclusion of MXene conductive frameworks enables a significant improvement in system‐level balance. In particular, the B‐WS_2_@MXene‐PP system demonstrates a transition toward near‐cornerstone behavior, achieving a positive PRI value through simultaneous enhancement of energy contribution, electrolyte compatibility, and structural optimization. This suggests that the primary limitation of WS_2_‐based systems is not intrinsic catalytic activity, but rather the lack of efficient electron transport and structural rationalization. Overall, WS_2_‐based systems remain predominantly compromise‐dominated; however, MXene‐assisted heterostructures provide a viable pathway toward transitioning this material class into the cornerstone regime under the PRI framework.

## 4 Metal Nitrides‐Based Separator Coating Materials

Transition‐metal nitrides have recently attracted increasing attention as separator modification materials due to their metallic‐like conductivity and strong polarity [284]. These materials not only provide efficient electron transport pathways but also exhibit strong chemical affinity toward LiPSs, enabling simultaneous adsorption and catalytic conversion [285]. Consequently, various nitrides, such as TiN [286], VN [287], and Mo_2_N [288], have been explored to construct multifunctional separator coatings. Representative metal nitride‐based systems and their electrochemical performances are summarized in Table S11.

### 4.1 Titanium Nitride

#### 4.1.1 Mechanistic Evolution of Titanium Nitride‐Based Inorganic Separator Systems for Polysulfide Regulation

In separator modification for Li–S batteries, early studies mainly relied on porous carbon materials [289]. Although these materials provide physical blocking, their nonpolar nature leads to weak interactions with polar LiPSs, making it difficult to maintain stable performance under high sulfur loading and long‐term cycling. This limitation has driven the exploration of metal compounds with both strong polarity and high conductivity [290]. Among them, transition‐metal nitrides, especially titanium nitride (TiN), have emerged as promising candidates [291]. The development of TiN‐based separator materials has followed a clear evolution from initial material validation to carbon‐composite optimization, and then to heterostructure engineering and multicomponent synergistic systems.

TiN was first introduced into Li–S batteries because of its high intrinsic conductivity and polar surface. In 2016, commercial TiN was treated by HF etching [292], which increased its surface area and exposed more active sites. The resulting Ti─S and N─S chemical bonds enabled strong polysulfide adsorption, providing the first systematic evidence that TiN can suppress the shuttle effect. Later, Qi et al. [293] developed mesoporous TiN microspheres as separator modifiers. Besides physical blocking, the conductive TiN layer acted as an “upper current collector” on the separator side, markedly promoting sulfur redox kinetics and rate capability. To overcome the limited surface area and structural tunability of pristine TiN, combining TiN with porous carbon became an effective strategy. Tang et al. [294] coated a TiN/micro–mesoporous carbon composite on a Celgard separator, where porous carbon provided physical confinement and TiN contributed polar adsorption and rapid electron transport, enabling a reversible capacity of about 500 mAh g^−1^ after 400 cycles at 1 C. Similarly, a nanosized TiN/Super C65 coating delivered stable cycling for 1000 cycles on a PP separator [295]. Other representative examples include TiN/nitrogen‐doped graphene composites [296] and the sandwich‐structured NC‐TiN‐BNC architecture [297], in which boron‐ and nitrogen‐doped carbon fibers sandwich a TiN interlayer, maintaining excellent capacity retention even under high sulfur loading. As the research focus shifted from polysulfide interception to catalytic conversion, heterostructure design became a key strategy. The TiO_2_‐TiN binary heterostructure reported by Zhou et al. [86] separates the roles of TiO_2_ for strong adsorption and TiN for fast electron transport and catalytic conversion, thus enabling a complete “adsorption‐diffusion‐conversion” process at the separator interface. This concept was further extended to device‐level engineering. For example, TiO_2_‐TiN heterostructured nanowires grown on carbonized cotton cloth served simultaneously as current collector and interlayer, supporting ultrahigh areal sulfur loading [298]. A MOF‐derived TiO_2_‐TiN heterostructure integrated with nitrogen‐doped carbon nanosheets (TiON@CNP) [299]. further demonstrated the synergy between porous frameworks and heterostructured nanoparticles. In addition, a bilayer separator based on readily synthesized TiO_2_‐TiN composites [92] highlighted the importance of combining synthetic simplicity with interfacial synergy. More refined structural design has also been introduced to further improve TiN‐based separator performance. In the 1D direction, Kim et al. [300] fabricated TiN nanowires embedded in N‐doped carbon and combined them with CNTs onto a PP separator by vacuum filtration. The resulting 1D conductive pathways enable efficient physical blocking and chemical anchoring simultaneously (Figure 14a,b). Lu et al. [286] prepared ultrathin TiN nanosheets with a thickness of 1.6 nm by topological chemical transformation, forming an adhesive‐free conductive network (Figure 14c). After coating these nanosheets onto the separator, the Li–S battery showed excellent electrochemical performance. After 200 cycles at 0.5 C, the capacity retention remained 769 mAh g^−1^, and the decay rate after 1000 cycles at 1 C was only 0.046% (Figure 14d). The Li_2_S deposition experiment (Figure 14e) showed a deposition capacity of 127.66 mAh g^−1^ for TiN nanosheets, much higher than that of the control sample, confirming their ability to anchor LiPSs and catalyze conversion. Similarly, the 2D TiN@C nanosheet/graphene composite coating designed by Fan et al. [301] also demonstrated the synergy of high surface area and catalytic anchoring. Hollow structures have also been explored to further enhance catalytic activity. For example, Liu et al. [302] designed hollow TiN/1T‐MoS_2_ heterostructured nanospheres (Figure 14f), in which favorable band alignment promotes rapid charge transfer (Figure 14g) and significantly enhances chemical adsorption capability (Figure 14h). Under practical conditions of high sulfur loading and lean electrolyte, TiN‐based separator strategies have further evolved toward multicomponent synergistic architectures and electronic‐structure regulation. TiN/Nb_4_N_5_ heterostructures [235] provide abundant adsorption sites and reduced charge‐transfer resistance through dual‐nitride cooperation. In titanium‐molybdenum nitride solid solutions (TMN@CNF), Mo incorporation strengthens electronic coupling [303]. Co‐doped TiN nanowires [304] combined with high donor‐number electrolytes further highlight the synergy between catalytic interfaces and electrolyte chemistry. Defect engineering has also proved effective: low‐crystallinity TiN [305] contains abundant defects and unsaturated bonds, which strengthen polysulfide adsorption and accelerate Li_2_S nucleation/decomposition. More complex multicomponent systems, such as TiN/CoO dispersed in N‐doped CNT networks [306] and the CoFe/FeN_0.0324_/TiN@N‐CNT heterostructure [307], enable full‐process regulation of polysulfide “adsorption‐diffusion‐conversion.” The latter shows an ultralow capacity decay rate of only 0.048% after 1000 cycles at 1 C (Figure 14i–k). In addition, a 2D TiN/MoN heterostructure [308] has been shown to promote the bidirectional catalysis of Li_2_S nucleation and decomposition.

**FIGURE 14:**
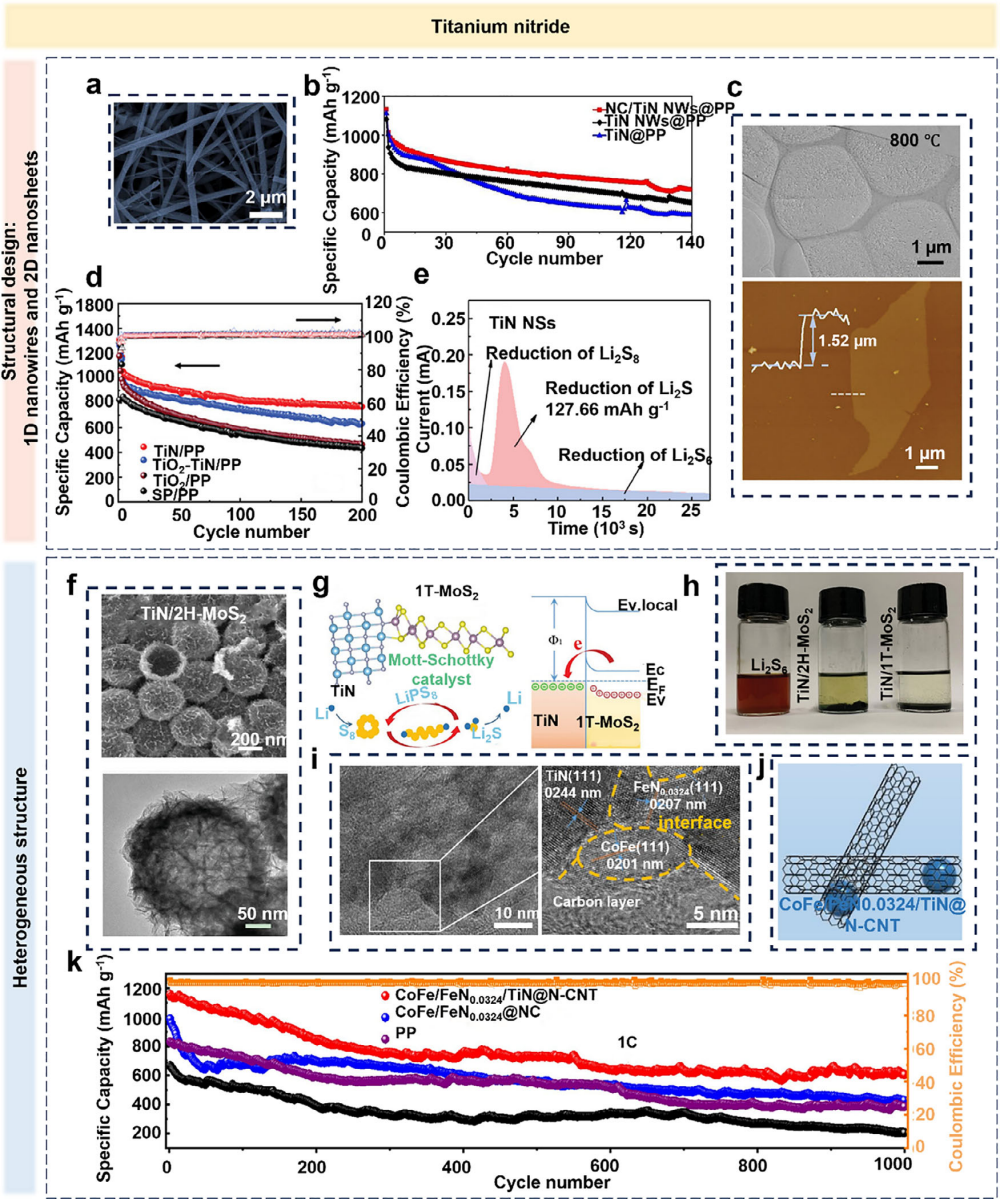
Titanium nitride modified separator strategy. (a) SEM images of the as‐spun NC/TiN NWs. (b) Galvanostatic discharge profiles of NC/TiN NWs@PP, TiN NWs@PP, and TiN@PP in the potential range of 1.7–2.7 V (versus Li^+^/Li) at 0.2 C. Reproduced with permission [300]. Copyright 2021, Elsevier. (c) TEM images show the morphology changes of the nitridation treatment at 800°C for 1 h. (upper) AFM images and the height profiles along the dash lines for TiN‐800°C.(lower). (d) Cycling performance and Coulombic efficiency of Li–S batteries with TiN/PP, TiO_2_‐TiN/PP, TiO_2_/PP, and SP/PP at 0.5 C for 200 cycles. (e) The Li_2_S precipitation experiments of TiN NSs. Reproduced with permission [286]. Copyright 2023, Wiley‐VCH. (f) Scanning and transmission electron microscope images of hollow TiN/2H‐MoS_2_ nanospheres. (g) Theoretical band structure diagram of the TiN/1T‐MoS_2_ heterostructure. (h) Static adsorption comparison experiment of Li_2_S_6_ solution by TiN/2H‐MoS_2_ and TiN/1T‐MoS_2_. Reproduced with permission [302]. Copyright 2023, Wiley‐VCH. (i) Schematic diagram of the CoFe/FeN_0.0324_/TiN@N‐CNT composite material. (j) The high‐resolution transmission electron microscopy (HRTEM) images clearly reveal the tightly heterogeneous interface formed by CoFe, FeN_0.0324_ and TiN. The lattice stripes correspond to the (110), (111) and (111) crystal planes of each material, confirming the successful construction of the heterogeneous structure. (k) The long‐cycle performance graph of Li–S batteries based on CoFe/FeN_0.0324_/TiN@N‐CNT composite material modified separator at 1 C current density. Reproduced with permission [308]. Copyright 2023, Elsevier.

Viewed holistically, TiN‐based separator materials have evolved from pristine TiN to carbon composites, heterostructures, and multicomponent synergistic systems. The key advantage of TiN lies in its combination of high electrical conductivity, strong polar adsorption, and catalytic activity, enabling efficient regulation of polysulfide capture, confinement, and conversion at the separator interface. These merits make TiN an important material platform for high‐energy‐density and long‐lifespan Li–S batteries.

#### 4.1.2 PRI‐Guided Evaluation of Titanium Nitride‐Based Inorganic Separator Systems: From Mechanistic Advantage to Practical Limitation

To enable a unified comparison of separator systems, representative architectures are summarized in Table 9 and evaluated within the PRI framework. This allows a consistent assessment of energy contribution (ECS), electrolyte utilization (LECS), structural penalty (SPS), and device‐level validation (DVS) under identical evaluation criteria, providing a quantitative basis for distinguishing cornerstone, transition, and compromise behaviors across different material systems (Table S12).

**TABLE 9:** Representative titanium nitride inorganic separator systems for Li–S batteries, summarizing key electrochemical parameters and practical operating conditions, including sulfur loading, electrolyte‐to‐sulfur ratio, areal capacity, coating mass, separator thickness, and device‐level validation. All systems are further evaluated using the PRI, which integrates energy contribution, lean electrolyte compatibility, structural penalty, and device validation into a unified constraint‐based framework. This table highlights representative architectures that illustrate the evolution from adsorption‐dominated designs to defect‐engineered and heterostructure‐based systems.

System	Mechanism	Sulfur loading (mg cm^−2^)	E/S (μ L mg^−1^)	Areal capacity (mAh cm^−2^)	Testing condition (rate, cycles)	Coating mass (mg cm^−2^)	Thickness (μ m)	Pouch cell	Refs.
2D TiN NSs	Adsorption‐catalysis synergistic separator	4.3	7.5	3.3	0.2C, 100 cycles	/	17	(S: 2 mg cm^−2^, 1177 mAh g^−1^, 0.1C, first)	[286]
MTN (mesoporous TiN microspheres)	Adsorption‐dominated	1.3	46.2	0.97	0.5C, 200 cycles	1.0	10	/	[293]
TiN/NG	Adsorption‐dominated	1.2	92.6	0.86	1C, 300 cycles	0.5	/	/	[296]
BNC‐TiN‐BNC	Adsorption‐dominated	2.5	80	1.66	1C, 250 cycles	1.3	28	/	[297]
2D TiN@C	Adsorption‐catalysis synergistic	0.71	56.3	/	1C, 600 cycles	0.2	21	/	[301]
TiN/1T‐MoS_2_	Adsorption‐catalysis synergistic	7.0	25	6.13	0.1C, 50 cycles	/	/	/	[302]
TMN@CNF	Heterojunction/defect‐engineered separator	1.1	15	1.0	2C, 1000 cycles	2.0	70	/	[304]
TiN/CoO (TCCNT)	Adsorption‐catalysis synergistic separator	1.0	10	0.9	1C, 500 cycles	/	/	/	[307]
CoFe/FeN_0.0324_/TiN@N‐CNT	Heterojunction/defect‐engineered separator	2.3	/	1.5	0.5C, 400 cycles	0.15	13	/	[308]
M‐TiN/MoN	Heterojunction/defect‐engineered separator	5.12	10	4.7	0.1C, 80 cycles	/	6.55	/	[270]
TiC‐TiN‐TiO_2_	Band structure/built‐in electric field modulated separator	3.7	6	3.0	0.2C, 150 cycles	0.75	16.4	/	[309]
MMC‐TiN	Adsorption‐dominated	0.53	56.6	0.37	1C, 400 cycles	/	15	/	[294]
μ FGF‐MoS_2_/C‐TiN	Adsorption‐catalysis synergistic	2.5	10	2.18	1C, 1000 cycles	1.8	30	/	[230]
h‐ITO@PP	Adsorption‐dominated	3.0	40	1.76	0.5C, 500 cycles	0.25	7	/	[310]
NC/TiN NWs@PP	Adsorption‐catalysis synergistic	1.6	10	1.05	0.2C, 150 cycles	1.1	35	/	[311]
TiN/Nb_4_N_5_	Adsorption‐catalysis synergistic separator	1.0	/	0.8	0.1C, 200 cycles	3.1	23	/	[312]
TiN/PI	Adsorption‐catalysis synergistic separator	4.0	6	2.1	0.1C, 140 cycles	/	25		[313]
L‐TiN‐Cu‐CNF	Heterojunction/defect‐engineered separator	3.8	10	2.5	0.2C, 100 cycles	0.056(TiN) 0.5(CNF)	20	/	[314]
GPC‐TiN‐10	Adsorption‐catalysis synergistic separator	4.1	6	3.4	0.2C, 100 cycles	0.9	45	/	[315]

The PRI‐based evaluation of TiN‐ and TiO_2_‐based separator systems reveals a highly heterogeneous performance landscape, where practical relevance is strongly governed by the balance between electrochemical contribution, electrolyte utilization, and structural penalty. Compared with sulfide‐based systems that mainly rely on intrinsic catalytic conversion activity, TiN/TiO_2_‐derived architectures provide multifunctional advantages through high electrical conductivity, interfacial polarization regulation, and accelerated polysulfide conversion; however, their practical benefits remain highly dependent on structural optimization and device‐level configuration. A considerable fraction of TiN‐based systems exhibits limited PRI values due to the combined effects of insufficient electrolyte compatibility and structural penalties associated with excessive coating mass, thick separator modification, or complex composite construction. These limitations offset the improvements in ECS provided by conductive nitride frameworks. Nevertheless, several engineered architectures demonstrate improved practical potential. For example, TiO_2_‐TiN coupled systems and TiC‐TiN‐TiO_2_ heterostructures achieve relatively favorable PRI characteristics by effectively balancing energy contribution and structural burden, suggesting that rational oxide‐nitride interface integration can provide a promising pathway toward enhanced separator functionality. Similarly, TiN/1T‐MoS_2_ and M‐TiN/MoN systems exhibit high energy contribution capability, although their overall practical relevance requires further validation considering incomplete structural information. Overall, TiN/TiO_2_‐based separator systems display a transition from compromise‐dominated to partially optimized regimes rather than a uniformly limited performance pattern. The PRI analysis highlights that future development should focus not only on improving electronic conductivity or catalytic activity, but also on minimizing structural penalties and optimizing electrolyte efficiency to achieve practical Li–S battery operation.

### 4.2 Molybdenum Nitride

#### 4.2.1 Mechanistic Evolution of Molybdenum Nitride‐Based Inorganic Separator Systems for Polysulfide Regulation

Among various separator modification materials for Li–S batteries, transition‐metal nitrides have attracted extensive attention because of their metallic‐like conductivity and strong polar interactions with lithium LiPSs. Among them, molybdenum nitride (MoN) has gradually emerged as an important research system owing to its unique electronic structure, high electrical conductivity, and strong chemical affinity toward LiPSs [316, 317]. The development of MoN‐based separator materials has followed a clear evolution from initial adsorption‐conductivity dual‐functional design to structural engineering optimization, and further to dynamic interfacial evolution and heterostructure‐regulated catalytic systems.

One of the earliest studies introducing MoN into Li–S battery separators employed a MoN‐graphene bifunctional interlayer strategy [318]. In this system, MoN nanosheets were grown in situ on a graphene substrate and inserted between the sulfur cathode and a commercial separator, forming a conductive and polar functional interlayer (Figure 15a). The MoN nanosheets provide strong chemical anchoring for LiPSs through Mo─S bonding, while the graphene framework reduces interfacial resistance and accelerates sulfur‐species conversion. The corresponding battery delivered an initial capacity of 1061 mAh g^−1^ at 0.5 C and showed a low‐capacity decay rate of only 0.072% per cycle after 500 cycles (Figure 15b). This study demonstrated that MoN in separator structures can serve not only as a passive adsorbent, but also as an active regulator of reaction kinetics through its intrinsic conductivity. To further improve active‐site exposure and structural stability, multidimensional structural design was subsequently introduced. For example, Zhou et al. [319] developed a CCC@MoN multifunctional interlayer, in which carbonized cotton cloth with woven microtubular structures provides physical confinement, while porous MoN nanosheets grown on the surface offer strong chemical adsorption and catalytic conversion capability (Figure 15c,d). This architecture maintained a high areal capacity of 4.2 mAh cm^−2^ at a sulfur loading of 5 mg cm^−2^, demonstrating considerable practical potential (Figure 15e). Similarly, MoN@CNFs prepared by electrospinning followed by in situ hydrothermal treatment and nitridation also illustrate the synergistic advantages of conductive fibrous frameworks and MoN catalytic centers [320]. As separator functional layers were traditionally regarded as static structures, further mechanistic understanding led to the recognition of dynamic interfacial evolution. Yue et al. [321] showed that a magnetron sputtering‐derived MoN‐modified graphene interlayer undergoes in situ evolution during battery operation, forming microreactive heterostructures composed of bulk MoN and edge‐enriched MoS_2_ (Figure 15f,g). In this dynamically reconstructed interface, MoN dominates rapid adsorption and conversion, while MoS_2_ edges provide strong anchoring and confinement, together enabling efficient regulation of the entire polysulfide adsorption‐diffusion‐conversion process. As a result, the battery exhibited an ultralow capacity decay rate of only 0.024% per cycle after 750 cycles under high sulfur loading (4.8 mg cm^−2^), marking a transition from static material design to dynamic interfacial regulation (Figure 15h). Based on these mechanistic insights, heterostructure construction was further employed to regulate electronic states and enhance catalytic activity. For instance, Kong et al. [322] reported a Co/MoN heterostructure‐modified separator (Co/MoN@PP), in which the difference in work functions between Co and MoN induces electron transfer and establishes a built‐in electric field at the interface (Figure 15i). This interfacial field promotes both polysulfide adsorption and catalytic conversion. More importantly, the modified separator also homogenizes the electric field distribution at the lithium anode surface, guiding uniform Li^+^ deposition and suppressing dendrite growth (Figure 15j), thereby achieving integrated adsorption‐catalysis‐regulation synergy across cathode and anode processes (Figure 15k). In addition, a 2D TiN/MoN heterostructure derived from MXene precursors was shown to regulate the d‐band center through interfacial coupling, significantly lowering the energy barriers for Li_2_S nucleation and decomposition [323]. This further establishes MoN as an important catalytic center in heterostructure systems. Compared with other separator modification systems, such as MoO_2_ nanosheets [324], porous organic frameworks (POFs) [325], and single‐atom Co catalysts [326], MoN exhibits distinct advantages in conductivity, adsorption strength, thermal stability, structural robustness, and scalability. From the initial adsorption‐conductivity dual‐functional design to porous structural engineering, dynamic interfacial evolution, and heterostructure‐mediated electronic regulation, MoN‐based separator materials have developed into a relatively complete strategy system.

**FIGURE 15:**
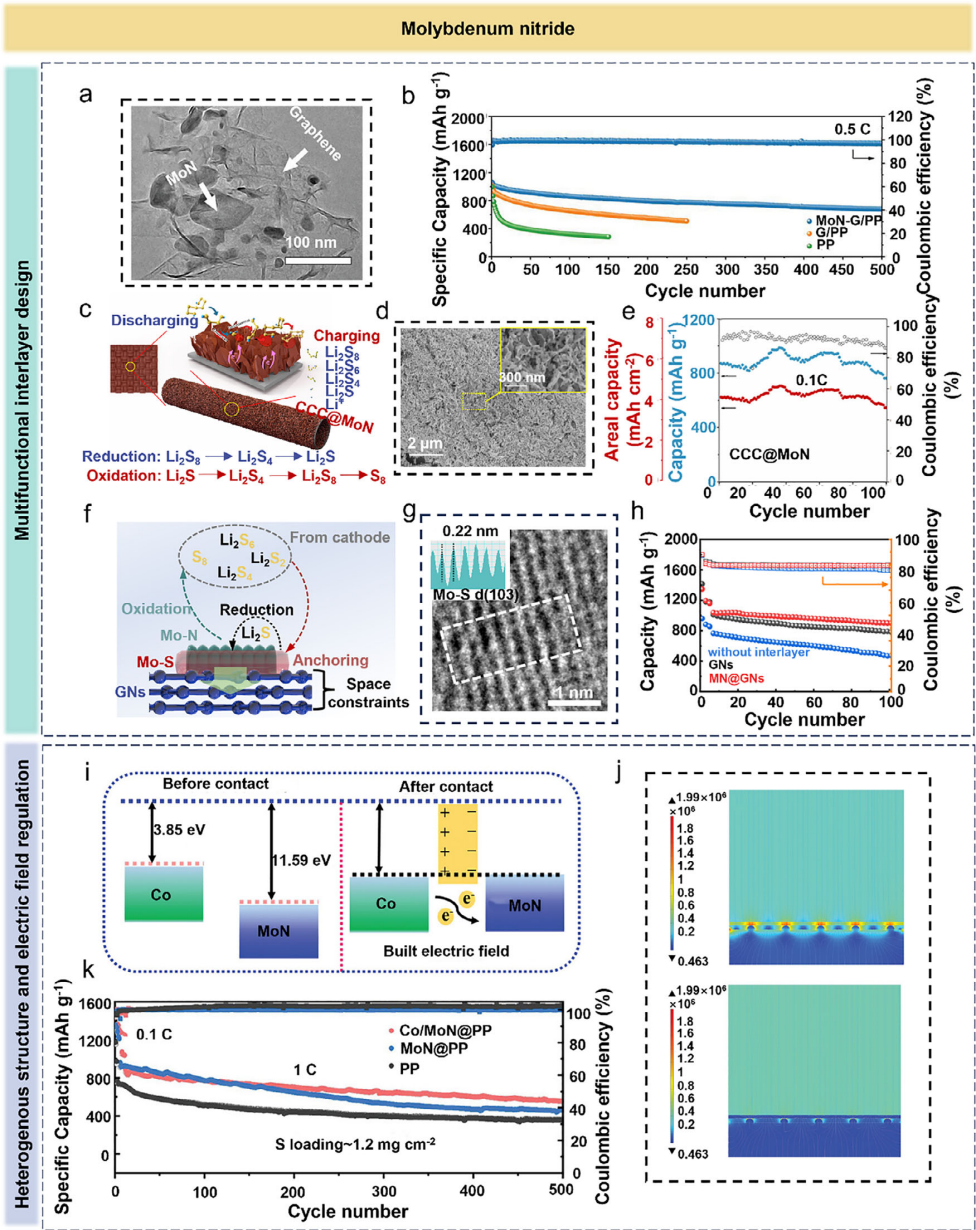
Molybdenum nitride modified separator strategy. (a) TEM images of MoN‐G. (b) Cycling performances of the batteries with MoN‐G/PP, G/PP, and bare PP separators at a current of 0.5 C. Reproduced with permission [318]. Copyright 2023, Wiley‐VCH. (c) Scanning electron microscope (SEM) images of CCC@MoN. (d) This figure shows the microstructure of CCC@MoN after ammonia nitridation treatment. (e) Cycling performance of CCC@MoN‐based Li–S batteries under high sulfur loading. Reproduced with permission [319]. Copyright 2022, Elsevier. (f) Schematic diagram of the synergistic catalytic mechanism of MoN‐MoS_2_ microreactors in the intermediate layer of S‐MN@GNs for LiPSs. (g) High‐resolution transmission electron microscopy (HR) image of the MoS_2_ phase region of MN@GNs after cycling. (h) Comparison of the effects of different intermediate layers on the cycling performance of Li–S batteries. Reproduced with permission [321]. Copyright 2022, Elsevier. (i) Schematic diagram of the work function difference between Co and MoN and the interface charge transfer. (j) Finite element simulation comparison of the finite electrode potential distribution of Co/MoN@PP and the original PP separator with the lithium anode. (k) Long‐term cycling performance of Li–S batteries using different separators at 1 C rate. Reproduced with permission [322]. Copyright 2024, Wiley‐VCH.

The development of MoN‐based separator materials follows a well‐defined technological trajectory, advancing from fundamental functional validation to structural engineering optimization, and further toward dynamic interfacial understanding and electronically regulated catalytic systems. The intrinsic advantage of MoN lies in its ability to simultaneously integrate high electrical conductivity, strong polar adsorption, and tunable catalytic activity, thereby enabling effective control over polysulfide adsorption and conversion while offering a promising strategy for stabilizing lithium metal anodes. These characteristics position MoN as a highly promising material platform for the realization of practical, high‐performance Li–S batteries.

#### 4.2.2 PRI‐Guided Evaluation of Molybdenum Nitride‐Based Inorganic Separator Systems: From Mechanistic Advantage to Practical Limitation

To evaluate MoN‐based separator systems, representative architectures are summarized in Table 10 and analyzed within the PRI framework, enabling unified comparison of their practical relevance under consistent constraints.

**TABLE 10:** Representative molybdenum nitride‐based inorganic separator systems for Li–S batteries, summarizing key electrochemical parameters and practical operating conditions, including sulfur loading, electrolyte‐to‐sulfur ratio, areal capacity, coating mass, separator thickness, and device‐level validation. All systems are further evaluated using the PRI, which integrates energy contribution, lean electrolyte compatibility, structural penalty, and device validation into a unified constraint‐based framework (Table S13). This table highlights representative architectures that illustrate the evolution from adsorption‐dominated designs to defect‐engineered and heterostructure‐based systems.

System	Mechanism	Sulfur loading (mg cm^−2^)	E/S (μ L mg^−1^)	Areal capacity (mAh cm^−2^)	Testing condition (rate, cycles)	Coating mass (mg cm^−2^)	Thickness (μ m)	Pouch cell	Refs.
MoN‐G/PP	Adsorption‐catalysis synergistic separator	7.0	20	6.02	0.1C, 40 cycles	/	12	/	[318]
CCC@MoN	Adsorption‐catalysis synergistic separator	5.0	20	4.2	0.1C, 100cycles	/	/	/	[319]
MoN@CNFs/PP	Adsorption‐catalysis synergistic separator	2.3	20	1.92	0.5C, 150 cycles	/	8.4	/	[320]
Co/MoN@PP	Adsorption‐catalysis synergistic separator	4.92	10	4.61	0.1C, 60 cycles	0.35	6.95	/	[322]
SA‐Co/NGM@PP	Heterojunction/defect‐engineered separator	6.5	6.8	4.73	0.2C, 200 cycles	0.156	5	/	[325]
M‐TiN/MoN@PP	Heterojunction/defect‐engineered separator	5.12	10	3.66	0.1C, 80 cycles	/	6.55	/	[327]

The PRI‐based evaluation of MoN‐ and Co‐Mo‐N‐based separator systems reveals a clear transition from structure‐constrained architectures toward near‐cornerstone performance, depending strongly on interface design and nitrogen‐mediated electronic regulation. Compared with previously analyzed oxide‐ and sulfide‐based systems, MoN‐based architectures exhibit significantly improved energy contribution (ECS), indicating enhanced catalytic and conductive synergy. However, their practical relevance is still strongly influenced by structural penalties (SPS), particularly in carbon‐supported composites with high inactive mass or thick separator configurations.

Notably, SA‐Co/NGM@PP demonstrates the most balanced performance among all evaluated systems, achieving simultaneously high ECS and moderate SPS, thus representing a near‐cornerstone candidate under the PRI framework. In contrast, CNF‐ and carbon‐heavy architectures suffer from severe structural penalties, resulting in strongly compromise‐dominated behavior. Overall, MoN‐based systems occupy an intermediate position between catalytic enhancement and structural limitation, with a subset of atomically dispersed or heterostructure‐optimized systems approaching cornerstone‐like behavior.

### 4.3 Vanadium Nitride

#### 4.3.1 Mechanistic Evolution of Vanadium Nitride‐Based Inorganic Separator Systems for Polysulfide Regulation

Among separator modification materials for Li–S batteries, vanadium nitride (VN), a typical metallic transition‐metal nitride, has attracted increasing attention owing to its exceptional electrical conductivity, which can reach about 10^6^ S m^−1^ and approaches that of metals [326]. This intrinsic property is significantly superior to that of many metal oxides and sulfides [328]. As a result, VN can function not only as an efficient polysulfide “capture layer” on the separator side but also as a highly conductive electron‐transport channel and catalytic reaction interface [329]. Research on VN‐based separator modification has clearly demonstrated a technological evolution from single‐phase functional layers to carbon‐composite optimization, and further toward nanoscale regulation and heterostructure‐based interfacial engineering.

Early studies mainly introduced VN as a single‐phase inorganic functional layer into separator systems to verify its intrinsic advantages. In 2022, Jing et al. [330] reported a separator modified with porous VN nanoribbons. The porous VN nanoribbons were synthesized via wet‐chemical methods followed by ammonia annealing and subsequently deposited onto a Celgard separator through vacuum filtration (Figure 16a). The resulting modification layer effectively suppressed polysulfide shuttling and accelerated the nucleation and decomposition kinetics of Li_2_S, thereby catalyzing the bidirectional conversion between LiPSs and Li_2_S while significantly lowering the reaction activation energy (Figure 16b). The corresponding battery exhibited an extremely low‐capacity decay rate during cycling at 0.5 C (Figure 16c), providing the first systematic demonstration of the intrinsic advantages of VN in separator modification. Based on the verification of the intrinsic performance of single‐phase VN, researchers recognized that its structural stability and interfacial compatibility could still be further improved. Consequently, integrating VN with carbon materials to construct composite systems became an important research direction. For example, a sandwich‐structured separator fabricated via magnetron sputtering incorporated a VN layer on the cathode side to adsorb and catalyze polysulfide conversion, while a hexagonal boron nitride (hBN) layer on the anode side improved thermal management and suppressed lithium dendrite growth [331]. This design highlights the modular integration capability of VN within multilayer separator engineering. To further enhance the interfacial efficiency of VN, research efforts have gradually shifted toward nanoscale regulation. Ma et al. [287] proposed anchoring VN quantum dots onto nitrogen‐doped carbon nanosheets (VN@NC). The ultrafine VN quantum dots (about 2 nm) dramatically increase active‐site utilization (Figure 16d,e), significantly improving both cycling stability and rate capability (Figure 16f). This strategy represents a transition from macroscopic composite design to the precise construction of nanoscale catalytic centers. Currently, heterostructure engineering and electronic‐structure regulation have become the frontier directions in VN‐based separator research. By constructing heterointerfaces between VN and other functional phases, interfacial electric fields and electronic‐reconstruction effects can be utilized to enhance both polysulfide adsorption and catalytic conversion kinetics. In a VN/Mo_2_C heterostructure separator reported in 2024 [332], VN nanocrystals were highly dispersed on a carbon substrate and formed synergistic catalytic centers with Mo_2_C at the interface (Figure 16g). Experimental results showed that the VN/Mo_2_C@CNS battery exhibited the lowest shuttle current (1.5 × 10^−2^ mA cm^−2^), indicating that VN, with its high conductivity and low ion‐diffusion barrier, cooperatively works with Mo_2_C to anchor and catalyze polysulfide conversion (Figure 16h). Building on this concept, Guo et al. [333] further constructed a VN‐MoC heterostructure, where precise regulation of the interfacial electronic structure shifts the d‐band center upward, simultaneously optimizing the adsorption strength toward LiPSs and catalytic activity (Figure 16i,j).

**FIGURE 16:**
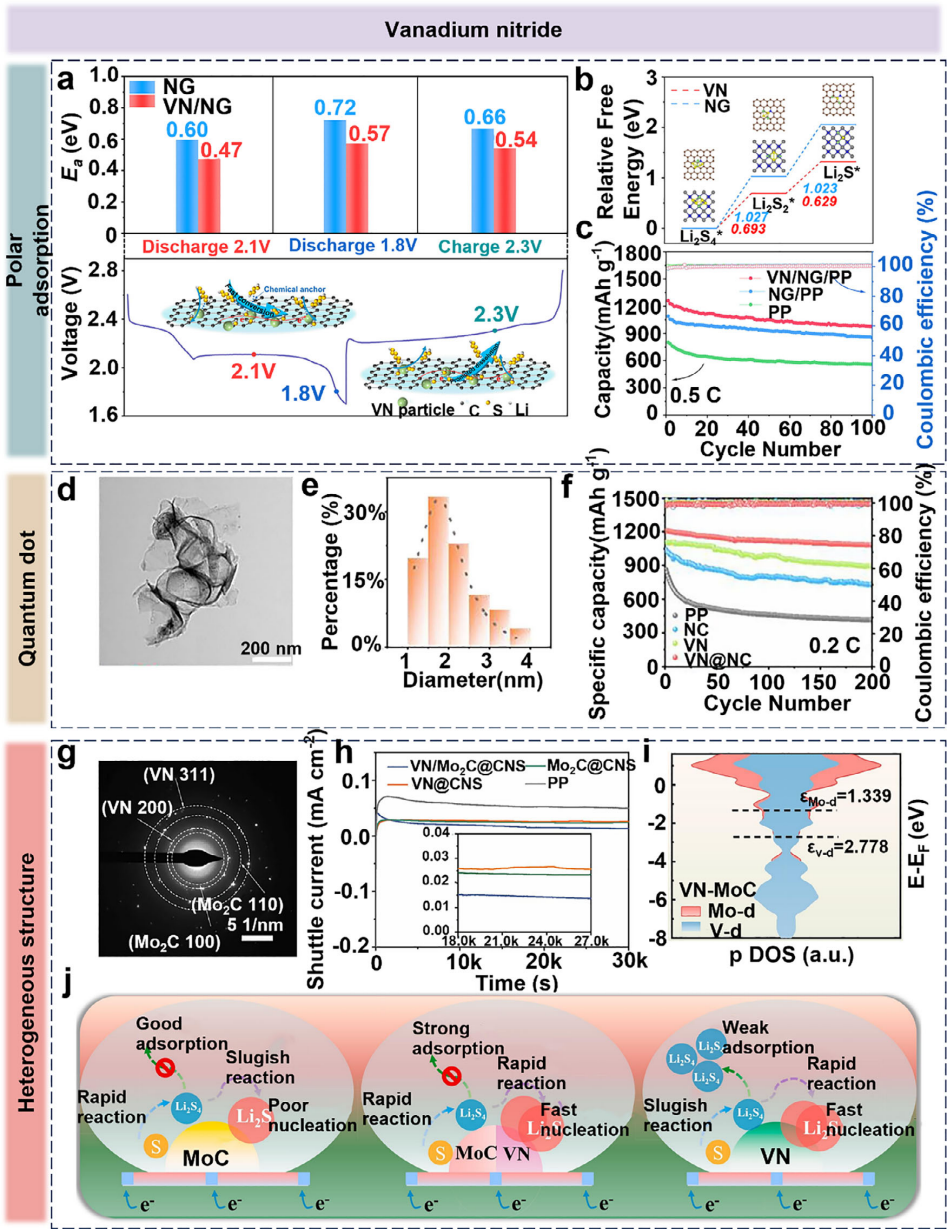
Strategy of vanadium nitride modified separator. (a) Comparison of activation energy between VN/NG and NG modified separators, showing that VN/NG significantly reduces the reaction energy barrier of polysulfide reduction and Li_2_S oxidation. (b) DFT calculations indicate that the Gibbs free energy change of the Li_2_S_4_→Li_2_S reaction on the VN surface is lower than that on NG, theoretically confirming its catalytic advantage. (c) Cycling performance comparison of different separators at 0.5 C, with the VN/NG modified separator demonstrating the highest discharge capacity and the best capacity retention rate. Reproduced with permission [330]. Copyright 2023, Elsevier. (d) TEM images show that VN quantum dots are uniformly anchored on N‐doped carbon nanosheets. (e) Statistical analysis of quantum dot size distribution indicates an average diameter of 1.5–2 nm, forming a highly catalytically active interface. (f) Long cycle performance of VN@NC‐based Li–S batteries. Reproduced with permission [287]. Copyright 2023, Elsevier. (g) SAED patterns present clear polycrystalline diffraction rings, respectively attributed to the (200) and (311) planes of VN and the (100) and (110) planes of Mo_2_C. (h) Shuttle current test in potentiostatic mode, with the battery using the VN/Mo_2_C@CNS modified separator showing the lowest shuttle current (1.5 × 10^−2^ mA cm^−2^). Reproduced with permission [332]. Copyright 2024, Elsevier. (i) Electronic state density (DOS) analysis of VN‐MoC@PC heterostructure. (j) Schematic illustration of the cooperative catalytic mechanism of VN‐MoC heterostructure for LiPSs conversion. Reproduced with permission [334]. Copyright 2025, Elsevier.

Notably, with the continued development of heterostructure engineering, the role of VN‐based separator systems has gradually expanded from regulating polysulfide behavior at the cathode side to simultaneously stabilizing the lithium‐metal anode interface. Several studies have demonstrated that VN‐based heterostructure separators often exhibit lithophilic characteristics, which help regulate lithium‐ion flux distribution during cycling and reduce local current density, thereby suppressing lithium dendrite growth to a certain extent [327, 335]. This separator design concept—simultaneously regulating sulfur cathode reaction kinetics and lithium deposition behavior—marks the evolution of VN‐based separator engineering toward bifunctional separator systems.

Research on VN‐based separator modification has evolved from the validation of single‐phase functional layers to carbon‐composite optimization and further toward nanoscale regulation and heterostructure‐mediated interfacial engineering [336, 337]. The key advantage of VN lies in its combination of metallic‐level conductivity, strong polar adsorption capability, and tunable catalytic activity, which collectively enable full‐process regulation of LiPSs through strong adsorption, rapid conversion, and efficient reutilization. Moreover, the ability of VN‐based heterostructures to stabilize the lithium‐metal anode interface further highlights their potential for integrated electrode regulation. These advances provide important design principles for separator engineering toward high‐energy‐density and long‐lifetime Li–S batteries.

#### 4.3.2 PRI‐Guided Evaluation of Vanadium Nitride‐Based Inorganic Separator Systems: From Mechanistic Advantage to Practical Limitation

Representative VN‐based separator systems are summarized in Table 11 and evaluated within the PRI framework. This enables a unified comparison of energy contribution (ECS), electrolyte utilization (LECS), structural penalty (SPS), and device‐level validation (DVS) under consistent evaluation criteria, providing a quantitative basis for distinguishing cornerstone, transition, and compromise behaviors across different structural designs (Table S14).

**TABLE 11:** Representative vanadium nitride‐based inorganic separator systems for Li–S batteries, summarizing key electrochemical parameters and practical operating conditions, including sulfur loading, electrolyte‐to‐sulfur ratio, areal capacity, coating mass, separator thickness, and device‐level validation. All systems are further evaluated using the PRI, which integrates energy contribution, lean electrolyte compatibility, structural penalty, and device validation into a unified constraint‐based framework. This table highlights representative architectures that illustrate the evolution from adsorption‐dominated designs to defect‐engineered and heterostructure‐based systems.

System	Mechanism	Sulfur loading (mg cm^−2^)	E/S (μ L mg^−1^)	Areal capacity (mAh cm^−2^)	Testing condition (rate, cycles)	Coating mass (mg cm^−2^)	Thickness (μ m)	Pouch cell	Refs.
VN quantum dots@N‐doped carbon nanosheets (VN@NC)	Adsorption‐catalysis synergistic	5.4	4.6	4.02	0.1C, 50 cycles	/	3.3	(S:5.4 mg cm^−2^, 4.02 mAh cm^−3^, 0.1C, 50 cycles)	[287]
VN/NG (nanoparticles on N‐doped graphene)	Adsorption‐catalysis synergistic	3.2	10	2.56	0.2C, 200 cycles	/	/	/	[330]
VN‐MoC@PC	Heterojunction/defect‐engineered separator	4.8	10	5.13	0.2C, 100 cycles	/	/	/	[334]
VN@NC (nanocrystals in porous carbon nanosheets	Adsorption‐catalysis synergistic separator	4.5	8	3.78	0.1C, 70 cycles	0.4	17	/	[333]
VN (porous nanobelts)	Adsorption‐dominated	3.8	10.5	2.7	0.2C, 100 cycles	0.5	25	/	[338]
VN (porous, from MAX phase)	Adsorption‐catalysis synergistic separator	1.0	40	0.69	1C, 400 cycles	0.5	15	/	[338]
VN (Multi‐Branched, MB‐VN)	Adsorption‐catalysis synergistic	6.0	6	3.77 (0.2C, 50 cycles)	0.5C, 400 cycles	/		/	[328]
VN/rGO	Adsorption‐catalysis synergistic separator	1.9	20	1.58	0.5C, 200 cycles	/	30	/	[339]
FeN_4_‐O‐NC‐VN	Heterojunction/defect‐engineered	1.1	20	0.68	2C, 500 cycles	/	/	/	[35]
VN/Mo_2_C@CNS	Heterojunction/defect‐engineered separator	5.6	10	4.2	0.2C, 100 cycles	/	/	/	[332]
PEG‐VN (PEG‐VN@PP)	Adsorption‐catalysis synergistic	1.2	15	0.94	1C, 700 cycles	0.1	4	/	[340]
VN‐V_2_O_5_@rGO	Band structure/built‐in electric field modulated separator	4.3	10	4.8	0.2C, 100 cycles	/	13	/	[327]

The PRI‐based evaluation of VN‐based separator systems reveals a moderately diversified performance landscape, where the practical relevance is strongly determined by the balance between electrochemical contribution, electrolyte utilization, and structural penalty. Benefiting from the intrinsic metallic conductivity and favorable catalytic activity of VN, these systems generally exhibit competitive ECS values; however, their overall PRI performance remains highly dependent on structural integration and device‐level validation. Carbon‐supported VN architectures, particularly VN@NC, demonstrate promising practical potential by simultaneously achieving high energy contribution, improved electrolyte compatibility, and reduced structural penalties. The enhanced PRI value of VN@NC is further supported by device‐level validation, indicating that rational coupling between conductive carbon frameworks and VN active sites can effectively translate intrinsic catalytic advantages into practical separator functionality. Similarly, VN‐based hybrid systems such as VN‐V_2_O_5_@rGO and VN@NC‐derived architectures show improved comprehensive performance through interfacial engineering and conductive network optimization.

In contrast, VN systems with insufficient structural optimization, such as porous VN architectures or carbon‐free configurations, suffer from limited electrolyte efficiency and/or increased structural penalties, resulting in reduced PRI values despite maintaining moderate electrochemical contributions. Therefore, VN‐based separators can be classified as a partially optimized performance regime under the PRI framework, where carefully engineered carbon‐supported heterostructures approach practical relevance, while excessive structural complexity remains a key limitation.

### 4.4 Other Nitrides

#### 4.4.1 Mechanistic Evolution of Other Nitrides Modified Inorganic Separator Systems for Polysulfide Regulation

Within the separator modification material systems for Li–S batteries, late transition‐metal nitrides‐such as iron‐based [327, 341], nickel‐based [342], and cobalt‐based nitrides [343] have been reported less frequently compared with early transition‐metal nitrides such as TiN, MoN, and VN. Nevertheless, these materials have demonstrated comparable and sometimes even more distinctive advantages. Owing to the strong affinity between metal‐nitrogen bonds and lithium LiPSs, together with the typically higher surface reactivity of late transition‐metal nitrides, these materials not only effectively anchor LiPSs but also promote their redox conversion and accelerate Li_2_S nucleation and decomposition [344, 345]. Consequently, they drive separator functionality from adsorption‐dominated regulation toward adsorption‐catalysis synergistic control. The research trajectory of these materials clearly shows a progressive evolution from basic functional application to defect engineering, heterostructure design, and ultimately system‐level integration.

At the fundamental application stage, constructing nitride‐based functional coatings on separator surfaces through simple deposition strategies represents a basic approach that combines physical confinement with chemical adsorption. For example, Pu et al. [346] coated Co_4_N nanocrystals onto the surface of a Celgard separator to form a functional layer approximately 10 μ m thick. Benefiting from the high electrical conductivity of Co_4_N and its strong bonding interaction with LiPSs, the modified separator significantly improved polysulfide anchoring and conversion kinetics. This work provided an early demonstration of the feasibility of employing late transition‐metal nitrides in separator modification. To further enhance intrinsic catalytic activity, researchers subsequently employed defect engineering to precisely regulate the electronic structure of nitrides, thereby simultaneously strengthening adsorption and catalytic functions. Luo et al. [347] introduced nitrogen vacancies into CoN, which enhanced chemical interactions with LiPSs and reduced the Li^+^ diffusion energy barrier. Similarly, Zhao et al. [348] selectively etched Fe atoms from Ni_3_FeN in situ, producing a vacancy‐rich Ni_3_Fe_1‐δ_N phase. The generated high‐density active vacancies significantly accelerated surface reaction kinetics (Figure 17a). Even under lean‐electrolyte conditions, the Ni_3_FeN/G battery maintained high‐capacity output (Figure 17b). Beyond optimizing individual materials, constructing nitride‐based heterostructures or composite architectures has been demonstrated as an effective strategy to achieve substantial performance enhancement through interfacial synergy. For instance, a Ni_3_N/Nb_4_N_5_ heterostructure‐modified separator improved both polysulfide adsorption and catalytic activity via interfacial charge regulation [349]. The Co‐Fe_3_N Mott–Schottky heterostructure proposed by Yi et al. [350] not only facilitates charge transfer through the built‐in electric field but also provides a stable reaction host and interface at the structural level. The resulting material exhibits excellent mechanical flexibility (Figure 17c) and remarkable thermal dimensional stability, showing almost no thermal shrinkage at 150°C (Figure 17d), which contributes to enhanced physical reliability and safety of the battery system. The built‐in electric field of this heterostructure effectively promotes charge transfer, strengthens polysulfide adsorption and catalytic conversion, and enables outstanding electrochemical stability (Figure 17e). In another example, Li et al. [351] derived a Co‐Ni_3_N heterostructure from layered double hydroxide (LDH) precursors and anchored it onto nitrogen‐doped carbon nanofibers, forming a porous conductive network with both high electrical conductivity and strong catalytic activity. Recent studies have increasingly moved toward system‐level integration and multifunctional design, aiming to construct composite functional layers that simultaneously integrate adsorption, catalysis, and electron transport. For example, the CoN@NCNT‐modified separator developed by Jia et al. [352] exploits the physical confinement effect of hollow carbon nanotubes, the chemical adsorption capability of nitrogen doping, and the catalytic activity of CoN nanoparticles to achieve a triple synergistic regulation of confinement‐adsorption‐catalysis (Figure 17f,g). Li et al. [353] further proposed introducing CoN/Co_9_S_8_@RGO and Co_9_S_8_ functional layers on the cathode and separator sides, respectively, forming a continuous catalytic interface and establishing a closed‐loop regulation system for strong adsorption and rapid conversion. Moreover, the SA‐Fe/Fe_2_N dual‐active‐site separator reported by Zhang et al. [354] employs atomically dispersed Fe single atoms in combination with Fe_2_N nanocrystals, significantly enhancing the bidirectional liquid‐solid conversion kinetics of LiPSs (Figure 17h,i). The battery maintained a capacity retention of 80.42% after 200 cycles at 0.2 C (Figure 17j), providing a promising strategy for developing high‐efficiency and long‐lifetime Li–S batteries.

**FIGURE 17:**
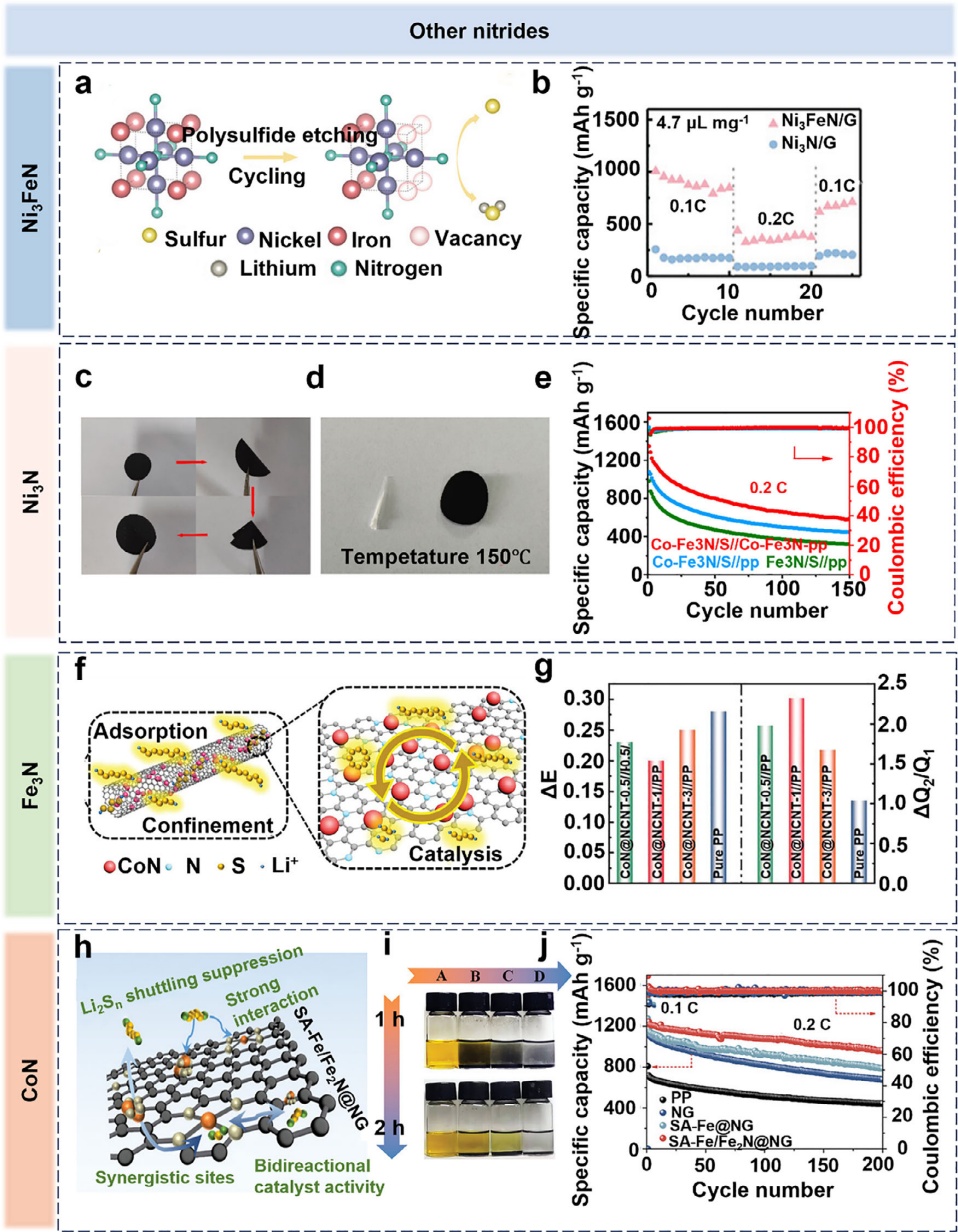
Other nitrides modified separator strategy. (a) Atomic structure model of cubic Ni_3_FeN and illustration of the polysulfide etching process toward an active Ni_3_Fe_1‐δ_ N phase. (b) Rate performance of lean‐electrolyte batteries. Reproduced with permission [327]. Copyright 2019, Wiley‐VCH. (c) Mechanical flexibility of the Co‐Fe_3_N‐coated separator. (d) Mechanical flexibility of the Co‐Fe_3_N‐coated separator. (e) Long‐term stability under high sulfur loading. Reproduced with permission [350]. Copyright 2024, Elsevier. (f) The mechanisms of confinement‐adsorption‐catalysis of LiPSs at CoN@NCNT‐1 composites. (g) Δ*E* and ΔQ2/ΔQ1 obtained from galvanostatic charge/discharge profiles of CoN@NCNT‐0.5//PP, CoN@NCNT‐1//PP, CoN@NCNT‐3//PP, and pure PP separator. Reproduced with permission [352]. Copyright 2024, Wiley‐VCH. (h) Schematic diagram of the catalytic mechanism of SA‐Fe/Fe_2_N@NG. (i) Visual comparison of polysulfide adsorption performance. (j) Long‐term cycling performance of batteries using different separators at 0.2 C. Reproduced with permission [354]. Copyright 2021, Wiley‐VCH.

In conclusion, separator modification strategies based on late transition‐metal nitrides have evolved from single nitride functional layers to diversified approaches involving defect engineering, heterostructure design, and system‐level synergistic regulation. The core principle lies in tuning the electronic structure of nitride materials to achieve efficient polysulfide adsorption and rapid catalytic conversion. Future research should further focus on the long‐term stability of active sites, deeper understanding of heterointerface synergistic mechanisms, and adaptability under practical operating conditions, thereby advancing the development of high‐energy‐density Li–S batteries toward real‐world applications [355].

#### 4.4.2 PRI‐Guided Evaluation of Other Nitrides Modified Inorganic Separator Systems: From Mechanistic Advantage to Practical Limitation

Transition‐metal nitride‐based separator systems are summarized in Table 12 and evaluated within the PRI framework. This enables a unified comparison of energy contribution (ECS), electrolyte utilization (LECS), structural penalty (SPS), and device‐level validation (DVS), providing a consistent basis for assessing the balance between intrinsic catalytic activity and structural design efficiency (Table S15).

**TABLE 12:** Representative other nitrides modified separator systems for Li–S batteries, summarizing key electrochemical parameters and practical operating conditions, including sulfur loading, electrolyte‐to‐sulfur ratio, areal capacity, coating mass, separator thickness, and device‐level validation. All systems are further evaluated using the PRI, which integrates energy contribution, lean electrolyte compatibility, structural penalty, and device validation into a unified constraint‐based framework. This table highlights representative architectures that illustrate the evolution from adsorption‐dominated designs to defect‐engineered and heterostructure‐based systems.

System	Mechanism	Sulfur loading (mg cm^−2^)	E/S (μ L mg^−1^)	Areal capacity (mAh cm^−2^)	Testing condition (rate, cycles)	Coating mass (mg cm^−2^)	Thickness (μ m)	Pouch cell	Refs.
Co_4_N‐Celgard	Adsorption‐catalysis synergistic separator	3.2	15	2.5	1C, first cycle	/	10	/	[346]
CNT‐CoN‐VN	Heterojunction/defect‐engineered separator	4.5	20	4.5	0.2C, 50 cycles	0.5	10	/	[347]
Li‐CON@GN/Celgard	Adsorption‐catalysis synergistic separator	1.2	20	0.9	0.5C, 200 cycles	0.3	/	/	[356]
CoN‐CNT/HPC	Adsorption‐catalysis synergistic separator	5.5	/	3.1	0.5C, 100 cycles	0.3	30	/	[357]
Co_2_P/Co/C	Adsorption‐catalysis synergistic separator	2.68	30	2.1	0.5C, 100 cycles	/	16	/	[358]
SNP‐PC@MPC@CoSe	Adsorption‐catalysis synergistic separator	5.65	4	5.54	0.1C, first cycle	0.28	15.5	/	[359]
CoN@NCNT	Adsorption‐catalysis synergistic separator	1.2	20	0.88	0.2C, 200 cycles	/	14	/	[352]
Co‐N‐C@CeO_2_‐C	Heterojunction/defect‐engineered separator	5.0	/	3.5	0.1C, 120 cycles	/	9	/	[360]
CoN‐Mo_2_N	Heterojunction/defect‐engineered separator	1.7	/	1.47	0.2C, 200 cycles	1.3	/	/	[361]
Ni_3_N/Nb_4_N_5_	Heterojunction/defect‐engineered	1.5	20	1.19	0.1C, 150 cycles	1.9	23.65	/	[362]
N_i3_FeN	Heterojunction/defect‐engineered	4.8	4.7	4.2	1C, first cycle	0.3	/	/	[363]
Co‐Ni_3_N‐CNFs	Heterojunction/defect‐engineered	1.2	/	0.87	2C, 1000 cycles	0.0008	/	/	[351]
Co‐Fe_3_N/PP	Band structure/built‐in electric field modulated separator	3.1	28	1.26	0.1C, 300 cycles	/	3.5	/	[350]
Fe_2_N/N‐rGO	Adsorption‐catalysis synergistic separator	8	5.5	4.04	0.5C, 250 cycles	0.25	10	/	[355]
Fe_3_C/Fe_3_N@NCNT	Adsorption‐catalysis synergistic separator	13.12	/	2.20	0.13C, 50 cycles	/	/	/	[364]
SA‐Fe/Fe_3_N@NG	Adsorption‐catalysis synergistic separator	5	/	5.02	0.1C, 100 cycles	/	5	/	[354]

The PRI‐based evaluation of transition‐metal nitride‐based separator systems reveals a distinctly heterogeneous performance landscape governed by a strong competition between catalytic activity and structural penalty. Compared with oxide‐ and sulfide‐based systems, nitrides generally exhibit higher intrinsic electronic conductivity and improved redox kinetics, leading to enhanced energy contribution (ECS) in several architectures. However, the analysis clearly shows that high catalytic activity alone does not guarantee high practical relevance. Systems with excessive structural complexity, thick coatings, or insufficient electrolyte optimization suffer from significant SPS penalties, which substantially suppress their PRI values. Notably, only a few optimized architectures, such as SNP‐PC@MPC@CoSe and Ni_3_FeN‐based systems, achieve transition or near‐cornerstone behavior, indicating that balanced structural design is essential to translate intrinsic nitride advantages into practical performance. In contrast, most multicomponent nitride composites remain compromise‐dominated due to structural overengineering and incomplete electrolyte utilization. Overall, nitride‐based systems occupy a “high‐potential but structure‐sensitive” regime, where practical applicability is strongly dependent on the balance between catalytic enhancement and structural efficiency.

## 5 MXene‐Based 2D Transition Metal Carbides/Nitrides Materials

### 5.1 Mechanistic Evolution of MXene‐Modified Inorganic Separator Systems for Polysulfide Regulation

Beyond conventional transition‐metal compounds, MXenes have emerged as a promising class of 2D materials for separator engineering in Li–S batteries [365]. Their high electrical conductivity, layered structure, and abundant surface functional groups enable efficient polysulfide adsorption while facilitating rapid ion/electron transport [366]. Moreover, the tunable interlayer spacing and surface chemistry make MXenes ideal platforms for constructing multifunctional catalytic interfaces [367]. Representative MXene‐based separator systems reported in recent studies are summarized in Table S16.

### 5.2 Surface Chemical Regulation of MXene and Reaction Pathways With LiPSs

Early studies on MXene‐modified separators mainly relied on the dense barrier formed by stacked 2D MXene nanosheets. However, such restacking inevitably introduces a trade‐off, as it may hinder ion transport [368]. To address this issue, subsequent research gradually shifted toward interlayer‐spacing regulation and interfacial structure engineering. For example, Zhang et al. in 2025 [369] systematically compared pristine MXene (M‐MXene), LiCl‐intercalated MXene (L‐MXene), and TMAOH‐intercalated MXene (T‐MXene), demonstrating that intercalation engineering can effectively expand the interlayer spacing while exposing more surface ‐O/‐OH groups. This structural regulation strengthens polysulfide blocking while maintaining efficient Li^+^ transport, providing important guidance for the rational design of MXene‐based separators.

On this basis, further studies advanced toward ultrathin architectures and precise sieving mechanisms. Liao et al. [370] reported an ultrathin MXene separator modification layer functionalized with tannic acid, with a thickness of only 530 nm. The key feature of this system is the construction of stable sub‐nanometer interlayer channels induced by organic molecules. On the one hand, this structure enables effective physical sieving of larger polysulfide anions; on the other hand, it promotes early desolvation of solvated Li^+^ ions within the confined channels, thereby accelerating Li^+^ transport and sulfur‐species reaction kinetics (Figure 18a). Further characterization showed that the sub‐nanometer interlayer spacing remains highly stable even in the electrolyte environment, providing a reliable structural basis for long‐term physical sieving during battery operation (Figure 18b). Correspondingly, ion‐transport measurements revealed that such a structure does not sacrifice Li^+^ mobility, but instead exhibits a higher Li^+^ transference number, confirming that effective physical blocking can be achieved without compromising ionic transport (Figure 18c). Beyond interlayer‐spacing sieving, the concept of charge‐regulated 2D nanofluidic channels has further expanded MXene‐based separator design. Luo et al. [371] showed that alternating stacking of negatively charged porous silicon nanosheets with MXene can construct angstrom‐scale 2D ionic channel networks on the separator side (Figure 18d). In this architecture, spatial confinement and interfacial electrostatic interactions work together to selectively accelerate Li^+^ transport under an electric field, while polysulfide anions are suppressed by both steric constraints and electrostatic repulsion. Rather than directly visualizing the nanofluidic channels, this work validated their effectiveness through rational structural design and the pronounced improvement in selective ion‐transport behavior (Figure 18e). Li^+^ transference measurements further confirmed significantly enhanced Li^+^‐selective transport, functionally demonstrating the validity of the 2D nanofluidic channel design (Figure 18f). In addition to 2D layered structures, 3D porous or fibrous networks have also been shown to improve the overall performance of MXene‐based separators. Zhao et al. [372] introduced Ti_3_C_2_ MXene uniformly into a carbon nanofiber network to construct a continuous 3D interconnected framework on the separator side. Different from local pore confinement or interlayer sieving, this strategy regulates Li^+^ migration pathways from the perspective of the entire separator. By controlling the distribution of MXene within the fibrous network, a 3D mass‐transport framework that is continuous but not overly congested can be established, thereby homogenizing Li^+^ flux. As illustrated in the structural schematic, an appropriate MXene loading forms an interconnected network that prevents localized ion accumulation, whereas excessively low or high MXene contents lead to insufficient flux regulation or blocked transport pathways, respectively (Figure 18g). These results indicate that the 3D framework is not merely a passive support, but directly participates in ion‐transport regulation. At the functional level, the regulation of Li^+^ transport by the 3D interconnected MXene framework is ultimately reflected in improved lithium deposition behavior. Both in situ and ex situ characterizations showed that lithium deposition becomes smoother and more uniform under the influence of the 3D MXene network, with reduced localized protrusions and void formation. AFM images directly reveal the uniform deposition morphology induced by this structure, while small‐angle neutron scattering (SANS) provides quantitative evidence from the structural scale perspective. The significantly reduced characteristic size (Rg) indicates that lithium deposition proceeds on a more homogeneous spatial scale (Figure 18h,i). These results demonstrate that, unlike 2D layered or nanofluidic‐channel structures that mainly emphasize size sieving or selective transport, the 3D interconnected MXene framework can fundamentally alter interfacial deposition behavior by regulating overall Li^+^ flux distribution. Similarly, the MXene‐TMO/PVDF electrospun interlayer reported by Busra et al. in 2025 [373] and the earlier MXene/SiO_2_ aerogel separator design [374] both employ 3D frameworks to suppress MXene restacking while improving electrolyte wettability and ion diffusion.

**FIGURE 18:**
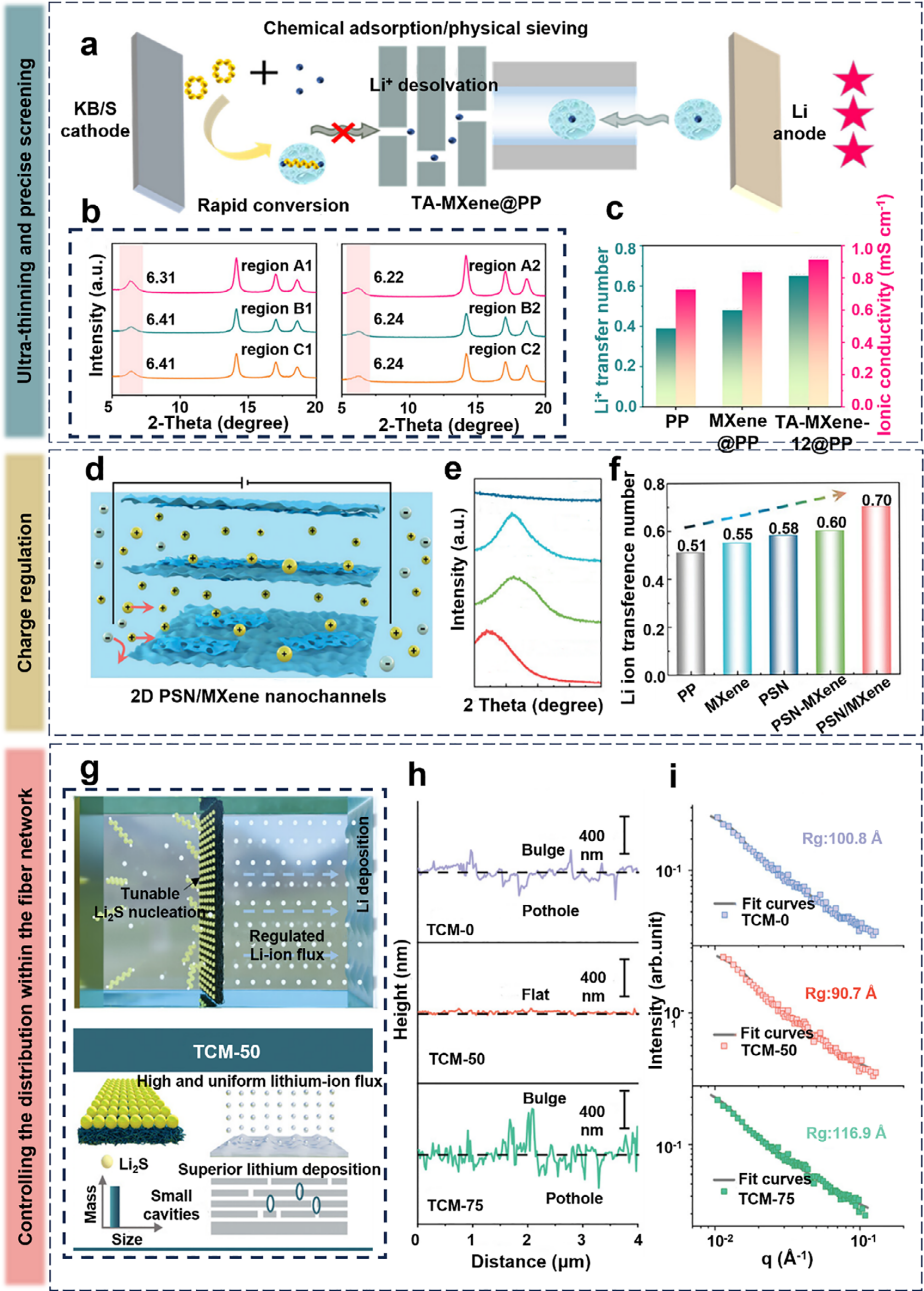
(a) Schematic diagram of the working mechanisms of TA‐MXene@PP separators in Li–S batteries. b) XRD patterns of the TA‐MXene‐12@PP separator (b) before and (c) after soaking in DME/DOL for 12 h (the three XRD patterns in each figure come from three different regions). (c) Li^+^ transfer number and ionic conductivity of PP, MXene@PP, and TA‐MXene‐12@PP separators. Reproduced with permission [370]. Copyright 2025, Wiley‐VCH. (d) Schematic depiction of the negatively charged nanochannels in PSN/MXene membrane facilitating the transport of cation ions and rejecting anions. (e) XRD patterns of PSN, MXene, PSN/MXene, and PSN‐MXene, respectively. (f) t_Li+_ of Li//Li symmetric batteries with different modified separators. Reproduced with permission [371]. Copyright 2025, Wiley‐VCH. (g) (Above) Schematic showing the vital merits of TCM‐x on concurrently regulating both sulfur cathode and lithium anode. (Below) Schematic showing the importance of TCM content adjustment for TCM‐x to lithium deposition. (h) Surface depth profiles, (i) SANS fitting curves of scattering intensity signals of cycled Li anodes in Li//Li symmetric batteries with TCM‐0, TCM‐50, and TCM‐75. Reproduced with permission [284]. Copyright 2025, Wiley‐VCH.

These studies highlight structural design itself as the origin of functionality, showing that regulation of interlayer spacing, pore size, stacking configuration, and interfacial morphology can construct effective physical barriers against LiPSs while maintaining rapid Li^+^ transport. Owing to their intuitive design principles and controllable architectures, such strategies provide an important foundation for the development of MXene‐based functional separators.

### 5.3 The Layered Structure Characteristics of MXene and Its Regulatory Effect on the Conversion of LiPSs

Although structural engineering can suppress polysulfide diffusion to some extent, strategies relying only on physical blocking often struggle to maintain stable performance under high sulfur loading or long‐term cycling [375, 376]. As a result, researchers have gradually introduced chemical adsorption mechanisms by regulating the surface chemistry and defect structures of MXene to strengthen its anchoring capability toward LiPSs [377]. Among these strategies, defect engineering is one of the most representative approaches.

Li et al. [378] constructed an Ag‐ZnSe@MXene heterostructure through aliovalent Ag^+^ doping, in which ZnSe nanodots were anchored on MXene (Figure 19a). This strategy induces selenium vacancies in the ZnSe lattice. Electronic‐structure analysis and defect characterization show that Ag^+^ doping significantly modifies the local electronic environment of ZnSe (Figure 19b), thereby creating more polar adsorption sites for LiPSs. Benefiting from this defect‐induced interfacial regulation, the material exhibits markedly improved rate performance compared with the control samples (Figure 19c), indicating enhanced polysulfide conversion kinetics. A similar concept was reported by Zhen et al. in 2024 in the Vo‐LDHs/MXene heterostructure [379]. By introducing oxygen vacancies into layered double hydroxides (LDHs), a defect‐rich polar interface was constructed on the material surface (Figure 19d), which significantly strengthens the chemical adsorption of LiPSs. Further electrochemical and theoretical analyses revealed that vacancy‐induced electronic‐structure modulation not only increases polysulfide binding strength, but also accelerates the nucleation and decomposition kinetics of Li_2_S (Figure 19e), thereby effectively mitigating the shuttle effect. Heteroatom doping represents another important chemical regulation strategy. In addition to defect engineering, heteroatom incorporation can effectively enhance the interfacial regulation capability of MXene. For example, the N, S co‐doped wrinkled MXene (NSMX) reported by Feng et al. in 2024 [380] exhibits a pronounced wrinkled nanosheet morphology according to SEM observations (Figure 19f), which helps enlarge the specific surface area and expose more accessible interfaces. Elemental mapping further confirms the uniform distribution of N and S within the MXene nanosheets (Figure 19g), demonstrating successful dual‐heteroatom doping. At the separator level, ionic conductivity measurements show that the NSMX‐modified separator exhibits higher ionic conductivity in the temperature range of 30°C–60°C (Figure 19h). This result indicates that the combined effects of wrinkled morphology and heteroatom doping improve Li^+^ transport and interfacial ion‐migration kinetics, thus providing a more favorable mass‐transport environment for rapid polysulfide conversion. Similarly, iodine‐doped MXene [381] and Nb‐doped partially oxidized MXene [382] also strengthen polysulfide anchoring by modulating the intrinsic electronic structure of MXene. Surface functional‐group engineering also plays an important role in regulating MXene‐polysulfide interactions. In the O‐Ti_3_C_2_@CNF interlayer reported by He et al. in 2022 [383], a surface dominated by oxygen terminations was constructed through stepwise oxidation regulation. Morphological comparison shows that the pristine CNF substrate exhibits a typical porous fibrous structure (Figure 19i), whereas after introducing oxygen‐terminated Ti_3_C_2_ MXene, nanosheets are uniformly anchored on the carbon nanofiber surface to form a continuous 3D conductive network (Figure 19j). This structure provides a robust framework for constructing a polar interfacial environment. Wettability measurements further show that the O‐Ti_3_C_2_@CNF surface exhibits significantly enhanced affinity toward the electrolyte, with electrolyte droplets rapidly spreading within a very short time (Figure 19k), indicating that oxygen termination groups effectively improve interfacial polarity and electrolyte wettability. Such a highly wettable polar interface facilitates the retention and regulated transport of LiPSs within the interlayer, thereby suppressing shuttle behavior and stabilizing sulfur redox reactions. In addition, a fluorine‐free MXene‐Ti‐N coordination system [384] has also been reported to enhance adsorption and structural stability through coordination chemistry regulation.

**FIGURE 19:**
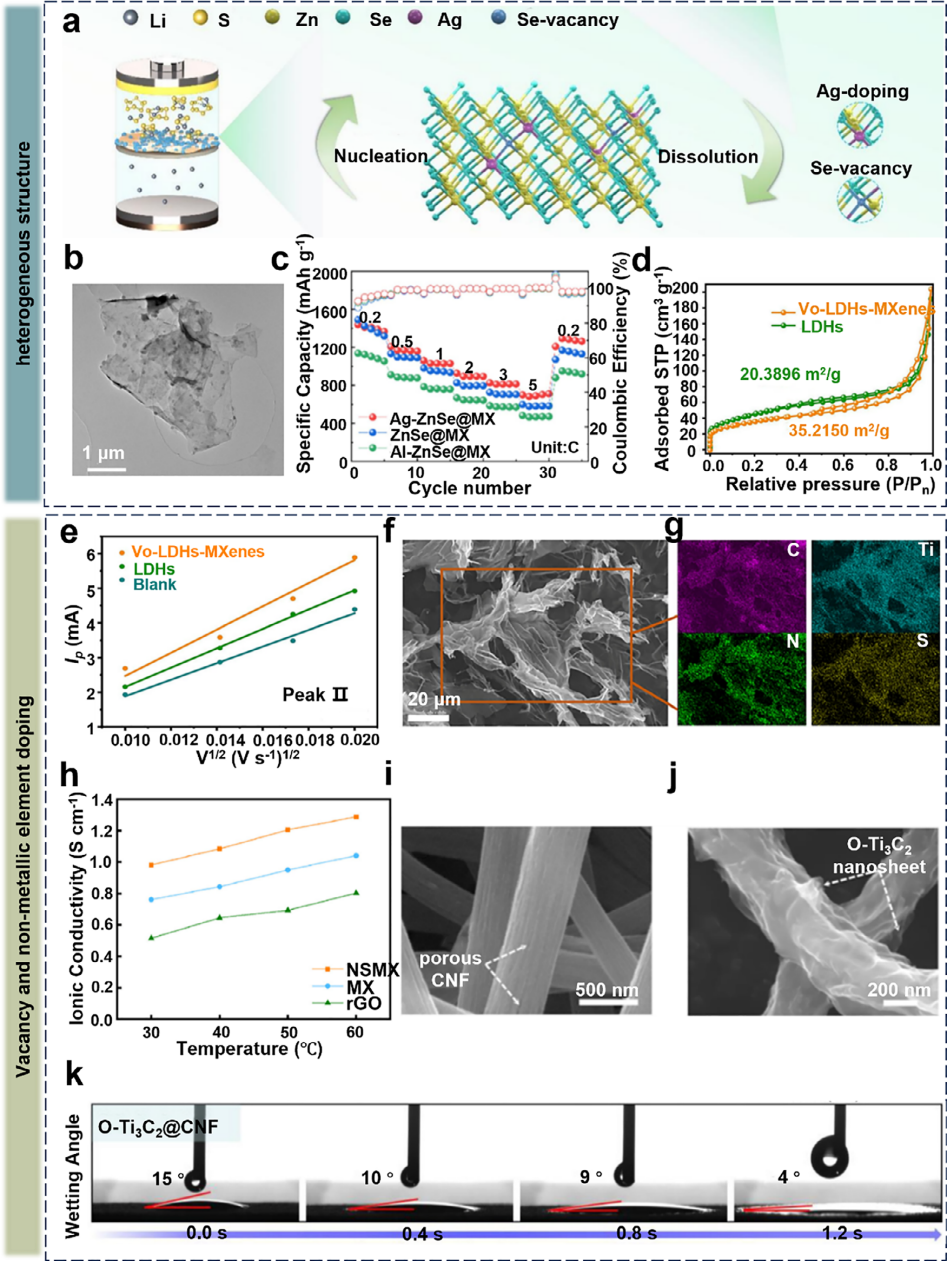
(a) Schematic illustration of the role of Ag‐ZnSe@MX catalyst. (b) TEM images of Ag‐ZnSe@MX. Reproduced with permission [378]. Copyright 2026, Elsevier. (c)Rate performance between Ag‐ZnSe@MX and other catalysts. (d) adsorption–desorption isotherms of Vo‐LDHs‐MXenes and LDHs. (e) Plots of Peak II in CV curves versus the square root of scan rates. Reproduced with permission [379]. Copyright 2024, Elsevier. (f) SEM images of NSMX and (g) EDS mapping. (h) Ion conductivity of NSMX, MX, and rGO‐modified separators at 30°C–60°C. Reproduced with permission [380]. Copyright 2024, Elsevier. SEM images of (i) porous CNF and (j) O‐Ti_3_C_2_@CNF. (k) Contact angle images of O‐Ti_3_C_2_@CNF. Reproduced with permission [383]. Copyright 2022, Elsevier.

Overall, these studies strengthen MXene‐polysulfide interactions from a chemical perspective, enabling more stable suppression of the shuttle effect than purely physical blocking strategies. Through defect engineering, heteroatom doping, and surface functional‐group regulation, the MXene interface can be precisely tuned to achieve stronger chemical anchoring of LiPSs. These advances provide an important foundation for the further introduction of catalytic functionalities into MXene‐based separator systems.

### 5.4 The Adsorption‐Catalysis Synergy Effect of MXene and the Kinetics of Interface Reactions

With the deepening understanding of the reaction mechanisms in Li–S batteries, researchers have gradually recognized that merely “capturing” LiPSs is insufficient to fundamentally solve the shuttle problem [385]. Instead, the key lies in accelerating their reversible conversion kinetics. Consequently, catalytic MXene‐based separators have become one of the most active research directions in recent years [386, 387].

The introduction of single‐atom catalytic systems has provided a new design paradigm for catalytic MXene separators. Among them, the work reported by Gu et al. [388] on Cu‐N_1_C_2_ single‐atom sites anchored on MXene substrates is particularly representative. In this study, asymmetrically coordinated Cu‐N_1_C_2_ active centers were constructed, which significantly modulate the local electronic structure of the metal sites at the atomic scale (Figure 20a). XAFS characterization (Figure 20b,c) confirms that Cu atoms are stabilized within the host matrix in a low‐coordination, asymmetric N_1_C_2_ configuration. Compared with conventional symmetric coordination environments, such electronically unsaturated active sites exhibit stronger polarization capability and higher catalytic activity. This work systematically elucidates the advantages of single‐atom catalytic separators from the perspectives of structural design, electronic‐state regulation, and reaction mechanisms, highlighting the concept of integrated adsorption‐catalysis regulation. Similar single‐atom strategies—including CoSA‐CoNC/NMX [389], MXene@CeSA [390], and Ni_0.7_Co_0.3_S_2_/MXene [391]—have also achieved significant improvements in sulfur reaction kinetics by regulating the coordination environment and electronic states of metal atoms, and therefore will not be discussed in detail here.

**FIGURE 20:**
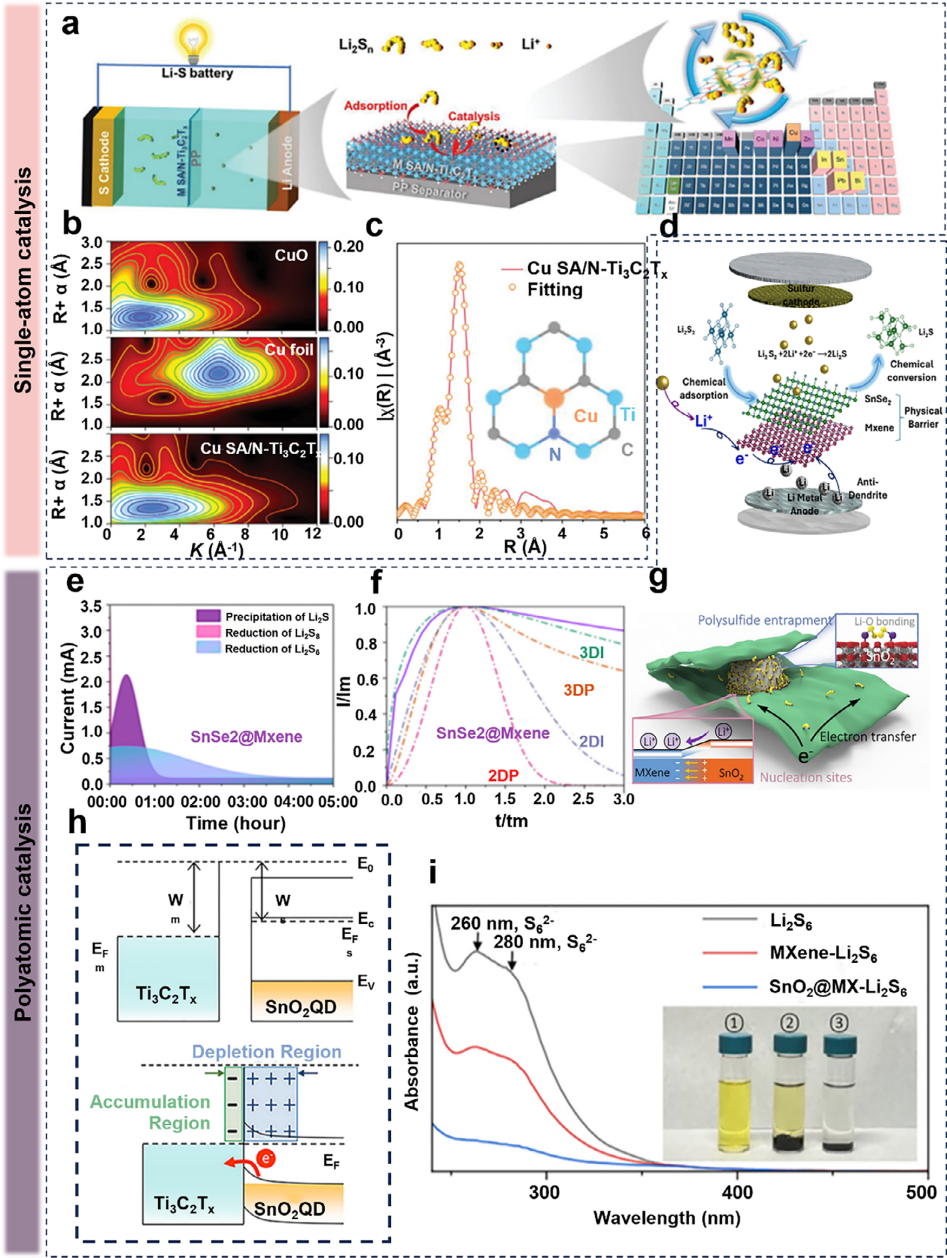
(a) Scheme of the Li–S batteries with M SA/N‐Ti_3_C_2_T_x_/PP. (b) WT‐EXAFS plots of CuO, Cu foil, and Cu SA/N‐Ti_3_C_2_Tx, respectively. (c) FT‐EXAFS fitting curves of Cu SA/N‐Ti_3_C_2_Tx. The inset is the local atomic structure of Cu (Ti, cyan; C, gray; N, blue; Cu, orange). Reproduced with permission [388]. Copyright 2023, Wiley‐VCH. (d) Schematic illustration of the multifunctional role of the SnSe_2_@MXene separator coating in Li–S batteries. PITT‐derived analysis of Li–S batteries with SnSe_2_@MXene separators: (e) decomposition into Li_2_S precipitation and Li_2_S_8_/Li_2_S_6_ reduction regimes; (f) normalized l/lm versus t/tm fits to the 2DI, 2DP, 3DI, and 3DP nucleation models. Reproduced with permission [392]. Copyright 2025, Elsevier. (g) Schematic illustration of the efficient LiPSs adsorption and catalytic conversion process in SnO_2_@MX heterostructures, where the 2D flakes are MXene (green color), LiPSs chains are adsorbed on SnO_2_ (sphere with white color) surface. (h) Energy band diagram of Mott–Schottky type contact between MXene (work function Wm = 4.37 eV) and SnO_2_ (work function Ws = 3.84eV) (upper before and (lower) after contacting. (i) From left to right, the solutions are: Li_2_S_6_ solution, MXene‐Li_2_S_6_ solution, and SnO_2_@MX‐Li_2_S_6_ solution [393]. Copyright 2024, Springer Nature.

Beyond single‐atom systems, heterostructures formed by combining MXene with metal sulfides, selenides, or phosphides have also demonstrated excellent catalytic performance. For example, in the SnSe_2_@MXene system proposed by Yu et al. [392], the catalytic mechanism of the separator is illustrated in Figure 20d. In this heterostructure, the interfacial synergy enables efficient adsorption, conversion, and shuttle suppression of lithium LiPSs. The regulation of Li_2_S nucleation and deposition behavior is shown in Figure 20e,f. At a more refined interfacial level, electronic‐structure engineering has gradually emerged as an important strategy for optimizing MXene‐based separators. A representative example is the SnO_2_ quantum dots@MXene system [393]. The ultrathin lamellar SnO_2_@MXene heterostructure significantly enhances interfacial adsorption of lithium LiPSs while accelerating their conversion kinetics. Meanwhile, it also regulates Li^+^ flux distribution, thereby suppressing the shuttle effect and improving lithium deposition behavior at the anode (Figure 20g). These performance enhancements originate from the uniform anchoring of SnO_2_ quantum dots on the surface and within the interlayers of MXene (Figure 20h,i). The difference in work functions between the two components induces the formation of a Mott–Schottky heterojunction, which establishes a built‐in electric field at the interface and redistributes the electronic states of MXene. This electronic reconstruction shifts the transition‐metal d‐band center toward the Fermi level, strengthening the chemical interaction with lithium LiPSs and lowering the reaction energy barrier. Similar built‐in electric‐field regulation strategies have also been widely validated in systems such as 1T‐VSe_2_@Ti_3_C_2_ [394], carbide/nitride multiphase heterostructures [309], and other MXene‐based interfacial catalytic systems [395]. These studies further highlight the universality and effectiveness of interfacial electric‐field engineering in optimizing sulfur reaction kinetics. Overall, this category of research marks a clear transition of MXene‐based separators from passive physical barriers to active catalytic interfaces, representing one of the key directions driving performance breakthroughs in Li–S batteries.

### 5.5 MXene Multifunctional Coordination and System Integration

Under practical operating conditions such as high sulfur loading, lean electrolyte, and long‐term cycling, a single functional mechanism is often insufficient to meet the performance requirements of Li–S batteries. Consequently, recent research has increasingly focused on multifunctional synergy [396] and system‐level integration strategies [397].

For instance, Fu et al. reported in 2025 a MXene/ZIF‐67 Janus separator, in which the cathode‐facing side suppresses polysulfide shuttling while the anode‐facing side homogenizes Li^+^ flux, thereby achieving bidirectional functional partitioning [398]. Similar concepts have also been demonstrated in asymmetric bimetallic‐MXene separators, which further highlight the effectiveness of structural asymmetry in achieving differentiated regulation at the cathode and anode interfaces [399]. While Janus or asymmetric separators realize functional differentiation through structural partitioning, another design philosophy aims to construct cathode‐separator cooperative systems through intrinsically multifunctional materials. A representative example of this “multi‐role material” strategy was reported by Bonilla et al. in 2025 using Ti_3_C_2_T_X_ MXene [400]. In this system, MXene simultaneously serves as a sulfur‐cathode additive and a separator modification layer, enabling cross‐interface regulation of polysulfide behavior between the cathode and separator. As illustrated schematically (Figure 21a), MXene catalyzes stepwise polysulfide conversion on the cathode side while forming an effective barrier layer and regulating Li^+^ migration on the separator side, thereby simultaneously optimizing reaction kinetics and suppressing the shuttle effect. Corresponding experimental results show that the MXene‐modified separator significantly promotes Li_2_S nucleation and conversion (Figure 21b) and effectively suppresses polysulfide diffusion across the separator (Figure 21c). This configuration demonstrates a “functional asymmetry” advantage, which differs from the structural asymmetry of conventional Janus separators. Furthermore, a cathode‐separator integrated architecture based on Nb‐doped MXene has also been reported to further reduce interfacial resistance and improve overall electrochemical performance [401].

**FIGURE 21:**
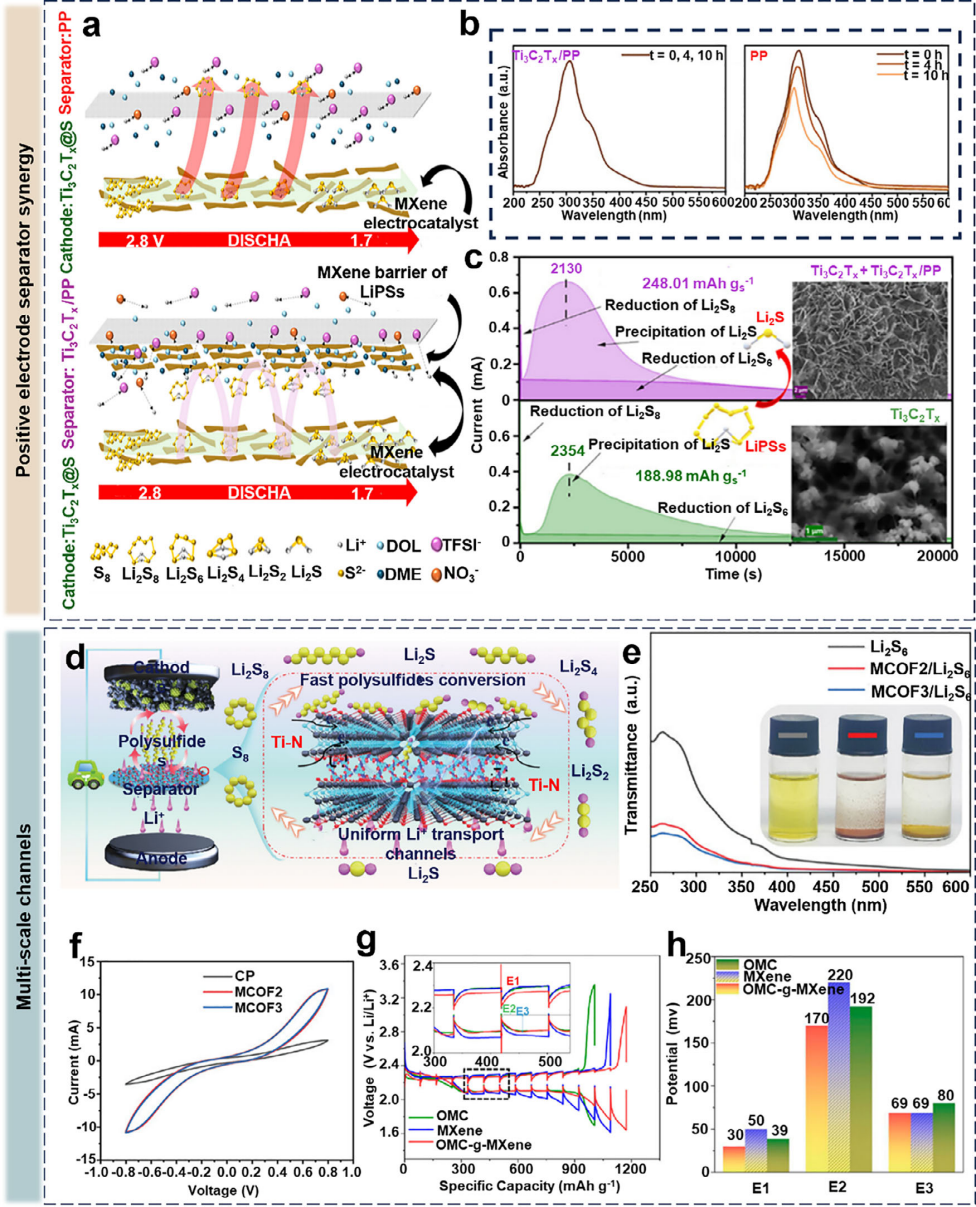
(a) Schematic illustration of the selected LiPS paths during discharge in the PP + Ti_3_C_2_T_x_@S (upper) and Ti_3_C_2_T_x_/PP + Ti_3_C_2_T_x_@S (lower) batteries. (b) UV–vis spectra for polysulfide permeation testing using H‐type visible batteries for the functionalized Ti_3_C_2_T_x_/PP separator (left) and the commercial separator (right) at 0, 4, and 10 h. (c) Potentiostatic discharge profiles of Li_2_S_8_ for the nucleation of Li_2_S for the Ti_3_C_2_T_x_+Ti_3_C_2_T_x_/PP (upper) and Ti_3_C_2_T_x_ (lower) batteries. Reproduced with permission [400]. Copyright 2025, Wiley‐VCH. (d) Schematic illustration of MCOF modified separator inhibiting polysulfide shuttle and lithium dendrite growth. (e) UV–vis spectra of Li_2_S_6_ solution with and without MCOF (inset: the corresponding electronic photos). (f) Corresponding fitting lines of peak current versus square root of the scan rates. Reproduced with permission [401]. Copyright 2025, Wiley‐VCH. (g) The GITT of Li–S batteries with batteries based on OMC, MXene and OMC‐g‐MXene at 0.1 C and (h) the corresponding overpotential calculated from GITT technique. Reproduced with permission [402]. Copyright 2023, American Chemical Society.

In addition, multidimensional composite networks and organic‐inorganic hybrid systems have shown unique advantages in improving the comprehensive performance of MXene‐based separators. For example, Yang et al. [402] reported a COF‐MXene hybrid separator, whose structure and working mechanism are illustrated in Figure 21d. In this design, the ordered nanopores of the covalent organic framework (COF) cooperate with the polar surface of MXene to construct multiscale channels that simultaneously enable polysulfide adsorption and Li^+^ transport regulation. The corresponding adsorption and diffusion behaviors were experimentally verified (Figure 21e,f), demonstrating that the hybrid network can simultaneously suppress polysulfide shuttling while promoting interfacial conversion reactions. Similarly, Li et al. [403] developed an OMC‐MXene ion‐pump separator, where ordered mesoporous carbon (OMC) forms a heterointerface with MXene. As illustrated in Figure 21g, this structure generates a directional Li^+^ flux at the separator interface and accelerates sulfur/polysulfide conversion reactions. The corresponding electrochemical performance and diffusion characteristics (Figure 21h) further confirm its advantages in achieving synergistic enhancement of ion regulation and reaction kinetics.

These studies demonstrate that multidimensional composite architectures and organic–inorganic hybrid strategies can achieve integrated regulation of chemical adsorption, catalytic conversion, and ion transport through synergistic structural and interfacial design [394, 404, 405, 406]. Such system‐integrated strategies represent an important transition of MXene‐based functional separators from material‐level optimization toward system‐level engineering, offering promising solutions for the practical application of high‐performance Li–S batteries.

### 5.6 PRI‐Guided Evaluation of MXene‐Modified Inorganic Separator Systems: From Mechanistic Advantage to Practical Limitation

To systematically evaluate MXene‐based and MXene‐hybrid separator systems under unified practical constraints, representative architectures are summarized in Table 13. These systems encompass a wide range of design strategies, including adsorption‐dominated interfaces, heterostructure engineering, defect modulation, and band‐structure tuning via MXene‐derived conductive frameworks. Compared with previously discussed material systems, MXene‐based architectures exhibit the highest structural diversity and interfacial tunability, leading to a broad distribution in electrochemical performance and practical relevance. Within the PRI framework, all systems are quantitatively evaluated in terms of energy contribution (ECS), electrolyte utilization efficiency (LECS), structural penalty (SPS), and device‐level validation (DVS), enabling a unified comparison of intrinsic activity enhancement versus structural optimization effects under identical constraints. Accordingly, Table S17 provides a comprehensive benchmark for identifying cornerstone, transition, and compromise regimes across MXene‐based separator system.

**TABLE 13:** Representative MXene modified separator systems for Li–S batteries, summarizing key electrochemical parameters and practical operating conditions, including sulfur loading, electrolyte‐to‐sulfur ratio, areal capacity, coating mass, separator thickness, and device‐level validation. This table highlights representative architectures that illustrate the evolution from adsorption‐dominated designs to defect‐engineered and heterostructure‐based systems.

System	Mechanism	Sulfur loading (mg cm^−2^)	E/S (μ L mg^−1^)	Areal capacity (mAh cm^−2^)	Testing condition (rate, cycles)	Coating mass (mg cm^−2^)	Thickness (μ m)	Pouch cell	Refs.
CM/MoS_2_ (Crumpled MXene/MoS_2_)	Adsorption‐catalysis synergistic	5.0	7	5.5	1C, 200 cycles	0.4	16	/	[317]
L‐MXene	Adsorption‐dominated separator	1.2	20	0.8	1C, 500 cycles	0.2	5	/	[369]
TA‐MXene	Adsorption‐catalysis synergistic separator	3.5	10	4.0	0.1C, 300 cycles	0.042	0.53	/	[370]
PSN/MXene 2D	Heterojunction/defect‐engineered separator	5.2	15	5.1	0.1C, 50 cycles	0.34	7	/	[371]
MXene/MoS_2_/SnS@C	Adsorption‐catalysis synergistic	5.1	7.84	4.22	0.2C, 50 cycles	0.5	12	/	[377]
Ag‐ZnSe@MXene	Adsorption‐catalysis synergistic separator	1.2	20	0.8	5C, 1000 cycles	0.3	10	/	[378]
Ni_0.7_Co_0.3_S_2_/MXene	Adsorption‐catalysis synergistic separator	4.0	7.5	4.6	0.2C, 100 cycles	0.95	0.29	/	[391]
1T‐VSe_2_@Ti_3_C_2_	Heterojunction/defect‐engineered separator	1.0	25	1.0	1C, 1000 cycles	0.4	10	/	[394]
Ti_3_C_2_T_x_@S Ti_3_C_2_T_x_/PP	Adsorption‐dominated separator	9.0	5.0	6.0	0.05C, 50 cycles	0.35	6	(S: 9.0 mg cm^−2^, > 60 mAh cm^−2^)	[407]
CoSA‐CoNC/NMX	Adsorption‐catalysis synergistic separator	10.0	4.5	6.32	0.1C, 80 cycles	4	4	/	[408]
Ti_3_C_2_ MXene	Adsorption‐catalysis synergistic separator	9.5	10	7.4	0.2C, 100 cycles	0.4	20	(S: 0.9g, 74.2%, 0.5C, 200 cycles)	[372]
CoNi@TiO_2_/C	Adsorption‐catalysis synergistic separator	3.33	20	4.2	0.5C, 100 cycles	0.5	25	/	[409]
Co‐CoP@MX	Adsorption‐catalysis synergistic separator	8.36	15	6.3	0.1C, 100 cycles	0.4	8	/	[410]
Zn‐Co‐Ni‐S@MXene@CNTs	Adsorption‐catalysis synergistic separator	2.0	20	1.6	0.5C, 400 cycles	0.4	12	/	[411]
Ti_3_C_2_T_x_‐UIO‐66‐NH_2_	Adsorption‐dominated separator	8.2	20	3.4	2.5C, 600 cycles	1.5	20	/	[412]
NiCoSAA‐MXene	Adsorption‐catalysis synergistic separator	4.7	15	3.1	0.1C, 100 cycles	0.35	18	/	[413]
NiS_2_@MXene	Adsorption‐catalysis synergistic separator	5.01	20	3.2	0.2C, 100 cycles	0.5	8	/	[414]
SnSe_2_@Ti_3_C_2_T_x_ MXene	Heterojunction/defect‐engineered separator	5.0	6.0	3.5	0.2C, 120 cycles	2.0	6.8	/	[392]
O‐Ti_2.7_Nb_0.3_C_2_T_x_	Band structure/built‐in electric field modulated separator	5.8	10.0	3.8	0.05C, 60 cycles	0.278	6	/	[382]
Co‐VC	Band structure/built‐in electric field modulated separator	7.6	4.0	9.0	0.1C, 50 cycles	0.3	8	/	[415]
Co_3_Fe_7_‐MXene	Adsorption‐catalysis synergistic separator	3.2	4.0	2.5	0.2C, 50 cycles	0.4	3	/	[416]
Ti_3_C_2_T_x_ MXene@CuCo_2_O_4_	Band structure/built‐in electric field modulated separator	10.0	2.6	9.4	0.2C, 100 cycles	0.6	4	(S: 10 mg cm^−2^, 74.2%, 0.2C, 100 cycles)	[417]
Co/CoN_4_@KB‐MXene	Adsorption‐catalysis synergistic separator	6.1	6.1	4.8	0.2C, 100 cycles	0.13	5	(0.1C, 60 cycles)	[418]
MXene@TBCOF	Heterojunction/defect‐engineered separator	9.31	10	7.14	0.1C, 90 cycles	0.31	17	(931.5 mAh g^−1^, 0.1C, 60 cycles)	[419]
MXene‐LDH	Adsorption‐dominated separator	5.0	30	3.2	0.1C, 50 cycles	0.2	<1(760 nm)	/	[420]
MXene/NiS_2_/Co_3_S_4_	Adsorption‐catalysis synergistic separator	10.0	15	5.8	0.2C, 80 cycles	0.8	16	/	[421]
NiTe_2_@MXene	Heterojunction/defect‐engineered separator	8.31	6.52	5.6	0.1C, 84 cycles	0.35	17.8	(903.9 mAh g^−1^, 0.1C, 80 cycles)	[422]
CACNM@PP	Adsorption‐catalysis synergistic separator	4.87	8.0	2.9	0.1C, 100 cycles	0.5	14	/	[399]
S/rGO@Ti_3_C_2_ QDs	Adsorption‐catalysis synergistic separator	5.3	6.0	4.0	0.1C, 50 cycles	0.3	5	/	[423]
Vo‐LDHs‐MXenes	Adsorption‐catalysis synergistic separator	6.7	6.0	5.06	0.1C, 50 cycles	0.3	4	/	[379]
ZnS/MoS_2_@MXene	Adsorption‐catalysis synergistic separator	5.6	15	3.6	0.2C, 100 cycles	0.5	21	/	[206]
SnO_2_ QDs/MXene	Heterojunction/defect‐engineered separator	7.5	10.0	6.0	0.02C, 50 cycles	0.1	0.54	/	[393]
Cu SA/N‐Ti_3_C_2_T_x_	Band structure/built‐in electric field modulated separator	7.19	/	5.28	0.1C, 50 cycles	0.2	10	/	[424]
BN@MXene	Band structure/built‐in electric field modulated separator	4.6	4.35	1.82	0.2C, 100 cycles	0.06	0.419	/	[425]
Ti_3_C_2_T_x_@CoSe_2_	Band structure/built‐in electric field	4.0	9	3.26	0.2C, 60 cycles	0.2	15		[426]
CNVM (N, V‐codoped MXene)	Adsorption‐catalysis synergistic	7.2	10	3.0	0.1C, 50 cycles	0.38	13	/	[427]
Co_3_Se_4_@N‐C/Ti_3_C_2_T_x_	Adsorption‐catalysis synergistic	2.24	/	2.37	0.2C, 300 cycles	0.4	15	/	[428]
Fe_3_Se_4_/FeSe@MXene	Band structure/built‐in electric field	5.8	3.45	5.0	0.2C, 120 cycles	/	16	/	[429]
NiS_2_/MXene	Adsorption‐catalysis synergistic	5.25	8	5.76	0.2C, 50 cycles	3.4	8	/	[430]
Ti_3_C_2_T_x_‐CoBDC	Adsorption‐catalysis synergistic	7.5	20	8.22	0.2C, 100 cycles	0.1	5	/	[431]
Co_1‐x_S/3D‐Ti_3_C_2_T_x_	Adsorption‐catalysis synergistic	4.2	4.76	4.87	0.2C, 50 cycles	0.15	/	/	[432]
Pt SAs/In_2_S_3_/Ti_3_C	Adsorption‐catalysis synergistic	6.4	10	5.54	0.2C, 300 cycles	0.43	8.94	/	[433]
Ti_3_C_2_T_x_/Ni‐Co MOF	Adsorption‐catalysis synergistic	5.8	4	4.23	0.1C, 80 cycles	0.2	4.5	/	[434]
Ti_3_C_2_/CPNC (CoP)	Adsorption‐catalysis synergistic	5.4	8	7.8	0.1C, 120 cycles	0.8	15	/	[435]
V_4_C_3_T_x_	Adsorption‐catalysis synergistic	4.0	10	4.3	0.1C, 120 cycles	1.0	2.7	/	[436]
N‐MX‐CoS_2_	Adsorption‐catalysis synergistic	2.5	20	/	0.5 C, 200 cycles	0.2	15	/	[189]
V_2_C/V_2_O_5_/CNTs	Band structure/built‐in electric field	6.0	4.4	5.9	0.5 C, 100 cycles	0.9	10	/	[437]
Ti_3_C_2_T_x_/CNTs‐PP	Adsorption‐dominated separator	1.2	/	0.77	1 C, 200 cycles	0.016	0.1	/	[438]
N‐Ti_3_C_2_/C@PP	Adsorption‐dominated separator	3.4	8	2.43	0.5 C, 500 cycles	0.6	6	/	[439]

The PRI‐based evaluation of MXene‐based and MXene‐hybrid separator systems reveals the most diverse and highly dispersed performance landscape among all material classes investigated in this work. This diversity arises from the multifunctional role of MXenes, which simultaneously act as conductive frameworks, catalytic hosts, and structural regulators. Notably, several optimized architectures, including Ti_3_C_2_‐based heterostructures, oxide‐modified MXenes, and quantum dot‐decorated systems, achieve true cornerstone‐level performance by simultaneously maximizing energy contribution, improving electrolyte utilization, and minimizing structural penalties. These systems demonstrate that MXene can effectively serve as a universal platform for integrating multiple functional components into a balanced separator design. However, despite their structural versatility, a significant number of MXene‐based composites remain compromise‐dominated due to excessive structural loading, incomplete interfacial optimization, or inefficient electrolyte utilization. This indicates that MXene alone does not guarantee high practical relevance; rather, its performance strongly depends on rational interface engineering and structural minimization strategies. Overall, MXene‐based systems represent a “high‐dimensional design space” within the PRI framework, where both cornerstone and compromise behaviors coexist depending on the balance between structural engineering and electrochemical functionality.

## 6 Unified Constraint‐Driven Framework for Separator Engineering in Li–S Batteries

To systematically evaluate separator performance in Li–S batteries under highly heterogeneous experimental conditions, we develop a constraint‐aware evaluation framework based on the PRI. This framework is designed to bridge intrinsic electrochemical performance and system‐level application constraints, enabling a unified interpretation of reported separator systems. Within this framework, a localized quantitative descriptor (figure‐of‐merit, FoM) is introduced as a complementary analytical tool. Rather than serving as an independent scoring or ranking metric, the FoM captures intrinsic electrochemical contributions associated with separator modification, including areal energy contribution, cycling stability evolution, and normalized reaction efficiency under comparable electrochemical conditions. These intrinsic contributions correspond to the cornerstone component of the PRI framework, reflecting the ability of inorganic separators to enhance polysulfide adsorption, accelerate catalytic conversion, and regulate interfacial charge transfer kinetics. In contrast, the PRI framework further incorporates system‐level compromise effects, including sulfur loading, electrolyte‐to‐sulfur ratio, separator‐induced transport resistance, and inactive mass contribution, which collectively determine the practical applicability of separator systems. Accordingly, FoM and PRI are not independent or competing evaluation systems. Instead, FoM is interpreted as an intrinsic projection within the PRI constraint space, whereas PRI provides a comprehensive description of both electrochemical enhancement and practical energy‐density limitations. Importantly, all FoM and PRI results are intended to represent semi‐quantitative trends rather than absolute cross‐system rankings, due to inherent heterogeneity in reported experimental conditions across literature studies. A representative implementation of the FoM‐PRI mapping procedure is provided in the Supporting Information (Figure S2) to illustrate the calculation workflow and ensure methodological transparency without interrupting the main conceptual narrative. Based on the constraint‐aware PRI framework, the comparative analysis of representative separator systems is further visualized in Figure 22 to illustrate how intrinsic electrochemical contributions and system‐level penalties jointly determine practical performance outcomes. Unlike the FoM representation, which focuses on intrinsic electrochemical projections, Figure 22 reflects the integrated PRI perspective, where both cornerstone effects and compromise factors are simultaneously considered to evaluate practical applicability. The detailed formulas of the FoM framework are provided in Supporting Information S2.

**FIGURE 22:**
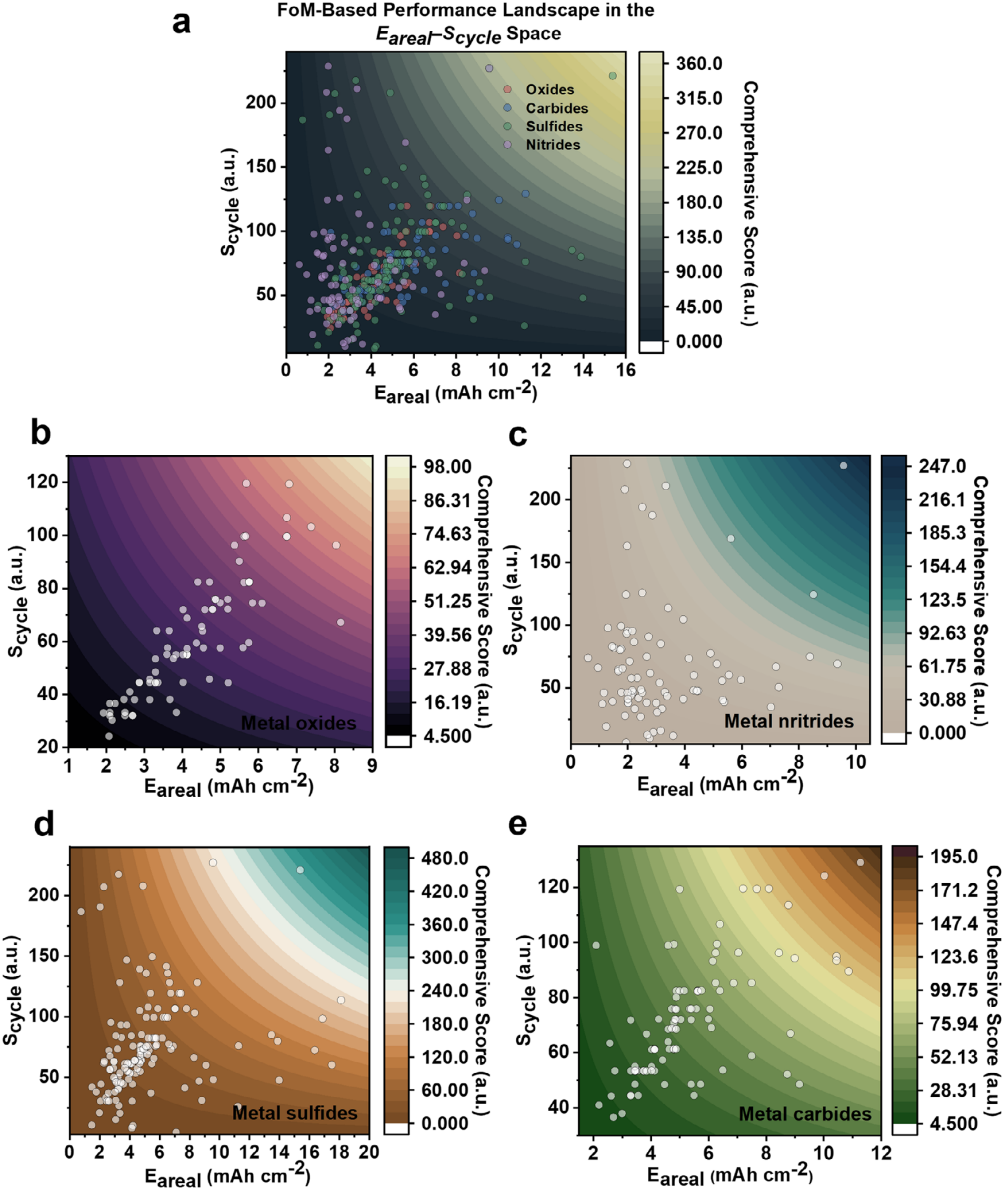
(a) Global contour map of separator performance projected in the *E*_areal_ − *S*_cycle_space, where the background color encodes the magnitude of the FoM as an intrinsic electrochemical projection within the PRI framework. In this representation, the performance landscape is interpreted as a continuous constraint space, where both intrinsic electrochemical enhancement and system‐level limitations jointly determine the accessible region. The upper‐right region corresponds to the simultaneous optimization of areal energy output and cycling stability under unified constraints. (b–e) Material‐dependent PRI‐based contour distributions for (b) metal oxides, (c) metal nitrides, (d) metal sulfides, and (e) metal carbides. Distinct occupancy patterns are observed across different material families within the constraint space. Oxides and carbides are mainly distributed in a constrained region, reflecting limited capability in simultaneously balancing polysulfide regulation and reaction kinetics. In contrast, sulfides and nitrides exhibit a broader distribution range, indicating enhanced synergy between intrinsic electrochemical activity and interfacial charge‐transfer processes. Importantly, these contour maps should not be interpreted as absolute performance rankings, but rather as a constraint‐governed visualization of separator behavior under heterogeneous experimental conditions, enabling a unified comparison of different material strategies within the PRI framework.

To address the intrinsic limitations caused by heterogeneous literature data and the lack of a constraint‐consistent evaluation framework for separator performance, a comprehensive performance landscape is constructed within the PRI framework, where the background distribution encodes the intrinsic electrochemical contribution projected by the FoM (Figure 22a). In this representation, separator performance is interpreted as a continuous constraint‐driven space, where both electrochemical enhancement and system‐level penalties jointly determine the accessible performance region. Unlike isolated FoM‐based evaluations, this PRI‐guided mapping avoids direct numerical ranking and instead focuses on the distribution of achievable performance under heterogeneous experimental constraints. As shown in Figure 22b–e, different material families exhibit distinct occupancy patterns within this constraint space. Oxide‐ and carbide‐based systems are primarily concentrated within a relatively constrained region, reflecting their limited capability in simultaneously balancing polysulfide regulation and reaction kinetics. In contrast, sulfide‐ and nitride‐based systems exhibit a broader distribution across the PRI space, indicating enhanced synergy between adsorption and catalytic conversion processes. However, despite this expanded distribution, a persistent trade‐off between high energy output and cycling stability remains evident. Systems achieving high initial performance are often associated with lower sulfur loading conditions, whereas highly stable systems tend to operate under reduced capacity regimes. Only a limited number of advanced designs occupy the upper‐right region of the PRI space, where both intrinsic electrochemical performance and practical constraints are simultaneously optimized. Collectively, this PRI‐based evaluation framework provides a unified perspective for interpreting separator performance across heterogeneous literature reports. It resolves the “scale mismatch” issue in conventional comparisons by integrating intrinsic electrochemical descriptors (FoM projection) with system‐level constraints, thereby enabling a physically consistent and application‐relevant assessment of separator engineering strategies.

## 7 Conclusion and Perspective

Overall, metal compound‐based separator engineering is evolving from isolated functional modification toward a constraint‐driven, system‐integrated interfacial design paradigm for Li–S batteries. This evolution is consistently reflected across three interconnected dimensions: intrinsic electrochemical functionality, interfacial reaction regulation, and full‐cell system integration. At the intrinsic level, separator materials are progressively transitioning from adsorption‐dominated polysulfide confinement toward reaction‐pathway‐oriented electrochemical regulation, where catalytic conversion, charge redistribution, and electronic‐structure modulation collectively determine sulfur redox kinetics. This behavior is captured as the intrinsic projection within the PRI framework, corresponding to the cornerstone contribution of separator engineering. At the same time, practical implementation introduces unavoidable system‐level constraints, including inactive mass accumulation, electrolyte limitation, transport resistance, and cell‐level energy dilution. These competing effects define the compromise space of separator design, which must be jointly considered with intrinsic activity to avoid misleading performance interpretation. In this context, the PRI framework provides a unified constraint‐aware perspective that links intrinsic electrochemical descriptors with practical system limitations, while the FoM serves as a localized projection of intrinsic behavior within this constraint space. Together, they enable a consistent interpretation of separator performance across heterogeneous literature conditions. As illustrated in the developmental roadmap (Figure 23), future separator design will further evolve toward dynamic, adaptive, and system‐level integrated architectures, ultimately converging toward cathode‐separator‐anode co‐design, closed‐loop electrochemical regulation, and AI‐assisted materials discovery. However, the success of these advanced systems will depend critically on their compatibility with practical operating conditions, including high sulfur loading, lean electrolyte environments, scalable fabrication, and long‐term cycling stability. By integrating mechanistic understanding, constraint‐aware evaluation, and system‐level design principles, metal compound‐based separators are expected to transition from laboratory‐scale functional components toward practically deployable interfacial architectures for next‐generation high‐energy‐density Li–S batteries.

**FIGURE 23:**
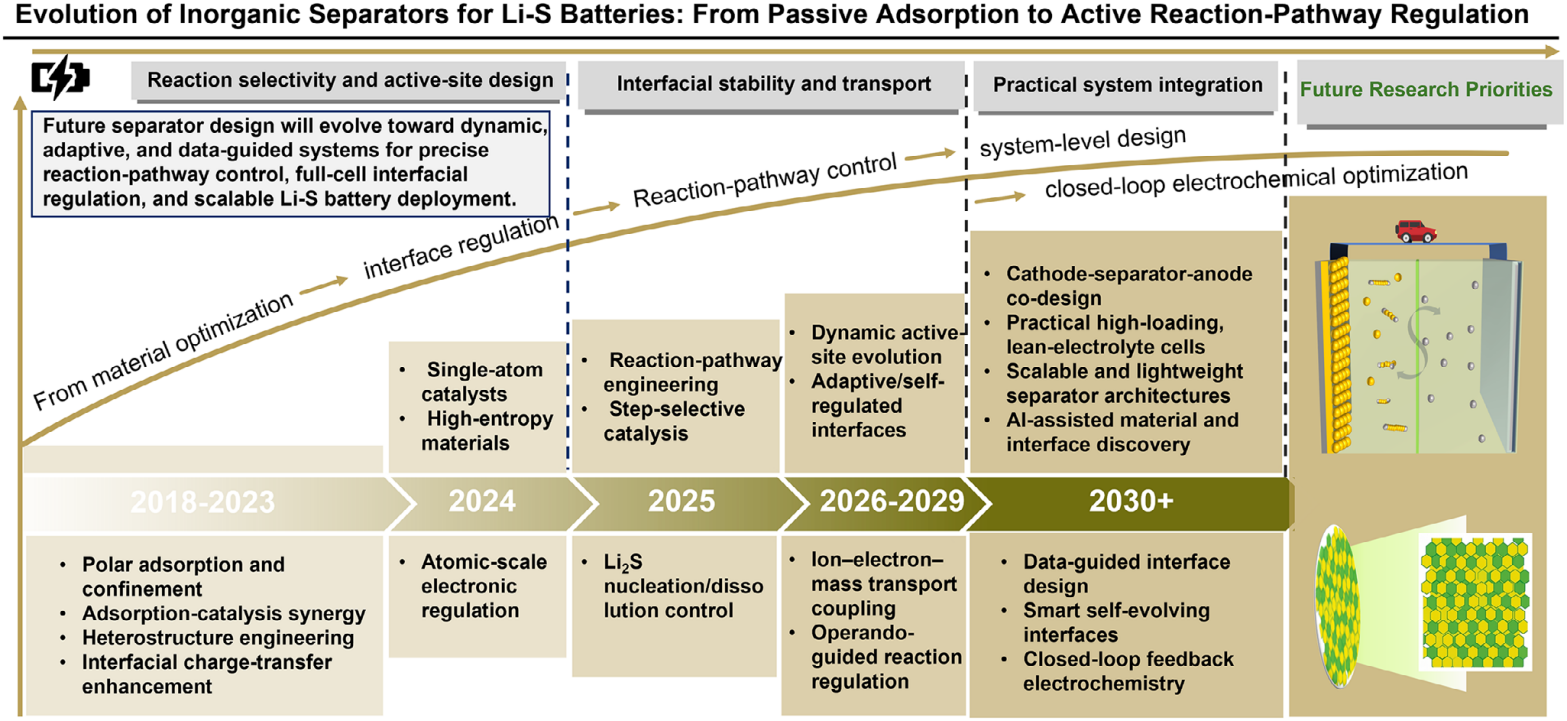
Future prospects of inorganic material‐modified separator in Li–S batteries.

## Funding

This work is supported by the National Natural Science Foundation of China (Grant No. 22472145), the Postgraduate Research & Practice Innovation Program of Jiangsu Province (Yangzhou University) (KYCX24_3729).

## Conflicts of Interest

The authors declare no conflicts of interest.

## Supporting information




**Supporting File**: adma74605‐sup‐0001‐SuppMat.docx.

## Data Availability

The data that support the findings of this study are available from the corresponding author upon reasonable request.

## References

[1] T. Bolsen , “Framing Renewable Energy,” Nature Energy 7 (2022): 1003–1004, 10.1038/s41560-022-01100-y.

[2] K. Detka and K. Górecki , “Selected Technologies of Electrochemical Energy Storage—A Review,” Energies 16 (2023): 5034, 10.3390/en16135034.

[3] X. Zhang , H. Xie , C. S. Kim , K. Zaghib , A. Mauger , and C. M. Julien , “Advances in Lithium—Sulfur Batteries,” Materials Science and Engineering: R: Reports 121 (2017): 1–29, 10.1016/j.mser.2017.09.001.

[4] Q. Pang , X. Liang , C. Y. Kwok , J. Kulisch , and L. F. Nazar , “A Comprehensive Approach toward Stable Lithium–Sulfur Batteries With High Volumetric Energy Density,” Advanced Energy Materials 7 (2017): 1601630, 10.1002/aenm.201601630.

[5] C. Zhang , Y. Yang , X. Liu , et al., “Mobile Energy Storage Technologies for Boosting Carbon Neutrality,” Innovation 4 (2023): 100518, 10.1016/j.xinn.2023.100518. PMC10568306 37841885

[6] S. M. Sirin and B. N. Yilmaz , “Energy Transition and Non‐Energy Firms′ Financial Performance: Do Markets Value Capability‐Based Energy Transition Strategies?,” Energy Economics 136 (2024): 107658, 10.1016/j.eneco.2024.107658.

[7] L. Carbone , S. G. Greenbaum , and J. Hassoun , “Lithium Sulfur and Lithium Oxygen Batteries: New Frontiers of Sustainable Energy Storage,” Sustainable Energy & Fuels 1 (2017): 228–247, 10.1039/C6SE00124F.

[8] Q. J. Shao , S. D. Zhu , and J. Chen , “A Review on Lithium‐Sulfur Batteries: Challenge, Development, and Perspective,” Nano Research 16 (2023): 8097–8138, 10.1007/s12274-022-5227-0.

[9] C. Li , R. Liu , Y. Xiao , F. F. Cao , and H. Zhang , “Recent Progress of Separators in Lithium‐Sulfur Batteries,” Energy Storage Materials 40 (2021): 439–460, 10.1016/j.ensm.2021.05.034.

[10] H. Li , S. Ma , J. Li , et al., “Altering the Reaction Mechanism to Eliminate the Shuttle Effect in Lithium‐Sulfur Batteries,” Energy Storage Materials 26 (2020): 203–212, 10.1016/j.ensm.2020.01.002.

[11] C. X. Bi , N. Yao , X. Y. Li , et al., “Unveiling the Reaction Mystery Between Lithium Polysulfides and Lithium Metal Anode in Lithium–Sulfur Batteries,” Advanced Materials 36 (2024): 2411197, 10.1002/adma.202411197. 39149771

[12] J. Sun , Y. Liu , L. Liu , et al., “Interface Engineering Toward Expedited Li_2_S Deposition in Lithium–Sulfur Batteries: A Critical Review,” Advanced Materials 35 (2023): 2211168, 10.1002/adma.202211168. 36756778

[13] Z. Zhu , Y. Dong , S. Zhou , et al., “Strategies for Inhibiting the ‘Shuttle Effect’ in Lithium‑Sulfur Batteries: Surface/Interface Modulation and Bulk Phase Optimization,” Coordination Chemistry Reviews 548 (2026): 217220, 10.1016/j.ccr.2025.217220.

[14] Y. Z. Huang , L. Lin , C. K. Zhang , et al., “Recent Advances and Strategies toward Polysulfides Shuttle Inhibition for High‐Performance Li–S Batteries,” Advanced Science 9 (2022): 2106004, 10.1002/advs.202106004. PMC9036004 35233996

[15] Z. W. Seh , W. Li , J. J. Cha , et al., “Sulphur–TiO_2_ yolk–shell nanoarchitecture with internal void space for long‐cycle lithium–sulphur batteries,” Nature Communications 4 (2013): 1331, 10.1038/ncomms2327. 23299881

[16] Z. Li , J. Wang , H. Yuan , Y. Yu , and Y. Tan , “Recent Progress and Challenge in Metal–Organic Frameworks for Lithium–Sulfur Battery Separators,” Advanced Functional Materials 34 (2024): 2405890, 10.1002/adfm.202405890.

[17] L. P. Hou , N. Yao , J. Xie , et al., “Modification of Nitrate Ion Enables Stable Solid Electrolyte Interphase in Lithium Metal Batteries,” Angewandte Chemie International Edition 61 (2022): e202201406, 10.1002/anie.202201406. 35233916

[18] C. Y. Im , G. Y. Lee , J. G. Kim , J. H. Choi , and S. J. Kim , “Controlling Lithium Surface Diffusivity via 2D PtTe_2_, PdTe_2_, and NiTe_2_ Coatings for Anode‐Free and Lithium Metal Batteries,” Advanced Materials 37 (2025): 2501261, 10.1002/adma.202501261. PMC12369682 40451751

[19] L. Wu , Z. Jin , X. Xie , F. Lian , J. Lu , and W. Wang , “Lithium‐Reinforced Polyoxometalate as Effective Catalytic Interlayer for High‐Sulfur‐Loading and Long‐Life Lithium‐Sulfur Batteries,” Energy Storage Materials 77 (2025): 104167, 10.1016/j.ensm.2025.104167.

[20] S. Liu , J. Li , X. Yan , et al., “Superhierarchical Cobalt‐Embedded Nitrogen‐Doped Porous Carbon Nanosheets as Two‐in‐One Hosts for High‐Performance Lithium–Sulfur Batteries,” Advanced Materials 30 (2018): 1706895, 10.1002/adma.201706895. 29423940

[21] H. Han , S. Niu , Y. Zhao , T. Tan , and Y. Zhang , “TiO_2_/Porous Carbon Composite‐Decorated Separators for Lithium/Sulfur Battery,” Nanoscale Research Letters 14 (2019): 176, 10.1186/s11671-019-3010-2. PMC6538749 31140042

[22] S. Nong , D. Huang , Y. Li , et al., “Multifunction‐Balanced Porous Carbon and Its Application in Sulfur‐Loading Host and Separator Modification for Lithium–Sulfur Batteries,” Journal of Energy Storage 81 (2024): 110296, 10.1016/j.est.2023.110296.

[23] Y. Li , T. Gao , D. Ni , et al., “Two Birds With One Stone: Interfacial Engineering of Multifunctional Janus Separator for Lithium–Sulfur Batteries,” Advanced Materials 34 (2022): 2107638, 10.1002/adma.202107638. 34762349

[24] X. Lv , T. Lei , B. Wang , et al., “An Efficient Separator With Low Li‐Ion Diffusion Energy Barrier Resolving Feeble Conductivity for Practical Lithium–Sulfur Batteries,” Advanced Energy Materials 9 (2019): 1901800, 10.1002/aenm.201901800.

[25] K. Zhu , L. Li , P. Xue , et al., ““Three‐in‐one” Strategy: Heat Regulation and Conversion Enhancement of a Multifunctional Separator for Safer Lithium–Sulfur Batteries,” Carbon Energy 5 (2023): 352, 10.1002/cey2.352.

[26] R. Yang , F. Wang , W. Cui , et al., “Regenerative Redox Mediator for the Suppression of Dead Lithium for Lithium Sulfur Pouch Cell,” Energy Storage Materials 75 (2025): 104030, 10.1016/j.ensm.2025.104030.

[27] X. Li , R. Yang , L. Xia , et al., “Engineering a Dual‐Functional Polyarylether Nitrile Nanofibrous Framework for Stable Lithium Metal Anodes in Low N/P Lithium Metal Anodes,” Advanced Functional Materials 36 (2026): 09246, 10.1002/adfm.202509246.

[28] N. Wei , J. Chen , B. Shang , et al., “Shear Force Field‐Tailored Fibrillar Active Layer Morphology for High‐Efficiency Organic Solar Cells,” Advanced Functional Materials 36 (2026): 24598, 10.1002/adfm.202524598.

[29] X. Liu , J. Q. Huang , Q. Zhang , and L. Mai , “Nanostructured Metal Oxides and Sulfides for Lithium–Sulfur Batteries,” Advanced Materials 29 (2017): 1601759, 10.1002/adma.201601759. 28160327

[30] G. Zhu , Q. Wu , X. Zhang , et al., “Designing Metal Sulfide‐Based Cathodes and Separators for Suppressing Polysulfide Shuttling in Lithium‐Sulfur Batteries,” Nano Research 17 (2024): 2574–2591, 10.1007/s12274-023-6227-4.

[31] H. Zhang , R. Dai , S. Zhu , L. Zhou , Q. Xu , and Y. Min , “Bimetallic Nitride Modified Separator Constructs Internal Electric Field for High‐Performance Lithium‐Sulfur Battery,” Chemical Engineering Journal 429 (2022): 132454, 10.1016/j.cej.2021.132454.

[32] J. L. Cheong , C. Hu , W. Liu , M. F. Ng , M. B. Sullivan , and J. Y. Ying , “3d Carbonaceous Nanostructured Transition Metal Nitride, Carbonitride and Carbide as Polysulfide Regulators for Lithium‐Sulfur Batteries,” Nano Energy 102 (2022): 107659, 10.1016/j.nanoen.2022.107659.

[33] H. Wang , W. Zhang , J. Xu , and Z. Guo , “Advances in Polar Materials for Lithium–Sulfur Batteries,” Advanced Functional Materials 28 (2018): 1707520, 10.1002/adfm.201707520.

[34] J. Guo , Y. Huang , S. Zhao , et al., “Array‐Structured Double‐Ion Cooperative Adsorption Sites as Multifunctional Sulfur Hosts for Lithium–Sulfur Batteries With Low Electrolyte/Sulfur Ratio,” ACS Nano 15 (2021): 16322–16334, 10.1021/acsnano.1c05536. 34590488

[35] Q. Xu , H. Liu , S. Lv , et al., “Dynamic Anchoring and Catalysis via Co‐Nb Dual Sites in Lithium‐Sulfur Batteries,” Journal of Energy Chemistry 111 (2025): 719–727, 10.1016/j.jechem.2025.08.016.

[36] X. Hao , J. Ma , X. Cheng , et al., “Electron and Ion Co‐Conductive Catalyst Achieving Instant Transformation of Lithium Polysulfide Towards Li_2_S,” Advanced Materials 33 (2021): 2105362, 10.1002/adma.202105362. 34658075

[37] M. Zhang , W. Chen , L. Xue , et al., “Adsorption‐Catalysis Design in the Lithium‐Sulfur Battery,” Advanced Energy Materials 10 (2020): 1903008, 10.1002/aenm.201903008.

[38] C. Ye , Y. Jiao , H. Jin , et al., “2D MoN‐VN Heterostructure To Regulate Polysulfides for Highly Efficient Lithium‐Sulfur Batteries,” Angewandte Chemie International Edition 57 (2018): 16703–16707, 10.1002/anie.201810579. 30325094

[39] F. Ma , X. Xiong , Z. Zhang , et al., “Interface Engineering of Heterostructural Quantum Dots Towards High‐Rate and Long‐Life Lithium‐Sulfur Full Batteries,” Nano Energy 133 (2025): 110445, 10.1016/j.nanoen.2024.110445.

[40] K. Zou , T. Zhou , Y. Chen , et al., “Defect Engineering in a Multiple Confined Geometry for Robust Lithium–Sulfur Batteries,” Advanced Energy Materials 12 (2022): 2103981, 10.1002/aenm.202103981.

[41] J. Zhou , S. Sun , X. Zhou , et al., “Defect Engineering Enables an Advanced Separator Modification for High‐Performance Lithium‐Sulfur Batteries,” Chemical Engineering Journal 487 (2024): 150574, 10.1016/j.cej.2024.150574.

[42] D. He , X. Zhang , L. Chen , et al., “Thermoelectric Field Enhanced Sulfur Evolution Kinetics for High Performance Lithium‐Sulfur Batteries,” Advanced Materials 37 (2025): 2500457, 10.1002/adma.202500457. 40357839

[43] S. Wei , J. Liu , F. Lv , et al., “Doping Strategy for Regulating CoSe_2_ Electronic Structure to Enhance Sulfur Reaction Kinetics in Lithium–Sulfur Batteries,” Nano Research 18 (2025): 94908022, 10.26599/NR.2025.94908022.

[44] Y. Xiao , S. Guo , Y. Xiang , et al., “Engineering Configuration Compatibility and Electronic Structure in Axially Assembled Metal–Organic Framework Nanowires for High‐Performance Lithium Sulfur Batteries,” ACS Energy Letters 8 (2023): 5107–5115, 10.1021/acsenergylett.3c01698.

[45] H. Song , T. T. Nguyen , R. Chu , Y. Bai , N. H. Kim , and J. H. Lee , “Coupling Interfacial Effect in Heterogeneous RuP_2_‐RuP for Accelerating Sulfur Reduction Reaction of Lithium Sulfur Batteries,” Nano Energy 128 (2024): 109859, 10.1016/j.nanoen.2024.109859.

[46] X. Li , J. Zhu , R. Sheng , et al., “Defect‐Mediated Dual Role Interfaces Enables the Sulfur Reduction Reaction Kinetics for Highly Stable Lithium Sulfur Batteries,” Chemical Engineering Journal 525 (2025): 170497, 10.1016/j.cej.2025.170497.

[47] Y. H. Liu , L. X. Li , A. Y. Wen , F. F. Cao , and H. Ye , “A Janus MXene/MOF Separator for the All‐in‐One Enhancement of Lithium‐Sulfur Batteries,” Energy Storage Materials 55 (2023): 652–659, 10.1016/j.ensm.2022.12.028.

[48] Q. Wei , L. Wu , Y. Ye , et al., “Spatially Engineered Janus Separator Enables Bidirectional Regulation of Sulfur Electrochemistry and Lithium Deposition in High‐Energy‐Density Lithium–Sulfur Pouch Cells,” Advanced Functional Materials 36 (2026): 21571, 10.1002/adfm.202521571.

[49] M. A. Al‐Tahan , B. Miao , S. Xu , et al., “The “dual‐Layer Sulfur Cathode” Strategy: An In_2_S_3_/Bi_2_S_3_@rGO Heterostructure as an Interlayer/Modified Separator for Boosting the Areal Capacities of Lithium‐Sulfur Batteries,” Journal of Colloid and Interface Science 654 (2024): 753–763, 10.1016/j.jcis.2023.10.081. 37866047

[50] H. Pei , R. Guo , Y. Li , W. Liu , and J. Xie , “Integrated Design of Cathode and Separator for Lithium‐Sulfur Battery,” in ECS Meeting Abstracts MA2016‐03 (The Electrochemical Society, 2016), 1155, 10.1149/MA2016-03/2/1155.

[51] C. Fan , R. Yang , B. Chen , et al., “Multifunctional Integrated Separator Based on Electrospinning Structure Engineering for High‐Stability Lithium Sulfur Batteries,” Chemical Engineering Journal 521 (2025): 166813, 10.1016/j.cej.2025.166813.

[52] Z. Zhou , B. Chen , T. Fang , et al., “A Multifunctional Separator Enables Safe and Durable Lithium/Magnesium–Sulfur Batteries under Elevated Temperature,” Advanced Energy Materials 10 (2020): 1902023, 10.1002/aenm.201902023.

[53] X. Liang , C. Y. Kwok , F. Lodi‐Marzano , et al., “Tuning Transition Metal Oxide–Sulfur Interactions for Long Life Lithium Sulfur Batteries: The “Goldilocks” Principle,” Advanced Energy Materials 6 (2016): 1501636, 10.1002/aenm.201501636.

[54] G. Lin , M. Liang , L. Liu , et al., “P–P Orbital Interaction Enables Single‐Crystalline Semimetallic β‐MoTe_2_ Nanosheets as Efficient Electrocatalysts for Lithium–Sulfur Batteries,” ACS Applied Materials & Interfaces 14 (2022): 55616–55626, 10.1021/acsami.2c17326. 36475586

[55] X. Kang , T. He , H. Dang , et al., “Designing Amino Functionalized Titanium‐Organic Framework on Separators Toward Sieving and Redistribution of Polysulfides in Lithium‐Sulfur Batteries,” Nano‐Micro Letters 17 (2025): 277, 10.1007/s40820-025-01733-0. PMC12106290 40418466

[56] S. Fan , H. Q. Cao , J. Song , M. Cao , and J. Su , “Interfacial Charge‐Transfer Enhances the Dual‐Function of the Porous NiCo_2_S_4_@SiO_2_ Heterojunction for High‐Performance Li–S Batteries,” Chemical Communications 58 (2022): 1155–1158, 10.1039/D1CC05707C. 34981089

[57] S. Qiu , X. Liang , S. Niu , Q. Chen , G. Wang , and M. Chen , “Quantitative Defect Regulation of Heterostructures for Sulfur Catalysis Toward Fast and Long Lifespan Lithium‐Sulfur Batteries,” Nano Research 15 (2022): 7925–7932, 10.1007/s12274-022-4453-9.

[58] S. W. Han , G. B. Cha , K. Kim , and S. C. Hong , “Hydrogen Interaction with a Sulfur‐Vacancy‐Induced Occupied Defect State in the Electronic Band Structure of MoS_2_ ,” Physical Chemistry Chemical Physics 21 (2019): 15302–15309, 10.1039/C9CP01030K. 31025992

[59] A. Kim , S. H. Oh , A. Adhikari , B. R. Sathe , S. Kumar , and R. Patel , “Recent Advances in Modified Commercial Separators for Lithium–Sulfur Batteries,” Journal of Materials Chemistry A 11 (2023): 7833–7866, 10.1039/D2TA09266B.

[60] R. Yi , X. Lin , Y. Zhao , et al., “Fabrication of a Light‐Weight Dual‐Function Modified Separator Towards High‐Performance Lithium‐Sulfur Batteries,” ChemElectroChem 6 (2019): 3648–3656, 10.1002/celc.201900670.

[61] N. S. Grundish , C. D. Amos , A. Agrawal , H. Khani , and J. B. Goodenough , “Low‐Cost Self‐Assembled Oxide Separator for Rechargeable Batteries,” Advanced Functional Materials 29 (2019): 1903550, 10.1002/adfm.201903550.

[62] P. Feng , K. Dong , Y. Xu , et al., “Efficient and Homogenous Precipitation of Sulfur Within a 3D Electrospun Heterocatalytic Rutile/Anatase TiO_2‐x_ Framework in Lithium–Sulfur Batteries,” Advanced Fiber Materials 6 (2024): 810–824, 10.1007/s42765-024-00380-1.

[63] Z. Du , C. Guo , L. Wang , et al., “Atom‐Thick Interlayer Made of CVD‐Grown Graphene Film on Separator for Advanced Lithium–Sulfur Batteries,” ACS Applied Materials & Interfaces 9 (2017): 43696–43703, 10.1021/acsami.7b14195. 29172433

[64] Y. Wang , L. F. Zhu , J. X. Wang , Z. Zhang , J. Yu , and Z. Y. Yang , “Enhanced Chemisorption and Catalytic Conversion of Polysulfides via CoFe@NC Nanocubes Modified Separator for Superior Li–S Batteries,” Chemical Engineering Journal 433 (2022): 133792, 10.1016/j.cej.2021.133792.

[65] Z. L. Wu , S. X. Chen , L. Wang , et al., “Implanting Nickel and Cobalt Phosphide Into Well‐Defined Carbon Nanocages: A Synergistic Adsorption‐Electrocatalysis Separator Mediator for Durable High‐Power Li‐S Batteries,” Energy Storage Materials 38 (2021): 381–388, 10.1016/j.ensm.2021.03.026.

[66] M. D. Zhang , J. W. Mu , Y. N. Li , et al., “Propelling Polysulfide Redox by Fe_3_C‐Fen Heterostructure@Nitrogen‐Doped Carbon Framework Towards High‐Efficiency Li‐S Batteries,” Journal of Energy Chemistry 78 (2023): 105–114, 10.1016/j.jechem.2022.11.026.

[67] C. Li , R. Liu , Y. Xiao , F. Cao , and H. Zhang , “Recent Progress of Separators in Lithium‐Sulfur Batteries,” Energy Storage Materials 40 (2021): 439–460, 10.1016/j.ensm.2021.05.034.

[68] J. Wu , T. Ye , Y. Wang , et al., “Understanding the Catalytic Kinetics of Polysulfide Redox Reactions on Transition Metal Compounds in Li–S Batteries,” ACS Nano 16 (2022): 15734–15759, 10.1021/acsnano.2c08581. 36223201

[69] Y. Min , X. Zou , Q. Lu , W. Cai , and Y. Bu , “Advances in the Catalytic Mechanism of Metal Oxides for Lithium–Sulfur Batteries,” Small 21 (2025): 2411794, 10.1002/smll.202411794. 39950501

[70] Z. He , T. Wan , Y. Luo , et al., “Three‐Dimensional Structural Confinement Design of Conductive Metal Oxide for Efficient Sulfur Host in Lithium‐Sulfur Batteries,” Chemical Engineering Journal 448 (2022): 137656, 10.1016/j.cej.2022.137656.

[71] J. Song , L. Yue , Y. Dou , et al., “Island‐Like FeS_2_@TiO_2_ Heterojunction Modified Separator for Enhanced Polysulfide Management in Lithium‐Sulfur Batteries,” Chemical Engineering Journal 511 (2025): 161934, 10.1016/j.cej.2025.161934.

[72] C. Shi , J. Huang , Y. Tang , et al., “A Hierarchical Porous Carbon Aerogel Embedded With Small‐Sized TiO_2_ Nanoparticles for High‐Performance Li‐S Batteries,” Carbon 202 (2023): 59–65, 10.1016/j.carbon.2022.09.086.

[73] Z.‐D. Huang , M.‐T. Yang , J.‐Q. Qi , et al., “Mitigating the Polysulfides “Shuttling” with TiO_2_ Nanowires/Nanosheets Hybrid Modified Separators for Robust Lithium‐Sulfur Batteries,” Chemical Engineering Journal 387 (2020): 124080, 10.1016/j.cej.2020.124080.

[74] Y.‐W. Tian , Y.‐J. Zhang , L. Wu , et al., “Bifunctional Separator With Ultra‐Lightweight MnO_2_ Coating for Highly Stable Lithium–Sulfur Batteries,” ACS Applied Materials & Interfaces 15 (2023): 6877–6887, 10.1021/acsami.2c20461. 36705989

[75] Z. Peng , J. Liu , Z. Wu , R. Liao , J. Zhou , and J. Li , “Carbon Layer Induced the Formation of an Oxygen Vacancy‐Rich γ‐MnO_2_ Porous Network on the Separator for Enhancing Lithium–Sulfur Battery Performance,” Industrial & Engineering Chemistry Research 64 (2025): 10898–10905, 10.1021/acs.iecr.5c01183.

[76] L. Wu , C. Cai , X. Yu , et al., “Scalable 3d Honeycombed Co_3_O_4_ Modified Separators as Polysulfides Barriers for High‐Performance Li–S Batteries,” ACS Applied Materials & Interfaces 14 (2022): 35894–35904, 10.1021/acsami.2c07263. 35881975

[77] X. Wu , J. Li , J. Li , et al., “Two‐Dimensional Layered Co_3_O_4_/CoSe_2_ Heterostructure Modified Separator for High‐Capacity and Long‐Cycle Lithium‐Sulfur Battery,” Journal of Colloid and Interface Science 690 (2025): 137366, 10.1016/j.jcis.2025.137366. 40120377

[78] N. Shi , B. Xi , Z. Feng , et al., “Insight Into different‐Microstructured ZnO/Graphene‐Functionalized Separators Affecting the Performance of Lithium–Sulfur Batteries,” Journal of Materials Chemistry A 7 (2019): 4009–4018, 10.1039/C8TA12409D.

[79] Z. Ouyang , B. Zheng , C. Shi , et al., “Oxygen‐Defect‐Rich ZnV_2_O_4_/ZnO Heterojunction as Multifunctional Separator to Boost Lithium Polysulfide Adsorption and Conversion for Superior Lithium‑Sulfur Batteries,” Journal of Energy Storage 94 (2024): 112405, 10.1016/j.est.2024.112405.

[80] Y. Li , Y. Sun , S. Jia , C. Song , Z. Chen , and Y. Li , “Research Progress on TiO_2_‐Modified Lithium and Lithium‐Sulfur Battery Separator Materials,” Ionics 30 (2024): 3723–3744, 10.1007/s11581-024-05595-1.

[81] J.‐Y. Hwang , H. M. Kim , S. K. Lee , et al., “High‐Energy, High‐Rate, Lithium–Sulfur Batteries: Synergetic Effect of Hollow TiO_2_ ‐Webbed Carbon Nanotubes and a Dual Functional Carbon‐Paper Interlayer,” Advanced Energy Materials 6 (2016): 201670001, 10.1002/aenm.201670001.

[82] Z. Xiao , Z. Yang , L. Wang , et al., “A Lightweight TiO_2_ /Graphene Interlayer, Applied as a Highly Effective Polysulfide Absorbent for Fast, Long‐Life Lithium–Sulfur Batteries,” Advanced Materials 27 (2015): 2891–2898, 10.1002/adma.201405637. 25820906

[83] T. Lei , Y. Xie , X. Wang , S. Miao , J. Xiong , and C. Yan , “TiO_2_ Feather Duster as Effective Polysulfides Restrictor for Enhanced Electrochemical Kinetics in Lithium–Sulfur Batteries,” Small 13 (2017): 1701013, 10.1002/smll.201701013. 28748580

[84] Y. Zhao , M. Liu , W. Lv , et al., “Dense Coating of Li_4_Ti_5_O_12_ and Graphene Mixture on the Separator to Produce Long Cycle Life of Lithium‐Sulfur Battery,” Nano Energy 30 (2016): 1–8, 10.1016/j.nanoen.2016.09.030.

[85] N. Liu , L. Wang , T. Tan , Y. Zhao , and Y. Zhang , “TiO_2_/GO‐Coated Functional Separator to Suppress Polysulfide Migration in Lithium–Sulfur Batteries,” Beilstein Journal of Nanotechnology 10 (2019): 1726–1736, 10.3762/bjnano.10.168. PMC6720060 31501744

[86] Y. Wan , X. Ma , J. Hao , et al., “Titanium‐Based Nanorods/Ketjen Black Modified Separator as Polysulfides Barrier for Lithium Sulfur Cell,” Journal of Alloys and Compounds 842 (2020): 155830, 10.1016/j.jallcom.2020.155830.

[87] P. Chen , Z. Wang , B. Zhang , et al., “Reduced Graphene Oxide/TiO_2_ (B) Nanocomposite‐Modified Separator as an Efficient Inhibitor of Polysulfide Shuttling in Li–S Batteries,” RSC Advances 10 (2020): 4538–4544, 10.1039/C9RA10185C. PMC9049200 35495225

[88] J. Pu , T. Wang , Y. Tan , S. Fan , and P. Xue , “Effect of Heterostructure‐Modified Separator in Lithium–Sulfur Batteries,” Small 19 (2023): 2303266, 10.1002/smll.202303266. 37292047

[89] T. Zhou , W. Lv , J. Li , et al., “Twinborn TiO_2_–TiN Heterostructures Enabling Smooth Trapping–Diffusion–Conversion of Polysulfides Towards Ultralong Life Lithium–Sulfur Batteries,” Energy & Environmental Science 10 (2017): 1694–1703, 10.1039/C7EE01430A.

[90] Q. Zhang , R. Gao , Z. Li , et al., “Manipulating the Conversion Kinetics of Polysulfides by Engineering Oxygen p‐Band of Halloysite for Improved Li‐S Batteries,” Small 18 (2022): 2105661, 10.1002/smll.202105661. 34854559

[91] H. D. Chen , Y. C. Qiu , Z. Y. Cai , et al., “Topological Insulator Heterojunction With Electric Dipole Domain to Boost Polysulfide Conversion in Lithium‐Sulfur Batteries,” Angewandte Chemie‐International Edition 64 (2025): 202423357, 10.1002/anie.202423357. 39743867

[92] K. C. Li , X. Chen , A. Sabbaghi , et al., “Single‐Step Synthesis of Titanium Nitride‐Oxide Composite and AI‐Driven Aging Forecast for Lithium–Sulfur Batteries,” Journal of Materials Chemistry A 12 (2024): 9017–9030, 10.1039/D4TA00234B.

[93] Y. Song , H. Li , J. Li , J. An , J.‐J. Shao , and G. Zhou , “Rationalizing the Impact of Oxygen Vacancy on Polysulfide Conversion Kinetics for Highly Efficient Lithium‐Sulfur Batteries,” Journal of Energy Chemistry 87 (2023): 51–60, 10.1016/j.jechem.2023.08.003.

[94] Z. H. Li , C. Zhou , J. H. Hua , et al., “Engineering Oxygen Vacancies in a Polysulfide‐Blocking Layer With Enhanced Catalytic Ability,” Advanced Materials 32 (2020): 1907444, 10.1002/adma.201907444. 31995271

[95] L. Ma , Y. Zhang , S. Zhang , et al., “Integrating Energy Band Alignment and Oxygen Vacancies Engineering of TiO_2_ Anatase/Rutile Homojunction for Kinetics‐Enhanced Li–S Batteries,” Advanced Functional Materials 33 (2023): 2305788, 10.1002/adfm.202305788.

[96] X. Du , D. Ma , Y. Zhang , et al., “Electrospun TiO_2_ Nanofibers Featuring Surface Oxygen Vacancies as a Multifunctional Interlayer for High‐Performance Lithium–Sulfur Batteries in a Wide Temperature Range,” Inorganic Chemistry 62 (2023): 5134–5144, 10.1021/acs.inorgchem.2c04417. 36960495

[97] Z. Zhao , X. Duan , L. Zhang , et al., “Elevated Electrochemical Performances Enabled by a Core–Shell Titanium Hydride Coated Separator in Lithium–Sulphur Batteries,” RSC Advances 11 (2021): 30755–30762, 10.1039/d1ra04281e. PMC9041292 35498953

[98] X. Fan , H. Liu , H. Wang , W. Hu , X. Tian , and L. Pang , “Understanding the Synergistic Improving Adsorbed and Catalytic Activity of Polysulfide by Design of Oxygen Vacancies and Li^+^ Lithiation/Delithiation Behavior in Titanium Oxide Separator,” Electrochimica Acta 485 (2024): 144142, 10.1016/j.electacta.2024.144142.

[99] A. Chen , W. Liu , J. Yan , and K. Liu , “A Novel Separator Modified by Titanium Dioxide Nanotubes/Carbon Nanotubes Composite for High Performance Lithium‐Sulfur Batteries,” Functional Materials Letters 12 (2019): 1950016, 10.1142/s1793604719500164.

[100] A. Ansari Hamedani , B. Cetiner , B. Yarar Kaplan , S. Alkan Gürsel , and A. Yürüm , “Nanofibrous Interlayers With Ultrafine Decoration of Titanium Oxide to Intercept Lithium Polysulfides in Lithium‐Sulfur Batteries,” Chemphyschem 26 (2025): 202401136, 10.1002/cphc.202401136. 40589050

[101] J. Wang , X. Zhang , J. Liu , et al., “High‐Spin Cobalt Enables Strong Metal‐Sulfur Orbital Hybridization for Accelerated Polysulfide Conversion in Lithium‐Sulfur Batteries,” Advanced Materials 37 (2025): 2502075, 10.1002/adma.202502075. 40384160

[102] S. Liang , S. Chauque , M. Ricci , and R. P. Zaccaria , “Enhancing Lithium‐Sulfur Battery Performance through Electronic/Ionic Co‐Conductive Multi‐Walled Carbon Nanotubes/Lithium Lanthanum Titanium Oxide Separator Modification,” Materials Today Energy 40 (2024): 101497, 10.1016/j.mtener.2024.101497.

[103] M. Qian , Y. Tang , X. Li , Y. Zhang , W. Jiang , and L. Liu , “Regulating the Proximity of D‐P Band Center in TiP2O7 by the Crystalline‐Amorphous Heterointerface to Boost Adsorption‐Catalysis for LiPSs in Li‐S Batteries,” Journal of Energy Chemistry 108 (2025): 458–467, 10.1016/j.jechem.2025.04.030.

[104] M. Zlámalová , B. Pitňa Lásková , M. Vinarčíková , M. Zukalová , and L. Kavan , “Inherent Electrochemical Activity of TiO_2_ (Anatase, Rutile) Enhances the Charge Capacity of Cathodes of Lithium‐Sulfur Batteries,” Journal of Solid State Electrochemistry 26 (2022): 639–647, 10.1007/s10008-022-05115-z.

[105] Y. Zhong , F. Lin , M. Wang , et al., “Metal Organic Framework Derivative Improving Lithium Metal Anode Cycling,” Advanced Functional Materials 30 (2020): 1907579, 10.1002/adfm.201907579.

[106] R. Li , J. Li , X. Wang , et al., “Surface Design for High Ion Flux Separator in Lithium‐Sulfur Batteries,” Journal of Colloid and Interface Science 654 (2024): 13–24, 10.1016/j.jcis.2023.10.018. 37832231

[107] Y. Liu , Z. Wei , B. Zhong , et al., “O‐, N‐Coordinated Single Mn Atoms Accelerating Polysulfides Transformation in Lithium‐Sulfur Batteries,” Energy Storage Materials 35 (2021): 12–18, 10.1016/j.ensm.2020.11.011.

[108] X. Song , G. Chen , S. Wang , et al., “Self‐Assembled Close‐Packed MnO_2_ Nanoparticles Anchored on a Polyethylene Separator for Lithium–Sulfur Batteries,” ACS Applied Materials & Interfaces 10 (2018): 26274–26282, 10.1021/acsami.8b07663. 30027731

[109] X. Wang , L. Yang , Y. Wang , et al., “Novel Functional Separator with Self‐Assembled MnO_2_ Layer via a Simple and Fast Method in Lithium‐Sulfur Battery,” Journal of Colloid and Interface Science 606 (2022): 666–676, 10.1016/j.jcis.2021.08.062. 34418754

[110] J. Luo and J. Zheng , “Mitigating the Polysulfide Diffusion via Using Functional Manganese Dioxide Nanofiber Interlayer for High Electrochemical Activity,” Ionics 27 (2021): 2495–2499, 10.1007/s11581-021-04047-4.

[111] D. W. Kim , C. Senthil , S. M. Jung , et al., “Selective Ion Transport of Catalytic Hybrid Aerofilm Interlayer for Long‐Stable Li‐S Batteries,” Energy Storage Materials 47 (2022): 472–481, 10.1016/j.ensm.2022.02.036.

[112] Y. Yu , X. Zhang , L. He , et al., “Manganese Oxide Nanosheets With Mixed Valence States as a Separator Coating for Lithium–Sulfur Batteries,” ACS Applied Energy Materials 6 (2023): 7298–7304, 10.1021/acsaem.3c01137.

[113] Z. Ma , Z. Qi , G. Song , et al., “α‐MnO_2_ /RuO_2_ Heterostructure‐Modified Polypropylene Separator for High‐Performance Lithium–Sulfur Battery,” Advanced Functional Materials 34 (2024): 2403101, 10.1002/adfm.202403101.

[114] Y. Si , J. Zhang , Q. Huang , et al., “Manganese–Nickel Bimetallic Oxide Electrocatalyzing Redox Reactions of Lithium Polysulfides in Lithium–Sulfur Batteries,” Sustainable Energy & Fuels 6 (2022): 1426–1435, 10.1039/D1SE01987B.

[115] Z. Hao , J. Chen , X. Lu , et al., “Precisely Visit the Performance Modulation of Functionalized Separator in Li‐S Batteries via Consecutive Multiscale Analysis,” Energy Storage Materials 49 (2022): 85–92, 10.1016/j.ensm.2022.04.003.

[116] Y. Wang , X. Guo , C. Chen , et al., “Alleviating the Shuttle Effect via Bifunctional MnFe_2_O_4_/Ab Modified Separator for High Performance Lithium Sulfur Battery,” Electrochimica Acta 354 (2020): 136704, 10.1016/j.electacta.2020.136704.

[117] L. Zhou , H. Li , Y. Zhang , et al., “Enhanced Sulfur Utilization in Lithium‐Sulfur Batteries by Hybrid Modified Separators,” Materials Today Communications 26 (2021): 102133, 10.1016/j.mtcomm.2021.102133.

[118] M. Yao , R. Wang , Z. Zhao , Y. Liu , Z. Niu , and J. Chen , “A Flexible All‐in‐One Lithium‐Sulfur Battery,” ACS Nano 12 (2018): 12503–12511, 10.1021/acsnano.8b06936. 30507142

[119] L. Bai , Y. Ren , Q. Zheng , J. Nie , and B. Du , “Modification of Polypropylene Separator with Nickel and Manganese Oxide‐Decorated Carbon Nanotubes Toward High‐Performance Lithium‐Sulfur Batteries,” Journal of Power Sources 633 (2025): 236353, 10.1016/j.jpowsour.2025.236353.

[120] F. Teng , L. Zhu , J. Ma , et al., “Nanostructured MnFe_2_O_4_/MnO Heterojunctions as Highly‐Efficient Bi‐Functional Catalyst for Complementary Conversions of Polysulfides Toward Robust Lithium‐Sulfur Batteries,” Chemical Engineering Journal 505 (2025): 159216, 10.1016/j.cej.2025.159216.

[121] L. Gao , Y. Cao , J. Wang , H. Ren , J. Wang , and J. Huang , “Construction of Polypyrrole Coated Hollow Cobalt Manganate Nanocages as an Effective Sulfur Host for Lithium‐Sulfur Batteries,” Ceramics International 46 (2020): 18224–18233, 10.1016/j.ceramint.2020.04.145.

[122] H. Raza , J. Cheng , S. Kandasamy , et al., “Manganese‐Incorporated Single‐Phase High‐Entropy Oxide Modified Separator Enabled High Performance of Lithium‐Sulfur Batteries at High Sulfur Loading,” Energy & Environmental Materials 8 (2025): 70058, 10.1002/eem2.70058.

[123] X. Wang , P. Lin , C. Wu , et al., “Superior‐Performance Lithium–Sulfur Batteries: A face‐Centered‐Cubic‐Structure High‐Entropy Alloy Improves the Bidirectional Catalytic Conversion of Polysulfides/Sulfides,” Inorganic Chemistry Frontiers 11 (2024): 6425–6437, 10.1039/D4QI01434K.

[124] T. Zhang , D. Luo , H. Xiao , et al., “A Transmetalation Synthetic Strategy to Engineer Atomically Dispersed MnN_2_O_2_ Electrocatalytic Centers Driving High‐Performance Li‐S Battery,” Small 19 (2023): 2302249, 10.1002/smll.202302249. 37226368

[125] S. D. Hirt , M. Opitz , H. Kappl , et al., “Attenuating the Polysulfide Shuttle Mechanism by Separator Coating,” Chemphyschem 25 (2024): 202300858, 10.1002/cphc.202300858. 38483867

[126] L. Wu , J. Hu , X. Yang , et al., “Synergistic Effect of Adsorption and Electrocatalysis of CoO/NiO Heterostructure Nanosheet Assembled Nanocages for High‐Performance Lithium–Sulfur Batteries,” Journal of Materials Chemistry A 10 (2022): 23811–23822, 10.1039/D2TA06876A.

[127] N. Wang , B. Chen , K. Qin , et al., “Rational Design of Co_9_S_8_/CoO Heterostructures With Well‐Defined Interfaces for Lithium Sulfur Batteries: A Study of Synergistic Adsorption‐Electrocatalysis Function,” Nano Energy 60 (2019): 332–339, 10.1016/j.nanoen.2019.03.060.

[128] K. Zhang , L. Jin , X. Qian , Q. Hao , S. Zhao , and B. Li , “Double Hollow Spherical SnO_2_@Co_3_O_4_ Applied to Separator for Efficient Catalysis of Polysulfides in Lithium‐Sulfur Batteries,” Journal of Industrial and Engineering Chemistry 137 (2024): 260–268, 10.1016/j.jiec.2024.03.013.

[129] M. Liu , L. Wan , P. Su , et al., “Co/Co_3_O_4_@NC‐CNTs Modified Separator of Li‐S Battery Achieving the Synergistic Effect of Adsorption‐Directional Migration‐Catalysis via Built‐in Electric Field,” Journal of Colloid and Interface Science 682 (2025): 436–445, 10.1016/j.jcis.2024.11.211. 39631315

[130] Y. Jiang , M. Du , P. Geng , B. Sun , R. Zhu , and H. Pang , “CoO/MoO_3_@Nitrogen‐Doped Carbon Hollow Heterostructures for Efficient Polysulfide Immobilization and Enhanced Ion Transport in Lithium‐Sulfur Batteries,” Journal of Colloid and Interface Science 664 (2024): 617–625, 10.1016/j.jcis.2024.03.015. 38490037

[131] B. Fan , W. Chen , K. Li , et al., “Synergistic Adsorption and Catalytic Effects of Ti_3_C_2_T_x_/CoO/MoO_3_ Composite on Lithium Polysulfides for High‐Performance Lithium–Sulfur Batteries,” Interdisciplinary Materials 3 (2024): 726–737, 10.1002/idm2.12178.

[132] R. Li , D. Rao , J. Zhou , et al., “Amorphization‐Induced Surface Electronic States Modulation of Cobaltous Oxide Nanosheets for Lithium‐Sulfur Batteries,” Nature Communications 12 (2021): 3102, 10.1038/s41467-021-23349-9. PMC8149689 34035271

[133] R. Liu , W. Yin , Y. Chen , et al., “Facile Utilization of Spent LiCoO_2_ in Separator Decoration of Lithium‐Sulfur Batteries,” Industrial & Engineering Chemistry Research 59 (2020): 17911–17917, 10.1021/acs.iecr.0c03490.

[134] L.‐L. Gu , C. Wang , S.‐Y. Qiu , et al., “Cobalt‐Iron Oxide Nanotubes Decorated with Polyaniline as Advanced Cathode Hosts for Li‐S Batteries,” Electrochimica Acta 390 (2021): 138873, 10.1016/j.electacta.2021.138873.

[135] Z. Wang , Y. Cheng , S. Wang , et al., “Promoting Polysulfide Conversions via Cobalt Single‐Atom Catalyst for Fast and Durable Lithium‐Sulfur Batteries,” Nano Research 16 (2023): 9335–9343, 10.1007/s12274-023-5557-6.

[136] L. Zhang , F. Wan , H. Cao , L. Liu , Y. Wang , and Z. Niu , “Integration of Binary Active Sites: Co_3_V_2_O_8_ as Polysulfide Traps and Catalysts for Lithium‐Sulfur Battery With Superior Cycling Stability,” Small 16 (2020): 1907153, 10.1002/smll.201907153. 32285595

[137] H. Li , Y. Wang , H. Chen , B. Niu , W. Zhang , and D. Wu , “Synergistic Mediation of Polysulfide Immobilization and Conversion by a Catalytic and Dual‐Adsorptive System for High Performance Lithium‐Sulfur Batteries,” Chemical Engineering Journal 406 (2021): 126802, 10.1016/j.cej.2020.126802.

[138] M. Zhang , H. Xiao , Y. Liu , et al., “Interfacial Build‐in Electric Field Unlocking Hetero‐Architectured CoO‐Co_3_O_4_ Nanotubes Toward High‐Performance Li‐S Batteries,” Advanced Functional Materials 18 (2026): 20708, 10.1002/adfm.202520708.

[139] W. Ma , Z. Shao , J. Yao , et al., “Mott‐Schottky Electrocatalyst Selectively Mediates the Sulfur Species Conversion in Lithium‐Sulfur Batteries,” Journal of Colloid and Interface Science 631 (2023): 114–124, 10.1016/j.jcis.2022.11.041. 36399804

[140] Y.‐M. Lu , J.‐L. Yang , S.‐X. Zhao , X.‐T. Zeng , L.‐Q. Yu , and C. Huang , “Double Role of CoO Co_4_N Hetero‐Nanocages As Sulfur Host For Lithium‐Sulfur Batteries,” Journal of Materiomics 7 (2021): 1301–1308, 10.1016/j.jmat.2021.02.019.

[141] Z. Bai , Z. Wang , T. Wang , et al., “Cation‐Vacancy Engineering Modulated Perovskite Oxide for Boosting Electrocatalytic Conversion of Polysulfides,” Advanced Functional Materials 35 (2025): 2419105, 10.1002/adfm.202419105.

[142] W. Zhou , M. Chen , D. Zhao , et al., “Engineering Spin State in Spinel Co_3_O_4_ Nanosheets by V‐Doping for Bidirectional Catalysis of Polysulfides in Lithium–Sulfur Batteries,” Advanced Functional Materials 34 (2024): 2402114, 10.1002/adfm.202402114.

[143] A. Gao , Q. Wu , H. Chen , et al., “Oxygen Vacancies‐Enriched CeO_2_‐CoxOy Heterostructures Facilitate the “Trapping‐Conversion” Process of Lithium Polysulfides for High‐Performance Li‐S Batteries,” Chemical Engineering Journal 512 (2025): 162145, 10.1016/j.cej.2025.162145.

[144] L. Jin , Z. Fu , X. Qian , et al., “Catalytic Co‐N‐C Hollow Nanocages as Separator Coating Layer for Lithium‐Sulfur Batteries,” Microporous and Mesoporous Materials 316 (2021): 110927, 10.1016/j.micromeso.2021.110927.

[145] Y. C. Jeong , J. H. Kim , S. H. Kwon , et al., “Rational Design of Exfoliated 1T MoS2@CNT‐based Bifunctional Separators for Lithium Sulfur Batteries,” Journal of Materials Chemistry A 5 (2017): 23909–23918, 10.1039/C7TA08153G.

[146] S. Zhao , Q. Hao , X. Qian , L. Jin , B. Li , and H. Xu , “Application of Y‐Zn‐MOF Derived Y_2_O_3_/ZnO@C in Modification of Lithium‐Sulfur Battery Separator,” Journal of Energy Storage 101 (2024): 113833, 10.1016/j.est.2024.113833.

[147] S. Pi , X. Xu , W. Lei , et al., “Multi‐Active Sites Synergistic Surface‐Functionalized Carbon Nanotubes Modified Separators Facilitate the Application of High‐Performance Lithium‐Sulfur Batteries,” Journal of Power Sources 659 (2025): 238359, 10.1016/j.jpowsour.2025.238359.

[148] R. Bo , M. Taheri , B. Liu , et al., “Hierarchical Metal‐Organic Framework Films with Controllable Meso/Macroporosity,” Advanced Science 7 (2020): 2002368, 10.1002/advs.202002368. PMC7740079 33344131

[149] S. Wang , F. Gao , R. Ma , et al., “ZnO Nanoparticles Anchored on a N‐Doped Graphene‐Coated Separator for High Performance Lithium/Sulfur Batteries,” Metals 8 (2018): 755, 10.3390/met8100755.

[150] F. Yin , J. Ren , Y. Zhang , T. Tan , and Z. Chen , “A Ppy/Zno Functional Interlayer to Enhance Electrochemical Performance of Lithium/Sulfur Batteries,” Nanoscale Research Letters 13 (2018): 307, 10.1186/s11671-018-2724-x. PMC6170249 30284111

[151] J. Zou , Y. Cao , B. Zhang , and W. Wang , “Suppressing the Shuttle Effect in Lithium‐Sulphur Batteries by Defective Single‐Walled ZnO Nanotube: A DFT Study,” The Canadian Journal of Chemical Engineering 101 (2023): 347–353, 10.1002/cjce.24357.

[152] S. H. Li , X. H. Xia , Y. D. Wang , X. L. Wang , and J. P. Tu , “Reconstruction of Multidimensional Carbon Hosts With Combined 0D, 1D and 2D Networks for Enhanced Lithium‐Sulfur Batteries,” Journal of Power Sources 342 (2017): 224–230, 10.1016/j.jpowsour.2016.12.060.

[153] H. Han , T. Wang , Y. Zhang , A. Nurpeissova , and Z. Bakenov , “Three‐Dimensionally Ordered Macroporous ZnO Framework as Dual‐Functional Sulfur Host for High‐Efficiency Lithium–Sulfur Batteries,” Nanomaterials 10 (2020): 2267, 10.3390/nano10112267. PMC7697050 33207623

[154] X. Qian , J. Cheng , L. Jin , Y. Wang , B. Huang , and J. Chen , “ZIF‐8/Ketjen Black Derived ZnO/N/Kb Composite for Separator Modification of Lithium Sulfur Batteries,” Colloids and Surfaces A: Physicochemical and Engineering Aspects 648 (2022): 129036, 10.1016/j.colsurfa.2022.129036.

[155] W. Zhang , D. Hong , Z. Su , et al., “Tailored Zno‐Zns Heterostructure Enables a Rational Balancing of Strong Adsorption and High Catalytic Activity of Polysulfides for Li‐S Batteries,” Energy Storage Materials 53 (2022): 404–414, 10.1016/j.ensm.2022.09.018.

[156] Q. Hao , X. Qian , L. Jin , et al., “Application of ZIF‐67/ZIF‐8 Derived Co_3_O_4_/ZnO Heterojunction in Lithium‐Sulfur Battery Separators,” Journal of Alloys and Compounds 967 (2023): 171605, 10.1016/j.jallcom.2023.171605.

[157] C. Zhang , L. Pan , H. Liu , et al., “Construction of Porous Hetero Structured Materials Defluorinated Carbon Nanotubes@SiO_2_‐ZnO Modified Separator for Safety and High Performance Lithium‑Sulfur Batteries,” Journal of Energy Storage 95 (2024): 112528, 10.1016/j.est.2024.112528.

[158] S. Blanga , R. Yemini , E. Teblum , M. Tsubery , S. Taragin , and M. Noked , “Improving Li Anode Reversibility in Li–S Batteries by ZnO Coated Separators Using Atomic Layer Deposition,” Journal of The Electrochemical Society 169 (2022): 110512, 10.1149/1945-7111/ac9dec.

[159] X. W. Chang , X. Zeng , Y. Wen , et al., “Thermal Conductive Aramid Nanofiber/Surface‐Decorated Alumina Microsphere Composite Separator,” Polymer 296 (2024): 126793, 10.1016/j.polymer.2024.126793.

[160] S. L. Hong , X. Tang , Z. Sun , and F. Li , “Self‐Supporting Porous CoS_2_/RGO Sulfur Host Prepared by Bottom‐Up Assembly for Lithium‐Sulfur Batteries,” Journal of Alloys and Compounds 749 (2018): 586–593, 10.1016/j.jallcom.2018.03.331.

[161] X. Men , T. Deng , J. Che , and J. Wang , “Accelerating Sulfur Conversion Kinetics via CoS_2_–MgS Heterostructure for Lithium Sulfur Batteries,” Journal of Materials Chemistry A 12 (2024): 31972–31981, 10.1039/D4TA06086E.

[162] F. Li , X. Qian , and L. Jin , “MOF‐Derived MnS/N–C@CNT Composites as Separator Coating Materials for Long‐Cycling Li–S Batteries,” ACS Sustainable Chemistry & Engineering 9 (2021): 15469–15477, 10.1021/acssuschemeng.1c04636.

[163] L. Sun , C. Yue , J. Zhao , et al., “CoS_2_‐MoS_2_‐CNT Composite Modified Separator With Enhanced LiPS Adsorption Capacity for Lithium‐Sulfur Battery,” Journal of Alloys and Compounds 972 (2024): 172733, 10.1016/j.jallcom.2023.172733.

[164] F. Xie , M. Xiong , J. Liu , et al., “A Multi‐Functional Separator for Li‐S Batteries: WS_2_@C Nanoflowers Catalyze the Rapid Recycling of Lithium Polysulfides by Polar Attraction,” ChemElectroChem 9 (2022): 202200474, 10.1002/celc.202200474.

[165] M. A. Al‐Tahan , Y. Dong , A. E. Shrshr , et al., “Enormous‐Sulfur‐Content Cathode and Excellent Electrochemical Performance of Li‐S Battery Accouched by Surface Engineering of Ni‐Doped WS_2_@RGO Nanohybrid as a Modified Separator,” Journal of Colloid and Interface Science 609 (2022): 235–248, 10.1016/j.jcis.2021.12.035. 34906909

[166] J. Chen , T. Sun , J. Liu , et al., “Recent Advances on Transition Metal Sulfides for Key Components of Lithium–Sulfur Batteries,” Advanced Science 12 (2025): 12191, 10.1002/advs.202512191. PMC12561350 40946194

[167] K. Zhang , F. Chen , H. Pan , et al., “Study on the Effect of Transition Metal Sulfide in Lithium–Sulfur Battery,” Inorganic Chemistry Frontiers 6 (2019): 477–481, 10.1039/C8QI01193A.

[168] Y. Lin , Z. Ouyang , S. He , et al., “An Individual Sandwich Hybrid Nanostructure of Cobalt Disulfide In‐Situ Grown on N‐Doped Carbon Layer Wrapped on Multi‐Walled Carbon Nanotubes for High‐Efficiency Lithium Sulfur Batteries,” Journal of Colloid and Interface Science 610 (2022): 560–572, 10.1016/j.jcis.2021.11.102. 34838317

[169] W. Zhang , K. Zhao , Q. Jin , et al., “CoS_2_‐NC@CNTs Hierarchical Nanostructures for Efficient Polysulfide Regulation in Lithium‐Sulfur Batteries,” Electrochimica Acta 430 (2022): 141104, 10.1016/j.electacta.2022.141104.

[170] Y. Li , J. Li , J. Yuan , et al., “3D CoS_2_/RGO Aerogel as Trapping‐Catalyst Sulfur Host to Promote Polysulfide Conversion for Stable Li‐S Batteries,” Journal of Alloys and Compounds 873 (2021): 159780, 10.1016/j.jallcom.2021.159780.

[171] R. Bi , J. Zhao , M. Yang , J. Wang , R. Yu , and D. Wang , “Multifunctional Separator Modified With Catalytic Multishelled Structural CoS_2_ Enables a Stable Lithium–Sulfur Battery,” Inorganic Chemistry Frontiers 11 (2024): 8837–8846, 10.1039/D4QI02340D.

[172] S.‐J. Jiang , C.‐X. Wu , R. Liu , J. Wang , Y.‐S. Xu , and F.‐F. Cao , “Multifunctional Interlayer Engineering for Silkworm Excrement‐Derived Porous Carbon Enabling High‐Energy Lithium Sulfur Batteries,” Chemsuschem 17 (2024): 202301110, 10.1002/cssc.202301110. 37653603

[173] L. Wu , Y. Zhao , Y. Dai , S. Gao , B. Liao , and H. Pang , “CoS_2_@montmorillonite as an Efficient Separator Coating for High‐Performance Lithium–Sulfur Batteries,” Inorganic Chemistry Frontiers 9 (2022): 3335–3347, 10.1039/D2QI00638C.

[174] P. Wan , H. Xu , R. Cao , et al., “A Hierarchically Porous CoS_2_‐NiS_2_ Heterojunction for High‐Performance Lithium Sulfur Batteries,” Journal of Colloid and Interface Science 687 (2025): 599–606, 10.1016/j.jcis.2025.02.084. 39978265

[175] Y. Ren , Y. Ma , B. Wang , et al., “Furnishing Continuous Efficient Bidirectional Polysulfide Conversion for Long‐Life and High‐Loading Lithium–Sulfur Batteries via the Built‐In Electric Field,” Small 19 (2023): 2300065, 10.1002/smll.202300065. 37147776

[176] S. Ji , Z. Wang , M. Wang , et al., “Highly Dispersed FeS_2_‐CoS_2_ Heterostructural Nanospheres with Modulated Interfacial Electronic Structure for Adsorption‐Reinforced, Kinetics‐Accelerated, and Dendrite‐Inhibited Lithium‐Sulfur Batteries,” Surfaces and Interfaces 58 (2025): 105863, 10.1016/j.surfin.2025.105863.

[177] Y. Tian , Z. Wei , F. Li , et al., “Enhanced Multiple Anchoring and Catalytic Conversion of Polysulfides by SnO_2_‐Decorated MoS_2_ Hollow Microspheres for High‐Performance Lithium‐Sulfur Batteries,” Journal of Materials Science & Technology 100 (2022): 216–223, 10.1016/j.jmst.2021.06.002.

[178] G. Zhang , X. Chen , X. Yu , et al., “Interfacial Engineering Design Induces Enriched‐Defects Expediting Catalytic Conversion Kinetics of Polysulfides,” Journal of Materials Chemistry A 12 (2024): 8927–8939, 10.1039/D3TA07886H.

[179] Y. Li , D. Lei , T. Jiang , et al., “P‐Doped Co9S8 Nanoparticles Embedded on 3D Spongy Carbon‐Sheets as Electrochemical Catalyst for Lithium‐Sulfur Batteries,” Chemical Engineering Journal 426 (2021): 131798, 10.1016/j.cej.2021.131798.

[180] J. Wang , W. Cai , X. Mu , et al., “Construction of Multifunctional and Flame Retardant Separator towards Stable Lithium‐Sulfur Batteries With High Safety,” Chemical Engineering Journal 416 (2021): 129087, 10.1016/j.cej.2021.129087.

[181] J. Liu , Z. Qiao , Q. Xie , D.‐L. Peng , and R.‐J. Xie , “Phosphorus‐Doped Metal–Organic Framework‐Derived CoS_2_ Nanoboxes With Improved Adsorption‐Catalysis Effect for Li–S Batteries,” ACS Applied Materials & Interfaces 13 (2021): 15226–15236, 10.1021/acsami.1c00494. 33769028

[182] J. Ren and Q. Zhao , “Preparation of a Lithium‐Sulfur Battery Diaphragm Catalyst and Its Battery Performance,” RSC Advances 14 (2024): 36471–36487, 10.1039/d4ra06366j. PMC11565165 39553277

[183] H. Zhu , Y. Xiao , J. Zhao , et al., “Copper‐Doping‐Induced Lattice Distortion in CoS_2_ Nanocages for Enhanced Polysulfide Conversion in Advanced Li–S Batteries,” ACS Sustainable Chemistry & Engineering 13 (2025): 18703–18712, 10.1021/acssuschemeng.5c07370.

[184] S. Deng , Y. Lv , Y. Zhao , et al., “Exquisitely Constructing Hierarchical Carbon Nanoarchitectures Decorated With Sulfides for High‐Performance Li–S Batteries,” Dalton Transactions 53 (2024): 4753–4763, 10.1039/D3DT04163H. 38363131

[185] X. Xu , H. Xu , R. Wang , et al., “Enhanced Adsorption and Catalytic Conversion of Lithium Polysulfides Enabled by CoS_2_‐WS_2_/Gnc Heterostructure‐Modified Separator,” Applied Surface Science 717 (2026): 164736, 10.1016/j.apsusc.2025.164736.

[186] B. Zhang , J. Ma , M. Cui , Y. Zhao , and S. Wei , “A Rational Design of a CoS_2_‐CoSe_2_ Heterostructure for the Catalytic Conversion of Polysulfides in Lithium‐Sulfur Batteries,” Materials 16 (2023): 3992, 10.3390/ma16113992. PMC10254419 37297125

[187] J. Feng , Y. Li , J. Yuan , et al., “Energy‐Saving Synthesis of Functional CoS_2_/rGO Interlayer With Enhanced Conversion Kinetics for High‐Performance Lithium‐Sulfur Batteries,” Frontiers in Chemistry 9 (2021): 830485, 10.3389/fchem.2021.830485. PMC8867214 35223779

[188] S. Tian , Q. Zeng , G. Liu , et al., “Multi‐Dimensional Composite Frame as Bifunctional Catalytic Medium for Ultra‐Fast Charging Lithium–Sulfur Battery,” Nano‐Micro Letters 14 (2022): 196, 10.1007/s40820-022-00941-2. PMC9537413 36201063

[189] C. Yang , Y. Li , W. Peng , F. Zhang , and X. Fan , “In Situ N‐Doped CoS_2_ Anchored on Mxene Toward an Efficient Bifunctional Catalyst for Enhanced Lithium‐Sulfur Batteries,” Chemical Engineering Journal 427 (2022): 131792, 10.1016/j.cej.2021.131792.

[190] J. Wang , Y. Cao , Z. Wang , et al., “Superior and Safer Lithium Sulfur Batteries Realized by Robust Polysulfides‐Retarding Dam With High Flame Retardance,” Journal of Energy Chemistry 89 (2024): 471–486, 10.1016/j.jechem.2023.09.047.

[191] N. Wu , J. Wang , C. Liao , et al., “A Flame Retardant Separator Modified by MOFs‐Derived Hybrid for Safe and Efficient Li‐S Batteries,” Journal of Energy Chemistry 64 (2022): 372–384, 10.1016/j.jechem.2021.05.001.

[192] Y. Z. Wu , Y. Dai , S. Gao , B. Liao , and H. Pang , “CoS_2_@montmorillonite as an Efficient Separator Coating for High‐Performance Lithium–Sulfur Batteries,” Inorganic Chemistry Frontiers 13 (2022): 3335–3347, 10.1039/d2qi00368c.

[193] Q. Li , H. Liu , B. Jin , et al., “Anchoring Polysulfides via a CoS_2_/NC@1T MoS_2_ Modified Separator for High‐Performance Lithium–Sulfur Batteries,” Inorganic Chemistry Frontiers 10 (2023): 959–971, 10.1039/d2qi01884e.

[194] J. Feng , Y. Li , J. Yuan , et al., “Energy‐Saving Synthesis of Functional CoS_2_/rGO Interlayer With Enhanced Conversion Kinetics for High‐Performance Lithium‐Sulfur Batteries,” Frontiers in Chemistry 9 (2022): 830485, 10.3389/fchem.2021.830485. PMC8867214 35223779

[195] X. Men , T. Deng , X. Li , L. Huang , and J. Wang , “Electrospun Carbon Nanofibers Loaded With Sulfur Vacancy CoS_2_ as Separator Coating to Accelerate Sulfur Conversion in Lithium‐Sulfur Batteries,” Journal of Colloid and Interface Science 678 (2025): 345–354, 10.1016/j.jcis.2024.08.192. 39208762

[196] J. Ren and Q. Zhao , “Preparation of a Lithium–Sulfur Battery Diaphragm Catalyst and Its Battery Performance,” RSC Advances 49 (2024): 36471–36487, 10.1039/d4ra06720g. PMC11565165 39553277

[197] F. Ma , P. Hu , T. Wang , et al., “Yolk@Shell Structured MnS@Nitrogen‐Doped Carbon as a Sulfur Host and Polysulfide Conversion Booster for Lithium/Sodium Sulfur Batteries,” ACS Applied Energy Materials 4 (2021): 3487–3494, 10.1021/acsaem.0c03235.

[198] Y. Zhu , Y. Zuo , X. Jiao , et al., “Selective Sulfur Conversion With Surface Engineering of Electrocatalysts in a Lithium–Sulfur Battery,” Carbon Energy 5 (2023): 249, 10.1002/cey2.249.

[199] Y. Li , Y. Hao , U. Ali , et al., “Bimetallic Sulfides Heterostructure Efficient Adsorption and Catalytic of Polysulfides for High‐Performance Lithium‐Sulfur Batteries,” Chemical Engineering Journal 474 (2023): 145961, 10.1016/j.cej.2023.145961.

[200] Z. Chen , J. Wu , M. Jiang , et al., “Reinforcing the Adsorption and Conversion of Polysulfides in Li−S Battery by Incorporating Molybdenum Into MnS/MnO Nanorods,” Chemistry – A European Journal 30 (2024): 202303507, 10.1002/chem.202303507. 37994505

[201] X. Wang , X. Zhao , C. Ma , et al., “Electrospun Carbon Nanofibers With MnS Sulfiphilic Sites as Efficient Polysulfide Barriers for High‐Performance Wide‐Temperature‐Range Li–S Batteries,” Journal of Materials Chemistry A 8 (2020): 1212–1220, 10.1039/C9TA12137D.

[202] F. Li , X. Qian , and L. Jin , “MOF‐Derived MnS/N–C@CNT Composites as Separator Coating Materials for Long‐Cycling Li–S Batteries,” ACS Sustainable Chemistry & Engineering 9 (2021): 15469–15477, 10.1021/acssuschemeng.

[203] Z. Cheng , Y. Chen , Y. Yang , et al., “Lithium–Sulfur Batteries: Metallic MoS_2_ Nanoflowers Decorated Graphene Nanosheet Catalytically Boosts the Volumetric Capacity and Cycle Life of Lithium–Sulfur Batteries (Adv. Energy Mater. 12/2021),” Advanced Energy Materials 11 (2021): 2170048, 10.1002/aenm.202170048.

[204] L. Liu , M. Yan , X. Zhao , and H. Pan , “A Novel Pathway for Sustained Sulfides Conversion via Electrocatalyst‐Modified Separator in Lithium‐Sulfur Batteries,” Nano Energy 130 (2024): 110122, 10.1016/j.nanoen.2024.110122.

[205] L. Jin , B. Huang , X. Qian , et al., “Hollow Bimetallic Sulfide Composite Coated Separator for Hindering the Shuttle of Polysulfides in Li‐S Batteries,” Journal of Alloys and Compounds 926 (2022): 166515, 10.1016/j.jallcom.2022.166515.

[206] Y. Li , C. Xu , D. Li , Y. Zhang , B. Liu , and P. Huo , “ZnS/MoS_2_ Heterostructures on Mxene: A Strategy for Improved Polysulfide Adsorption and Conversion in Li‐S Batteries,” Chemical Engineering Journal 502 (2024): 158151, 10.1016/j.cej.2024.158151.

[207] J. W. González , E. Flórez , and J. D. Correa , “MoS_2_ 2D‐Polymorphs as Li‐/Na‐Ion Batteries: 1T' Vs 2h Phases,” Journal of Molecular Liquids 396 (2024): 123904, 10.1016/j.molliq.2023.123904.

[208] R. Li , Z. Bai , W. Hou , et al., “Enhancing Electrochemical Conversion of Lithium Polysulfide by 1T‐Rich MoSe_2_ Nanosheets for High Performance Lithium–Sulfur Batteries,” Chinese Chemical Letters 34 (2023): 108263, 10.1016/j.cclet.2023.108263.

[209] Y. Gao , Y. Bai , R. Sun , et al., “Advanced Separator Enabled by Sulfur Defect Engineering for High‐Performance Lithium–Sulfur Batteries,” Industrial & Engineering Chemistry Research 61 (2022): 6957–6966, 10.1021/acs.iecr.2c00634.

[210] Z. Wu , W. He , R. Xie , et al., “Nitrogen‐Doped Graphene Uniformly Loaded With Large Interlayer Spacing MoS_2_ Nanoflowers for Enhanced Lithium–Sulfur Battery Performance,” Molecules 29 (2024): 4968, 10.3390/molecules29204968. PMC11509959 39459336

[211] J. Wang , Y. Zhu , N. Wu , Y. Kan , and Y. Hu , “Designing Thermotolerant and Flame‐Resistant Pan‐Based Separator via Surface Engineering with Heteroatoms Doped Carbon Framework Encapsulated with CoS_2_ Nanocatalysts Towards Safe Lithium‐Sulfur Batteries,” Composites Part B: Engineering 233 (2022): 109644, 10.1016/j.compositesb.2022.109644.

[212] Z. A. Ghazi , X. He , A. M. Khattak , et al., “MoS_2_ /Celgard Separator as Efficient Polysulfide Barrier for Long‐Life Lithium–Sulfur Batteries,” Advanced Materials 29 (2017): 1606817, 10.1002/adma.201606817. 28318064

[213] S. Yang , Z. Junfan , T. Tan , Y. Zhao , N. Liu , and H. Li , “A 3D MoS_2_/Graphene Microsphere Coated Separator for Excellent Performance Li‐S Batteries,” Materials 11 (2018): 2064, 10.3390/ma11102064. PMC6213909 30360425

[214] P. Han , S.‐H. Chung , and A. Manthiram , “Thin‐Layered Molybdenum Disulfide Nanoparticles as an Effective Polysulfide Mediator in Lithium–Sulfur Batteries,” ACS Applied Materials & Interfaces 10 (2018): 23122–23130, 10.1021/acsami.8b05397. 29932321

[215] M. Shi , Z. Liu , S. Zhang , et al., “A Mott–Schottky Heterogeneous Layer for Li–S Batteries: Enabling Both High Stability and Commercial‐Sulfur Utilization,” Advanced Energy Materials 12 (2022): 2103657, 10.1002/aenm.202103657.

[216] Q. Jiang , H. Xu , K. S. Hui , et al., “Inner‐Layer Indium Doping Achieved Highly Active and Stable Sulfur Vacancies in MoS_2_ for Superior Sulfur Redox Kinetics,” Advanced Materials 37 (2025): 2415986, 10.1002/adma.202415986. 40504702

[217] D. Lei , W. Shang , X. Zhang , et al., “Competing Reduction Induced Homogeneous Oxygen Doping to Unlock MoS_2_ Basal Planes for Faster Polysulfides Conversion,” Journal of Energy Chemistry 73 (2022): 26–34, 10.1016/j.jechem.2022.06.002.

[218] D. Lei , W. Shang , X. Zhang , et al., “Facile Synthesis of Heterostructured MoS_2_–MoO_3_ Nanosheets With Active Electrocatalytic Sites for High‐Performance Lithium–Sulfur Batteries,” ACS Nano 15 (2021): 20478–20488, 10.1021/acsnano.1c09007. 34860017

[219] Y. Li , T. Li , Y. Deng , et al., “Tuning the D‐Band Center of Bi_2_S_3_ ─MoS_2_ Heterostructure Towards Superior Lithium‐Sulfur Batteries,” Small 20 (2024): 2401921, 10.1002/smll.202401921. 38813749

[220] H. Li , D. Li , Y. Li , H. Liu , J. Li , and W. Han , “Coupling 5D Metallic and Conductive Network for Promoting Polysulfide Conversion,” Chemical Engineering Journal 502 (2024): 158205, 10.1016/j.cej.2024.158205.

[221] L. Pan , C. Zhang , H. Liu , et al., “MoS_2_@SiO_2_ Heterostructures Modified Separator Utilized for Efficient Adsorption and Rapid Conversion of Polysulfides,” Journal of Alloys and Compounds 993 (2024): 174546, 10.1016/j.jallcom.2024.174546.

[222] G. Sun , M. Jin , C. Y. Zhang , et al., “Design of an Ultra‐Highly Stable Lithium–Sulfur Battery by Regulating the Redox Activity of Electrocatalyst and the Growth of Lithium Dendrite Through Localized Electric Field,” ACS Nano 19 (2025): 871–884, 10.1021/acsnano.4c12217. 39705517

[223] S. Chen , G. Du , K. Hu , et al., “Machine Learning‐Guided Construction of MoS_2_/MoO_3_ Heterostructures on Hollow Carbon Shells for Polysulfide Mitigation in Lithium‐Sulfur Batteries,” Journal of Colloid and Interface Science 702 (2026): 138830, 10.1016/j.jcis.2025.138830. 40896987

[224] P. Han , S.‐H. Chung , and A. Manthiram , “Thin‐Layered Molybdenum Disulfide Nanoparticles as an Effective Polysulfide Mediator in Lithium–Sulfur Batteries,” ACS Applied Materials & Interfaces 10 (2018): 23122–23130, 10.1021/acsami. 29932321

[225] S. Bhoyate , B. Park , S. H. Oh , and W. Choi , “Defect Engineered MoWS Alloy Catalyst Boost the Polysulfide Conversion in Lithium–Sulfur Battery,” Journal of Power Sources 509 (2021): 230426, 10.1016/j.jpowsour.2021.230426.

[226] X. Yu , T. Liao , J. Tang , et al., “Edge Engineering in 2D Molybdenum Disulfide: Simultaneous Regulation of Lithium and Polysulfides for Stable Lithium–Sulfur Batteries,” Advanced Energy and Sustainability Research 2 (2021): 2100053, 10.1002/aesr.202100053.

[227] Y. Tang , Y. Huang , L. Luo , D. Fan , Y. Lu , and A. Manthiram , “Self‐Supported MoO_2_/MoS_2_ Nano‐Sheets Embedded in a Carbon Cloth as a Binder‐Free Substrate for High‐Energy Lithium–Sulfur Batteries,” Electrochimica Acta 367 (2021): 137482, 10.1016/j.electacta.

[228] N. Zheng , G. Jiang , X. Chen , J. Mao , N. Jiang , and Y. Li , “Battery Separators Functionalized with Edge‐Rich MoS_2_/C Hollow Microspheres for the Uniform Deposition of Li_2_S in High‐Performance Lithium–Sulfur Batteries,” Nano‐Micro Letters 11 (2019): 43, 10.1007/s40820-019-0275-z. PMC7770949 34138007

[229] M. Waqas , A. M. Soomro , S. Ali , et al., “Multifunctional Cathodic Interlayer With Polysulfide Immobilization Mechanism for High‐Performance Li‐S Batteries,” Energy Technology 8 (2020): 2000713, 10.1002/ente.202000713.

[230] M. Waqas , Y. Han , D. Chen , et al., “Molecular ‘Capturing’ and ‘Seizing’ MoS_2_/TiN Interlayers Suppress Polysulfide Shuttling and Self‐Discharge of Li–S Batteries,” Energy Storage Materials 27 (2020): 333–341, 10.1016/j.ensm.2020.02.019.

[231] J. Wu , N. You , X. Li , et al., “SiO_2_@MoS_2_ Core–Shell Nanocomposite Layers With High Lithium Ion Diffusion as a Triple Polysulfide Shield for High Performance Lithium–Sulfur Batteries,” Journal of Materials Chemistry A 7 (2019): 7644–7653, 10.1039/C9TA00982E.

[232] J. Wu , H. Zeng , X. Li , et al., “Dual‐Functional Interlayer Based on Radially Oriented Ultrathin MoS_2_ Nanosheets for High‐Performance Lithium–Sulfur Batteries,” ACS Applied Energy Materials 2 (2019): 1702–1711, 10.1021/acsaem.8b01805.

[233] J. Wu , X. Li , H. Zeng , et al., “Fast Electrochemical Kinetics and Strong Polysulfide Adsorption by a Highly Oriented MOS 2 Nanosheet@N‐Doped Carbon Interlayer for Lithium–Sulfur Batteries,” Journal of Materials Chemistry A 7 (2019): 7897–7906, 10.1039/C9TA00458K.

[234] X. Wang , N. Deng , J. Ju , et al., “Flower‐Like Heterostructured MoP–MoS2 Hierarchical Nanoreactor Enabling Effective Anchoring for LiPS and Enhanced Kinetics for High Performance Li–S Batteries,” Journal of Membrane Science 642 (2022): 120003, 10.1016/j.memsci.2021.120003.

[235] K. Xiang , X. Wen , J. Hu , S. Wang , and H. Chen , “Rational Fabrication of Nitrogen and Sulfur Codoped Carbon Nanotubes/MoS_2_ for High‐Performance Lithium–Sulfur Batteries,” Chemsuschem 12 (2019): 3602–3614, 10.1002/cssc.201901368. 31081248

[236] R. Wang , J. Li , Y. Zhang , et al., “Improved Li‐S Batteries Obtained by Using Multifunctional Separators Modified with Vapor Grown Carbon Fiber/MoS_2_ Composites,” Ceramics International 46 (2020): 21898–21905, 10.1016/j.ceramint.2020.04.284.

[237] X. Yu , G. Zhou , and Y. Cui , “Mitigation of Shuttle Effect in Li–S Battery Using a Self‐Assembled Ultrathin Molybdenum Disulfide Interlayer,” ACS Applied Materials & Interfaces 11 (2019): 3080–3086, 10.1021/acsami.8b19354. 30588794

[238] J. Zhang , J. Zhang , K. Liu , et al., “Abundant Defects‐Induced Interfaces Enabling Effective Anchoring for Polysulfides and Enhanced Kinetics in Lean Electrolyte Lithium–Sulfur Batteries,” ACS Applied Materials & Interfaces 11 (2019): 46767–46775, 10.1021/acsami.9b15638. 31763822

[239] Y. Yang , S. Wang , S. Lin , et al., “Rational Design of Hierarchical TiO_2_ /Epitaxially Aligned MoS_2_–Carbon Coupled Interface Nanosheets Core/Shell Architecture for Ultrastable Sodium‐Ion and Lithium–Sulfur Batteries,” Small Methods 2 (2018): 1800295, 10.1002/smtd.201800295.

[240] X. Gu , H. Kang , C. Shao , X. Ren , and X. Liu , “A Typha Angustifolia‐Like MoS_2_/Carbon Nanofiber Composite for High Performance Li‐S Batteries,” Frontiers in Chemistry 8 (2020): 149, 10.3389/fchem.2020.00149. PMC7063458 32195225

[241] T. Yang , B. Guo , W. Du , et al., “Design and Construction of Sodium Polysulfides Defense System for Room‐Temperature Na–S Battery,” Advanced Science 6 (2019): 1901557, 10.1002/advs.201901557. PMC6891912 31832316

[242] S. Yang , J. Zhang , T. Tan , Y. Zhao , N. Liu , and H. Li , “A 3D MoS_2_/Graphene Microsphere Coated Separator for Excellent Performance Li‐S Batteries,” Materials 11 (2018): 2064, 10.3390/ma11102064. PMC6213909 30360425

[243] S. Jiang , M. Chen , X. Wang , et al., “MoS_2_‐Coated N‐Doped Mesoporous Carbon Spherical Composite Cathode and CNT/Chitosan Modified Separator for Advanced Lithium Sulfur Batteries,” ACS Sustainable Chemistry & Engineering 6 (2018): 16828–16837, 10.1021/acssuschemeng.8b04157.

[244] M. Zhen , S.‐Q. Guo , and B. Shen , “Constructing Defect‐Rich MoS_2_ /N‐Doped Carbon Nanosheets for Catalytic Polysulfide Conversion in Lithium–Sulfur Batteries,” ACS Sustainable Chemistry & Engineering 8 (2020): 13318–13327, 10.1021/acssuschemeng.

[245] J. Wu , H. Zeng , X. Li , et al., “Ultralight Layer‐by‐Layer Self‐Assembled MoS_2_ ‐Polymer Modified Separator for Simultaneously Trapping Polysulfides and Suppressing Lithium Dendrites,” Advanced Energy Materials 8 (2018): 1802430, 10.1002/aenm.201802430.

[246] K. Gao , D. Xia , H. Ji , et al., “Linear‐PEI‐Derived Hierarchical Porous Carbon Nanonet Flakes Decorated With MoS_2_ as Efficient Polysulfides Stabilization Interlayers for Lithium–Sulfur Battery,” Energy & Fuels 35 (2021): 10303–10314, 10.1021/acs.energyfuels.1c01242.

[247] Y. Dong , P. Lu , H. Shi , et al., “2D hierarchical Yolk‐Shell Heterostructures as Advanced Host‐interlayer Integrated Electrode for Enhanced Li‐S Batteries,” Journal of Energy Chemistry 36 (2019): 64–73, 10.1016/j.jechem.2019.04.022.

[248] B. Zhao , Z. Cong , Z. Cheng , et al., “Facile Microwave‐Impulse Synthesis of Multifunctional rGO/MoS_2_/MoO_2_ Composites as a Permselective Separator‐Coating Layer for Li–S Batteries,” ACS Applied Energy Materials 4 (2021): 10252–10262, 10.1021/acsaem.1c02148.

[249] F. Li , G. Hu , S. Li , C. Hou , and J. Gao , “Dual‐Confined Sulfur Cathodes Based on SnO_2_‐Decorated MoS_2_ Microboxes for Long‐Life Lithium–Sulfur Batteries,” Electrochimica Acta 340 (2020): 135991, 10.1016/j.electacta.2020.135991.

[250] S. Wang , Y. Luo , Y. Fan , A. Ali , Z. Liu , and P. K. Shen , “Uniformly Distributed 1T/2H‐MoS_2_ Nanosheets Integrated by Melamine Foam‐Templated 3D Graphene Aerogels as Efficient Polysulfides Trappers and Catalysts in Lithium‐Sulfur Batteries,” Journal of Electroanalytical Chemistry 909 (2022): 116099, 10.1016/j.jelechem.2022.116099.

[251] J. Wang and J. Li , “C@MoS_2_ Modified Separator as Efficient Trapper and Catalysis for Promoting Polysulfide Conversion in Li‐S Battery,” Journal of Colloid and Interface Science 616 (2022): 298–303, 10.1016/j.jcis.2022.02.070. 35219195

[252] Q. Sheng , H. Liu , B. Jin , et al., “1T‐MoS_2_ Grown on Amorphous Carbon‐Coated Carbon Nanotubes for High‐Performance Lithium‐Sulfur Batteries,” Journal of Electroanalytical Chemistry 929 (2023): 117119, 10.1016/j.jelechem.2022.117119.

[253] Y. Gong , Y. Wang , Z. Fang , et al., “Constructing a Catalytic Reservoir Using Cobalt Nanoparticles‐MoS_2_@Nitrogen Doped Carbon Nanotubes on the Separator to Immobilize Polysulfides and Accelerate Their Conversion for Lithium‐Sulfur Batteries,” Chemical Engineering Journal 446 (2022): 136943, 10.1016/j.cej.2022.136943.

[254] Q. Li , H. Liu , B. Jin , et al., “Anchoring Polysulfides via a CoS_2_ /NC@1T MoS_2_ Modified Separator for High‐Performance Lithium–Sulfur Batteries,” Inorganic Chemistry Frontiers 10 (2023): 959–971, 10.1039/D2QI01884E.

[255] S. Wu , X. Nie , Z. Wang , Z. Yu , and F. Huang , “Magnetron Sputtering Engineering of Typha‐Like Carbon Nanofiber Interlayer Integrating Brush Filter and Chemical Adsorption for Li–S Batteries,” Carbon 201 (2023): 285–294, 10.1016/j.carbon.2022.09.020.

[256] J. Zhang , G. Xu , Q. Zhang , et al., “Mo‐O‐C Between MoS_2_ and Graphene Toward Accelerated Polysulfide Catalytic Conversion for Advanced Lithium‐Sulfur Batteries,” Advanced Science 9 (2022): 2201579, 10.1002/advs.202201579. PMC9353409 35666043

[257] Y. Jiang , P. Liang , M. Tang , et al., “A High‐Throughput Screening Permeability Separator With High Catalytic Conversion Kinetics for Li–S Batteries,” Journal of Materials Chemistry A 10 (2022): 22080–22092, 10.1039/d2ta04592c.

[258] D. Lei , W. Shang , X. Zhang , et al., “Competing Reduction Induced Homogeneous Oxygen Doping to Unlock MoS_2_ Basal Planes for Faster Polysulfides Conversion,” Journal of Energy Chemistry 75 (2022): 455–463, 10.1016/j.jechem.2022.06.002.

[259] F. Liu , N. Wang , C. Shi , et al., “Phosphorus Doping of 3D Structural MoS_2_ to Promote Catalytic Activity for Lithium‐Sulfur Batteries,” Chemical Engineering Journal 431 (2022): 133923, 10.1016/j.cej.2021.133923.

[260] P. Cheng , L. Shi , W. Li , et al., “Efficient Regulation of Polysulfides by MoS_2_/MoO_3_ Heterostructures for High‐Performance Li‐S Batteries,” Small 19 (2023): 2206083, 10.1002/smll.202206083. 36683234

[261] S. Deng , Y. Lv , Y. Zhao , et al., “Exquisitely Constructing Hierarchical Carbon Nanoarchitectures Decorated With Sulfides for High‐Performance Li–S Batteries,” Dalton Transactions (2024): 4753–4763, 10.1039/D4DT01234A. 38363131

[262] L. Pan , C. Zhang , H. Liu , et al., “MoS_2_@SiO_2_ Heterostructures Modified Separator Utilized for Efficient Adsorption and Rapid Conversion of Polysulfides,” Journal of Alloys and Compounds 1005 (2024): 174546, 10.1016/j.jallcom.2024.174546.

[263] J. Han , Z. Shao , Y. Jia , et al., “Hierarchical NiS_2_/1T‐MoS_2_ Nanocomposite for Stable Lithium Sulfur Batteries,” ACS Applied Nano Materials 7 (2024): 25637–25644, 10.1021/acsanm.4c04738.

[264] Y. Jiang , T. Shi , H. Wang , and A. Song , “rGO@MoS_2_ as the Host‐Separator Modifier With Efficient Polysulfides Trapping and Fast Li^+^ Diffusion for Lithium‐Sulfur Batteries,” Journal of Alloys and Compounds 1005 (2024): 175491, 10.1016/j.jallcom.2024.175491.

[265] J. Deng , G. Du , Y. Wang , et al., “Hollow ZnS‐SnS_2_@MoS_2_ Heterostructures for High‐efficiency Adsorption‐Storage‐Catalysis of Polysulfides in Lithium‐Sulfur Batteries,” Journal of Alloys and Compounds 1010 (2025): 177172, 10.1016/j.jallcom.2024.177172.

[266] L.‐J. He , J. Liu , T.‐T. Lv , A.‐C. Wei , and T.‐Q. Yuan , “1T‐Rich MoS2 Nanosheets Anchored on Conductive Porous Carbon as Effective Polysulfide Promoters for Lithium–Sulfur Batteries,” Journal of Colloid and Interface Science 671 (2024): 175–183, 10.1016/j.jcis.2024.05.150. 38797143

[267] X. Wang , G. Zhang , B. Wang , Y. Wu , and S. Guo , “Micro–Nanostructure Designed CoP@MoS_2_ Accelerating Polysulfide Conversion and Reaction Kinetics for Lithium–Sulfur Battery,” ACS Sustainable Chemistry & Engineering 12 (2024): 14018–14027, 10.1021/acssuschemeng.4c04968.

[268] C. Ding , Y. Ding , K. Wang , et al., “Regulating the Polysulfide Behavior by a Large‐Scale Two‐Dimensional Superlattice Interface in Li–S Chemistry,” ACS Nano 19 (2025): 26818–26830, 10.1021/acsnano.5c07372. 40664637

[269] S. Chen , G. Du , K. Hu , et al., “Machine Learning‐Guided Construction of MoS_2_/MoO_3_ Heterostructures on Hollow Carbon Shells for Polysulfide Mitigation in Lithium‐Sulfur Batteries,” Journal of Colloid and Interface Science 702 (2025): 138830, 10.1016/j.jcis.2025.138830. 40896987

[270] L. Yin , C. Zhang , W. Chen , et al., “Accelerating Polysulfide Conversion by 2D‐2D MoN/TiN Heterostructure Towards High‐Performance Lithium–Sulfur Battery,” Chemical Engineering Journal 512 (2025): 162147, 10.1016/j.cej.2025.162147.

[271] J. Deng , G. Du , Y. Wang , et al., “Hollow ZnS‐NC@MoS_2_ Heterostructure‐Reinforced Separator for Lithium‐Sulfur Batteries via Enhanced Adsorption‐Catalytic Synergistic Effect,” Journal of Alloys and Compounds 1036 (2025): 182143, 10.1016/j.jallcom.2025.182143.

[272] G. Liu , Q. Zeng , S. Tian , et al., “Boosting Polysulfide Redox Kinetics by Temperature‐Induced Metal‐Insulator Transition Effect of Tungsten‐Doped Vanadium Dioxide for High‐Temperature Lithium‐Sulfur Batteries,” Small 20 (2024): 2307040, 10.1002/smll.202307040. 37967337

[273] C. Yang , N. Gong , T. Chen , et al., “Enhanced Catalytic Conversion of Polysulfides Using High‐Percentage 1T‐Phase Metallic WS_2_ Nanosheets for Li–S Batteries,” Green Energy & Environment 7 (2022): 1340–1348, 10.1016/j.gee.2021.03.001.

[274] S. Ali , M. Waqas , X. Jing , et al., “Carbon–Tungsten Disulfide Composite Bilayer Separator for High‐Performance Lithium–Sulfur Batteries,” ACS Applied Materials & Interfaces 10 (2018): 39417–39421, 10.1021/acsami.8b12682. 30387589

[275] A. Paolella , D. Laul , V. Timoshevskii , et al., “The Role of Metal Disulfide Interlayer in Li–S Batteries,” Journal of Physical Chemistry C 122 (2018): 1014–1023, 10.1021/acs.jpcc.7b08719.

[276] J. Liu , K. Li , Q. Zhang , et al., “3D Tungsten Disulfide/Carbon Nanotube Networks as Separator Coatings and Cathode Additives for Stable and Fast Lithium–Sulfur Batteries,” ACS Applied Materials & Interfaces 13 (2021): 45547–45557, 10.1021/acsami.1c13193. 34528435

[277] G. Wang , Q. Jiao , Z. Zhang , et al., “Improved Electrochemical Behavior of Li–S Battery With Functional WS_2_@PB–PPy–Modified Separator,” Chemical Engineering Journal Advances 8 (2021): 100145, 10.1016/j.ceja.2021.100145.

[278] Q. Wang , A. Liu , S. Qiao , et al., “Mott‐Schottky MXene@WS_2_ Heterostructure: Structural and Thermodynamic Insights and Application in Ultra Stable Lithium−Sulfur Batteries,” Chemsuschem 16 (2023): 202300507, 10.1002/cssc.202300507. 37314096

[279] A. E. Shrshr , B. Miao , M. M. Elsenety , et al., “An Innovative One‐Step Hydrothermal Approach for Fabricating Ta_2_O_5_/WS_2_@rGO Heterostructure as a Coated Separator for Li–S Batteries,” Materials Today Chemistry 50 (2025): 103136, 10.1016/j.mtchem.2025.103136.

[280] J. Huang , D. Wang , Z. Cheng , et al., “Morphology and Electron Regulation of B‐WS_2_ @MXene Nanosheet Array Heterojunctions to Upgrade the Capacity and Longevity of Lithium–Sulfur Batteries,” Journal of Materials Chemistry A 13 (2025): 18880–18891, 10.1039/D5TA02390D.

[281] C. Yang , N. Gong , T. Chen , et al., “Enhanced Catalytic Conversion of Polysulfides Using High‐Percentage 1T‐Phase Metallic WS2 Nanosheets for Li–S Batteries,” Green Energy & Environment 6 (2022): 1340–1348, 10.1016/j.gee.2021.03.001.

[282] Q. Wang , A. Liu , S. Qiao , et al., “Mott‐Schottky MXene@WS_2_ Heterostructure: Structural and Thermodynamic Insights and Application in Ultra Stable Lithium−Sulfur Batteries,” Chemsuschem 19 (2023): 202300507, 10.1002/cssc.202300507. 37314096

[283] X. Xu , H. Xu , R. Wang , et al., “Enhanced Adsorption and Catalytic Conversion of Lithium Polysulfides Enabled by CoS_2_‐WS_2_/GNC Heterostructure‐Modified Separator,” Applied Surface Science 690 (2025): 164736, 10.1016/j.apsusc.2025.164736.

[284] Z. Chen , W. Lv , F. Kang , and J. Li , “Theoretical Investigation of the Electrochemical Performance of Transition Metal Nitrides for Lithium–Sulfur Batteries,” Journal of Physical Chemistry C 123 (2019): 25025–25030, 10.1021/acs.jpcc.9b04670.

[285] J. Zhu , J. Chen , Y. Luo , et al., “Lithiophilic Metallic Nitrides Modified Nickel Foam by Plasma for Stable Lithium Metal Anode,” Energy Storage Materials 23 (2019): 539–546, 10.1016/j.ensm.2019.04.005.

[286] S. Lu , L. Cai , J. Wang , et al., “2D Ultrathin Titanium Nitride Nanosheets as Separator Coatings for Li–S Batteries,” Small 20 (2024): 2307784, 10.1002/smll.202307784. 38279620

[287] F. Ma , Z. Chen , K. Srinivas , et al., “Vn Quantum Dots Anchored N‐Doped Carbon Nanosheets as Bifunctional Interlayer for High‐Performance Lithium‐Metal and Lithium‐Sulfur Batteries,” Chemical Engineering Journal 459 (2023): 141526, 10.1016/j.cej.2023.141526.

[288] W. Zhang , H. Li , R. Tao , et al., “In‐Situ Constructing Hetero‐Structured Mo_2_C‐Mo_2_N Embedded in Carbon Nanosheet as an Efficient Separator Modifier for High‐Performance Lithium‐Sulfur Batteries,” Chemical Engineering Journal 475 (2023): 146133, 10.1016/j.cej.2023.146133.

[289] Z.‐L. Xu , J.‐K. Kim , and K. Kang , “Carbon Nanomaterials for Advanced Lithium Sulfur Batteries,” Nano Today 19 (2018): 84–107, 10.1016/j.nantod.2018.02.006.

[290] C. He , Z. F. Qi , and W. X. Zhang , “Design of Transition Metal Carbonitrides (MCNS) as Promising Anchoring and High Catalytic Performance Materials for Lithium‐Sulfur Batteries,” Journal of Alloys and Compounds 934 (2023): 167786, 10.1016/j.jallcom.2022.167786.

[291] X. He , Y. Shuai , L. Na , et al., “High Performance Lithium‐Sulfur Batteries With Facile Titanium Nitride Particles Modified Separator,” Materials Letters 215 (2018): 91–94, 10.1016/j.matlet.2017.12.069.

[292] Z. Hao , L. Yuan , C. Chen , et al., “TiN as a Simple and Efficient Polysulfide Immobilizer for Lithium–Sulfur Batteries,” Journal of Materials Chemistry A 4 (2016): 17711–17717, 10.1039/C6TA07411A.

[293] B. Qi , X. Zhao , S. Wang , et al., “Mesoporous TiN Microspheres as an Efficient Polysulfide Barrier for Lithium–Sulfur Batteries,” Journal of Materials Chemistry A 6 (2018): 14359–14366, 10.1039/C8TA04920C.

[294] Q. Tang , H. Li , Y. Pan , et al., “TiN Synergetic With Micro‐/Mesoporous Carbon for Enhanced Performance Lithium–Sulfur Batteries,” Ionics 24 (2018): 2983–2993, 10.1007/s11581-018-2510-x.

[295] G. Xu , Q.‐B. Yan , P. Bai , H. Dou , P. Nie , and X. Zhang , “Nano‐Sized Titanium Nitride Functionalized Separator Improves Cycling Performance of Lithium Sulfur Batteries,” ChemistrySelect 4 (2019): 698–704, 10.1002/slct.201803285.

[296] Q. Shen , L. Huang , G. Chen , X. Zhang , and Y. Chen , “One‐Step Synthesis of Titanium Nitride/Nitrogen‐Doped Graphene Nanocomposite as Separator Modifying Material for Advanced Lithium‐Sulfur Batteries,” Journal of Alloys and Compounds 845 (2020): 155543, 10.1016/j.jallcom.2020.155543.

[297] J. Zhu , C. Jiao , T. Kang , L. Zhong , S. Zhao , and Y. Qiu , “A Sandwich‐Structured TiN/BN‐C Composite Interlayer With Enhanced Performance for Li S Batteries,” Journal of Electroanalytical Chemistry 862 (2020): 113963, 10.1016/j.jelechem.2020.113963.

[298] N. Li , L. Yu , and J. Xi , “Integrated Design of Interlayer/Current‐Collector: Heteronanowires Decorated Carbon Microtube Fabric for High‐Loading and Lean‐Electrolyte Lithium–Sulfur Batteries,” Small 17 (2021): 2103001, 10.1002/smll.202103001. 34331399

[299] J. Zhu , C. Ma , X. Jia , et al., “TiO_2_–TiN Decorated Carbon Nanoplates Derived from NH_2_‐MIL‐125 With Efficient Polysulfide Catalytic Conversion for Long‐Life Lithium–Sulfur Batteries,” Industrial & Engineering Chemistry Research 62 (2023): 14380–14390, 10.1021/acs.iecr.3c01206.

[300] Y. Kim , Y. Noh , J. Bae , H. Ahn , M. Kim , and W. B. Kim , “N‐Doped Carbon‐Embedded TiN Nanowires as a Multifunctional Separator for Li–S Batteries With Enhanced Rate Capability and Cycle Stability,” Journal of Energy Chemistry 57 (2021): 10–18, 10.1016/j.jechem.2020.08.050.

[301] Y. Fan , K. Liu , A. Ali , X. Chen , and P. K. Shen , “2D TiN@C Sheets Derived From MXene as Highly Efficient Polysulfides Traps and Catalysts for Lithium−Sulfur Batteries,” Electrochimica Acta 384 (2021): 138187, 10.1016/j.electacta.2021.138187.

[302] K. Liu , X. Zhang , F. Miao , et al., “In Situ Electrochemical Intercalation‐Induced Phase Transition to Enhance Catalytic Performance for Lithium–Sulfur Battery,” Small 17 (2021): 2100065, 10.1002/smll.202100065. 33792162

[303] G. Li , S. Zhang , S. Zhao , et al., “Design of Spherical TiN/Nb_4_N_5_ Heterostructure as Bifunctional Adsorption/Catalytic Material for Lithium Sulfur Batteries,” Materials Today Communications 33 (2022): 104403, 10.1016/j.mtcomm.2022.104403.

[304] Y. Zhu , X. Wu , M. Li , et al., “Synthesis of Titanium Molybdenum Nitride‐Decorated Electrospun Carbon Nanofiber Membranes as Interlayers to Suppress Polysulfide Shuttling in Lithium–Sulfur Batteries,” ACS Sustainable Chemistry & Engineering 10 (2022): 776–788, 10.1021/acssuschemeng.1c05959.

[305] G. H. Park , W.‐G. Lim , Y. H. Jeong , et al., “Activation of the Radical‐Mediated Pathway and Facilitation of the Li_2_S Conversion by N‐Doped Carbon‐Embedded Ti_1–x_Co_x_N Nanowires as a Multifunctional Separator with a High Donor‐Number Solvent Toward Advanced Lithium–Sulfur Batteries,” Small Structures 5 (2024): 2400293, 10.1002/sstr.202400293.

[306] Y. Xiang , L. Lu , F. Yan , et al., “Tuning the Crystallinity of Titanium Nitride on Copper‐Embedded Carbon Nanofiber Interlayers for Accelerated Electrochemical Kinetics in Lithium–Sulfur Batteries,” Carbon Energy 6 (2024): 450, 10.1002/cey2.450.

[307] K. Yang , X. Xu , C. Li , F. Zhao , J. Li , and Y. He , “Realizing Dual Regulation of Polysulfides and Lithium Ions by a Versatile Separator,” Science China Materials 67 (2024): 116–124, 10.1007/s40843-023-2695-5.

[308] P. Wan , X. Guo , S. Jing , et al., “Multicomponent Synergy and Simultaneous Improvement of Catalytic Conversion and Chemisorption of Polysulfides via CoFe/FeN_0.0324_/TiN Modified Li‐S Battery Separator,” Chemical Engineering Journal 504 (2025): 159014, 10.1016/j.cej.2024.159014.

[309] M. Shen , Y. Liu , X. Gao , et al., “Eutectoid Reaction Enabling In Situ Construction of Titanium Based Ternary Heterostructures for Boosted Lithium Polysulfide Conversion,” Small Methods 9 (2025): 2500157, 10.1002/smtd.202500157. 40302295

[310] X. Qian , F. Li , X. Yang , et al., “Decoration of Indium Tin Oxide Nanoparticles With Different Crystal Structures and Morphologies on Polypropylene Separator for Li‐S battery,” International Journal of Energy Research 45 (2021): 8992–9005, 10.1002/er.6432.

[311] Y. Kim , Y. Noh , J. Bae , H. Ahn , M. Kim , and W. B. Kim , “N‐Doped Carbon‐Embedded TiN Nanowires as a Multifunctional Separator for Li‐S Batteries With Enhanced Rate Capability and Cycle Stability,” Journal of Energy Chemistry 55 (2021): 1–9, 10.1016/j.jechem.2020.08.050.

[312] G. Li , S. Zhang , S. Zhao , X. Jiang , Y. Cui , and J. Lian , “Design of Spherical TiN/Nb_4_N_5_ Heterostructure as Bifunctional Adsorption/Catalytic Material for Lithium Sulfur Batteries,” Materials Today Communications 33 (2022): 104403, 10.1016/j.mtcomm.2022.104403.

[313] H. Chen , Y. Wei , X. Cao , et al., “Boosting Polysulfide Capture and Redox Conversion by Functional Separator Combined With Porous Hosts for Advanced Li‐S Batteries,” Journal of Electroanalytical Chemistry 918 (2022): 116483, 10.1016/j.jelechem.2022.116483.

[314] Y. Xiang , L. Lu , F. Yan , et al., “Tuning the Crystallinity of Titanium Nitride on Copper‐Embedded Carbon Nanofiber Interlayers for Accelerated Electrochemical Kinetics in Lithium–Sulfur Batteries,” Carbon Energy 6 (2024): 450, 10.1002/cey2.450.

[315] F. Sultanov , N. Zhumasheva , A. Dangaliyeva , et al., “Graphene‐Like Porous Carbon‐titanium Nitride Composite as an Efficient Separator Modifier for Lithium‐Sulfur Batteries,” Nano Energy 145 (2025): 111420, 10.1016/j.nanoen.2025.111420.

[316] L. Yin , C. Zhang , W. Chen , et al., “Accelerating Polysulfide Conversion by 2D‐2D MoN/TiN Heterostructure Towards High‐Performance Lithium–Sulfur Battery,” Chemical Engineering Journal 512 (2025): 162147, 10.1016/j.cej.2025.159014.

[317] M. Wu , Z. Xing , R. Zhu , et al., “2D Nano‐Channeled Molybdenum Compounds for Accelerating Interfacial Polysulfides Catalysis in Li–S Battery,” Small 20 (2024): 2306991, 10.1002/smll.202306991. 37939298

[318] D. Tian , X. Song , M. Wang , et al., “MoN Supported on Graphene as a Bifunctional Interlayer for Advanced Li‐S Batteries,” Advanced Energy Materials 9 (2019): 1901940, 10.1002/aenm.201901940.

[319] X. Zhou , J.‐L. Yang , N. Li , J. Yang , and J. Xi , “Highly Catalytic Porous Mon Nanosheets Anchored Carbon Microtubes Interlayer for Lithium‐Sulfur Batteries,” Materials Today Energy 24 (2022): 100941, 10.1016/j.mtener.2021.100941.

[320] T. Tan , N. Chen , Z. Wang , et al., “Thorn‐Like Carbon Nanofibers Combined with Molybdenum Nitride Nanosheets as a Modified Separator Coating: An Efficient Chemical Anchor and Catalyst for Li–S Batteries,” ACS Applied Energy Materials 5 (2022): 6654–6662, 10.1021/acsaem.2c00080.

[321] X.‐Y. Yue , J. Zhang , J. Bao , et al., “Sputtered MoN Nanolayer as a Multifunctional Polysulfide Catalyst for High‐Performance Lithium‐Sulfur Batteries,” 2 (2022): 329–338, 10.1016/j.esci.2022.03.003.

[322] Y. Kong , L. Wang , M. Mamoor , et al., “Co/MoN Invigorated Bilateral Kinetics Modulation for Advanced Lithium–Sulfur Batteries,” Advanced Materials 36 (2024): 2310143, 10.1002/adma.202310143. 38134811

[323] L. Ban , “Novel Functional Separator With MoO_2_ Nanosheets for Advanced Electrochemical Performance in Li–S Battery,” Ionics 28 (2022): 2769–2773, 10.1007/s11581-022-04537-z.

[324] X. Zhang , J. Zhao , Y. Zhao , Z. Bakenov , and Y. Zhang , “Engineering Zwitterionic Barrier by Squaraine‐Based Porous Organic Framework Fiber for Superior Lithium‐Sulfur Batteries,” Electrochimica Acta 397 (2021): 139276, 10.1016/j.electacta.2021.139276.

[325] D. Wang , K. Ma , J. Hao , et al., “Engineering Single‐Atom Catalysts as Multifunctional Polysulfide and Lithium Regulators toward Kinetically Accelerated and Durable Lithium‐Sulfur Batteries,” Chemical Engineering Journal 466 (2023): 143182, 10.1016/j.cej.2023.143182.

[326] Z. Sun , J. Zhang , L. Yin , et al., “Conductive Porous Vanadium Nitride/Graphene Composite as Chemical Anchor of Polysulfides for Lithium‐Sulfur Batteries,” Nature Communications 8 (2017): 14627, 10.1038/ncomms14627. PMC5337987 28256504

[327] J. Hou , M. Yang , X. Guo , et al., “Chemisorption‐Catalysis Synergy Driven VN‐V_2_O_3_ Anchored on Reduced Graphene Oxide Heterostructure for Stable Lithium‐Sulfur Battery Separators,” Journal of Power Sources 645 (2025): 237197, 10.1016/j.jpowsour.2025.237197.

[328] L. Ma , Y. Wang , Z. Wang , et al., “Wide‐Temperature Operation of Lithium–Sulfur Batteries Enabled by Multi‐Branched Vanadium Nitride Electrocatalyst,” ACS Nano 17 (2023): 11527–11536, 10.1021/acsnano.3c01469. 37288710

[329] X. Yu , F. Cheng , and K. Xie , “Porous Single‐Crystalline Vanadium Nitride Octahedra with a Unique Electrocatalytic Performance,” New Journal of Chemistry 46 (2022): 1392–1398, 10.1039/D1NJ05504F.

[330] E. Jing , L. Chen , S. Xu , et al., “Dual Redox Catalysis of Vn/Nitrogen‐Doped Graphene Nanocomposites for High‐Performance Lithium‐Sulfur Batteries,” Journal of Energy Chemistry 64 (2022): 574–582, 10.1016/j.jechem.2021.05.015.

[331] J. Shi , Y. Yao , L. Xue , et al., “Application of Magnetron Sputtering to Deposit a Multicomponent Separator with Polysulfide Chemisorption and Electrode Stabilization for High‐Performance Lithium‑Sulfur Batteries,” Surface and Coatings Technology 405 (2021): 126580, 10.1016/j.surfcoat.2020.126580.

[332] Q. Sun , Q. Shu , C. Ma , et al., “A Bifunctional N‐Doped Carbon Nanosheet Embedded with VN/Mo_2_C Heterointerfaces Electrocatalyst for Expediting Sulfur Redox and Lithium Regulation Toward Advanced Li‐S Battery,” Chemical Engineering Journal 489 (2024): 151115, 10.1016/j.cej.2024.151115.

[333] G. Ruan , Y. Shen , J. Yao , et al., “Vanadium Nitride Nanocrystals Decorated Ultrathin, N‐Rich and Hierarchically Porous Carbon Nanosheets as Superior Polysulfides Mediator for Stable Lithium‐Sulfur Batteries,” Journal of Power Sources 566 (2023): 232922, 10.1016/j.jpowsour.2023.232922.

[334] T. Guo , M. Liu , W. Zhang , et al., “Tuning the D‐Band Center Upshift of Molybdenum via Constructing Well‐Matched VN‐MoC Heterostructures Enables Optimal Adsorption and Efficient Conversion for Lithium Polysulfides,” Chemical Engineering Journal 522 (2025): 167316, 10.1016/j.cej.2025.167316.

[335] Q. Chen , S. Chen , L. Zhao , J. Ma , H. Wang , and J. Zhang , “Interface Coating of Iron Nitride on Carbon Cloth for Reversible Lithium Redox in Rechargeable Battery,” Chemical Engineering Journal 431 (2022): 133961, 10.1016/j.cej.2021.133961.

[336] S. Liu , Y. Liu , X. Zhang , et al., “Multifunctional Vanadium Nitride‐Modified Separator for High‐Performance Lithium–Sulfur Batteries,” Nanomaterials 14 (2024): 656, 10.3390/nano14080656. PMC11053798 38668150

[337] X. Zhang , X. Bai , C. Wei , et al., “Triggering the Electronic Microenvironment of Extraordinary Nitrogen‐Bridged Atomic Iron Coordinated With In‐Plane Nitrogen by Manipulating Phase‐Reconfigured 2D Vanadium Nitride MXenes Toward Invigorated Lithium–Sulfur Batteries,” Energy & Environmental Science 17 (2024): 7403–7415, 10.1039/d4ee02979h.

[338] Y. Song , S. Zhao , Y. Chen , et al., “Enhanced Sulfur Redox and Polysulfide Regulation via Porous VN‐Modified Separator for Li–S Batteries,” ACS Applied Materials & Interfaces 11 (2019): 5687–5694, 10.1021/acsami.8b22014. 30714710

[339] Y.‐Y. Park , S.‐H. Moon , D.‐H. Park , et al., “Vanadium Nitride/Reduced Graphene Oxide Composite Interlayer With Dual Lithium‐Polysulfide Adsorption Effect for Lithium‐Sulfur Batteries,” Journal of Alloys and Compounds 960 (2023): 170812, 10.1016/j.jallcom.2023.170812.

[340] Y. Shi , G. Xu , G. Liang , et al., “PEG‐VN Modified PP Separator for High‐Stability and High‐Efficiency Lithium‐Sulfur Batteries,” Acta Physico‐Chimica Sinica 41 (2025): 100082, 10.1016/j.actphy.2025.100082.

[341] J. Pu , J. Hu , Z. Shen , Z. Liang , and Z. Wang , “Ultrathin Nanosheets of Iron Nitride–Iron Oxide Heterostructures as Amphiphilic Separator Modification Layer for Lithium–Sulfur Batteries,” ACS Applied Energy Materials 8 (2025): 17520–17529, 10.1021/acsaem.5c03077.

[342] A. Saleem , M. K. Majeed , R. Iqbal , et al., “Nitrogenized 2D Covalent Organic Framework Decorated Ni‐Rich Single Crystal Cathode to Ameliorate the Electrochemical Performance of Lithium Batteries,” Advanced Materials Interfaces 9 (2022): 2200800, 10.1002/admi.202200800.

[343] T. Wang , Q. Dong , F. Wang , et al., “Functionless Cobalt Toward Functional Cobalt Nitride: Catalytic Sulfur Carrier for Lithium‐Sulfur Pouch Batteries,” Matter 7 (2024): 1035–1053, 10.1016/j.matt.2023.12.018.

[344] A. Joshi , S. Mohapatra , A. Gupta , and B. Nandan , “Review of Metal Nitrides for Lithium–Sulfur Batteries: Design, Mechanisms, and Prospects,” Chemistry – A European Journal 31 (2025): 202500971, 10.1002/chem.202500971. 40405642

[345] Z. Li , Y. Luo , L. Zhang , J. Li , and W. Cai , “Recent Development of Transition Metal Nitride Composites as Cathode Carrier for Lithium Sulfur Battery,” Journal of Power Sources 651 (2025): 237553, 10.1016/j.jpowsour.2025.237553.

[346] J. Pu , J. Wu , Y. Tan , and S. Yu , “Cobalt Nitride Nanocrystals Coated Separator as Multifunctional Interlayer for Improving Polysulfides Regulation,” Journal of Alloys and Compounds 920 (2022): 165964, 10.1016/j.jallcom.2022.165964.

[347] M. Luo , Y. Bai , R. Sun , et al., “Enhancing Anchoring and Catalytic Conversion of Polysulfides by Nitrogen Deficient Cobalt Nitride for Advanced Lithium‐Sulfur Batteries,” Journal of Energy Chemistry 73 (2022): 407–415, 10.1016/j.jechem.2022.05.029.

[348] M. Zhao , H.‐J. Peng , Z.‐W. Zhang , et al., “Activating Inert Metallic Compounds for High‐Rate Lithium–Sulfur Batteries Through In Situ Etching of Extrinsic Metal,” Angewandte Chemie International Edition 58 (2019): 3779–3783, 10.1002/anie.201812062. 30548388

[349] X. Li , W. Shang , S. Zhang , C. Xu , J. Lian , and G. Li , “The Design of the Ni(3)N/Nb(4)N(5) Heterostructure as Bifunctional Adsorption/Electrocatalytic Materials for Lithium‐Sulfur Batteries,” Nanomaterials 15 (2025): 1015, 10.3390/nano15131015. PMC12251257 40648722

[350] F. Yi , H. Wang , J. Wang , et al., “Bifunctional Mott‐Schottky‐Type Co‐Fe_3_N Electrocatalyst as Sulfur Host and Separator Modifier to Enhance the Transformation Kinetics of Li_2_S,” Journal of Power Sources 624 (2024): 235562, 10.1016/j.jpowsour.2024.235562.

[351] J. Li , X. Zhang , Z. Zhang , Y. Li , and L. Zhu , “Preparation of Ldhs‐Derived Co‐Ni_3_N Heterostructure Modified Porous Carbon Nanofibers as Advanced Interlayers for Lithium‐Sulfur Batteries,” Journal of Alloys and Compounds 971 (2024): 172731, 10.1016/j.jallcom.2023.172731.

[352] H. Jia , J. Fan , P. Su , T. Guo , and M.‐C. Liu , “Cobalt Nitride Nanoparticles Encapsulated in N‐Doped Carbon Nanotubes Modified Separator of Li–S Battery Achieving the Synergistic Effect of Restriction‐Adsorption‐Catalysis of Polysulfides,” Small 20 (2024): 2311343, 10.1002/smll.202311343. 38236167

[353] Y. Tang , Y. Yuan , Y. Mo , et al., “CoN//Co_9_S_8_ Co‐Embedded in Reduced Graphene Oxide as Catalyst for Catalytic Conversion of Polysulfides,” Materials Today Physics 46 (2024): 101480, 10.1016/j.mtphys.2024.101480.

[354] C. Ma , Y. Zhang , Y. Feng , et al., “Engineering Fe–N Coordination Structures for Fast Redox Conversion in Lithium–Sulfur Batteries,” Advanced Materials 33 (2021): 2100171, 10.1002/adma.202100171. 34145629

[355] X. Wang , D. Wang , C. Ma , et al., “Conductive Fe_2_N/N‐RGO Composite Boosts Electrochemical Redox Reactions in Wide Temperature Accommodating Lithium‐Sulfur Batteries,” Chemical Engineering Journal 427 (2022): 131622, 10.1016/j.cej.2021.131622.

[356] Y. Cao , C. Liu , M. Wang , et al., “Lithiation of Covalent Organic Framework Nanosheets Facilitating Lithium‐Ion Transport in Lithium‐Sulfur Batteries,” Energy Storage Materials 29 (2020): 207–215, 10.1016/j.ensm.2020.04.029.

[357] L. Liu , F. Yang , L. Ge , X. Wang , L. Cui , and H. Yang , “Facile and Low‐Cost Preparation of Co and N Co‐Doped Hierarchical Porous Carbon as a Functional Separator for Li‐S Batteries,” Electrochimica Acta 401 (2022): 139380, 10.1016/j.electacta.2021.139380.

[358] N. Wu , J. Wang , C. Liao , et al., “A Novel Fire Safety Separator Decorated by Co_2_P/Co/C Multifunctional Layer for Li‐S Batteries,” Journal of Alloys and Compounds 910 (2022): 164919, 10.1016/j.jallcom.2022.164919.

[359] Y. Lin , S. He , Z. Ouyang , et al., “Synergistic Engineering of Cobalt Selenide and Biomass‐Derived S, N, P Co‐Doped Hierarchical Porous Carbon for Modulation of Stable Li‐S Batteries,” Journal of Materials Science & Technology 135 (2023): 170–181, 10.1016/j.jmst.2022.06.024.

[360] L. Jin , K. Zhang , J. Chen , et al., “ZIF 67@Ce‐MOF Derived Co‐N‐C@CeO_2_‐C for Separator Modification of Lithium Sulfur Batteries,” Materials Research Bulletin 174 (2024): 112785, 10.1016/j.materresbull.2024.112785.

[361] C. Xu , X. Jiang , M. Huang , et al., “A Multi‐Functional CoN‐Mo_2_N Heterostructure Nanorods for High Performance Lithium‐Sulfur Batteries,” Chemical Engineering Journal 488 (2024): 151132, 10.1016/j.cej.2024.151132.

[362] X. Li , W. Shang , S. Zhang , C. Xu , J. Lian , and G. Li , “The Design of the Ni_3_N/Nb_4_N_5_ Heterostructure as Bifunctional Adsorption/Electrocatalytic Materials for Lithium–Sulfur Batteries,” Nanomaterials 15 (2025): 1015, 10.3390/nano15131015. PMC12251257 40648722

[363] M. Zhao , H.‐J. Peng , Z.‐W. Zhang , et al., “Activating Inert Metallic Compounds for High‐Rate Lithium–Sulfur Batteries Through In Situ Etching of Extrinsic Metal,” Angewandte Chemie International Edition 58 (2019): 3779–3783, 10.1002/anie.201813067. 30548388

[364] T. Zhu , Y. Sha , H. Zhang , et al., “Embedding Fe_3_C and Fe_3_N on a Nitrogen‐Doped Carbon Nanotube as a Catalytic and Anchoring Center for a High‐Areal‐Capacity Li–S Battery,” ACS Applied Materials & Interfaces 13 (2021): 20153–20161, 10.1021/acsami.1c03358. 33877793

[365] Y. Zhang , C. Ma , W. He , et al., “Mxene and Mxene‐Based Materials for Lithium‐Sulfur Batteries,” Progress in Natural Science: Materials International 31 (2021): 501–513, 10.1016/j.pnsc.2021.07.003.

[366] X. Geng , C. Liu , Y. Sun , et al., “A Ti_3_C_2_T_x_ Mxene ‐ Carbon Nanocage‐Sulfur Cathode With High Conductivity for Improving the Performance of Li‐S Batteries,” Journal of Alloys and Compounds 895 (2022): 162586, 10.1016/j.jallcom.2021.162586.

[367] T. Yuan , X. Chen , Y. Zhao , et al., “Dual Regulation of Surface Termination and Interlayer Spacing of Mxene for High‐Performance Vanadium Redox Flow Batteries,” Chemical Engineering Journal 533 (2026): 174389, 10.1016/j.cej.2026.174389.

[368] N. Fu , Z. Liu , B. Shen , et al., “Carbon and MXene Dual Confinement and Dense Structural Engineering Toward Construct High Performance Micron‐SiO_x_ Anode for Li‐Ion Batteries,” Advanced Functional Materials 34 (2024): 2410839, 10.1002/adfm.202410839.

[369] J. Zhang , Y. Yao , X. Song , K. Shi , and Y. Song , “MXene Nanosheets Modified With Different Intercalators as the Separator for Lithium–Sulfur Batteries,” Journal of Materials Chemistry C 13 (2025): 10103–10110, 10.1039/D4TC05447D.

[370] L. Liao , F. Wang , J. He , L. Ma , Q. Liu , and Y. Deng , “Ultrathin TA‐MXene Modification Layer With Sub‐Nanometer Interlayer Channels Suppresses Polysulfide Shuttling and Accelerates Li^+^ Desolvation,” Advanced Functional Materials 10 (2026): 12612, 10.1002/adfm.202512612.

[371] Z. H. Luo , M. Zheng , M. X. Zhou , et al., “2D Nanochannel Interlayer Realizing High‐Performance Lithium–Sulfur Batteries,” Advanced Materials 37 (2025): 2417321, 10.1002/adma.202417321. 39846826

[372] B. Zhao , L. Song , Z. Zou , et al., “Electrospun Ti_3_C_2_ MXene Fiber‐Decorated Interlayer for Synchronous Sulfur Activation and Lithium Stabilization,” Advanced Functional Materials 35 (2025): 2500079, 10.1002/adfm.202500079.

[373] B. Cetiner , A. A. Hamedani , B. Iskandarani , et al., “High‐Performance Lithium–Sulfur Batteries With MXene‐Transition Metal Oxide Decorated Electrospun Interlayers for Optimized Polysulfide Conversion,” International Journal of Energy Research 17 (2025): 8862016, 10.1155/er/8862016.

[374] J. Zhou , Z. Pei , Z. Sui , et al., “Hierarchical Porous and Three‐Dimensional MXene/SiO_2_ Hybrid Aerogel Through a Sol‐Gel Approach for Lithium–Sulfur Batteries,” Molecules 27 (2022): 7073, 10.3390/molecules27207073. PMC9610511 36296667

[375] A. Bhattacharyya , S. Saha , S. Jena , et al., “Epoxy‐Based Vitrimeric Semi‐Interpenetrating Network/MXene Nanocomposites for Hydrogen Gas Barrier Applications,” Nanoscale 17 (2025): 5755–5769, 10.1039/D4NR04702H. 39906025

[376] C. Zhang , J. Feng , X. Guo , et al., “Blocking Polysulfide by Physical Confinement and Catalytic Conversion of SiO_2_@MXene for Li–S Battery,” Applied Physics Letters 122 (2023): 193901, 10.1063/5.0147522.

[377] Y. Cui , X. Zhou , X. Huang , L. Xu , and S. Tang , “Binary Transition‐Metal Sulfides/MXene Synergistically Promote Polysulfide Adsorption and Conversion in Lithium–Sulfur Batteries,” ACS Applied Materials & Interfaces 15 (2023): 49223–49232, 10.1021/acsami.3c11170. 37838949

[378] Y. Li , Z. Ni , Z. Wang , and J. Feng , “In‐Situ Construction of Ag‐Doped ZnSe Nanodots Anchored on MXene at Room Temperature for Boosted Polysulfide Conversion Kinetics,” Chemical Engineering Journal 527 (2026): 171824, 10.1016/j.cej.2025.171824.

[379] M. Zhen , X. Meng , X. Wang , et al., “Layered Double Hydroxides‐MXene Heterointerfaces With Abundant Anion Vacancies Expediting Sulfur Redox Kinetics for High‑Performance Lithium–Sulfur Batteries,” Chemical Engineering Journal 489 (2024): 151285, 10.1016/j.cej.2024.151285.

[380] J. Feng , W. Liu , C. Shi , et al., “Enabling Fast Diffusion/Conversion Kinetics by Thiourea‐Induced Wrinkled N, S Co‐Doped Functional MXene for Lithium‐Sulfur Battery,” Energy Storage Materials 67 (2024): 103328, 10.1016/j.ensm.2024.103328.

[381] W. Yu , S. Ma , M. He , et al., “Immobilization and Kinetic Acceleration of Lithium Polysulfides by Iodine‐Doped MXene Nanosheets in Lithium–Sulfur Batteries,” The Journal of Physical Chemistry C 126 (2022): 10986–10994, 10.1021/acs.jpcc.2c02689.

[382] Q. Wang , B. Deng , X. Zhang , et al., “Partially Oxidized Ti_3_–Nb C_2_T MXene Nanosheets as Efficient Sulfur Hosts and Separator Modification for Li S Batteries,” Journal of Materiomics 11 (2025): 100920, 10.1016/j.jmat.2024.07.005.

[383] Y. Zhao , Y. Zhang , Z. He , et al., “Building Flexibly Porous Conductive Skeleton Inlaid with Surface Oxygen‐Dominated MXene as an Amphiphilic Nanoreactor for Stable Li‐S Pouch Batteries,” Energy Storage Materials 47 (2022): 434–444, 10.1016/j.ensm.2022.02.006.

[384] X. Zhang , Z. Ni , X. Bai , et al., “Hierarchical Porous N‐doped Carbon Encapsulated Fluorine‐free MXene with Tunable Coordination Chemistry by One‐pot Etching Strategy for Lithium–Sulfur Batteries,” Advanced Energy Materials 13 (2023): 2301349, 10.1002/aenm.202301349.

[385] C. Wei , T. Fang , X. Tang , P. Wang , and X. Liu , “Non‐Negligible Role of Multifunctional MXene Hosts for Li–S Batteries: Anchoring and Electrocatalysis,” The Journal of Physical Chemistry C 126 (2022): 17066–17075, 10.1021/acs.jpcc.2c05214.

[386] Q. Gu , Y. Cao , J. Chen , et al., “Fluorine‐Modulated MXene‐Derived Catalysts for Multiphase Sulfur Conversion in Lithium–Sulfur Battery,” Nano‐Micro Letters 16 (2024): 266, 10.1007/s40820-024-01482-6. PMC11319705 39133318

[387] M. Du , P. Geng , J. Shi , H. Xu , W. Feng , and H. Pang , “Triple Effect of “Conductivity‐Adsorption‐Catalysis” Enables MXene@FeCoNiP to be Sulfur Hosts for Lithium–Sulfur Batteries,” Inorganic Chemistry 63 (2024): 10823–10831, 10.1021/acs.inorgchem.4c01553. 38803192

[388] H. Gu , W. Yue , J. Hu , et al., “Asymmetrically Coordinated Cu–N_1_C_2_ Single‐Atom Catalyst Immobilized on Ti_3_C_2_T_x_ MXene as Separator Coating for Lithium–Sulfur Batteries,” Advanced Energy Materials 13 (2023): 2204014, 10.1002/aenm.202204014.

[389] M. Zhen , Z. Zhang , X. Du , et al., “Atomic Environment Engineering of Cobalt Single Atom‐Nanocluster Synergistic Sites on Nitrogen‐Doped Mxene for Bidirectional Sulfur Electrocatalysis and Uniform Lithium Deposition,” Advanced Functional Materials 169 (2025): 1, 10.1002/adfm.202529884.

[390] S. L. Jing , X. Peng , L. Yuan , S. Lu , Y. Zhang , and H. Fan , “Highly Stable and Active Cerium Single‐Atom Immobilized Ti_3_C_2_T_X_ MXene for High‐Stability Lithium‑Sulfur Batteries,” Chemical Engineering Journal 521 (2025): 166774, 10.1016/j.cej.2025.166774.

[391] J. Yu , Y. Jia , Q. Jin , L. Bai , H. Gao , and J. Yao , “Bimetallic Sulfide Based on Mxene as a Multifunctional Separator for Lithium Sulfur Batteries,” Journal of Alloys and Compounds 1039 (2025): 183265, 10.1016/j.jallcom.2025.183265.

[392] A. Mirtaleb , M. Scekic , and R. Wang , “Synergistic SnSe_2_@Ti_3_C_2_T_x_MX ene Heterostructured Separator for Highly Efficient Polysulfide Adsorption, Catalytic Redox Acceleration, and Enhanced Electrochemical Stability in Advanced Lithium–Sulfur Batteries,” Energy & Environmental Materials 9 (2026): 70173, 10.1002/eem2.70173.

[393] W. S. Deng , J. Tang , M. Jafarpour , F. Nüesch , J. Heier , and C. Zhang , “Multifunctional SnO_2_ QDs/MXene Heterostructures as Laminar Interlayers for Improved Polysulfide Conversion and Lithium Plating Behavior,” Nano‐Micro Letters 16 (2024): 229, 10.1007/s40820-024-01446-w. PMC11213846 38940902

[394] J. Xu , C. Ma , J. Wang , W. Qiao , and L. Ling , “1T‐VSe_2_@Ti_3_C_2_ Heterostructure With a Built‐In Electric Field and Li_x_VSe_2_ Intercalation–Conversion for High‐Performance Li–S Chemistry: Accelerating Sulfur Conversion and Lithium‐Ion Transport,” ACS Applied Energy Materials 8 (2025): 7247–7256, 10.1021/acsaem.5c00633.

[395] X. Gao , S. Yu , X. Zhao , et al., “Enabling Efficient Polysulfide Conversion by Self‐Assembled Co‐CoP@MXene for Li‐S Batteries,” Advanced Sustainable Systems 9 (2025): 01196, 10.1002/adsu.202501196.

[396] K. Yang , Y. Liu , F. Zhao , et al., “Multifunction of MXene in Lithium–Sulfur Batteries: A Review,” Energy & Fuels 38 (2024): 13837–13857, 10.1021/acs.energyfuels.4c02385.

[397] Z. Liu , M. Chen , D. Zhou , and Z. Xiao , “Scavenging of “Dead Sulfur” and “Dead Lithium” Revealed by Integrated–Heterogeneous Catalysis for Advanced Lithium–Sulfur Batteries,” Advanced Functional Materials 33 (2023): 2306321, 10.1002/adfm.202306321.

[398] C. Fu , D. He , X. Liu , et al., “MXene/ZIF‐67‐Based Janus Separator for High‐Performance Lithium‐Sulphur Batteries,” Journal of Alloys and Compounds 1037 (2025): 182305, 10.1016/j.jallcom.2025.182305.

[399] Y. Ren , L. Hu , S. Chang , et al., “MXene‐Bimetallic Hybrids via Mixed Molten Salts Etching for Kinetics‐Enhanced and Dendrite‐Free Lithium–Sulfur Batteries,” Small 20 (2024): 2400068, 10.1002/smll.202400068. 38593293

[400] Á. Bonilla , A. Gentile , C. Ferrara , et al., “Dual Role of Ti_3_C_2_T_x_ MXene in Li‐S Batteries: Cathode Additive and Separator Modifier for Enhanced Performance,” Advanced Functional Materials 36 (2025): 19742, 10.1002/adfm.202519742.

[401] K. Yang , F. Zhao , J. Li , H. Yang , Y. Wang , and Y. He , “Designing Organic–Inorganic Hybrid Materials to Construct a Complementary Interface With Versatility for Li–S Batteries,” Advanced Functional Materials 34 (2024): 2410236, 10.1002/adfm.202410236.

[402] X. Li , Q. Guan , Z. Zhuang , et al., “Ordered Mesoporous Carbon Grafted Mxene Catalytic Heterostructure as Li‐Ion Kinetic Pump Toward High‐Efficient Sulfur/Sulfide Conversions for Li‐S Battery,” ACS Nano 17 (2023): 1653–1662, 10.1021/acsnano.2c11663. 36607402

[403] K. Xu , J. Shi , Y. Qin , D. He , C. Wang , and T. Wang , “Electronic‐State Polarization Engineering‐Regulated Fluorinated Covalent Organic Framework Nanocables for Fast Lithium‐Ion Storage,” Advanced Science 13 (2026): 75084, 10.1002/advs.75084. PMC13285149 41904940

[404] D. He , W. Gu , X. Liu , et al., “Biomimetic Mineralization Guided Protein‐Based Multifunctional Bio‐Separators Realizing Dendrites Free and High‐Performance Lithium‑Sulfur Batteries,” Journal of Colloid and Interface Science 700, no. Part 2 (2025): 138498, 10.1016/j.jcis.2025.138498. 40729870

[405] D. He , T. Wang , J. Lu , et al., “Protein‐Guided Biomimetic Calcification Constructing 3D Nitrogen‐Rich Core‐Shell Structures Realizing High‐Performance Lithium‐Sulfur Batteries,” Advanced Materials 37 (2025): 2416268, 10.1002/adma.202416268. 39797477

[406] W. Gu , D. He , Y. Qin , et al., “From Efficiency to Sustainability: Organic Additives for Interfacial Regulation in Lithium Metal Batteries,” Chemical Science 16 (2025): 18050–18091, 10.1039/D5SC03975D. PMC12466591 41019644

[407] A. Bonilla , A. Gentile , C. Ferrara , et al., “Dual Role of Ti_3_C_2_Tx MXene in Li‐S Batteries: Cathode Additive and Separator Modifier for Enhanced Performance,” Advanced Functional Materials 36 (2025): 19742, 10.1002/adfm.202519742.

[408] M. Zhen , Z. Zhang , X. Du , et al., “Atomic Environment Engineering of Cobalt Single Atom‐Nanocluster Synergistic Sites on Nitrogen‐Doped MXene for Bidirectional Sulfur Electrocatalysis and Uniform Lithium Deposition,” Advanced Functional Materials 36 (2025): 29884, 10.1002/adfm.202417.

[409] S. Jing , X. Peng , S. Li , et al., “Synergistic Realization of Fast Polysulfide Redox Kinetics and Stable Lithium Anode in Li‐S Battery From CoNi‐MOF/MXene Derived CoNi@TiO_2_/C Heterostructure,” Chinese Chemical Letters 36 (2025): 110732, 10.1016/j.cclet.2024.110732.

[410] X. Gao , S. Yu , X. Zhao , et al., “Hollow‐Structured Co‐CoP Intercalated MXene for High‐Performance Lithium‐Sulfur Batteries,” Advanced Functional Materials 35 (2025): 2410047, 10.1002/adfm.202410047.

[411] J. Zhang , L. Zhu , H. Ren , Y. Song , and K. Shi , “Trimetallic Synergy‐Enhanced Multidimensional Composite Separator for High‐Rate Lithium‐Sulfur Batteries,” Journal of Colloid and Interface Science 702 (2026): 138844, 10.1016/j.jcis.2025.138844. 40902483

[412] Y. Wang , R. Niu , L. Chen , Y. Yang , H. Yu , and X. Qiu , “Fabrication of MXene/MOF Composite Separators for High Performance Lithium‐Sulfur Batteries,” Chemical Engineering Journal 512 (2025): 162305, 10.1016/j.cej.2025.162305.

[413] H. Wang , Q. Shu , Z. Zhou , et al., “Engineering NiCo Single Atom Alloy on MXene as Cascade Catalyst for High‐Performance Li–S Batteries,” Nano Research 18 (2025): 94907557, 10.26599/NR.2025.94907557.

[414] B. Zhang , J. You , P. Liu , Y. Li , and W. Wang , “A Bifunctional NiS_2_@MXene (Ti_3_C_2_T_x_) Composite Separator for High‐Performance Lithium–Sulfur Batteries With Enhanced Polysulfide Inhibition and Uniform Lithium‐Ion Transport,” Journal of Materials Chemistry A 13 (2025): 17884–17897, 10.1039/d5ta01365h.

[415] Y. Song , Y. Sun , L. Chen , et al., “Seeding Co Atoms on Size Effect‐Enabled V_2_C MXene for Kinetically Boosted Lithium–Sulfur Batteries,” Advanced Functional Materials 34 (2024): 2409748, 10.1002/adfm.202409748.

[416] Z. Wang , H. Jiang , C. Wei , et al., “Ultrasmall CoFe Bimetallic Alloy Anchored on Fluoride‐Free MXene by One‐Pot Etching Strategy for the Barrier‐Adsorption‐Catalyst Functions of Polysulfides in Lithium‐Sulfur Batteries,” Advanced Functional Materials 34 (2024): 2315178, 10.1002/adfm.202315178.

[417] Z. Huang , Y. Liang , Z. Wu , et al., “Multifunctional Ultrathin Ti_3_C_2_T_x_ MXene@CuCo_2_O_4_/PE Separator for Ultra‐High‐Energy‐Density and Large‐Capacity Lithium‐Sulfur Pouch Cells,” Advanced Materials 37 (2025): 2410318, 10.1002/adma.202410318. 39529518

[418] J. Feng , C. Zhang , W. Liu , et al., “Enabling Efficient Anchoring‐Conversion Interface by Fabricating Double‐Layer Functionalized Separator for Suppressing Shuttle Effect,” Angewandte Chemie International Edition 63 (2024): 202407042, 10.1002/anie.202407042. 39004938

[419] T. Li , W. Liu , Y. Liu , et al., “Covalent Organic Frameworks Integrated MXene as Selective “Ion‐Sieving” Heterostructure Catalyst for Kinetics‐Reinforced Li–S Batteries,” Chemical Engineering Journal 498 (2024): 155817, 10.1016/j.cej.2024.155817.

[420] S. Ge , Q. Zhao , Y. Liu , et al., “Ultrathin 2D–2D MXene‐LDH Interlayer With High Polysulfide Adsorption Ability for Advanced Li–S Batteries,” ACS Applied Materials & Interfaces 16 (2024): 50650–50658, 10.1021/acsami.4c08834. 39259716

[421] Q. Wang , S. Qiao , C. Huang , et al., “Multi‐Heterostructured MXene/NiS_2_/Co_3_S_4_ With S‐Vacancies to Promote Polysulfide Conversion in Lithium–Sulfur Batteries,” ACS Applied Materials & Interfaces 16 (2024): 24502–24513, 10.1021/acsami.4c00660. 38706136

[422] T. Li , Y. Liu , J. Wang , H. Hao , Z. Yu , and H. Liu , “Constructing Metal Telluride‐Grafted MXene as Electron “Donor–Acceptor” Heterostructure for Accelerating redox Kinetics of High‐Efficiency Li–S Batteries,” Journal of Materials Chemistry A 12 (2024): 12691–12701, 10.1039/d4ta01278.

[423] T. Zhang , Z. Xu , H. Chen , et al., “Mxene Quantum Dots Decorated Reduced Graphene Oxide Networks as Multifunctional Electrocatalyst for Advanced Lithium‐sulfur Batteries,” Chemical Engineering Journal 488 (2024): 150886, 10.1016/j.cej.2024.150886.

[424] H. Gu , W. Yue , J. Hu , et al., “Asymmetrically Coordinated Cu–N_1_C_2_ Single‐Atom Catalyst Immobilized on Ti_3_C_2_T_x_ MXene as Separator Coating for Lithium–Sulfur Batteries,” Advanced Energy Materials 13, no. 20 (2023): 2204014, 10.1002/aenm.202204014.

[425] Y. Song , P. Tang , Y. Wang , et al., “Insight Into Accelerating Polysulfides Redox Kinetics by BN@MXene Heterostructure for Li–S Batteries,” Small 19, no. 38 (2023): 2302386, 10.1002/smll.202302386. 37196415

[426] H. Y. Cai , P. Huang , Z. Zhang , H. Tan , Q. Han , and W.‐Q. Han , “In‐Situ Grown Ti_3_C_2_T @CoSe_2_ Heterostructure as Trapping‐Electrocatalyst for Accelerating Polysulfides Conversion in Lithium‐Sulfur Battery,” Chemical Engineering Journal 474 (2023): 145862, 10.1016/j.cej.2023.145862.

[427] M. Zheng , Z. Luo , Y. Song , et al., “Carbon‐Coated Nitrogen, Vanadium Co‐Doped MXene Interlayer for Enhanced Polysulfide Shuttling Inhibition in Lithium‐Sulfur Batteries,” Journal of Power Sources 580 (2023): 233445, 10.1016/j.jpowsour.2023.233445.

[428] X. Wang , B. Zhu , D. Xu , et al., “Synergistic Effects of Co_3_Se_4_ and Ti_2_C_3_T_x_ for Performance Enhancement on Lithium–Sulfur Batteries,” ACS Applied Materials & Interfaces 22 (2023): 26882–26892, 10.1021/acsami.3c04767. 37246565

[429] X. Zhou , Y. Cui , X. Huang , Q. Zhang , B. Wang , and S. Tang , “Interface Engineering of Fe_3_Se_4_/FeSe Heterostructures Encapsulated in MXene for Boosting LiPS Conversion and Inhibiting Shuttle Effect,” Chemical Engineering Journal 457 (2023): 141139, 10.1016/j.cej.2022.141139.

[430] X. Wang , J. Guo , K. Xu , et al., “In Situ Self‐Assembled NiS_2_ Nanoparticles on MXene Nanosheets as Multifunctional Separators: Regulating Shuttling Effect and Boosting Redox Reaction Kinetics of Lithium Polysulfides,” Applied Surface Science 645 (2024): 158859, 10.1016/j.apsusc.2023.158859.

[431] G. Zhang , C. Zhou , J. Long , et al., “Bottom‐Up Synthesis of 2D Heterostructures Enables Effective Polysulfides Inhibition and Conversion,” Nano Research 16, no. 6 (2023): 8488–8496, 10.1007/s12274-023-5535-z.

[432] T. Zhu , D. Chen , Y. Mao , et al., “Hollow Structure Co_1‐x_S/3D‐Ti_3_C_2_Tx MXene Composite for Separator Modification of Lithium‐Sulfur Batteries,” ACS Applied Materials & Interfaces 15, no. 49 (2023): 57088–57098, 10.1021/acsami.3c13234. 38041635

[433] C. Zhou , M. Li , N. Hu , et al., “Single‐Atom‐Regulated Heterostructure of Binary Nanosheets to Enable Dendrite‐Free and Kinetics‐Enhanced Li–S Batteries,” Advanced Functional Materials 32, no. 33 (2022): 2204635, 10.1002/adfm.202204635.

[434] Y. Ren , Q. Zhai , B. Wang , et al., “Synergistic Adsorption‐Electrocatalysis of 2D/2D Heterostructure Toward High Performance Li‐S Batteries,” Chemical Engineering Journal 439 (2022): 135535, 10.1016/j.cej.2022.135535.

[435] Y. Ren , B. Wang , H. Liu , et al., “CoP Nanocages Intercalated MXene Nanosheets as a Bifunctional Mediator for Suppressing Polysulfide Shuttling and Dendritic Growth in Lithium‐Sulfur Batteries,” Chemical Engineering Journal 450 (2022): 138046, 10.1016/j.cej.2022.138046.

[436] X. Yu , Y. Yang , L. Si , J. Cai , X. Lu , and Z. Sun , “V_4_C_3_T_x_ MXene: First‐principles Computational and Separator Modification Study on Immobilization and Catalytic Conversion of Polysulfide in Li‐S Batteries,” Journal of Colloid and Interface Science 627 (2022): 992–1002, 10.1016/j.jcis.2022.07.082. 35905585

[437] T. Wang , Y. Liu , X. Zhang , et al., “Interspersing Partially Oxidized V_2_C Nanosheets and Carbon Nanotubes Toward Multifunctional Polysulfide Barriers for High‐Performance Lithium‐Sulfur Batteries,” ACS Applied Materials & Interfaces 13 (2021): 56085–56094, 10.1021/acsami.1c16191. 34783521

[438] N. Li , Y. Xie , S. Peng , X. Xiong , and K. Han , “Ultra‐Lightweight Ti_3_C_2_T MXene Modified Separator for Li–S Batteries: Thickness Regulation Enabled Polysulfide Inhibition and Lithium Ion Transportation,” Journal of Energy Chemistry 42 (2020): 116–125, 10.1016/j.jechem.2019.06.007.

[439] J. Wang , T. Zhao , Z. Yang , et al., “MXene‐Based Co, N‐Codoped Porous Carbon Nanosheets Regulating Polysulfides for High‐Performance Lithium–Sulfur Batteries,” ACS Applied Materials & Interfaces 42 (2019): 38654–38662, 10.1021/acsami.9b14574. 31566358

