# Supplementary material for: Review of Inorganic Separator Engineering for Next‐Generation Lithium–Sulfur Batteries: Compromise or Cornerstone?
Source: Adv Mater. 2026 Aug 20;38(59):e74605. doi: 10.1002/adma.74605 (PMC13689895; doi:10.1002/adma.74605)
Supplement: Supplementary file 1 [file ADMA-38-e74605-s001.docx]

**Review of Inorganic Separator Engineering for Next Generation Lithium–Sulfur Batteries: Compromise or Cornerstone?**

*Yuting Qin ^1^, Tianyi Wang ^1, *^, Xingyu Wang ^1^, Kaifu Xu ^1^, Yuzhou Wang ^3^, Chengyin Wang ^1^, Bing Sun ^2, *^, Guoxiu Wang ^2, *^*

^1^ School of Chemistry & Materials, Yangzhou University, 180 Si-Wang-Ting Road, Yangzhou, Jiangsu 225002, China.

^2^ Centre for Clean Energy Technology, School of Mathematical and Physical Science, Faculty of Science, University of Technology Sydney, Sydney, NSW 2007, Australia.

^3^ Department of Mechanical Engineering, National University of Singapore, 9 Engineering Drive 1, 117576, Singapore.

E-mail: [Wangty@yzu.edu.cn](mailto:Wangty@yzu.edu.cn) (T.Y. Wang); [Bing.sun@uts.edu.au](mailto:Bing.sun@uts.edu.au) (B. Sun); [Guoxiu.wang@uts.edu.au](mailto:Guoxiu.wang@uts.edu.au) (G.X. Wang);

**S1. Bibliometric data collection and analysis**

The bibliometric analysis presented in Figures 1 and 3 was conducted to provide a trend-level overview of research development in lithium–sulfur battery separator engineering. Literature data were retrieved from the Web of Science Core Collection using a Topic (TS) search strategy. The search was performed using the core terms “lithium-sulfur battery” and “separator” in combination with representative transition-metal compound-related keywords covering major material families (including metal oxides, sulfides, nitrides, carbides, and related inorganic systems). This strategy was designed to capture the overall evolution of separator research rather than to perform an exhaustive enumeration of all possible chemical systems. Only journal articles and review papers were included to ensure consistency of scientific content. Duplicate records were removed using the built-in Web of Science deduplication function prior to data export. For temporal trend analysis (Figure 1), publications were categorized according to year of publication to illustrate general research growth patterns. For keyword co-occurrence analysis (Figure 3), the cleaned dataset was imported into VOSviewer. A thesaurus file was applied to unify keyword variations, including synonyms and formatting inconsistencies, to improve visualization clarity. This step was used solely for standardization and does not alter the underlying co-occurrence relationships. All analyses are intended as trend-level bibliometric representations rather than fully exhaustive quantitative database mining results.

**S2. The formula of the FoM framework**

To enable a quantitative and unbiased comparison of separator engineering strategies in Li-S batteries, a data-driven evaluation framework, termed *FoM_L-S_*, is established. This framework is motivated by the intrinsic inconsistency across literature reports, where key electrochemical metrics-including initial capacity, cycle number, and decay rate-are often measured under vastly different sulfur loadings, rendering direct comparison unreliable.

By integrating essential electrochemical descriptors into unified mathematical formulations, this framework effectively decouples the long-standing trade-off between sulfur loading and cycling stability, thereby enabling a holistic assessment of practical performance. The areal energy factor (*E_areal_*) is defined as:

$$E_{areal}=\frac{L_{s}\times C_{initial}\times V_{avg}\times{CE}_{avg}}{1000} (1)$$

Here, *L_s_* represents the sulfur loading, *C_initia_* is the initial specific capacity, *V_avg_* denotes the average discharge voltage, and *CE_avg_* reflects the average Coulombic efficiency. The normalization factor (1000) ensures unit consistency in mWh cm^-2^. Unlike conventional gravimetric metrics, *E_areal_* inherently penalizes artificially inflated capacities obtained at ultralow sulfur loadings, thus providing a more realistic representation of energy density. To capture long-term durability, a cycle stability factor (*S_cycle_*) is introduced:

$$S_{cycle}=\frac{1}{{\Delta R}_{decay}}\times\ln\left( N \right)\times\left( 1-\frac{{\Delta R}_{decay}\times N}{100} \right) (2)$$

Here, *ΔR_decay_* denotes the capacity decay rate per cycle, and *N* is the cycle number. The inverse dependence on *ΔR_decay_* amplifies the contribution of highly stable systems, while the logarithmic term *ln(N)* mitigates the dominance of excessively long cycling data. The retention constraint further excludes systems suffering from severe degradation, effectively condensing multidimensional cycling behavior into a quantitative metric of reversibility. To unify energy output and durability, a normalized figure of merit is defined:

$${FoM}_{Li-S}\text{=}\text{ω}\text{1}\left( \frac{E_{areal}}{E_{ref}} \right)\text{+}\text{ω}\text{2}\left( \frac{S_{cycle}}{S_{ref}} \right) (3)$$

where *E_ref_* and *S_ref_* are normalization constants, and *ω₁* and *ω_2_* are weighting factors that allow flexible emphasis depending on application scenarios. This formulation enables direct ranking of materials on a common scale, overcoming inconsistencies arising from disparate testing conditions. Based on this framework, the collected literature data are first projected into the (*E_areal_*, *S_cycle_*) space, where distinct material-dependent clustering behavior is observed. Metal oxides are predominantly confined to the low-*E_areal_* and low-*S_cycle_* region, while carbides exhibit moderate improvement. In contrast, sulfides and nitrides extend toward higher areal capacities and improved cycling stability.

**Figure S1.** A network visualization graph of high-frequency keywords “separator”, “metal compounds”, and “lithium-sulfur batteries” during the recent 5 years (2022-April/2026) (data collected from Web of Science and analyzed by VOS-Viewer). Material-specific keyword networks for a) metal oxides, b) metal sulfides, c) metal nitrides and d) metal carbides, respectively, demonstrating their distinct functional emphases. Oxides are mainly associated with strong chemical adsorption, sulfides with catalytic conversion and reaction kinetics, and nitrides and carbides with enhanced conductivity and interfacial regulation. The comparison highlights the functional differentiation and synergistic evolution of inorganic material systems.

**Figure S2.** Distribution of Li-S separator systems in the *E_areal_- S_cycle_* performance space. a) Overall distribution of literature-reported separator systems, where each data point represents an individual study and is color-coded by material category (oxides, carbides, sulfides, and nitrides). The bubble size reflects the magnitude of the integrated performance indicator. b-e) Material-specific distributions for b) metal oxides, c) metal nitrides, d) metal sulfides, and e) metal carbides. Distinct clustering behaviors are observed among different material families, indicating their intrinsic differences in balancing areal energy output and cycling stability. Oxides are mainly confined to low- *E_areal_* and low-*S_cycle_* regions, while sulfides and nitrides extend toward higher performance domains. This scatter-based representation highlights the inherent trade-off between sulfur loading and cycling durability but lacks a unified metric for direct comparison.

**Table S1.** Representative metal oxide-based separator coating materials reported for Li-S batteries, summarizing their structural characteristics, separator modification strategies, and corresponding electrochemical performances.

| Material | Structural characteristics | Coating method | Cycling performance  (mAh g^-1^) | Electrolyte/sulfur ratio  (μL mg^-1^) | Ref. |
| --- | --- | --- | --- | --- | --- |
| ZnO/CNT/RGO | ZnO nanoparticles anchored in a CNT/RGO network | Vacuum filtration coating | 768  (150th, 0.2 C) | 20 | [1] |
| Li_4_Ti_5_O_12_ (LTO)/GO@PP | 0D/2D structure | Vacuum filtration | 697  (500th, 1 C) | / | [2] |
| TiO_2_/GO | 3D net structure | Doctor blade coating | 843.4  (100th, 0.2 C) | / | [3] |
| TiO_2_/KB | 1D/0D structure | Vacuum filtration | 754  (200th, 1 C) | / | [4] |
| RGO/TiO_2_（B） | 0D/2D structure | Vacuum filtration | 454  (100th, 0.2 C) | 30 | [5] |
| TiO_2_-TiN/GO/PP | 0D/2D structure | Vacuum filtration | 927  (300th,0.3 C) | 10.29-36.85 | [6] |
| Bi_2_Te_3_ /TiO_2_ | 0D/2D structure | Doctor blade coating | 77.9%  (1000th, 1 C) | 3.8 | [7] |
| TiO_2_-TiN | 0D/2D structure | Doctor blade coating | 517  (500th, 0.5 C) | / | [8] |
| OVs-TiO_2_ | Ultra-thin nanosheets | Doctor blade coating | 631  (500th, 2 C) | / | [9] |
| A/R-TiO_2_ | Homogeneous junction structure | Doctor blade coating | 63%  (300th, 0.5 C) | 4.0 | [10] |
| TCNFs | Hollow fiber structure | Doctor blade coating | 549  (1000th, 0.5 A g^-1^) | / | [11] |
| TiH_2_@TiO_2_ | Core-shell structure | Doctor blade coating | / | / | [12] |
| OVs-TiO_2_ | Flower-shaped microspheres | Doctor blade coating | / | 25 | [13] |
| TNTs/CNTs | Network structure | Doctor blade coating | 557  (200th, 1 C) | / | [14] |
| PAN-TiO | Nanofiber structure | Electrospinning | 695  (100th, 0.5 C) | 20 | [15] |
| MWCNTs/LLTO | Dense coating structure | Doctor blade coating | 828  (200th, 0.5 C) | 12/6 | [16] |
| HSCo/TiO_2-x_ | Porous nanosheet structure | Doctor blade coating | 1028.87  (100th, 0.2 C) | / | [17] |
| TiP_2_O_7_/CNTs | 3D net structure | Doctor blade coating | 835  (300th, 4 C) | 8 | [18] |
| TiO_2_ (P90) | Dense coating structure | Dip-coating | / | / | [19] |
| MnO_2_@PE | Self-assembled dense-packed MnO_2_ nanocrystal layers | Self-assembly | 603  (500th, 0.5 C) | 7.0-8.8 | [20] |
| SMO separator | Self-assembled dense-packed MnO_2_ nanocrystal layers | Self-assembly | 494  (500th, 0.5 C) | 20 | [21] |
| S-PTFE/α-MnO_2_ | Nanowire composite gas film | Spray-coating | 1189  (500th, 0.2 C) | 5 | [22] |
| Treated MnO_2_ nanosheets | Nanosheet structure | Doctor blade coating | 698  (500th, 1 C) | 6.46 | [23] |
| α-MnO_2_/RuO_2_ | Nanofiber | Doctor blade coating | / | 8 | [24] |
| MnO_x_-NiO_x_/C | Dense coating structure | Doctor blade coating | 510  (700th, 2 C) | 23/11 | [25] |
| MnFe_2_O_4_/AB | Dense coating structure | Doctor blade coating | 625  (500th, 1 C) | 25 | [26] |
| Mn-Fe LDH/CNT | MnOx layered double hydroxide | Doctor blade coating | 1138  (200th, 0.2 C) | 20 | [27] |
| MWCNTs/MnO_2_@PP@Li | Integrated monolithic structure | Blade coating and Vacuum evaporation | 560  (300th, 1 C) | 30 | [28] |
| Ni-MnO@CNTs | 0D/1D structure | Vacuum filtration | 979  (500th, 1 C) | 10 | [29] |
| S-PTFE/MnO_2_ | Nanowire gas film | Drop-coating | 1189  (500th, 0.2 C) | 5-15 | [30] |
| MS-HEO | 0D irregular-shaped high-entropy oxide (HEO) nanoparticles. | Doctor blade coating | 762  (450th, 1 C) | 15 | [31] |
| CoNiCuMnMo-HEA@rGO | 0D/2D structure | Doctor blade coating | 570  (1100th, 3 C) | / | [32] |
| MnSA@HNC | 3D hollow structure | Doctor blade coating | 479  (1400th, 1 C) | 15 | [33] |
| Co |  |  |  |  |  |
| Co_9_S_8_/CoO-G | 0D/3D structure | Spray drying and blade coating | 925  (1000th, 1 C) | / | [34] |
| Spent LiCoO_2_ | Micron-sized LiCoO_2_ particles with a surface-derived spinel phase (Co_3_O_4_) coating. | Doctor blade coating | 685.7  (200th, 1600 mA g^-1^) | / | [35] |
| Co_3_V_2_O_8_ (CVO) | Flake-like structure | Doctor blade coating | 1014  (3000th, 0.1 C) | 15 | [36] |
| Co@CoO@N-C/rGO | Core-shell structure | Doctor blade coating | 903  (100th, 0.2 C) | 20 | [37] |
| CoO/NiO@C-NC | Hollow nanocages | Filtration coating | / | / | [38] |
| CoO-Co_3_O_4_ HNTs | Hollow nanotubes | Doctor blade coating | 242  (1000th, 5 C) | 8-10.17 | [39] |
| CoSe_2_/CoO-CNTs | Mott-Schottky heterostructure rods blended with CNTs | Vacuum filtration coating | / | 20 | [40] |
| CoO-Co_3_O_4_ | Hollow Nanotubes | Doctor blade coating | 631.5  (1000th, 5 C) | 8.0 | [41] |
| V-Co_3_O_4_ NS | Hexagonal nanosheets | Doctor blade coating | 642.7  (1900th, 3 C) | 6.0-8.0 | [42] |
| CeO_2_-CoO/rGO | 2D rGO-supported CeO2-CoO nanoparticles enriched with oxygen vacancies | Doctor blade coating | 620  (1000th, 1 C) | 4.7 | [43] |
| ZnO/NGO (NDG) | ZnO nanoparticles anchored on N-doped graphene | Doctor blade coating | 848  (200th, 0.1 C) | / | [44] |
| Co_3_O_4_/ZnO | Hollow dodecahedral heterojunction | Doctor blade coating | 500.9  (400th, 0.5 C) | / | [45] |
| ZnO@N-CNTs | ZnO nanoparticles anchored on N-doped CNTs | Doctor blade coating | 722  (150th, 0.2 C) | 13 | [46] |
| ZnO/N/KB | ZnO/N-doped carbon composited with Ketjen Black (KB) | Doctor blade coating | 495.9  (400th, 0.5 C) | / | [47] |
| Y_2_O_3_/ZnO@C | Y_2_O_3_/ZnO heterojunction@C derived from Y-MOF/Zn-MOF | Doctor blade coating | 518.9  (400th, 0.5 C) | 25 | [48] |
| ZnO-ZnS/rGO | ZnO-ZnS heterostructure anchored on rGO | Doctor blade coating | 910  (100th, 0.5 C) | 12 | [49] |
| DCNT@SNT-ZnO | Triple-layer structure: Defluorinated CNT@SiO_2_@ZnO | Doctor blade coating | 1032  (200th, 0.2 C) | 10 | [50] |
| CoNi@ZnV_2_O_4_/ZnO-N, C | ZnV_2_O_4_/ZnO heterojunction and CoNi nanoalloys as well as N-doped carbon layer | Doctor blade coating | 533  (900th, 1 C) | / | [51] |

The PRI values of titanium oxide-, manganese oxide-, cobalt oxide-, and zinc oxide-based separators are systematically summarized in **Tables S2-S5**, where four key descriptors, including energy contribution score (ECS), lean-electrolyte compatibility score (LECS), structural penalty score (SPS), and device validation score (DVS), are integrated to establish a comprehensive evaluation framework. The comparative analysis reveals the intrinsic trade-offs among different inorganic separator architectures, balancing electrochemical enhancement with practical application limitations. High PRI values originate from the synergistic contribution of energy enhancement, electrolyte efficiency, structural optimization, and device-level validation rather than catalytic activity alone.

**Table S2. Component analysis of the Practical Relevance Index (PRI) for representative** Titanium Oxide-based **based separator systems.**

| System | ECS | LECS | SPS | DVS | PRI |
| --- | --- | --- | --- | --- | --- |
| TiO_2_-TiN/GO/PP | 1.533 | 0.486 | 1.428 | 0.2 | 0.791 |
| Bi_2_Te_3_/TiO_2_ | 3.863 | 1.316 | 0 | 1 | 6.179 |
| Anatase/rutile TiO_2_ heterojunction | 1.160 | / | 0.837 | 1 | 1.323 |
| OVs-TiO_2_ | 1.427 | 0.200 | 2.721 | 0.2 | -0.894 |
| Co/TiO_2-x_ | 2.668 | / | 1.737 | 1 | 1.931 |
| TiP_2_O_7_/CNTs | 1.600 | 0.625 | 1.775 | 0.2 | 0.650 |
| All PRI values are derived from the unified evaluation framework proposed in this work, rather than directly reported in original literature. | | | | | |

**Table S3. Component analysis of the Practical Relevance Index (PRI) for representative** Manganese Oxide-based **based separator systems.**

| System | ECS | LECS | SPS | DVS | PRI |
| --- | --- | --- | --- | --- | --- |
| MnO_2_@PE | 1.007 | 0.714 | 0.313 | 0 | 1.608 |
| MnO_2_@PP  (SMO) | 0.608 | 0.250 | 0.438 | 0 | 0.620 |
| Treated MnO_2_ | 2.277 | 0.774 | / | 0.2 | 3.551 |
| α-MnO_2_/RuO_2_  (MRO-8) | 2.140 | 0.625 | / | 2.765 | 3.318 |
| MnOx-NiOx/C@PP | 0.642 | 0.455 | 1.727 | 0 | -0.431 |
| MnFe_2_O_4_/AB@PP (MFO-5%) | 0.573 | 0.200 | 1.963 | 0 | -0.990 |
| MFLC (Mn-Fe LDH/CNT) | 0.661 | 0.250 | 1.656 | 0 | -0.545 |
| MWCNTs/S-MWCNTs/MnO_2_@PP@Li | 0.912 | 0.167 | 3.811 | 0 | -2.532 |
| Ni-MnO@CNTs/PP | 1.523 | 0.500 | 1.595 | 0 | 0.627 |
| MS-HEO | 2.249 | 0.333 | 1.187 | 0 | 1.896 |
| MnSA@HNC | 1.777 | 0.333 | 1.896 | 1 | 1.214 |
| All PRI values are derived from the unified evaluation framework proposed in this work, rather than directly reported in original literature. | | | | | |

**Table S4. Component analysis of the Practical Relevance Index (PRI) for representative** Cobalt Oxide-based **based separator systems.**

| System | ECS | LECS | SPS | DVS | PRI |
| --- | --- | --- | --- | --- | --- |
| CoO/NiO@C-NC | 1.884 | / | 1.378 | 0.5 | 1.006 |
| Co_9_S_8_/CoO-G | 1.169 | 0.333 | 1.974 | 0.2 | -0.272 |
| Co_3_V_2_O_8_ (CVO) | 0.700 | 0.333 | 1.323 | 1.0 | 0.710 |
| Co@CoO@N-C/rGO | 0.818 | 0.250 | / | 0.2 | 0.636 |
| CoO-Co_3_O_4_  (HNTs) | 1.632 | 0.492 | / | 0.5 | 2.624 |
| LaCoO_3_ (LCO-VLa) | 1.749 | / | 1.374 | 0.2 | 0.575 |
| Co_3_O_4_ (V-Co_3_O_4_ NS) | 4.485 | 0.625 | / | 0.5 | 5.610 |
| CeO_2_-CoO/rGO | 1.960 | 1.064 | / | 1.0 | 4.024 |
| TiN/CoO@N-CNT (TCCNT) | 0.593 | 0.500 | / | 0.2 | 1.293 |
| All PRI values are derived from the unified evaluation framework proposed in this work, rather than directly reported in original literature. | | | | | |

**Table S5. Component analysis of the Practical Relevance Index (PRI) for representative** Zinc Oxide-based **based separator systems.**

| System | ECS | LECS | SPS | DVS | PRI |
| --- | --- | --- | --- | --- | --- |
| ZnO/CNT/RGO | 1.545 | 0.500 | 0.922 | 0.2 | 1.323 |
| Y_2_O_3_/ZnO@C | 1.866 | 0.200 | 1.316 | 0.5 | 1.250 |
| ZnO@N-CNTs | 1.394 | 0.385 | 1.304 | 0.2 | 0.675 |
| ZnO/RGO | 1.425 | 0.200 | 3.301 | 0.2 | -1.476 |
| ZnO/N/KB | 1.073 | / | 1.000 | 0.2 | 0.273 |
| ZnO-ZnS/rGO | 2.087 | 0.833 | 1.241 | 0.5 | 2.180 |
| DCNT@SNT-ZnO | 1.734 | 0.500 | 2.311 | 0.2 | 0.123 |
| CoNi@ZnV_2_O_4_/ZnO-N, C | 1.671 | 0.417 | / | 0.2 | 2.287 |
| All PRI values are derived from the unified evaluation framework proposed in this work, rather than directly reported in original literature. | | | | | |

**Table S6.** Representative metal sulfide-based separator coating materials for Li-S batteries, highlighting their structural design, polysulfide regulation capability, and electrochemical performance.

| Material | Structural characteristics | Coating method | Cycling performance (mAh g^-1^) | Electrolyte/sulfur ratio  (μL mg^-1^) | Ref. |
| --- | --- | --- | --- | --- | --- |
| AB/CoS_2_-MgS | heterostructure deposited on acetylene black | Doctor blade coating | 573.4  (600th, 5 C) | 5.6 | [52] |
| MnS/N-C@CNT | MnS nanoparticles and ZIF-8 derived N-doped carbon and CNT | Doctor blade coating | 500.8  (500th, 0.5 C) | / | [53] |
| WS_2_@C | Nanoflower structure | Doctor blade coating | 943  (500th, 1 C) | 10 | [54] |
| Ni-WS_2_@rGO-Celgard | Nanosheet structure | Doctor blade coating | 450.7  (500th, 1 C) | 40 | [55] |
| MWCNTs@N-doped C@CoS_2_ | Sandwich structure | Doctor blade coating | 725  (500th, 1 C) | 15 | [56] |
| CoS_2_-NC@CNTs | ZIF-67-derived carbon polyhedra and CNT network, uniformly distributed CoS_2_ nanoparticles | Vacuum filtration coating | 619.2  (500th, 1 C) | / | [57] |
| CoS_2_ HoMS | Hollow multi-shell structure (1S/2S/3S) | Vacuum filtration coating | 1001  (400th, 1 C) | / | [58] |
| CoS_2_-NiS_2_ | Hollow nanocage structure | Doctor blade coating | 676  (1000th, 2 C) | / | [59] |
| CoS_2_/ZnS | p-n junction structure supported on N-doped carbon nanosheets | Doctor blade coating | 586.3  (1000th, 2 C) | 8 | [60] |
| CoS_2_/NC@1T MoS_2_ | MOF-derived CoS_2_ and 1T MoS_2_ composite structure | Doctor blade coating | 1126.1  (200th, 0.5 C) | 15 | [61] |
| CoS_2_-MoS_2_/CNT | Heterointerface-induced defects and 1T MoS_2_ phase transition | Doctor blade coating | 616  (500th, 1 C) | 7 | [62] |
| P-Co_9_S_8_@SC | P-doped Co_9_S_8_ nanoparticles embedded in 3D spongy carbon | Vacuum filtration coating | 512  (900th, 1 C) | 30-80 | [63] |
| P-CoS_2_/CNTs | P-doped CoS_2_ nanoboxes and CNT network | Vacuum filtration coating | / | 20 | [64] |
| B-ZnS/CoS_2_@CS | B-doped ZnS/CoS_2_ heterojunction supported on carbon shell | Vacuum filtration coating | / | 8 | [65] |
| Cu-CoS_2_@NC | Cu-doped CoS_2_ nanocages, N-doped carbon shell | Doctor blade coating | / | / | [66] |
| CoS_2_-NC-CNTs@MoS_2_ | Hierarchical carbon nanoarchitecture | Doctor blade coating | 1129.3  (100th, 0.1 C) | 20 | [67] |
| CoS_2_-WS_2_/GNC | CoS_2_-WS_2_ heterostructure supported on g-C_3_N_4_ | Doctor blade coating | 1079  (1000th, 1 C) | 4 | [68] |
| CoS_2_-CoSe_2_ | In-situ sulfurization of CoSe_2_ nanoribbons to form CoS_2_-CoSe_2_ heterojunction | Doctor blade coating | 722  (350th, 0.5 C) | / | [69] |
| MCCoS/PP | MXene nanosheets loaded with CoS_2_ nanoparticles and CNT to construct multi-dimensional framework | Vacuum filtration coating | 368.6  (1000th, 20 C) | 7.5 | [70] |
| CoS_2_-NCA@C | CoS_2_ embedded in N/S doped carbon aerogel | Vacuum filtration coating | 1536.9  (600th, 1 C) | 10 | [71] |
| MnS/CNFs | MnS nanoparticles embedded in carbon nanofibers | Electrospinning | 810  (400th, 1 C) | 25 | [72] |
| MnS@N-C (yolk@shell) | yolk@shell structure | Doctor blade coating | 504.2  (1000th, 1 C) | 20 | [73] |
| Mn_3_O_4-x_ | Mn_3_O_4_ with surface oxygen vacancy regulation | Doctor blade coating | / | 8.4 | [74] |
| FeS_2_-MnS/C | FeS_2_-MnS heterostructure | Doctor blade coating | 768  (500th, 1 C) | / | [75] |
| Mo-doped MnS/MnO@C | Mo-doped MnS/MnO nanorods with carbon shell coating | Doctor blade coating | 1483.1  (500th, 2 C) | / | [76] |
| Mo_2_S_3_/MoS_2_@CNFs | Flower-like molybdenum sulfides on CNFs; in-situ formation of Mo_2_S_3_/MoS_2_ composite | Doctor blade coating | / | 5-8 | [77] |
| ZnS-SnS_2_@MoS_2_ | Hollow ZnS-SnS_2_ hetero-cubes as core, wrapped with ultrathin MoS_2_ nanosheets. | Doctor blade coating | 582  (500th, 2 C) | / | [78] |
| MoS_2_/Celgard | Highly conductive MoS_2_ nanosheets (Li-intercalation) forming a layer on Celgard. | Vacuum filtration coating | 401  (600th, 0.5 C) | / | [79] |
| 3D MoS_2_/Graphene Microsphere | 3D flower-like MoS_2_ microspheres wrapped by graphene | Doctor blade coating | 880  (100th, 0.2 C) | / | [80] |
| MoS_2_-NPs | Nanoparticles with edge defects, expanded interlayer and metallic 1T phase. | Doctor blade coating | 525  (150th, 0.2 C) | / | [81] |
| RGO-PANI/MoS_2_ (RPM) | Vertically aligned MoS_2_ arrays on RGO-PANI, forming MoS_2_/MoN heterointerface and “reservoir” structure. | Doctor blade coating | 827  (200th, 0.5 C) | 9 | [82] |
| rGO/MoS_2_/MoO_2_ | MoS_2_/MoO_2_ heterojunctions on rGO sheets with micron-sized pores, synthesized via rapid microwave method | Vacuum filtration coating | 929  (300th, 0.5 C) | / | [83] |
| Bi_2_S_3_-MoS_2_ | Nanoflower-like heterostructure with abundant Bi_2_S_3_-MoS_2_ interfaces | Vacuum filtration coating | 974  (100th, 0.2 C) | 9 | [84] |
| MoS_2_-WS_2_@MXene | MoS_2_-WS_2_ heterostructure nanosheets grown on MXene (Ti_3_C_2_T_x_). | Doctor blade coating | 638.6  (500th, 2 C) | / | [85] |
| MoS_2_@SiO_2_ | Hollow SiO_2_ nanotubes as core, uniformly coated with MoS_2_ nanoflowers as shell. | Doctor blade coating | 620  (300th, 1 C) | / | [86] |
| In-Vs-MoS_2_ | Inner-layer Indium doping with surface sulfur vacancies in MoS_2_ lattice. | Doctor blade coating | 1042  (100th, 0.5 C) | / | [87] |
| 1T-WS_2_ | Metallic 1T-WS_2_ with high conductivity and edge active sites | Vacuum filtration coating | 609.9  (1000th, 1 C) | 20 | [88] |
| C-WS_2_ | Bilayer structure | Doctor blade coating | 416  (1000th, 1 C) | / | [89] |
| WS_2_/CNTs | 3D network | Vacuum filtration coating | 754  (1000th, 5 C) | 15 | [90] |
| MXene@WS_2_ | Mott-Schottky heterojunction | Vacuum filtration coating | / | / | [91] |
| Ta_2_O_5_/WS_2_@rGO | Ta_2_O_5_ nanoparticles loaded on WS_2_@rGO | Doctor blade coating | 618  (900th, 2 C) | 6.0 | [92] |
| B-WS_2_@MXene | Nanosheet array | Vacuum filtration coating | / | 5.0 | [93] |

The PRI values of cobalt sulfide-, manganese sulfide-, molybdenum sulfide-, and tungsten sulfide-based separators are systematically summarized in **Tables S7-S10**, where four key descriptors, including energy contribution score (ECS), lean-electrolyte compatibility score (LECS), structural penalty score (SPS), and device validation score (DVS), are integrated to establish a comprehensive evaluation framework. The comparative analysis reveals the intrinsic trade-offs among different inorganic separator architectures, balancing electrochemical enhancement with practical application limitations. High PRI values originate from the synergistic contribution of energy enhancement, electrolyte efficiency, structural optimization, and device-level validation rather than catalytic activity alone.

**Table S7. Component analysis of the Practical Relevance Index (PRI) for representative** Cobalt Sulfide-based **separator systems.**

| System | ECS | LECS | SPS | DVS | PRI |
| --- | --- | --- | --- | --- | --- |
| MWCNTs@N-doped-C@CoS_2_/PP | 1.686 | 0.333 | / | 0.2 | / |
| P-Co_9_S_8_@SC/PP | 1.732 | 0.350 | 1.508 | 0.2 | 0.774 |
| CoS_2_-MoS_2_-CNT/PP | 0.867 | 0.250 | / | 0.2 | / |
| NSPCFS@CoS_2_@C-200 | 0.749 | 0.25 | 2.183 | 0.2 | -0.984 |
| NSPCF@CoS_2_@C-150 | 1.023 | 0.25 | / | 0.2 | / |
| N-MX-CoS_2_/PP | 0.738 | 0.299 | 1.672 | 0.2 | -0.435 |
| P-CoS_2_/CNTs@Celgard | 1.880 | 0.250 | / | 0.2 | / |
| CoS_2_@Montmorillonite/PP | 1.576 | 0.588 | 2.086 | 0.2 | 0.279 |
| CoS_2_/NC@1T MoS_2_/PP | 0.556 | 0.333 | 1.922 | 0.2 | -0.833 |
| CoS_2_-NC@CNTs/PP | 0.617 | 0.250 | 2.646 | 0.2 | -1.579 |
| CoS_2_/rGO/PP | 1.149 | 1.118 | 1.129 | 0.2 | 1.221 |
| CoS_2_-CoSe_2_/GN/PP | 0.673 | 0.333 | 1.461 | 0.2 | -0.255 |
| CNFs/CoS_2-X_/PP | 1.548 | 0.250 | 1.777 | 0.2 | 0.221 |
| B-ZnS/CoS_2_@CS/PP | 2.352 | 0.625 | 1.385 | 0.2 | 1.792 |
| All PRI values are derived from the unified evaluation framework proposed in this work, rather than directly reported in original literature. | | | | | |

**Table S8. Component analysis of the Practical Relevance Index (PRI) for representative** Manganese Sulfide-based **separator systems.**

| System | ECS | LECS | SPS | DVS | PRI |
| --- | --- | --- | --- | --- | --- |
| MnS/CNFs | 1.737 | 0.200 | 2.130 | 0.2 | 0.007 |
| FeS_2_-MnS/C | 1.636 | / | 2.897 | 0.2 | / |
| MnS/N-C@CNT | 1.142 | 0.165 | 1.407 | 0.2 | 0.100 |
| Mn_3_O_4-x_ | 2.248 | 0.595 | 1.558 | 0.2 | 1.485 |
| Mo-doped MnS/MnO@C | 2.782 | / | 2.926 | 0.2 | / |
| All PRI values are derived from the unified evaluation framework proposed in this work, rather than directly reported in original literature. | | | | | |

**Table S9. Component analysis of the Practical Relevance Index (PRI) for representative** Molybdenum Sulfide-based **separator systems.**

| System | ECS | LECS | SPS | DVS | PRI |
| --- | --- | --- | --- | --- | --- |
| D-MoWS_2_-CNF | 4.114 | 0.500 | / | 0.2 | / |
| HATM-NRs | 2.159 | 1.000 | 0.624 | 0.2 | 2.735 |
| MoO_x_/MoS_2_@CC | 2.293 | 0.417 | / | 0.2 | / |
| MoS_2_/C HMs | 1.689 | 0.417 | 1.356 | 0.2 | 0.949 |
| PVDF/C MoS_2_ | 1.500 | 0.500 | 2.121 | 0.2 | 0.079 |
| u-PF MoS_2_/TiN | 1.489 | 0.500 | 1.449 | 0.2 | 0.740 |
| SiO_2_@MoS_2_ | 1.294 | 0.333 | 1.049 | 0.2 | 0.779 |
| 3D MoS_2_ nanospheres | 1.514 | 0.333 | 1.140 | 0.2 | 0.908 |
| MoS_2_@NC (core-shell) | 1.514 | 0.333 | 0.964 | 0.2 | 1.083 |
| MoS_2_-NP coated | 1.314 | 0.333 | 1.871 | 0.2 | -0.023 |
| MoP-MoS_2_/PCNFs | 1.323 | 0.500 | 1.414 | 0.2 | 0.609 |
| NSCNTs/MoS_2_ | 1.107 | 0.333 | 1.732 | 0.2 | -0.092 |
| VGCF/MoS_2_ | 1.067 | 0.500 | 1.204 | 0.2 | 0.563 |
| MoP/MoS_2_@C | 1.329 | 0.500 | 1.414 | 0.2 | 0.118 |
| TiO_2_/C-MoS_2_ | 0.938 | 0.333 | / | 0.2 | / |
| MoS_2_/BCF | 1.041 | 0.250 | 4.025 | 0.2 | -2.887 |
| S@HCS/MoS_2_ | 1.071 | 0.250 | 1.225 | 0.2 | -0.067 |
| 1T-MoS_2_@CNT | 1.039 | 0.333 | / | 0.2 | / |
| MoS_2_/graphene | 1.177 | 0.333 | 1.673 | 0.2 | -0.430 |
| NMCS@MoS_2_/CNT | 1.135 | 0.250 | 0.922 | 0.2 | 0.138 |
| MoS_2_/NC@PP | 1.171 | 0.250 | 0.990 | 0.2 | 0.110 |
| MoS_2_-polymer | 1.084 | 0.333 | 0.632 | 0.2 | 0.491 |
| CNF/MoS_2_ | 1.163 | 0.333 | 1.884 | 0.2 | -0.731 |
| MoS_2_/Celgard | 0.894 | 0.250 | 0.620 | 0.2 | 0.357 |
| G@void/MoS_2_ | 0.652 | 0.250 | / | 0.2 | / |
| GO/MoS_2_ | 1.094 | 0.250 | 1.140 | 0.2 | -0.080 |
| MoS_2_@SnO_2_ | 1.581 | 0.556 | / | 0.2 | / |
| MF-GA-MoS_2_ | 0.927 | / | 1.612 | 0.2 | / |
| C@MoS_2_ | 0.930 | / | / | 0.2 | / |
| CNT@C@1T-MoS_2_ | 0.877 | / | 1.149 | 0.2 | / |
| Co-MoS_2_@NC | 1.047 | 0.125 | 0.837 | 0.2 | 0.046 |
| CoS_2_/NC@1T-MoS_2_ | 0.947 | 0.333 | 1.360 | 0.2 | -0.329 |
| MoS_2_/Al_2_O_3_@CNF | 1.586 | 1.042 | 1.643 | 0.2 | 1.028 |
| MoSe_2_-MoS_2_@CNT | 1.325 | 0.500 | 1.140 | 0.2 | 1.941 |
| MXene/MoS_2_ | 1.642 | 0.714 | 1.414 | 0.2 | 1.200 |
| O-MoS_2_/CNS | 1.640 | / | / | 1.0 | / |
| P-MoS_2_ (3D network) | 1.468 | 0.333 | 1.517 | 0.2 | 0.531 |
| MoS_2_/MoO_x_@CNT | 1.755 | 0.500 | 1.225 | 0.2 | 2.116 |
| Bi_2_S_3_-MoS_2_-PP | 1.549 | 0.556 | / | 0.2 | / |
| CoS_2_-MoS_2_-CNT | 1.309 | 0.714 | / | 0.2 | / |
| MoS_2_-SnO_2_ | 1.609 | 1.000 | 1.396 | 0.2 | 1.412 |
| NiS_2_/1T-MoS_2_ | 0.607 | 0.714 | / | 0.2 | / |
| rGO@MoS_2_ | 1.326 | 0.420 | 1.304 | 0.2 | 0.642 |
| ZnS-SnS_2_@MoS_2_ | 1.427 | / | 1.095 | 0.2 | / |
| 1T-MoS_2_@PC | 1.758 | 0.625 | 1.253 | 0.2 | 1.330 |
| CoP@MoS_2_ | 1.554 | 0.500 | / | 0.2 | / |
| In-VS-MoS_2_ | 3.319 | / | 0.762 | 0.2 | / |
| MoSe_2_/MoS_2_@CNT | 1.432 | 0.476 | / | 0.2 | / |
| MoS_2_/MoO_2_-NCS | 2.746 | 0.833 | 1.688 | 0.2 | 2.091 |
| M-TiN/MoS_2_ | 2.194 | 0.500 | / | 0.2 | / |
| ZnS-NC@MoS_2_ | / | 0.424 | / | 0.2 | / |
| All PRI values are derived from the unified evaluation framework proposed in this work, rather than directly reported in original literature. | | | | | |

**Table S10. Component analysis of the Practical Relevance Index (PRI) for representative** Tungsten Sulfide-based **separator systems.**

| System | ECS | LECS | SPS | DVS | PRI |
| --- | --- | --- | --- | --- | --- |
| WS_2_@C-PP | 0.561 | 0.500 | / | 0.2 | / |
| C-WS_2_ | 0.672 | 0.500 | 1.513 | 0.2 | -0.141 |
| WS_2_-coated Celgard | 0.610 | / | 3.000 | 0.2 | -2.190 |
| WS_2_/CNTs | 0.774 | 0.333 | 0.762 | 0.2 | 0.545 |
| Ni-WS_2_@rGO-Celgard | 0.685 | 0.125 | 1.562 | 0.2 | -0.552 |
| 1T-WS_2_/rGO | 0.612 | 0.299 | / | 0.2 | / |
| MXene@WS_2_ | 0.523 | / | / | 0.2 | / |
| CoS_2_-WS_2_/GNC-PP | 0.618 | / | 1.342 | 0.2 | -0.524 |
| Ta_2_O_5_/WS_2_@rGO-PP | 0.614 | / | 1.794 | 0.2 | -0.980 |
| B-WS_2_@MXene-PP | 0.734 | 1.000 | / | 1 | / |
| All PRI values are derived from the unified evaluation framework proposed in this work, rather than directly reported in original literature. | | | | | |

**Table S11.** Representative metal nitride-based separator coating materials applied in Li-S batteries, summarizing their catalytic properties and corresponding electrochemical performances.

| Material | Structural characteristics | Coating method | Cycling performance  (mAh g^-1^) | Electrolyte/sulfur ratio  (μL mg^-1^) | Ref. |
| --- | --- | --- | --- | --- | --- |
| NC-Ti_0.95_Co_0.05_N NWs | N-doped carbon-embedded, Co-doped TiN nanowires. Co- doping introduces ferromagnetism and upshifts d-band center. | Doctor blade coating | 464  (200th, 0.5 C) | 25 | [94] |
| VN@NC | VN quantum dots anchored on N-doped carbon nanosheets | Doctor blade coating | 726  (1000th, 2 C) | 4.6 | [95] |
| e-TiN | Commercial TiN nanoparticles etched by HF. | Doctor blade coating | 660  (200th, 0.5 C) | 20 | [96] |
| Mesoporous TiN Microspheres (MTN) | Mesoporous TiN microspheres with high conductivity. | Doctor blade coating | 744  (200th, 0.5 C) | 56 | [97] |
| MMC-TiN | TiN nanoparticles synergetic with micro-/mesoporous carbon | Doctor blade coating | 500  (400th, 1 C) | 50 | [98] |
| TiN-C^65^ | Nano-sized TiN mixed with Super C^65^ carbon. | Doctor blade coating | 390  (1000th, 0.5 C) | / | [99] |
| TiN/NG | Nano-sized Ti mixed with Super C^65^ carbon. | Vacuum filtration coating | 718  (300th, 1 C) | 60 | [100] |
| BNC-TiN-BNC | Sandwich structure | Electrospinning | 594  (300th, 2 C) | 80 | [101] |
| CCC@TiO_2_-TiN | Self-standing carbonized cotton cloth (CCC) decorated with TiO_2_-TiN heteronanowires. | Doctor blade coating | / | 4.6 | [102] |
| TiO_2_-TiN@CNPs | N-doped porous carbon nanoplates embedded with ultrafine TiO_2_-TiN heterojunctions | Doctor blade coating | 1147  (300th, 0.2 C) | 35 | [103] |
| 2D Ultrathin TiN NSs | 2D ultrathin TiN nanosheets | Vacuum filtration coating | 463  (1000th, 1 C) | / | [104] |
| TiN@C/G | 2D TiN@C sheets | Vacuum filtration coating | 635.8  (600th, 1 C) | 56 | [105] |
| TiN/1T-MoS_2_ | Hollow TiN nanospheres decorated with metallic, few-layered 1T-MoS_2_ nanosheets (heterostructure) | Doctor blade coating | 459  (800th, 1 C) | 25 | [106] |
| TiN/Nb_4_N_5_ | Spherical TiN/Nb_4_N_5_ heterostructure (from nitridation of TiNb_2_O_7_ microspheres), with a rough, porous surface | Doctor blade coating | 663.2  (200th, 0.2 C) | / | [107] |
| TMN@CNF | Flexible | Electrospinning | 960  (100th, 2 C) | 36 | [108] |
| L-TiN-Cu-CNF | Low-crystalline TiN film coated on Cu-embedded carbon nanofibers (Cu-CNFs) via PVD | Electrospinning | 626  (300th, 0.5 C) | 15 | [109] |
| TiN/CoO (TCCNT) | N-doped carbon nanotubes with dispersed TiN/CoO particles | Vacuum filtration coating | 732  (500th, 2 C) | 10 | [110] |
| CoFe/FeN_0.0324_/TiN@N-CNT | Multicomponent heterostructure nanoparticles | Doctor blade coating | 708  (400th, 0.5 C) | / | [111] |
| M-TiN/MoN | 2D-2D TiN/MoN heterostructure derived from MXene/MoS_2_ | / | 490  (500th, 1 C) | 10 | [112] |
| MoN-G | MoN nanosheets on highly conductive graphene | Vacuum filtration coating | 665  (1500th, 1 C) | / | [113] |
| MoN@CNFs | Thorn-like carbon nanofibers with highly exposed MoN nanosheets on the surface | Doctor blade coating | 531  (500th, 2 C) | / | [114] |
| S-MN@GNs | MoN nanolayer decorated on graphene nanosheets via magnetron sputtering. | Doctor blade coating | 789  (700th, 0.5 C) | 15 | [115] |
| Co/MoN | Petal-shaped Co/MoN nanoparticle composite with heterointerfaces. | Doctor blade coating | 554.3  (500th, 1 C) | 10 | [116] |
| MONS | MoO_2_ nanosheets coated on a pristine separator. | Doctor blade coating | / | / | [117] |
| POF | Coral-like, fiber-shaped zwitterionic porous organic framework with sulfophilic and lithiophilic moieties. | Doctor blade coating | 654  (500th, 1 C) | / | [118] |
| SA-Co/NGM | Atomically dispersed single Co atoms with planar Co-N_4_ coordination on an ultrathin N-doped graphene mesh | Doctor blade coating | 731  (1000th, 2 C) | 6.8-10.5 | [119] |
| VN/NG | VN nanoparticles on N-doped graphene nanosheets | Doctor blade coating | 863  (300th, 1 C) | / | [120] |
| VN/PVDF-PMMA/hBN | Sandwich-type | Electrospinning | 905.1  (200th, 0.2 C) | / | [121] |
| VN/Mo_2_C@CNS | VN/Mo_2_C heterointerfaces embedded in N-doped carbon nanosheets | Doctor blade coating | 558.8  (1000th, 1 C) | / | [122] |
| VN-MoC@PC | VN-MoC heterogeneous nanoparticles in 3D porous carbon | Doctor blade coating | 901.8  (300th, 1 C) | / | [123] |
| VN@NC | VN nanocrystals in hierarchically porous, ultrathin N-doped carbon nanosheets | Doctor blade coating | 712.1  (500th, 1 C) | 8 | [124] |
| VN-V_2_O_3_@rGO | VN-V_2_O_3_ heterostructure nanoparticles wrapped in rGO | Doctor blade coating | 685.1  (1000th, 2 C) | / | [125] |
| Co_4_N | Ultrafine Co_4_N nanocrystals | Doctor blade coating | 381  (400th, 4 C) | 10-15 | [126] |
| CNT-CoN-VN | CoN with nitrogen vacancies grown on multi-walled CNTs | Doctor blade coating | 660  (250th, 2 C) | 20 | [127] |
| Co-Fe_3_N | Mott-Schottky type Co-Fe_3_N heterostructure with regular octahedral shape | Doctor blade coating | 534  (400th, 1 C) | / | [128] |
| CoN@NCNT | CoN nanoparticles encapsulated in N-doped carbon nanotubes | Doctor blade coating | 558.7  (800th, 1 C) | / | [129] |
| CoN/Co_9_S_8_@RGO | CoN and Co_9_S_8_ nanoparticles on RGO (used as both S host and in interlayer) | Doctor blade coating | 425  (700th, 1 C) | / | [130] |
| SA-Fe/Fe_2_N@NG | Single-atom Fe (SA-Fe) and Fe_2_N nanocrystals co-decorated on N-doped graphene | Doctor blade coating | 801.3  (500th, 1 C) | 10-15 | [131] |
| Fe_2_N/N-rGO | Fe_2_N nanoparticles evenly dispersed on N-doped rGO | Doctor blade coating | 659  (700th, 1 C) | / | [132] |
| Ni_3_N/Nb_4_N_5_ | Ni_3_N/Nb_4_N_5_ heterostructure (from nitridation of NiNb_2_O_6_ nanorods) | Doctor blade coating | 796.2  (150th, 2 C) | / | [133] |

The PRI values of titanium nitride-, molybdenum nitride-, vanadium nitride-, and other nitrides -based separators are systematically summarized in **Tables S12-S15**, where four key descriptors, including energy contribution score (ECS), lean-electrolyte compatibility score (LECS), structural penalty score (SPS), and device validation score (DVS), are integrated to establish a comprehensive evaluation framework. The comparative analysis reveals the intrinsic trade-offs among different inorganic separator architectures, balancing electrochemical enhancement with practical application limitations. High PRI values originate from the synergistic contribution of energy enhancement, electrolyte efficiency, structural optimization, and device-level validation rather than catalytic activity alone.

**Table S12. Component analysis of the Practical Relevance Index (PRI) for representative** Titanium Nitride-based **separator systems.**

| System | ECS | LECS | SPS | DVS | PRI |
| --- | --- | --- | --- | --- | --- |
| MTN (Mesoporous TiN microspheres) | 0.704 | 0.109 | 1.414 | 0.2 | -0.402 |
| MMC-TiN | 0.400 | 0.088 | / | 0.2 | / |
| μFGF-MoS_2_/C-TiN | 1.143 | 0.500 | 1.897 | 0.2 | -0.054 |
| BNC-TiN-BNC | 1.039 | 0.063 | 2.002 | 0.2 | -0.723 |
| TiN/NG | 0.662 | 0.053 | / | 0.2 | / |
| 2D TiN@C | / | 0.089 | 1.086 | 0.2 | / |
| h-ITO@PP | 1.127 | 0.125 | 0.791 | 0.2 | 0.661 |
| NC/TiN NWs@PP | 0.776 | 0.500 | 1.803 | 0.2 | -0.327 |
| TiN/1T-MoS_2_ | 2.409 | 0.200 | / | 0.2 | / |
| TiN/Nb_4_N_5_ | 0.607 | / | 2.864 | 0.2 | / |
| TiN/PI | 1.314 | 0.833 | / | 0.2 | / |
| TMN@CNF | 0.669 | 0.333 | 2.121 | 0.2 | -0.919 |
| TiN/CoO (TCCNT) | 0.627 | 0.500 | / | 0.2 | / |
| TiO_2_-TiN | 1.460 | 0.833 | 1.369 | 0.2 | 1.124 |
| L-TiN-Cu-CNF | 1.372 | 0.500 | 1.446 | 0.2 | 0.626 |
| 2D TiN NSs | 1.587 | 0.667 | / | 1.0 | / |
| M-TiN/MoN | 1.952 | 0.500 | / | 0.2 | / |
| CoFe/FeN_0.0324_/TiN@N-CNT | 0.978 | / | 0.809 | 0.2 | / |
| GPC-TiN-10 | 1.586 | 0.833 | 2.387 | 0.2 | 0.232 |
| TiC-TiN-TiO_2_ | 1.460 | 0.833 | 1.369 | 0.2 | 1.124 |
| All PRI values are derived from the unified evaluation framework proposed in this work, rather than directly reported in original literature. | | | | | |

**Table S13. Component analysis of the Practical Relevance Index (PRI) for representative** Molybdenum Nitride-based **separator systems.**

| System | ECS | LECS | SPS | DVS | PRI |
| --- | --- | --- | --- | --- | --- |
| MoN-G/PP | 1.844 | 0.250 | 1.304 | 0.2 | 0.990 |
| CCC@MoN | 1.883 | 0.250 | / | 0.2 | / |
| MoN@CNFs/PP | 1.003 | 0.250 | 0.917 | 0.2 | 0.536 |
| Co/MoN@P | 1.592 | 0.500 | 0.923 | 0.2 | 1.369 |
| SA-Co/NGM@PP | 1.707 | 0.735 | 0.748 | 0.2 | 1.894 |
| M-TiN/MoN@PP | 1.588 | 0.500 | 0.809 | 0.2 | 1.479 |
| All PRI values are derived from the unified evaluation framework proposed in this work, rather than directly reported in original literature. | | | | | |

**Table S14. Component analysis of the Practical Relevance Index (PRI) for representative** Vanadium Nitride-based **separator systems.**

| System | ECS | LECS | SPS | DVS | PRI |
| --- | --- | --- | --- | --- | --- |
| VN Quantum Dots@N-doped Carbon Nanosheets (VN@NC) | 1.973 | 1.087 | 0.574 | 1 | 3.486 |
| VN (Porous Nanobelts) | 1.533 | 0.500 | / | 0.2 | / |
| VN/NG (Nanoparticles on N-doped Graphene) | 2.054 | 0.500 | / | 0.2 | / |
| VN (Porous, from MAX phase) | 1.734 | 0.625 | 1.487 | 0.2 | 1.072 |
| VN (Multi-Branched, MB-VN) | 1.377 | 0.476 | 1.871 | 0.2 | 0.182 |
| VN/rGO | 0.615 | 0.125 | 1.581 | 0.2 | -0.641 |
| VN@NC (Nanocrystals in Porous Carbon Nanosheets | 1.969 | 0.833 | / | 0.2 | / |
| FeN_4_-O-NC-VN | 0.958 | 0.250 | 1.803 | 0.2 | -0.395 |
| VN/Mo_2_C@CNS | 0.578 | 0.250 | / | 0.2 | / |
| PEG-VN (PEG-VN@PP) | 1.716 | 0.500 | / | 0.2 | / |
| VN-MoC@PC | 0.889 | 0.333 | 0.632 | 0.2 | 0.790 |
| VN-V_2_O_5_@rGO | 1.959 | 0.500 | 1.378 | 0.2 | 1.281 |
| All PRI values are derived from the unified evaluation framework proposed in this work, rather than directly reported in original literature. | | | | | |

**Table S15. Component analysis of the Practical Relevance Index (PRI) for representative** other Nitride-based **separator systems.**

| System | ECS | LECS | SPS | DVS | PRI |
| --- | --- | --- | --- | --- | --- |
| Li-CON@GN/Celgard | 0.670 | 0.250 | / | 0.2 | / |
| CoN-CNT/HPC | 1.669 | / | 1.817 | 0.2 | / |
| CNT-CoN-VN | 1.849 | 0.250 | 1.225 | 0.2 | 1.074 |
| Co_2_P/Co/C | 1.152 | 0.167 | / | 0.2 | / |
| Co_4_N-Celgard | 1.300 | 0.333 | / | 02 | / |
| SNP-PC@MPC@CoSe | 2.171 | 1.250 | 1.353 | 0.2 | 2.268 |
| CoN@NCNT | 0.666 | 0.250 | / | 0.2 | / |
| Co-N-C@CeO_2_-C | 1.700 | / | / | 0.2 | / |
| CoN-Mo_2_N | 0.877 | / | / | 0.2 | / |
| Ni_3_N/Nb_4_N_5_ | 0.786 | 0.250 | 2.065 | 0.2 | -0.829 |
| N_i3_FeN | 1.820 | 1.064 | / | 0.2 | / |
| Co-Ni_3_N-CNFs | 0.664 | / | / | 0.2 | / |
| Co-Fe_3_N/PP | 1.039 | 0.179 | / | 0.2 | / |
| Fe_2_N/N-rGO | 2.073 | 0.909 | 1.118 | 0.2 | 2.064 |
| Fe_3_C/Fe_3_N@NCNT | 2.060 | / | / | 0.2 | / |
| SA-Fe/Fe_3_N@NG | 2.004 | / | / | 0.2 | / |
| All PRI values are derived from the unified evaluation framework proposed in this work, rather than directly reported in original literature. | | | | | |

**Table S16.** Representative MXene-based separator modification strategies for Li-S batteries, summarizing structural design features and their corresponding electrochemical performances.

| Material | Structural characteristics | Coating method | Cycling performance (mAh g^-1^) | Electrolyte/sulfur ratio  (μL mg^-1^) | Ref. |
| --- | --- | --- | --- | --- | --- |
| L-MXene | Few-layered MXene nanosheets with increased interlayer spacing. | Doctor blade coating | 559  (500th, 1 C) | / | [134] |
| Tannic Acid-MXene | Ultrathin layer with sub-nanometer interlayer channels. | Vacuum filtration coating | / | 10 | [135] |
| Porous Silica Nanosheet/MXene | 2D nanochannel interlayer with cation-selective transport. | Doctor blade coating | / | 3.9 | [136] |
| PVDF-MXene/TMO | Electrospun PVDF nanofibers embedded with MXene/TMO hybrids. | Electrospinning | 1032  (100th, 0.1 C) | 20 | [137] |
| MXene/SiO_2_ Hybrid Aerogel | 3D hierarchical porous network of SiO_2_ cross-linked with MXene. | Doctor blade coating | 623  (200th, 0.5 C) | / | [138] |
| Ag-ZnSe@MX | Ag-doped ZnSe nanodots anchored on MXene nanosheets. | Doctor blade coating | 1017.9  (1000th, 2 C) | / | [139] |
| Vo-LDHs-MXenes | NiCo-LDHs nanosheets with oxygen vacancies anchored on MXene. | Doctor blade coating | 701  (300th, 1 C) | 6.0 | [140] |
| N, S co-doped MXene | Wrinkled, N and S co-doped functionalized MXene. | Vacuum filtration coating | 716  (300th, 1 C) | 7.0 | [141] |
| Loadine-doped MXene | 2D layered structure with iodine doping. | Vacuum filtration coating | 761  (100th, 0.2 C) | 15/10 | [142] |
| Oxygen-dominated MXene | Porous carbon nanofiber inlaid with O-dominated MXene. | Electrospinning | / | 6.0 | [143] |
| Fluorine-free MXene with TiN | F-free MXene with Ti coordinated to N, encapsulated in porous N-doped carbon | Doctor blade coating | 889.5  (100th, 0.5 A g^-1^) | / | [144] |
| Cu SA/N-Ti_3_C_2_Tx | Asymmetrically coordinated Cu-N_1_C_2_ single atoms on N-doped MXene. | Doctor blade coating | / | / | [145] |
| CoSA-CoNC/NMX | Co single atoms (CoN_1_C_3_) and nanoclusters on N-doped MXene. | Doctor blade coating | 558  (1000th, 2 C) | 4.5 | [146] |
| MXene@CeSA | Ce single atoms anchored on MXene matrix via MOF-derived method. | Doctor blade coating | 543  (1000th, 1 C) | 7 | [147] |
| Ni_0.7_Co_0.3_S_2_/MXene | Bimetallic sulfide grown in-situ on MXene nanosheets. | Vacuum filtration coating | 437  (1500th, 1 C) | 15 | [148] |
| SnSe_2_@Ti_3_C_2_Tx MXene | SnSe_2_ nanosheets anchored on MXene. | Doctor blade coating | 751  (120th, 0.2 C) | 25/6 | [149] |
| SnO_2_ QDs/MXene | 0D-2D heterostructure with SnO_2_ quantum dots on MXene. | Doctor blade coating | / | 10 | [150] |
| 1T-VSe_2_@Ti_3_C_2_ | Metallic 1T-VSe_2_ anchored on MXene with a built-in electric field. | Doctor blade coating | 1034  (100th, 0.2 C) | / | [151] |
| TiC-TiN-TiO_2_ | Ternary accordion heterostructure from eutectoid reaction of Ti_3_CN MXene. | Doctor blade coating | 722  (500th, 0.5 C) | 20/6 | [152] |
| MXene/ZIF-67 Janus Separator | ZIF-67 on cathode side, MXene on anode side. | Vacuum filtration coating | 791  (100th, 0.5 C) | / | [153] |
| CACNM@PP | Co-Ni/MXene (cathode) and Cu-Ag/MXene (anode). | Doctor blade coating | 477  (1500th, 5 C) | 8 | [154] |
| Ti_3_C_2_Tx MXene | Pristine MXene used as both cathode scaffold and separator coating. | Doctor blade coating | / | 5 | [155] |
| MXene@COF | COF in-situ grown on MXene surface via Ti-N bonds. | Doctor blade coating | 872.9  (1000th, 1 C) | / | [156] |
| OMC-g-MXene | Ordered mesoporous carbon grafted onto MXene nanosheets. | Doctor blade coating | 770  (800th, 1 C) | 7.7 | [157] |

The PRI values of MXene-based separators are systematically summarized in **Tables S17**, where four key descriptors, including energy contribution score (ECS), lean-electrolyte compatibility score (LECS), structural penalty score (SPS), and device validation score (DVS), are integrated to establish a comprehensive evaluation framework. The comparative analysis reveals the intrinsic trade-offs among different inorganic separator architectures, balancing electrochemical enhancement with practical application limitations. High PRI values originate from the synergistic contribution of energy enhancement, electrolyte efficiency, structural optimization, and device-level validation rather than catalytic activity alone.

**Table S17. Component analysis of the Practical Relevance Index (PRI) for representative** MXene-based **separator systems.**

| System | ECS | LECS | SPS | DVS | PRI |
| --- | --- | --- | --- | --- | --- |
| CM/MoS_2_ (Crumpled MXene/MoS_2_) | 1.717 | 0.714 | 0.894 | 0.2 | 1.737 |
| L-MXene | 0.489 | 0.250 | 0.574 | 0.2 | 0.365 |
| TA-MXene | 1.494 | 0.500 | 0.358 | 0.2 | 1.836 |
| PSN/MXene 2D | 1.688 | 0.333 | 1.039 | 0.2 | 1.182 |
| MXene/MoS_2_/SnS@C | 1.615 | 0.676 | 1.183 | 0.2 | 1.308 |
| Ag-ZnSe@MXene | 0.835 | 0.250 | 1.265 | 0.2 | 0.020 |
| Ni_0.7_Co_0.3_S_2_/MXene | 1.572 | 0.667 | 1.114 | 0.2 | 1.325 |
| 1T-VSe_2_@Ti_3_C_2_ | 0.894 | 0.200 | 1.049 | 0.2 | 0.245 |
| Ti_3_C_2_T_x_@S Ti_3_C_2_T_x_/PP | 1.566 | 1.000 | 0.954 | 1.0 | 2.712 |
| CoSA-CoNC/NMX | 2.248 | 1.111 | 2.025 | 0.2 | 1.534 |
| Ti_3_C_2_ MXene | 2.062 | 0.500 | 1.549 | 1.0 | 2.013 |
| CoNi@TiO_2_/C | 1.579 | 0.250 | 1.871 | 0.2 | 0.158 |
| Co-CoP@MX | 1.956 | 0.333 | 1.265 | 0.2 | 1.224 |
| Zn-Co-Ni-S@MXene@CNTs | 0.971 | 0.250 | 1.304 | 0.2 | 0.117 |
| Ti_3_C_2_T_x_-UIO-66-NH_2_ | 1.530 | 0.250 | 1.581 | 0.2 | 0.399 |
| NiCoSAA-MXene | 1.262 | 0.333 | 1.272 | 0.2 | 0.523 |
| NiS_2_@MXene | 1.640 | 0.250 | 1.311 | 0.2 | 0.779 |
| SnSe_2_@Ti_3_C_2_T_x_ MXene | 1.537 | 0.833 | 1.624 | 0.2 | 0.946 |
| O-Ti_2.7_Nb_0.3_C_2_T_x_ | 1.382 | 0.500 | 0.894 | 0.2 | 1.188 |
| Co-VC | 2.089 | 1.250 | 0.922 | 0.2 | 2.617 |
| Co_3_Fe_7_-MXene | 1.356 | 1.250 | 0.922 | 0.2 | 1.884 |
| Ti_3_C_2_T_x_ MXene@CuCo_2_O_4_ | 2.247 | 1.923 | 1.095 | 0.2 | 3.275 |
| Co/CoN_4_@KB-MXene | 1.965 | 0.820 | 1.049 | 0.2 | 1.936 |
| MXene@TBCOF | 2.031 | 0.500 | 1.746 | 0.2 | 0.985 |
| MXene-LDH | 1.632 | 0.167 | 0.922 | 0.2 | 1.007 |
| MXene/NiS_2_/Co_3_S_4_ | 2.047 | 0.333 | 1.549 | 0.2 | 1.031 |
| NiTe_2_@MXene | 1.880 | 0.769 | 1.789 | 0.2 | 1.060 |
| CACNM@PP | 1.218 | 0.625 | 1.183 | 0.2 | 0.860 |
| S/rGO@Ti_3_C_2_ QDs | 1.353 | 0.833 | 0.837 | 0.2 | 1.549 |
| Vo-LDHs-MXenes | 1.702 | 0.833 | 0.894 | 0.2 | 1.841 |
| ZnS/MoS_2_@MXene | 1.543 | 0.333 | 1.732 | 0.2 | 0.344 |
| SnO_2_ QDs/MXene | 1.600 | 0.500 | 0.359 | 0.2 | 1.941 |
| Cu SA/N-Ti_3_C_2_T_x_ | 1.673 | / | 1.000 | 0.2 | / |
| BN@MXene | 1.047 | 1.163 | 0.227 | 0.2 | 2.183 |
| Ti_3_C_2_T_x_@CoSe_2_ | 1.230 | 0.556 | 1.342 | 0.2 | 0.644 |
| CNVM (N, V-codoped MXene) | 1.490 | 0.500 | 1.396 | 0.2 | 0.794 |
| Co_3_Se_4_@N-C/Ti_3_C_2_T_x_ | 1.149 | / | 1.225 | 0.2 | / |
| Fe_3_Se_4_/FeSe@MXene | 1.623 | 1.471 | 1.265 | 0.2 | 2.029 |
| NiS_2_/MXene | 1.764 | 0.625 | 1.789 | 0.2 | 0.800 |
| Ti_3_C_2_T_x_-CoBDC | 2.061 | 0.250 | 0.714 | 0.2 | 1.797 |
| Co_1-x_S/3D-Ti_3_C_2_T_x_ | 1.686 | 1.064 | / | 0.2 | / |
| Pt SAs/In_2_S_3_/Ti_3_C | 1.654 | 0.500 | 1.300 | 0.2 | 1.054 |
| Ti_3_C_2_T_x_/Ni-Co MOF | 1.632 | 1.250 | 0.949 | 0.2 | 2.133 |
| Ti_3_C_2_/CPNC (CoP) | 2.086 | 0.625 | 1.581 | 0.2 | 1.330 |
| V_4_C_3_T_x_ | 1.294 | 0.500 | 1.000 | 0.2 | 0.994 |
| N-MX-CoS_2_ | 0.707 | 0.250 | 1.581 | 0.2 | -0.424 |
| V_2_C/V_2_O_5_/CNTs | 2.094 | 1.136 | 1.378 | 0.2 | 2.052 |
| Ti_3_C_2_T_x_/CNTs-PP | 0.663 | / | 0.131 | 0.2 | / |
| N-Ti_3_C_2_/C@PP | 1.170 | 0.625 | 1.049 | 0.2 | 0.946 |
| All PRI values are derived from the unified evaluation framework proposed in this work, rather than directly reported in original literature. | | | | | |

**References**

1. N. Shi, B. Xi, Z. Feng, F. Wu, D. Wei, J. Liu and S. Xiong, “Insight into Different-Microstructured Zno/Graphene-Functionalized Separators Affecting the Performance of Lithium-Sulfur Batteries,” *Journal of Materials Chemistry A* 7 (2019): 4009-4018, https://doi.org/10.1039/C8TA12409D.

2. Y. Zhao, M. Liu, W. Lv, Y.B. He, C. Wang, Q. Yun, B. Li, F. Kang and Q.H. Yang, “Dense Coating of Li_4_Ti_5_O_12_ and Graphene Mixture on the Separator to Produce Long Cycle Life of Lithium-Sulfur Battery,” *Nano Energy* 30 (2016): 1-8, https://doi.org/10.1016/j.nanoen.2016.09.030.

3. N. Liu, L. Wang, T. Tan, Y. Zhao and Y. Zhang, “TiO_2_/GO-Coated Functional Separator to Suppress Polysulfide Migration in Lithium-Sulfur Batteries,” *Beilstein Journal of Nanotechnology* 10 (2019): 1726-1736, https://doi.org/10.3762/bjnano.10.168.

4. Y. Wan, X. Ma, J. Hao, H. Min, H. You, X. Liu and H. Yang, “Titanium-Based Nanorods/Ketjen Black Modified Separator as Polysulfides Barrier for Lithium Sulfur Cell,” *Journal of Alloys and Compound*s 842 (2020): 155830, https://doi.org/10.1016/j.jallcom.2020.155830.

5. P. Chen, Z. Wang, B. Zhang, H. Liu, W. Liu, J. Zhao, Z. Ma, W. Dong and Z. Su “Reduced Graphene Oxide/TiO_2_(B) Nanocomposite-Modified Separator as an Efficient Inhibitor of Polysulfide Shuttling in Li-S Batteries,” *RSC Advances* 10 (2020): 4538-4544, https://doi.org/10.1039/C9RA10185C.

6. T. Zhou, W. Lv, J. Li, G. Zhou, Y. Zhao, S. Fan, B. Liu, B. Li, F. Kang and Q.H. Yang, “Twinborn TiO_2_-Tin Heterostructures Enabling Smooth Trapping-Diffusion-Conversion of Polysulfides Towards Ultralong Life Lithium-Sulfur Batteries,” *Energy & Environmental Science* 10 (2017): 1694-1703, https://doi.org/10.1039/C7EE01430A.

7. H.D. Chen, Y.C. Qiu, Z.Y. Cai, W.H. Liang, L. Liu, M.M. Li, X.H. Hou, F.M. Chen, X.Z. Zhou, T.F. Cheng, L.Q. He, J.Z. Wang, X. Zhang, S.X. Dou and L. Li, “Topological Insulator Heterojunction with Electric Dipole Domain to Boost Polysulfide Conversion in Lithium-Sulfur Batteries,” *Angewandte Chemie-International Edition* 64 (2025): e202423357, https://doi.org/10.1002/anie.202423357.

8. K.C. Li, X. Chen, A. Sabbaghi, C.H. Wong, C.y. Tang, F.L.Y. Lam and X. Hu, “Single-Step Synthesis of Titanium Nitride-Oxide Composite and Ai-Driven Aging Forecast for Lithium-Sulfur Batteries,” *Journal of Materials Chemistry A* 12 (2024): 9017-9030, https://doi.org/10.1039/D4TA00234B.

9. Z.H. Li, C. Zhou, J.H. Hua, X.F. Hong, C.L. Sun, H.W. Li, X. Xu and L.Q. Mai, “Engineering Oxygen Vacancies in a Polysulfide-Blocking Layer with Enhanced Catalytic Ability,” Advanced Materials 32 (2020): 1907444, https://doi.org/10.1002/adma.201907444.

10. L. Ma, Y. Zhang, S. Zhang, L. Wang, C. Zhang, Y. Chen, Q. Wu, L. Chen, L. Zhou and W. Wei, “Integrating Energy Band Alignment and Oxygen Vacancies Engineering of TiO_2_ Anatase/Rutile Homojunction for Kinetics-Enhanced Li-S Batteries,” *Advanced Functional Materials* 33 (2023): 2305788, https://doi.org/10.1002/adfm.202305788.

11. X. Du, D. Ma, Y. Zhang, J. Ma, J. Wang, Q. Xiao, B. Wang, L. Tian and J. Zhuang, “Electrospun TiO_2_ Nanofibers Featuring Surface Oxygen Vacancies as a Multifunctional Interlayer for High-Performance Lithium-Sulfur Batteries in a Wide Temperature Range,” *Inorganic Chemistry* 62 (2023): 5134-5144, https://doi.org/10.1021/acs.inorgchem.2c04417.

12. Z. Zhao, X. Duan, L. Zhang, Z. Che, K. Wang, B. Zheng and X. Wang, “Elevated Electrochemical Performances Enabled by a Core-Shell Titanium Hydride Coated Separator in Lithium-Sulphur Batteries,” *RSC Adv* 11 (2021): 30755-30762, https://doi.org/10.1039/d1ra04281e.

13. X. Fan, H. Liu, H. Wang, W. Hu, X. Tian and L. Pang, “Understanding the Synergistic Improving Adsorbed and Catalytic Activity of Polysulfide by Design of Oxygen Vacancies and Li^+^ Lithiation/Delithiation Behavior in Titanium Oxide Separator,” *Electrochimica Acta* 485 (2024): 144142, https://doi.org/10.1016/j.electacta.2024.144142.

14. A. Chen, W. Liu, J. Yan and K. Liu, “A Novel Separator Modified by Titanium Dioxide Nanotubes/Carbon Nanotubes Composite for High Performance Lithium-Sulfur Batteries,” *Functional Materials Letters* 12 (2019): 1950016, https://doi.org/10.1142/s1793604719500164.

15. A. Ansari Hamedani, B. Cetiner, B. Yarar Kaplan, S. Alkan Gürsel and A. Yürüm, “Nanofibrous Interlayers with Ultrafine Decoration of Titanium Oxide to Intercept Lithium Polysulfides in Lithium-Sulfur Batteries,” *ChemPhysChem* 26 (2025): e202401136, https://doi.org/10.1002/cphc.202401136.

16. S. Liang, S. Chauque, M. Ricci and R.P. Zaccaria, “Enhancing Lithium-Sulfur Battery Performance through Electronic/Ionic Co-Conductive Multi-Walled Carbon Nanotubes/Lithium Lanthanum Titanium Oxide Separator Modification,” *Materials Today Energy* 40 (2024): 101497, https://doi.org/10.1016/j.mtener.2024.101497.

17. J. Wang, X. Zhang, J. Liu, X. Wang, Y. Nie, K. Zong, X. Zhang, C. Zhao, L. Yang, X. Wang and Z. Chen, “High-Spin Cobalt Enables Strong Metal-Sulfur Orbital Hybridization for Accelerated Polysulfide Conversion in Lithium-Sulfur Batteries,” *Advanced Materials* 37 (2025): 2502075, https://doi.org/10.1002/adma.202502075.

18. M. Qian, Y. Tang, X. Li, Y. Zhang, W. Jiang and L. Liu, “Regulating the Proximity of D-P Band Center in TiP_2_O_7_ by the Crystalline-Amorphous Heterointerface to Boost Adsorption-Catalysis for LiPSs in Li-S Batteries,” *Journal of Energy Chemistry* 108 (2025): 458-467, https://doi.org/10.1016/j.jechem.2025.04.030.

19. M. Zlámalová, B. Pitňa Lásková, M. Vinarčíková, M. Zukalová and L. Kavan, “Inherent Electrochemical Activity of TiO_2_ (Anatase, Rutile) Enhances the Charge Capacity of Cathodes of Lithium-Sulfur Batteries,” J*ournal of Solid State Electrochemistry* 26 (2022): 639-647, https://doi.org/10.1007/s10008-022-05115-z.

20. X. Song, G. Chen, S. Wang, Y. Huang, Z. Jiang, L.X. Ding and H. Wang, “Self-Assembled Close-Packed MnO_(2)_ Nanoparticles Anchored on a Polyethylene Separator for Lithium-Sulfur Batteries,” *ACS Appl Mater Interfaces* 10 (2018): 26274-26282, https://doi.org/10.1021/acsami.8b07663.

21. X. Wang, L. Yang, Y. Wang, Q. Li, C. Chen, B. Zhong, Y. Chen, X. Guo, Z. Wu, Y. Liu, Y. Liu and Y. Sun, “Novel Functional Separator with Self-Assembled MnO_2_ Layer Via a Simple and Fast Method in Lithium-Sulfur Battery,” *Journal of Colloid and Interface Science* 606 (2022): 666-676, https://doi.org/10.1016/j.jcis.2021.08.062.

22. D.W. Kim, C. Senthil, S.M. Jung, S.-S. Kim, H.-S. Kim, J.W. Hong, J.-H. Ahn and H.Y. Jung “Selective Ion Transport of Catalytic Hybrid Aerofilm Interlayer for Long-Stable Li-S Batteries,” *Energy Storage Materials* 47 (2022): 472-481, https://doi.org/10.1016/j.ensm.2022.02.036.

23. Y. Yu, X. Zhang, L. He, F. Duan, J. Ba, C. Wang, Y. Wei and Y. Wang, “Manganese Oxide Nanosheets with Mixed Valence States as a Separator Coating for Lithium-Sulfur Batteries,” *ACS Applied Energy Materials* 6 (2023): 7298-7304, https://doi.org/10.1021/acsaem.3c01137.

24. Z. Ma, Z. Qi, G. Song, S. Huang, Z. Du, J. Dong, C. Guan, P. Luo, P. Gan, B. Yu, B. Guo, J. Chen, M. Wang, J. Zhang, X. Li, J. Zhang and F. Li, “Α-MnO_2_/RuO_2_ Heterostructure-Modified Polypropylene Separator for High-Performance Lithium-Sulfur Battery,” *Advanced Functional Materials* 34 (2024): 2403101, https://doi.org/10.1002/adfm.202403101.

25. Y. Si, J. Zhang, Q. Huang, J. Wei, C. Wang, K. Ji and M. Chen, “Manganese-Nickel Bimetallic Oxide Electrocatalyzing Redox Reactions of Lithium Polysulfides in Lithium-Sulfur Batteries,” *Sustainable Energy & Fuels* 6 (2022): 1426-1435, https://doi.org/10.1039/D1SE01987B.

26. Y. Wang, X. Guo, C. Chen, Y. Wang, Q. Li, Z. Wu, B. Zhong and Y. Chen, “Alleviating the Shuttle Effect Via Bifunctional MnFe_2_O_4_/A_b_ Modified Separator for High Performance Lithium Sulfur Battery,” *Electrochimica Acta* 354 (2020): 136704, https://doi.org/10.1016/j.electacta.2020.136704.

27. L. Zhou, H. Li, Y. Zhang, M. Jiang, D.L. Danilov, R.-A. Eichel and P.H.L. Notten, “Enhanced Sulfur Utilization in Lithium-Sulfur Batteries by Hybrid Modified Separators,” *Materials Today Communications* 26 (2021): 102133, https://doi.org/10.1016/j.mtcomm.2021.102133.

28. M. Yao, R. Wang, Z. Zhao, Y. Liu, Z. Niu and J. Chen, “A Flexible All-in-One Lithium-Sulfur Battery,” *Acs Nano* 12 (2018): 12503-12511, https://doi.org/10.1021/acsnano.8b06936.

29. L. Bai, Y. Ren, Q. Zheng, J. Nie and B. Du, “Modification of Polypropylene Separator with Nickel and Manganese Oxide-Decorated Carbon Nanotubes toward High-Performance Lithium-Sulfur Batteries,” *Journal of Power Sources* 633 (2025): 236353, https://doi.org/10.1016/j.jpowsour.2025.236353.

30. Z. Hao, J. Chen, X. Lu, L. Kang, C. Tan, R. Xu, L. Yuan, D.J.L. Brett, P.R. Shearing, F.R. Wang and Y. Huang, “Precisely Visit the Performance Modulation of Functionalized Separator in Li-S Batteries Via Consecutive Multiscale Analysis,” *Energy Storage Materials* 49 (2022): 85-92, https://doi.org/10.1016/j.ensm.2022.04.003.

31. H. Raza, J. Cheng, S. Kandasamy, M. Madithedu, N. Tewari, I.T. Bello, J. Wei, J. Xu, L. An, G. Zheng and S.T. Boles, “Manganese-Incorporated Single-Phase High-Entropy Oxide Modified Separator Enabled High Performance of Lithium-Sulfur Batteries at High Sulfur Loading,” *ENERGY & ENVIRONMENTAL MATERIALS*8 (2025): e70058, https://doi.org/10.1002/eem2.70058.

32. X. Wang, P. Lin, C. Wu, Y. Zhu, C. Wang, D. Guo, X.a. Chen and S. Wang, “Superior-Performance Lithium-Sulfur Batteries: A Face-Centered-Cubic-Structure High-Entropy Alloy Improves the Bidirectional Catalytic Conversion of Polysulfides/Sulfides,” *Inorganic Chemistry Frontiers* 11 (2024): 6425-6437, https://doi.org/10.1039/D4QI01434K.

33. T. Zhang, D. Luo, H. Xiao, X. Liang, F. Zhang, H. Zhuang, M. Li, L. Zheng and Q. Gao, “A Transmetalation Synthetic Strategy to Engineer Atomically Dispersed MnN_2_O_2_ Electrocatalytic Centers Driving High-Performance Li-S Battery,” *Small* 19 (2023): 2302249, https://doi.org/10.1002/smll.202302249.

34. N. Wang, B. Chen, K. Qin, E. Liu, C. Shi, C. He and N. Zhao, “Rational Design of Co_9_S_8_/CoO Heterostructures with Well-Defined Interfaces for Lithium Sulfur Batteries: A Study of Synergistic Adsorption-Electrocatalysis Function,” *Nano Energy* 60 (2019): 332-339, https://doi.org/10.1016/j.nanoen.2019.03.060.

35. R. Liu, W. Yin, Y. Chen, B. Zhong, G. Wang, Y. Liu, Y. Song, Z. Wu, L. Yang and X. Guo, “Facile Utilization of Spent LiCoO_2_ in Separator Decoration of Lithium-Sulfur Batteries,” *Industrial & Engineering Chemistry Research* 59 (2020): 17911-17917, https://doi.org/10.1021/acs.iecr.0c03490.

36. L. Zhang, F. Wan, H. Cao, L. Liu, Y. Wang and Z. Niu, “Integration of Binary Active Sites: Co_3_V_2_O_8_ as Polysulfide Traps and Catalysts for Lithium-Sulfur Battery with Superior Cycling Stability,” *Small* 16 (2020): 1907153, https://doi.org/10.1002/smll.201907153.

37. H. Li, Y. Wang, H. Chen, B. Niu, W. Zhang and D. Wu, “Synergistic Mediation of Polysulfide Immobilization and Conversion by a Catalytic and Dual-Adsorptive System for High Performance Lithium-Sulfur Batteries,” *Chemical Engineering Journal* 406 (2021): 126802, https://doi.org/10.1016/j.cej.2020.126802.

38. L. Wu, J. Hu, X. Yang, Z. Liang, S. Chen, L. Liu, H. Hou and J. Yang, “Synergistic Effect of Adsorption and Electrocatalysis of CoO/NiO Heterostructure Nanosheet Assembled Nanocages for High-Performance Lithium-Sulfur Batteries,” *Journal of Materials Chemistry A* 10 (2022): 23811-23822, <https://doi.org/10.1039/D2TA06876A>.

39. M. Zhang, H. Xiao, Y. Liu, W. Hou, X. Li, J. Deng, L. Hou and C. Yuan, “Interfacial Build-in Electric Field Unlocking Hetero-Architectured CoO-Co_3_O_4_ Nanotubes toward High-Performance Li-S Batteries,” *Advanced Functional Materials* 18 (2026): e20708, https://doi.org/10.1002/adfm.202520708.

40. W. Ma, Z. Shao, J. Yao, K. Zhao, X. Ma, L. Wu and X. Zhang, “Mott-Schottky Electrocatalyst Selectively Mediates the Sulfur Species Conversion in Lithium-Sulfur Batteries,” *Journal of Colloid and Interface Science* 631 (2023): 114-124, https://doi.org/10.1016/j.jcis.2022.11.041.

41. Z. Bai, Z. Wang, T. Wang, Z. Wu, X. Gao, Y. Bai, G. Wang and K. Sun, “Cation-Vacancy Engineering Modulated Perovskite Oxide for Boosting Electrocatalytic Conversion of Polysulfides,” *Advanced Functional Materials* 35 (2025): 2419105, https://doi.org/10.1002/adfm.202419105.

42. W. Zhou, M. Chen, D. Zhao, C. Zhu, N. Wang, W. Lei, Y. Guo and L. Li, “Engineering Spin State in Spinel Co_3_O_4_ Nanosheets by V-Doping for Bidirectional Catalysis of Polysulfides in Lithium-Sulfur Batteries,” *Advanced Functional Materials* 34 (2024): 2402114, https://doi.org/10.1002/adfm.202402114.

43. A. Gao, Q. Wu, H. Chen, Y. Pan, B. Niu, Y. Zhang and D. Long, “Oxygen Vacancies-Enriched CeO_2_-Co_x_O_y_ Heterostructures Facilitate the “Trapping-Conversion” Process of Lithium Polysulfides for High-Performance Li-S Batteries,” *Chemical Engineering Journal* 512 (2025): 162145, https://doi.org/10.1016/j.cej.2025.162145.

44. S. Wang, F. Gao, R. Ma, A. Du, T. Tan, M. Du, X. Zhao, Y. Fan and M. Wen, “ZnO Nanoparticles Anchored on a N-Doped Graphene-Coated Separator for High Performance Lithium/Sulfur Batteries,” *Metals* 8 (2018): 755,. https://doi.org/10.3390/met8100755.

45. J. Zou, Y. Cao, B. Zhang and W. Wang, “Suppressing the Shuttle Effect in Lithium-Sulphur Batteries by Defective Single-Walled Zno Nanotube: A Dft Study,” The Canadian *Journal of Chemical Engineering* 101 (2023): 347-353, <https://doi.org/10.1002/cjce.24357>.

46. S. Pi, X. Xu, W. Lei, R. Jiang, Z. Ma, Y. Zou, X. Wang and Y. Wang, “Multi-Active Sites Synergistic Surface-Functionalized Carbon Nanotubes Modified Separators Facilitate the Application of High-Performance Lithium-Sulfur Batteries,” *Journal of Power Sources* 659 (2025): 238359, https://doi.org/10.1016/j.jpowsour.2025.238359.

47. X. Qian, J. Cheng, L. Jin, Y. Wang, B. Huang and J. Chen, “ZIF-8/Ketjen Black Derived ZnO/N/Kb Composite for Separator Modification of Lithium Sulfur Batteries,” *Colloids and Surfaces A: Physicochemical and Engineering Aspects* 648 (2022): 129036, https://doi.org/10.1016/j.colsurfa.2022.129036.

48. W. Zhang, D. Hong, Z. Su, S. Yi, L. Tian, B. Niu, Y. Zhang and D. Long, “Tailored Zno-Zns Heterostructure Enables a Rational Balancing of Strong Adsorption and High Catalytic Activity of Polysulfides for Li-S Batteries,” *Energy Storage Materials* 53 (2022): 404-414, https://doi.org/10.1016/j.ensm.2022.09.018.

49. Q. Hao, X. Qian, L. Jin, J. Cheng, S. Zhao, J. Chen, K. Zhang, B. Li, S. Pang and X. Shen, “Application of ZIF-67/ZIF-8 Derived Co_3_O_4_/ZnO Heterojunction in Lithium-Sulfur Battery Separators,” *Journal of Alloys and Compounds* 967 (2023): 171605, https://doi.org/10.1016/j.jallcom.2023.171605.

50. C. Zhang, L. Pan, H. Liu, X. Qi, F. You, X. Cao, Z. Yang, Z. Zhang and K. Li, “Construction of Porous Hetero Structured Materials Defluorinated Carbon Nanotubes@SiO_2_-ZnO Modified Separator for Safety and High Performance Lithium‑Sulfur Batteries,” *Journal of Energy Storage* 95 (2024): 112528, https://doi.org/10.1016/j.est.2024.112528.

51. X. Men, T. Deng, J. Che and J. Wang, “Accelerating Sulfur Conversion Kinetics Via CoS_2_-MgS Heterostructure for Lithium Sulfur Batteries,” *Journal of Materials Chemistry A* 12 (2024): 31972-31981, https://doi.org/10.1039/D4TA06086E.

52. F. Li, X. Qian and L. Jin, “MOF-Derived Mns/N-C@Cnt Composites as Separator Coating Materials for Long-Cycling Li-S Batteries,” *Acs Sustainable Chemistry & Engineering* 9 (2021): 15469-15477, https://doi.org/10.1021/acssuschemeng.1c04636.

53. F. Xie, M. Xiong, J. Liu, J. Qian, T. Mei, J. Li, J. Wang, L. Yu, J.P. Hofmann and X. Wang, “A Multi-Functional Separator for Li-S Batteries: WS2@C Nanoflowers Catalyze the Rapid Recycling of Lithium Polysulfides by Polar Attraction,” *ChemElectroChem* 9 (2022): e202200474, https://doi.org/10.1002/celc.202200474.

54. M.A. Al-Tahan, Y. Dong, A.E. Shrshr, X. Liu, R. Zhang, H. Guan, X. Kang, R. Wei and J. Zhang, “Enormous-Sulfur-Content Cathode and Excellent Electrochemical Performance of Li-S Battery Accouched by Surface Engineering of Ni-Doped WS2@RGO Nanohybrid as a Modified Separator,” *Journal of Colloid and Interface Science* 609 (2022): 235-248, https://doi.org/10.1016/j.jcis.2021.12.035.

55. Y. Lin, Z. Ouyang, S. He, X. Song, Y. Luo, J. Zhao, Y. Xiao, S. Lei, C. Yuan and B. Cheng, “An Individual Sandwich Hybrid Nanostructure of Cobalt Disulfide in-Situ Grown on N Doped Carbon Layer Wrapped on Multi-Walled Carbon Nanotubes for High-Efficiency Lithium Sulfur Batteries,” *Journal of Colloid and Interface Science* 610 (2022): 560-572, https://doi.org/10.1016/j.jcis.2021.11.102.

56. W. Zhang, K. Zhao, Q. Jin, J. Xiao, H. Lu, X. Zhang and L. Wu, “CoS_2_-NC@Cnts Hierarchical Nanostructures for Efficient Polysulfide Regulation in Lithium-Sulfur Batteries,” *Electrochimica Acta* 430 (2022): 141104, https://doi.org/10.1016/j.electacta.2022.141104.

57. R. Bi, J. Zhao, M. Yang, J. Wang, R. Yu and D. Wang, “Multifunctional Separator Modified with Catalytic Multishelled Structural CoS_2_ Enables a Stable Lithium-Sulfur Battery,” *Inorganic Chemistry Frontiers* 11 (2024): 8837-8846, https://doi.org/10.1039/D4QI02340D.

58. P. Wan, H. Xu, R. Cao, X. Liu, Y. Xia, A. Xu, M. Yuan, H. Jin and H. Xu, “A Hierarchically Porous CoS_2_-NiS_2_ Heterojunction for High-Performance Lithium Sulfur Batteries,” *Journal of Colloid and Interface Science* 687 (2025): 599-606, https://doi.org/10.1016/j.jcis.2025.02.084.

59. Y. Ren, Y. Ma, B. Wang, S. Chang, Q. Zhai, H. Wu, Y. Dai, Y. Yang, S. Tang and X. Meng, “Furnishing Continuous Efficient Bidirectional Polysulfide Conversion for Long-Life and High-Loading Lithium-Sulfur Batteries Via the Built-in Electric Field,” *Small* 19 (2023): 2300065, https://doi.org/10.1002/smll.202300065.

60. Y. Tian, Z. Wei, F. Li, S. Li, L. Shao, M. He, P. Sun and Y. Li, “Enhanced Multiple Anchoring and Catalytic Conversion of Polysulfides by SnO_2_-Decorated MoS_2_ Hollow Microspheres for High-Performance Lithium-Sulfur Batteries,” *Journal of Materials Science & Technology* 100 (2022): 216-223, https://doi.org/10.1016/j.jmst.2021.06.002.

61. G. Zhang, X. Chen, X. Yu, Q. Li, H. Wang, S. Hu, J. Jiang, Y. Huang and Z. Ma, “Interfacial Engineering Design Induces Enriched-Defects Expediting Catalytic Conversion Kinetics of Polysulfides,” *Journal of Materials Chemistry A* 12 (2024): 8927-8939, https://doi.org/10.1039/D3TA07886H.

62. Y. Li, D. Lei, T. Jiang, J. Guo, X. Deng, X. Zhang, C. Hao and F. Zhang, “P-Doped Co_9_S_8_ Nanoparticles Embedded on 3D Spongy Carbon-Sheets as Electrochemical Catalyst for Lithium-Sulfur Batteries,” *Chemical Engineering Journal* 426 (2021): 131798, https://doi.org/10.1016/j.cej.2021.131798.

63. J. Liu, Z. Qiao, Q. Xie, D.-L. Peng and R.-J. Xie, “Phosphorus-Doped Metal-Organic Framework-Derived CoS_2_ Nanoboxes with Improved Adsorption-Catalysis Effect for Li-S Batteries,” *ACS Applied Materials & Interfaces* 13 (2021): 15226-15236, https://doi.org/10.1021/acsami.1c00494.

64. J. Ren and Q. Zhao, “Preparation of a Lithium-Sulfur Battery Diaphragm Catalyst and Its Battery Performance,” *RSC Advances* 14 (2024): 36471-36487, https://doi.org/10.1039/d4ra06366j.

65. H. Zhu, Y. Xiao, J. Zhao, R. Luo, Z. Liu, Y. Jiang, Y. Kong, M. Ouyang, Q. Wei and N. Cheng, “Copper-Doping-Induced Lattice Distortion in CoS_2_ Nanocages for Enhanced Polysulfide Conversion in Advanced Li-S Batteries,” *Acs Sustainable Chemistry & Engineering* 13 (2025): 18703-18712, https://doi.org/10.1021/acssuschemeng.5c07370.

66. S. Deng, Y. Lv, Y. Zhao, H. Lu, Z. Han, L. Wu and X. Zhang, “Exquisitely Constructing Hierarchical Carbon Nanoarchitectures Decorated with Sulfides for High-Performance Li-S Batteries,” *Dalton Transactions* 53 (2024): 4753-4763, https://doi.org/10.1039/D3DT04163H.

67. X. Xu, H. Xu, R. Wang, Y. Liang, G. Chen, P. Ke, W. Lei and Z. Ma, “Enhanced Adsorption and Catalytic Conversion of Lithium Polysulfides Enabled by CoS_2_-WS_2_/Gnc Heterostructure-Modified Separator,” *Applied Surface Science* 717 (2026): 164736, https://doi.org/10.1016/j.apsusc.2025.164736.

68. B. Zhang, J. Ma, M. Cui, Y. Zhao and S. Wei, “A Rational Design of a CoS_2_-CoSe_2_ Heterostructure for the Catalytic Conversion of Polysulfides in Lithium-Sulfur Batteries,” *Materials* 16 (2023): 3992, https://doi.org/10.3390/ma16113992.

69. S. Tian, Q. Zeng, G. Liu, J. Huang, X. Sun, D. Wang, H. Yang, Z. Liu, X. Mo, Z. Wang, K. Tao and S. Peng, “Multi-Dimensional Composite Frame as Bifunctional Catalytic Medium for Ultra-Fast Charging Lithium-Sulfur Battery,” *Nano-Micro Letters* 14 (2022): 196, https://doi.org/10.1007/s40820-022-00941-2.

70. J. Wang, Y. Cao, Z. Wang, Y. Zhao, C. He, F. Zhao, C. Han and S. Yu, “Superior and Safer Lithium Sulfur Batteries Realized by Robust Polysulfides-Retarding Dam with High Flame Retardance,” *Journal of Energy Chemistry* 89 (2024): 471-486, https://doi.org/10.1016/j.jechem.2023.09.047.

71. X. Wang, X. Zhao, C. Ma, Z. Yang, G. Chen, L. Wang, H. Yue, D. Zhang and Z. Sun, “Electrospun Carbon Nanofibers with Mns Sulfiphilic Sites as Efficient Polysulfide Barriers for High-Performance Wide-Temperature-Range Li-S Batteries,” *Journal of Materials Chemistry* A 8 (2020): 1212-1220, https://doi.org/10.1039/C9TA12137D.

72. F. Ma, P. Hu, T. Wang, J. Liang, R. Han, J. Han and Q. Li, “Yolk@Shell Structured MnS@Nitrogen-Doped Carbon as a Sulfur Host and Polysulfide Conversion Booster for Lithium/Sodium Sulfur Batteries,” *ACS Applied Energy Materials* 4 (2021): 3487-3494, https://doi.org/10.1021/acsaem.0c03235.

73. Y. Zhu, Y. Zuo, X. Jiao, R. Manjunatha, E.R. Ezeigwe, W. Yan and J. Zhang, “Selective Sulfur Conversion with Surface Engineering of Electrocatalysts in a Lithium-Sulfur Battery,” *Carbon Energy* 5 (2023): e249, https://doi.org/10.1002/cey2.249.

74. Y. Li, Y. Hao, U. Ali, Q. Zhang, Z. Jin, H. Sun, L. Li, L. Zhang, C. Wang and B. Liu, “Bimetallic Sulfides Heterostructure Efficient Adsorption and Catalytic of Polysulfides for High-Performance Lithium-Sulfur Batteries,” *Chemical Engineering Journal* 474 (2023): 145961, https://doi.org/10.1016/j.cej.2023.145961.

75. Z. Chen, J. Wu, M. Jiang, Y. Yang, W. Cao, J. Zhang, X. Gao, E. Hu and Z. Chen, “Reinforcing the Adsorption and Conversion of Polysulfides in Li-S Battery by Incorporating Molybdenum into MnS/MnO Nanorods,” *Chemistry* 30 (2024): e202303507, https://doi.org/10.1002/chem.202303507.

76. L. Liu, M. Yan, X. Zhao and H. Pan, “A Novel Pathway for Sustained Sulfides Conversion Via Electrocatalyst-Modified Separator in Lithium-Sulfur Batteries,” *Nano Energy* 130 (2024): 110122, https://doi.org/10.1016/j.nanoen.2024.110122.

77. Y. Li, C. Xu, D. Li, Y. Zhang, B. Liu and P. Huo, “ZnS/MoS_2_ Heterostructures on Mxene: A Strategy for Improved Polysulfide Adsorption and Conversion in Li-S Batteries,” *Chemical Engineering Journal* 502 (2024): 158151, https://doi.org/10.1016/j.cej.2024.158151.

78. Z.A. Ghazi, X. He, A.M. Khattak, N.A. Khan, B. Liang, A. Iqbal, J. Wang, H. Sin, L. Li and Z. Tang, “MoS_2_/Celgard Separator as Efficient Polysulfide Barrier for Long-Life Lithium-Sulfur Batteries,” *Advanced Materials* 29 (2017): 1606817, https://doi.org/10.1002/adma.201606817.

79. S. Yang, Z. Junfan, T. Tan, Y. Zhao, N. Liu and H. Li, “A 3D MoS_2_/Graphene Microsphere Coated Separator for Excellent Performance Li-S Batteries,” *Materials* 11 (2018): 2064, https://doi.org/10.3390/ma11102064.

80. P. Han, S.-H. Chung and A. Manthiram, “Thin-Layered Molybdenum Disulfide Nanoparticles as an Effective Polysulfide Mediator in Lithium-Sulfur Batteries,” *ACS Applied Materials & Interfaces* 10 (2018): 23122-23130, https://doi.org/ 10.1021/acsami.8b05397.

81. M. Shi, Z. Liu, S. Zhang, S. Liang, Y. Jiang, H. Bai, Z. Jiang, J. Chang, J. Feng, W. Chen, H. Yu, S. Liu, T. Wei and Z. Fan, “A Mott-Schottky Heterogeneous Layer for Li-S Batteries: Enabling Both High Stability and Commercial-Sulfur Utilization,” *Advanced Energy Materials* 12 (2022): 2103657, https://doi.org/10.1002/aenm.202103657.

82. D. Lei, W. Shang, X. Zhang, Y. Li, S. Qiao, Y. Zhong, X. Deng, X. Shi, Q. Zhang, C. Hao, X. Song and F. Zhang, “Facile Synthesis of Heterostructured MoS_2_-MoO_3_ Nanosheets with Active Electrocatalytic Sites for High-Performance Lithium-Sulfur Batteries,” *Acs Nano* 15 (2021): 20478-20488, https://doi.org/10.1021/acsnano.1c09007.

83. Y. Li, T. Li, Y. Deng, W. Tang, H. Wu, M. Feng, P. Yan and R. Liu, “Tuning the D-Band Center of Bi_2_S_3_-MoS_2_ Heterostructure Towards Superior Lithium-Sulfur Batteries,” *Small* 20 (2024): 2401921, https://doi.org/10.1002/smll.202401921.

84. H. Li, D. Li, Y. Li, H. Liu, J. Li and W. Han, “Coupling 5 D Metallic and Conductive Network for Promoting Polysulfide Conversion,” *Chemical Engineering Journal* 502 (2024): 158205, https://doi.org/10.1016/j.cej.2024.158205.

85. L. Pan, C. Zhang, H. Liu, X. Qi, Z. Zhang, Z. Yang and K. Li, “MoS_2_@SiO_2_ Heterostructures Modified Separator Utilized for Efficient Adsorption and Rapid Conversion of Polysulfides,” *Journal of Alloys and Compounds* 993 (2024): 174546, https://doi.org/10.1016/j.jallcom.2024.174546.

86. Q. Jiang, H. Xu, K.S. Hui, Y. Wei, L. Liu, Z. Ye, C. Zha, M. Zheng, J. Lu and K.N. Hui, “Inner-Layer Indium Doping Achieved Highly Active and Stable Sulfur Vacancies in MoS_2_ for Superior Sulfur Redox Kinetics,” *Advanced Materials* 37 (2025): 2415986, https://doi.org/10.1002/adma.202415986.

87. C. Yang, N. Gong, T. Chen, Y. Li, W. Peng, F. Zhang and X. Fan, “Enhanced Catalytic Conversion of Polysulfides Using High-Percentage 1t-Phase Metallic WS_2_ Nanosheets for Li-S Batteries,” *Green Energy & Environment* 7 (2022): 1340-1348, https://doi.org/10.1016/j.gee.2021.03.001.

88. S. Ali, M. Waqas, X. Jing, N. Chen, D. Chen, J. Xiong and W. He, “Carbon-Tungsten Disulfide Composite Bilayer Separator for High-Performance Lithium-Sulfur Batteries,” *ACS Applied Materials & Interfaces* 10 (2018): 39417-39421, https://doi.org/10.1021/acsami.8b12682.

89. J. Liu, K. Li, Q. Zhang, X. Zhang, X. Liang, J. Yan, H.H. Tan, Y. Yu and Y. Wu, “3D Tungsten Disulfide/Carbon Nanotube Networks as Separator Coatings and Cathode Additives for Stable and Fast Lithium-Sulfur Batteries,” *ACS Appl Mater Interfaces* 13 (2021): 45547-45557, https://doi.org/10.1021/acsami.1c13193.

90. Q. Wang, A. Liu, S. Qiao, Q. Zhang, C. Huang, D. Lei, X. Shi, G. He and F. Zhang, “Mott-Schottky Mxene@WS2 Heterostructure: Structural and Thermodynamic Insights and Application in Ultra Stable Lithium-Sulfur Batteries,” *ChemSusChem* 16 (2023): e202300507, https://doi.org/10.1002/cssc.202300507.

91. A.E. Shrshr, B. Miao, M.M. Elsenety, A.Z. Alhakemy, M.S. Nadeem, I.A. Mir, S. Ahmed, L. Rasheed, M. Yasir, J. Bai, J. Zhang and M.A. Al-Tahan, “An Innovative One-Step Hydrothermal Approach for Fabricating Ta_2_O_5_/WS_2_@RGO Heterostructure as a Coated Separator for Li-S Batteries,” *Materials Today Chemistry* 50 (2025): 103136, https://doi.org/10.1016/j.mtchem.2025.103136.

92. J. Huang, D. Wang, Z. Cheng, X. Xie, B. Chang, X. Li and R. Wang, “Morphology and Electron Regulation of B-WS_2_@Mxene Nanosheet Array Heterojunctions to Upgrade the Capacity and Longevity of Lithium-Sulfur Batteries,” *Journal of Materials Chemistry A* 13 (2025): 18880-18891, https://doi.org/10.1039/D5TA02390D.

93. S. Lu, L. Cai, J. Wang, H. Ying, Z. Han, W. Han and Z. Chen, “2D Ultrathin Titanium Nitride Nanosheets as Separator Coatings for Li-S Batteries,” *Small* 20 (2024): 2307784, https://doi.org/10.1002/smll.202307784.

94. F. Ma, Z. Chen, K. Srinivas, D. Liu, Z. Zhang, Y. Wu, M.-q. Zhu, Q. Wu and Y. Chen, “Vn Quantum Dots Anchored N-Doped Carbon Nanosheets as Bifunctional Interlayer for High-Performance Lithium-Metal and Lithium-Sulfur Batteries,” *Chemical Engineering Journal* 459 (2023): 141526, https://doi.org/10.1016/j.cej.2023.141526.

95. Z. Hao, L. Yuan, C. Chen, J. Xiang, Y. Li, Z. Huang, P. Hu and Y. Huang, “TiN as a Simple and Efficient Polysulfide Immobilizer for Lithium-Sulfur Batteries,” *Journal of Materials Chemistry A* 4 (2016): 17711-17717, https://doi.org/10.1039/C6TA07411A.

96. B. Qi, X. Zhao, S. Wang, K. Chen, Y. Wei, G. Chen, Y. Gao, D. Zhang, Z. Sun and F. Li, “Mesoporous TiN Microspheres as an Efficient Polysulfide Barrier for Lithium-Sulfur Batteries,” *Journal of Materials Chemistry A* 6 (2018): 14359-14366, https://doi.org/10.1039/C8TA04920C.

97. G. Xu, Q.-b. Yan, P. Bai, H. Dou, P. Nie and X. Zhang, “Nano-Sized Titanium Nitride Functionalized Separator Improves Cycling Performance of Lithium Sulfur Batteries,” *ChemistrySelect* 4 (2019): 698-704, https://doi.org/10.1002/slct.201803285.

98. Q. Shen, L. Huang, G. Chen, X. Zhang and Y. Chen, “One-Step Synthesis of Titanium Nitride/Nitrogen-Doped Graphene Nanocomposite as Separator Modifying Material for Advanced Lithium-Sulfur Batteries,” *Journal of Alloys and Compounds* 845 (2020): 155543, https://doi.org/10.1016/j.jallcom.2020.155543.

99. J. Zhu, C. Jiao, T. Kang, L. Zhong, S. Zhao and Y. Qiu, “A Sandwich-Structured TiN/BN-C Composite Interlayer with Enhanced Performance for Li-S Batteries,” *Journal of Electroanalytical Chemistry* 862 (2020): 113963, https://doi.org/10.1016/j.jelechem.2020.113963.

100. N. Li, L. Yu and J. Xi, “Integrated Design of Interlayer/Current-Collector: Heteronanowires Decorated Carbon Microtube Fabric for High-Loading and Lean-Electrolyte Lithium-Sulfur Batteries,” *Small* 17 (2021): 2103001, https://doi.org/10.1002/smll.202103001.

101. J. Zhu, C. Ma, X. Jia, Y. Zhang, X. Liu, J. Wang, W. Qiao and L. Ling, “TiO_2_-TiN Decorated Carbon Nanoplates Derived from NH_2_-MIL-125 with Efficient Polysulfide Catalytic Conversion for Long-Life Lithium-Sulfur Batteries,” *Industrial & Engineering Chemistry Research* 62 (2023): 14380-14390, https://doi.org/10.1021/acs.iecr.3c01206.

102. Y. Kim, Y. Noh, J. Bae, H. Ahn, M. Kim and W.B. Kim, “N-Doped Carbon-Embedded TiN Nanowires as a Multifunctional Separator for Li-S Batteries with Enhanced Rate Capability and Cycle Stability,” *Journal of Energy Chemistry* 57 (2021): 10-18, https://doi.org/10.1016/j.jechem.2020.08.050.

103. Y. Fan, K. Liu, A. Ali, X. Chen and P.K. Shen, “2D TiN@C Sheets Derived from Mxene as Highly Efficient Polysulfides Traps and Catalysts for Lithium-Sulfur Batteries,” *Electrochimica Acta* 384 (2021): 138187, https://doi.org/10.1016/j.electacta.2021.138187.

104. K. Liu, X. Zhang, F. Miao, Z. Wang, S. Zhang, Y. Zhang, P. Zhang and G. Shao, “In Situ Electrochemical Intercalation-Induced Phase Transition to Enhance Catalytic Performance for Lithium-Sulfur Battery,” *Small* 17 (2021): 2100065, https://doi.org/10.1002/smll.202100065.

105. G. Li, S. Zhang, S. Zhao, X. Jiang, Y. Cui, J. Lian and Y. Wang, “Design of Spherical TiN/Nb_4_N_5_ Heterostructure as Bifunctional Adsorption/Catalytic Material for Lithium Sulfur Batteries,” *Materials Today Communications* 33 (2022): 104403, https://doi.org/10.1016/j.mtcomm.2022.104403.

106. Y. Zhu, X. Wu, M. Li, Y. Ji, Q. Li, X. He, Z. Lei, Z. Liu, R. Jiang and J. Sun, “Synthesis of Titanium Molybdenum Nitride-Decorated Electrospun Carbon Nanofiber Membranes as Interlayers to Suppress Polysulfide Shuttling in Lithium-Sulfur Batteries,” *Acs Sustainable Chemistry & Engineering* 10 (2022): 776-788, https://doi.org/10.1021/acssuschemeng.1c05959.

107. G.H. Park, W.-G. Lim, Y.H. Jeong, S.K. Kang, M. Kim, J. Ji, J. Ha, S.R. Mangishetti, S. Kim, Y. Park, C. Jo and W.B. Kim, “Activation of the Radical-Mediated Pathway and Facilitation of the Li_2_S Conversion by N-Doped Carbon-Embedded Ti_1-x_Co_x_N Nanowires as a Multifunctional Separator with a High Donor-Number Solvent toward Advanced Lithium-Sulfur Batteries,” *Small Structures* 5 (2024): 2400293, https://doi.org/10.1002/sstr.202400293.

108. Y. Xiang, L. Lu, F. Yan, D. Sengupta, P. Rudolf, A.G.P. Kottapalli and Y. Pei, “Tuning the Crystallinity of Titanium Nitride on Copper-Embedded Carbon Nanofiber Interlayers for Accelerated Electrochemical Kinetics in Lithium-Sulfur Batteries,” *Carbon Energy* 6 (2024): e450, https://doi.org/10.1002/cey2.450.

109. K. Yang, X. Xu, C. Li, F. Zhao, J. Li and Y. He, “Realizing Dual Regulation of Polysulfides and Lithium Ions by a Versatile Separator,” *Science China Materials* 67 (2024): 116-124, https://doi.org/10.1007/s40843-023-2695-5.

110. P. Wan, X. Guo, S. Jing, S. Li, S. Lu, Y. Zhang and H. Fan, “Multicomponent Synergy and Simultaneous Improvement of Catalytic Conversion and Chemisorption of Polysulfides Via CoFe/FeN_0.0324_/TiN Modified Li-S Battery Separator,” *Chemical Engineering Journal* 504 (2025): 159014, https://doi.org/10.1016/j.cej.2024.159014.

111. L. Yin, C. Zhang, W. Chen, J. Xu, C. Shi, X. Qi, Y. Liu, J. Liu, S. Chen, X. Zhao and J. Song, “Accelerating Polysulfide Conversion by 2d-2d MoN/TiN Heterostructure Towards High-Performance Lithium-Sulfur Battery,” *Chemical Engineering Journal* 512 (2025): 162147, https://doi.org/10.1016/j.cej.2025.162147.

112. D. Tian, X. Song, M. Wang, X. Wu, Y. Qiu, B. Guan, X. Xu, L. Fan, N. Zhang and K. Sun, “MoN Supported on Graphene as a Bifunctional Interlayer for Advanced Li-S Batteries,” *Advanced Energy Materials* 9 (2019): 1901940, https://doi.org/10.1002/aenm.201901940.

113. T. Tan, N. Chen, Z. Wang, Z. Tang, H. Zhang, Q. Lai and Y. Liang, “Thorn-Like Carbon Nanofibers Combined with Molybdenum Nitride Nanosheets as a Modified Separator Coating: An Efficient Chemical Anchor and Catalyst for Li-S Batteries,” ACS *Applied Energy Materials* 5 (2022): 6654-6662, https://doi.org/10.1021/acsaem.2c00080.

114. X.-Y. Yue, J. Zhang, J. Bao, Y.-F. Bai, X.-L. Li, S.-Y. Yang, Z.-W. Fu, Z.-H. Wang and Y.-N. Zhou, “Sputtered MoN Nanolayer as a Multifunctional Polysulfide Catalyst for High-Performance Lithium-Sulfur Batteries,” *eScience* 2 (2022): 329-338, https://doi.org/10.1016/j.esci.2022.03.003.

115. Y. Kong, L. Wang, M. Mamoor, B. Wang, G. Qu, Z. Jing, Y. Pang, F. Wang, X. Yang, D. Wang and L. Xu, “Co/MoN Invigorated Bilateral Kinetics Modulation for Advanced Lithium-Sulfur Batteries,” *Advanced Materials* 36 (2024): 2310143, https://doi.org/10.1002/adma.202310143.

116. L. Ban, “Novel Functional Separator with MoO_2_ Nanosheets for Advanced Electrochemical Performance in Li-S Battery,” *Ionics* 28 (2022): 2769-2773, https://doi.org/10.1007/s11581-022-04537-z.

117. X. Zhang, J. Zhao, Y. Zhao, Z. Bakenov and Y. Zhang, “Engineering Zwitterionic Barrier by Squaraine-Based Porous Organic Framework Fiber for Superior Lithium-Sulfur Batteries,” *Electrochimica Acta* 397 (2021): 139276, https://doi.org/10.1016/j.electacta.2021.139276.

118. D. Wang, K. Ma, J. Hao, W. Zhang, H. Shi, C. Wang, Z. Xiong, Z. Bai, F.-R. Chen, J. Guo, B. Xu, X. Yan and Y. Gu, “Engineering Single-Atom Catalysts as Multifunctional Polysulfide and Lithium Regulators toward Kinetically Accelerated and Durable Lithium-Sulfur Batteries,” *Chemical Engineering Journal* 466 (2023): 143182, https://doi.org/10.1016/j.cej.2023.143182.

119. E. Jing, L. Chen, S. Xu, W. Tian, D. Zhang, N. Wang, Z. Bai, X. Zhou, S. Liu, D. Duan and X. Qiu, “Dual Redox Catalysis of Vn/Nitrogen-Doped Graphene Nanocomposites for High-Performance Lithium-Sulfur Batteries,” *Journal of Energy Chemistry* 64 (2022): 574-582, https://doi.org/10.1016/j.jechem.2021.05.015.

120. J. Shi, Y. Yao, L. Xue, K. Li, J. Ning, F. Jiang and F. Huang, “Application of Magnetron Sputtering to Deposit a Multicomponent Separator with Polysulfide Chemisorption and Electrode Stabilization for High-Performance Lithium‑Sulfur Batteries,” *Surface and Coatings Technology* 405 (2021): 126580, https://doi.org/10.1016/j.surfcoat.2020.126580.

121. Q. Sun, Q. Shu, C. Ma, W. Qiao, J. Wang, L. Ling, Z. Yu and Y. Zhang, “A Bifunctional N-Doped Carbon Nanosheet Embedded with VN/Mo_2_C Heterointerfaces Electrocatalyst for Expediting Sulfur Redox and Lithium Regulation toward Advanced Li-S Battery,” *Chemical Engineering Journal* 489 (2024): 151115, https://doi.org/10.1016/j.cej.2024.151115.

122. G. Ruan, Y. Shen, J. Yao, Y. Huang, S. Yang, S. Hu, H. Wang, Y. Fang and X. Cai, “Vanadium Nitride Nanocrystals Decorated Ultrathin, N-Rich and Hierarchically Porous Carbon Nanosheets as Superior Polysulfides Mediator for Stable Lithium-Sulfur Batteries,” *Journal of Power Sources* 566 (2023): 232922, https://doi.org/10.1016/j.jpowsour.2023.232922.

123. J. Hou, M. Yang, X. Guo, H. Li, Z. Liu, Y. Cui, D. Shi, H. Hu, B. Zhang, Y. Shao, Y. Wu and X. Hao, “Chemisorption-Catalysis Synergy Driven VN-V_2_O_3_ Anchored on Reduced Graphene Oxide Heterostructure for Stable Lithium-Sulfur Battery Separators,” *Journal of Power Sources* 645 (2025): 237197, https://doi.org/10.1016/j.jpowsour.2025.237197.

124. J. Pu, J. Wu, Y. Tan and S. Yu, “Cobalt Nitride Nanocrystals Coated Separator as Multifunctional Interlayer for Improving Polysulfides Regulation,” *Journal of Alloys and Compounds* 920 (2022): 165964, https://doi.org/10.1016/j.jallcom.2022.165964.

125. M. Luo, Y. Bai, R. Sun, M. Qu, M. Wang, Z. Yang, Z. Wang, W. Sun and K. Sun, “Enhancing Anchoring and Catalytic Conversion of Polysulfides by Nitrogen Deficient Cobalt Nitride for Advanced Lithium-Sulfur Batteries,” *Journal of Energy Chemistry* 73 (2022): 407-415, https://doi.org/10.1016/j.jechem.2022.05.029.

126. M. Zhao, H.-J. Peng, Z.-W. Zhang, B.-Q. Li, X. Chen, J. Xie, X. Chen, J.-Y. Wei, Q. Zhang and J.-Q. Huang, “Activating Inert Metallic Compounds for High-Rate Lithium-Sulfur Batteries through in Situ Etching of Extrinsic Metal,” *Angewandte Chemie International Edition* 58 (2019): 3779-3783, https://doi.org/10.1002/anie.201812062.

127. X. Li, W. Shang, S. Zhang, C. Xu, J. Lian and G. Li, “The Design of the Ni_(3)_N/Nb_(4)_N_(5)_ Heterostructure as Bifunctional Adsorption/Electrocatalytic Materials for Lithium-Sulfur Batteries,” *Nanomaterials (Basel)* 15 (2025): https://doi.org/10.3390/nano15131015.

128. F. Yi, H. Wang, J. Wang, W. Liu, M. Li, J. Yao, J. Cui, Y. Wang and M. Ren, “Bifunctional Mott-Schottky-Type Co-Fe_3_N Electrocatalyst as Sulfur Host and Separator Modifier to Enhance the Transformation Kinetics of Li_2_S,” *Journal of Power Sources* 624 (2024): 235562, https://doi.org/10.1016/j.jpowsour.2024.235562.

129. J. Li, X. Zhang, Z. Zhang, Y. Li and L. Zhu, “Preparation of Ldhs-Derived Co-Ni_3_N Heterostructure Modified Porous Carbon Nanofibers as Advanced Interlayers for Lithium-Sulfur Batteries,” *Journal of Alloys and Compounds* 971 (2024): 172731, https://doi.org/10.1016/j.jallcom.2023.172731.

130. H. Jia, J. Fan, P. Su, T. Guo and M.-C. Liu, “Cobalt Nitride Nanoparticles Encapsulated in N-Doped Carbon Nanotubes Modified Separator of Li-S Battery Achieving the Synergistic Effect of Restriction-Adsorption-Catalysis of Polysulfides,” *Small* 20 (2024): 2311343, https://doi.org/10.1002/smll.202311343.

131. Y. Tang, Y. Yuan, Y. Mo, H. Tang, M. Wei, H. Zhou, T. Sheng, C. Li, Y. Sun and X. Wei, “CoN//Co_9_S_8_ Co-Embedded in Reduced Graphene Oxide as Catalyst for Catalytic Conversion of Polysulfides,” *Materials Today Physics* 46 (2024): 101480, https://doi.org/10.1016/j.mtphys.2024.101480.

132. C. Ma, Y. Zhang, Y. Feng, N. Wang, L. Zhou, C. Liang, L. Chen, Y. Lai, X. Ji, C. Yan and W. Wei, “Engineering Fe-N Coordination Structures for Fast Redox Conversion in Lithium-Sulfur Batteries,” *Advanced Materials* 33 (2021): 2100171, https://doi.org/10.1002/adma.202100171.

133. X. Wang, D. Wang, C. Ma, Z. Yang, H. Yue, D. Zhang and Z. Sun, “Conductive Fe_2_N/N-RGO Composite Boosts Electrochemical Redox Reactions in Wide Temperature Accommodating Lithium-Sulfur Batteries,” *Chemical Engineering Journal* 427 (2022): 131622, https://doi.org/10.1016/j.cej.2021.131622.

134. J. Zhang, Y. Yao, X. Song, K. Shi and Y. Song, “Mxene Nanosheets Modified with Different Intercalators as the Separator for Lithium-Sulfur Batteries,” *Journal of Materials Chemistry C* 13 (2025): 10103-10110, https://doi.org/10.1039/D4TC05447D.

135. L. Liao, F. Wang, J. He, L. Ma, Q. Liu and Y. Deng, “Ultrathin Ta-Mxene Modification Layer with Sub-Nanometer Interlayer Channels Suppresses Polysulfide Shuttling and Accelerates Li^+^ Desolvation,” *Advanced Functional Materials* 10 (2026) e12612, https://doi.org/10.1002/adfm.202512612.

136. Z.H. Luo, M. Zheng, M.X. Zhou, X.T. Sheng, X.L. Chen, J.J. Shao, T.S. Wang and G. Zhou, “2D Nanochannel Interlayer Realizing High-Performance Lithium-Sulfur Batteries,” *Advanced Materials* 37 (2025): 2417321, https://doi.org/10.1002/adma.202417321.

137. B. Cetiner, A.A. Hamedani, B. Iskandarani, S. Deng, J. Heier, B. Yarar Kaplan, S. Alkan Gürsel and A. Yürüm, “High‐Performance Lithium-Sulfur Batteries with Mxene‐Transition Metal Oxide Decorated Electrospun Interlayers for Optimized Polysulfide Conversion,” *International Journal of Energy Research* 17 (2025): 8862016, https://doi.org/10.1155/er/8862016.

138. J. Zhou, Z. Pei, Z. Sui, Y. Liang, X. Xu, Y. Li, Y. Li, J. Qiu and Q. Chen, “Hierarchical Porous and Three-Dimensional MXene/SiO_2_ Hybrid Aerogel through a Sol-Gel Approach for Lithium-Sulfur Batteries,” *Molecules* 27 (2022): 7073, https://doi.org/10.3390/molecules27207073.

139. Y. Li, Z. Ni, Z. Wang and J. Feng, “In-Situ Construction of Ag-Doped ZnSe Nanodots Anchored on MXene at Room Temperature for Boosted Polysulfide Conversion Kinetics,” *Chemical Engineering Journal* 527 (2026): 171824, https://doi.org/10.1016/j.cej.2025.171824.

140. M. Zhen, X. Meng, X. Wang, Z. Zhang, S.-Q. Guo, Z. Hu and B. Shen, “Layered Double Hydroxides-MXene Heterointerfaces with Abundant Anion Vacancies Expediting Sulfur Redox Kinetics for High‑Performance Lithium-Sulfur Batteries,” *Chemical Engineering Journal* 489 (2024): 151285, https://doi.org/10.1016/j.cej.2024.151285.

141. J. Feng, W. Liu, C. Shi, C. Zhang, X. Zhao, T. Wang, S. Chen, Q. Li and J. Song, “Enabling Fast Diffusion/Conversion Kinetics by Thiourea-Induced Wrinkled N, S Co-Doped Functional MXene for Lithium-Sulfur Battery,” *Energy Storage Materials* 67 (2024): 103328, https://doi.org/10.1016/j.ensm.2024.103328.

142. W. Yu, S. Ma, M. He, R. Li, H. Yang, Y. Li, G. Yin and P. Zuo, “Immobilization and Kinetic Acceleration of Lithium Polysulfides by Iodine-Doped MXene Nanosheets in Lithium-Sulfur Batteries,” *The Journal of Physical Chemistry C* 126 (2022): 10986-10994, https://doi.org/10.1021/acs.jpcc.2c02689.

143. Q. Wang, B. Deng, X. Zhang, L. Cao, K. Wang, W. Yao, C. Chen, H. Zhao and J. Xu, “Partially Oxidized Ti_3-y_NB_y_C_2_T_x_ Mxene Nanosheets as Efficient Sulfur Hosts and Separator Modification for Li-S Batteries,” *Journal of Materiomics* 11 (2025): 100920, https://doi.org/10.1016/j.jmat.2024.07.005.

144. Y. , Y. Zhao, Y. Zhang, Z. He, G. Liu, J. Li, C. Liang and Q. Li, “Building Flexibly Porous Conductive Skeleton Inlaid with Surface Oxygen-Dominated MXene as an Amphiphilic Nanoreactor for Stable Li-S Pouch Batteries,” *Energy Storage Materials* 47 (2022): 434-444, https://doi.org/10.1016/j.ensm.2022.02.006.

145. X. Zhang, Z. Ni, X. Bai, H. Shen, Z. Wang, C. Wei, K. Tian, B. Xi, S. Xiong and J. Feng, “Hierarchical Porous N-Doped Carbon Encapsulated Fluorine-Free MXene with Tunable Coordination Chemistry by One-Pot Etching Strategy for Lithium-Sulfur Batteries,” *Advanced Energy Materials* 13 (2023): 2301349, https://doi.org/10.1002/aenm.202301349.

146. H. Gu, W. Yue, J. Hu, X. Niu, H. Tang, F. Qin, Y. Li, Q. Yan, X. Liu, W. Xu, Z. Sun, Q. Liu, W. Yan, L. Zheng, Y. Wang, H. Wang, X. Li, L. Zhang, G. Xia and W. Chen, “Asymmetrically Coordinated Cu-N_1_C_2_ Single-Atom Catalyst Immobilized on Ti_3_C_2_T_x_ Mxene as Separator Coating for Lithium-Sulfur Batteries,” *Advanced Energy Materials* 13 (2023): 2204014, https://doi.org/10.1002/aenm.202204014.

147. M. Zhen, Z. Zhang, X. Du, Y. Fan, H. Li, S.Q. Guo and T. Ma, “Atomic Environment Engineering of Cobalt Single Atom‐Nanocluster Synergistic Sites on Nitrogen‐Doped Mxene for Bidirectional Sulfur Electrocatalysis and Uniform Lithium Deposition,” *Advanced Functional Materials* 169 1 (2025): https://doi.org/10.1002/adfm.202529884.

148. S. Jing, S. Li, X. Peng, L. Yuan, S. Lu, Y. Zhang and H. Fan, “Highly Stable and Active Cerium Single-Atom Immobilized Ti_3_C_2_T_X_ MXene for High-Stability Lithium‑Sulfur Batteries,” *Chemical Engineering Journal* 521 (2025): 166774, https://doi.org/10.1016/j.cej.2025.166774.

149. J. Yu, Y. Jia, Q. Jin, L. Bai, H. Gao and J. Yao, “Bimetallic Sulfide Based on Mxene as a Multifunctional Separator for Lithium Sulfur Batteries,” *Journal of Alloys and Compounds* 1039 (2025): 183265, https://doi.org/10.1016/j.jallcom.2025.183265.

150. A. Mirtaleb, M. Scekic and R. Wang, “Synergistic SnSe2@Ti_3_C_2_T_x_ MXene Heterostructured Separator for Highly Efficient Polysulfide Adsorption, Catalytic Redox Acceleration, and Enhanced Electrochemical Stability in Advanced Lithium-Sulfur Batteries,” *Energy & Environmental Materials* 9 (2026): e70173, https://doi.org/10.1002/eem2.70173.

151. S. Deng, W. Sun, J. Tang, M. Jafarpour, F. Nüesch, J. Heier and C. Zhang, “Multifunctional SnO_2_ QDs/MXene Heterostructures as Laminar Interlayers for Improved Polysulfide Conversion and Lithium Plating Behavior,” *Nano-Micro Letters* 16 (2024): 229, https://doi.org/10.1007/s40820-024-01446-w.

152. J. Xu, C. Ma, J. Wang, W. Qiao and L. Ling, “1T-VSe_2_@Ti_3_C_2_ Heterostructure with a Built-in Electric Field and Li_x_VSe_2_ Intercalation-Conversion for High-Performance Li-S Chemistry: Accelerating Sulfur Conversion and Lithium-Ion Transport,” *ACS Applied Energy Materials* 8 (2025): 7247-7256, https://doi.org/10.1021/acsaem.5c00633.

153. M. Shen, Y. Liu, X. Gao, X. Liu, Y. Zhao, L. Hou and C. Yuan, “Eutectoid Reaction Enabling in Situ Construction of Titanium Based Ternary Heterostructures for Boosted Lithium Polysulfide Conversion,” *Small Methods* 9 (2025): 2500157, https://doi.org/10.1002/smtd.202500157.

154. C. Fu, D. He, X. Liu, W. Gu, Y. Qin, J. Lu, C. Wang and T. Wang, “MXene/ZIF-67-Based Janus Separator for High-Performance Lithium-Sulphur Batteries,” *Journal of Alloys and Compounds* 1037 (2025): 182305, https://doi.org/10.1016/j.jallcom.2025.182305.

155. Y. Ren, L. Hu, S. Chang, Y. Ma, B. Wang, H. Wu, F. Li, Y. Yang, S. Tang and X. Meng, “MXene-Bimetallic Hybrids Via Mixed Molten Salts Etching for Kinetics-Enhanced and Dendrite-Free Lithium-Sulfur Batteries,” *Small* 20 (2024): 2400068, https://doi.org/10.1002/smll.202400068.

156. Á. Bonilla, A. Gentile, C. Ferrara, F. Picciacchia, J.L. Gómez-Cámer, S. Tosoni, Á. Caballero and R. Ruffo, “Dual Role of Ti_3_C_2_T_x_ MXene in Li-S Batteries: Cathode Additive and Separator Modifier for Enhanced Performance,” *Advanced Functional Materials* (2025): e19742, https://doi.org/10.1002/adfm.202519742.

157. K. Yang, F. Zhao, J. Li, H. Yang, Y. Wang and Y. He, “Designing Organic-Inorganic Hybrid Materials to Construct a Complementary Interface with Versatility for Li-S Batteries,” *Advanced Functional Materials* 34 (2024): 2410236, https://doi.org/10.1002/adfm.202410236.
